# Computational Studies of Dimerization of [*n*]-Cyclacenes

**DOI:** 10.1021/acs.jpca.4c02833

**Published:** 2024-08-12

**Authors:** Ankit Somani, Divanshu Gupta, Holger F. Bettinger

**Affiliations:** Institut für Organische Chemie, Eberhard Karls Universität Tübingen, Auf der Morgenstelle 18, Tübingen 72076, Germany

## Abstract

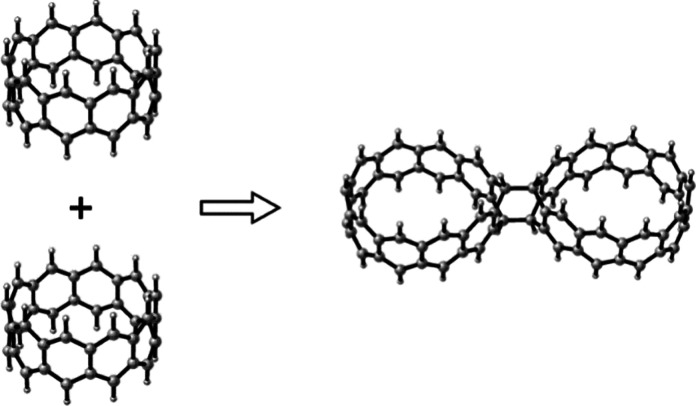

Cyclacenes, C_4*n*_H_2*n*_, consist of *n* linearly fused benzene rings that are arranged to result in a closed nanohoop structure. Cyclacenes are thus the cyclic versions of acenes and have so far escaped synthesis. In order to estimate the tendency of [*n*]-cyclacenes (6 ≤ *n* ≤ 20) to undergo dimerization, which is assumed to be a major pathway of degradation under oxygen-free conditions, we here report the energy of dimerization as computed by density functional theory using spin-restricted, spin-unrestricted, and thermally assisted-occupation (TAO) formalisms. It is found that the energy of dimerization increases with increasing size of *n* but that this increase is not monotonic for the smaller members of the series. This is due to the combination of the cryptoannulenic effect and the inherent strain of the cyclacenes. The energy of dimerization of the largest member inspected, [20]-cyclacene, is −59.3 kcal/mol, while we expect it to converge to −46 kcal/mol for *n* → ∞ based on comparison with data obtained for acenes.

## Introduction

**Figure 1:**
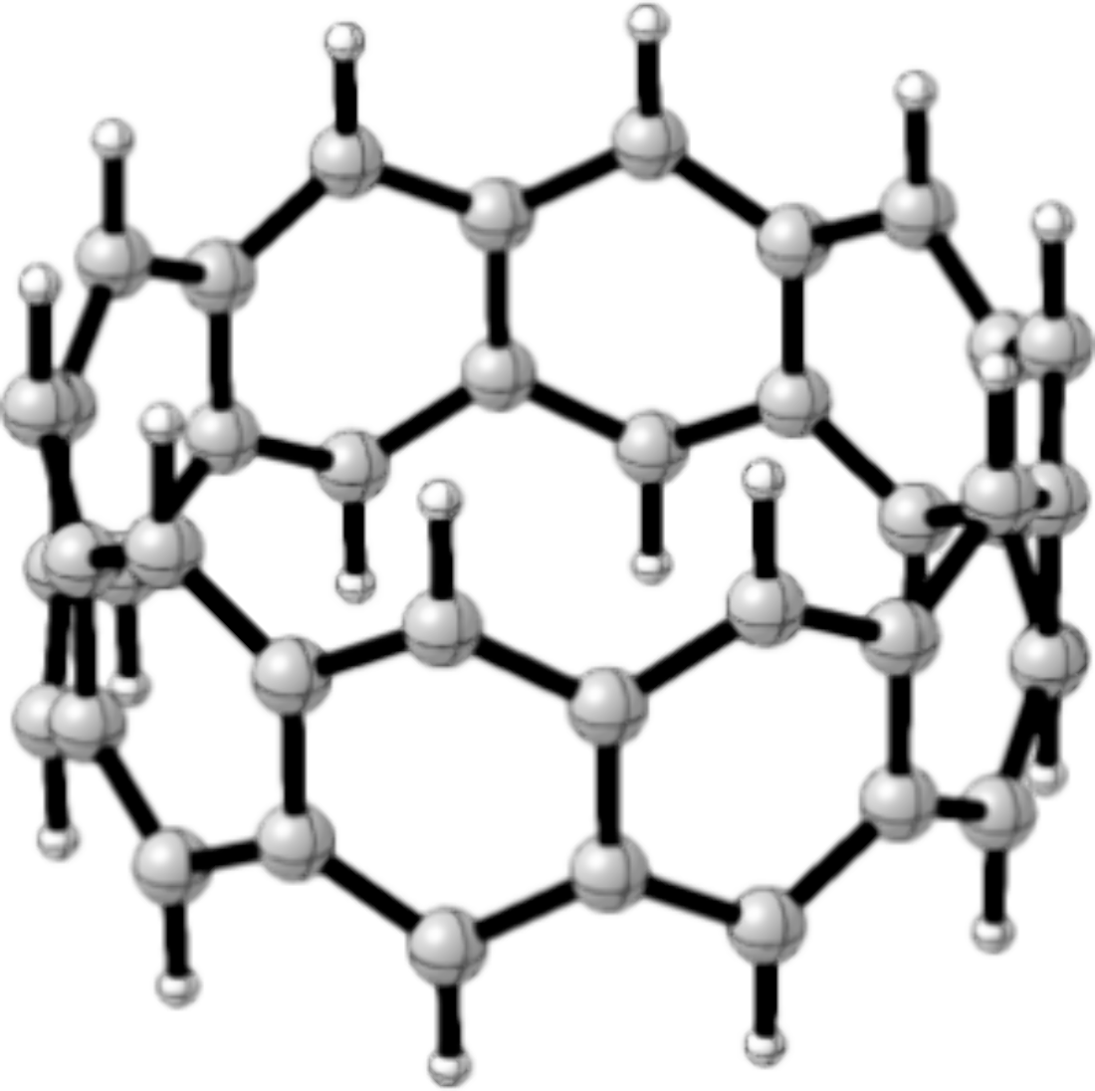
Structure of [10]-cyclacene.

Cyclacenes of the general formula C_4*n*_H_2*n*_ are fascinating carbon-based molecules that have intrigued chemists due to their distinctive structural and electronic properties.^1^ Cyclacenes belong to the family of polycyclic aromatic hydrocarbons (PAHs) and are characterized by their closed-loop, annulated carbon frameworks, reminiscent of zigzag carbon nanotubes (Figure 1).^2^ Heilbronner introduced the concept of the hoop-shaped structures of cyclacenes in 1954.^3^ Cyclacenes exhibit potential in electronics due to their tunable electronic properties, making them a viable candidate for integration into organic semiconductors used in transistors.^4^ The highly strained structures of [*n*]-cyclacenes and expected high reactivity present a significant challenge to their synthesis, resulting in multiple unsuccessful attempts to produce [*n*]-cyclacenes.^2,5^ These organic compounds are expected to have a strong tendency to react with the environment due to the lack of a Clar sextet and their diradical (or polyradical) character.^6,7^

Despite the difficulty in directly synthesizing [*n*]-cyclacenes, previous research has reported the synthesis of precursor molecules and derivatives.^2,8−12^ Notable contributions include seminal work by Stoddart et al.^5,13−16^ toward [12]-cyclacene, efforts by Cory et al.^17,18^ toward [8]-cyclacene, and investigations by Schlüter et al.^19,20^ into [18]-cyclacene. More recently, Itami et al. have described a breakthrough in the synthesis of carbon nanobelts, including [12]-, [16]-, and [24]-membered rings, which exhibit linearly and angularly fused sections.^21,22^ While not strictly [*n*]-cyclacenes, these carbon nanobelts represent an exciting step forward in pursuing cyclacene-like structures, offering new insights into their synthetic pathways. Wang and co-workers reported formation of [8]-cyclacene by retro-Diels–Alder reaction of a [8]-cyclacene derivative upon laser irradiation under mass spectrometry conditions.^23^ On-surface generation of [12]-cyclacene, attempted by Gross, Peña et al. was not successful.^65^

Cyclacenes are closely related to acenes, which are, unless in the solid state,^24,25^ notoriously reactive for systems larger than pentacene.^26^ In the absence of oxygen, which reacts with acenes initially by endoperoxide formation, the major pathway of decomposition is dimerization or oligomerization.^24,27−29^ This decomposition pathway is also expected to be dominant for the yet-unknown cyclacenes.

In this study, we computationally explore the thermochemistry of cyclacene dimerization with a focus on delineating the impact of cyclacene size on the tendency to dimerize. Due to strong static correlation effects,^30−34^ traditional electronic structure methods may encounter significant challenges when applied to *n*-cyclacenes.^35^ We here employ and compare semilocal and hybrid Kohn–Sham density functional theory (KS-DFT) methods^36^ with thermally assisted-occupation density functional theory (TAO–DFT).^31,37−40^ TAO–DFT efficiently manages large systems with strong static correlation effects by utilizing fractional orbital occupations and incorporating an entropy contribution that effectively reduces the total energy in multireference systems.

## Methods

All the structures were fully optimized using density functional theory^41,42^ (DFT) with the M06-2X^43^ global hybrid functional, as well as the B3LYP^44,45^ hybrid exchange-correlation energy functional along with Grimme’s^46^ London dispersion correction with Becke–Johnson damping B3LYP-D3(BJ).^47^ The 6-31G(d) basis set was adopted for all geometry optimizations.^48^ Harmonic vibrational frequencies were computed analytically, which confirmed the nature of the stationary points as minima. These computations were performed with Gaussian 16.^49^ The M06-2X method was employed previously by Bendikov et al.^29^ in their investigation of acene dimerization and oligomerization, thus allowing direct comparison with their data.

Initial geometries of cyclacenes were provided with the highest symmetry possible, where the hydrogen atoms are placed perpendicular to the plane that passes through all of the rung bonds of cyclacenes. The geometries after optimization at the B3LYP-D3(BJ)/6-31G(d) level of theory have *D*_*nh*_ symmetry for even *n* [*n*]-cyclacenes, except for [20]-cyclacene where *C*_1_ symmetry is observed when a spin-unrestricted approach is used. For odd *n* [*n*]-cyclacenes, either *C*_2*v*_ or *C*_1_ symmetry is obtained. Similar results were obtained in the case of M06-2X with some exceptions (Supporting Information). These results indicate that the even *n* [*n*]-cyclacenes possess a delocalized structure resulting in equal lengths of the perimeter bonds and all rung bonds. In contrast, a localized structure is favored for odd *n* [*n*]-cyclacenes, leading to bond-length alternation, as discussed by Choi and Kim.^50^ Our results slightly differ from theirs as they obtained *D*_*nh*_ symmetry for *n* ≤ 14. The optimized dimer product always had *D*_2*h*_ symmetry, except for [19]-cyclacene where *D*_2_ symmetry was observed at the UB3LYP level of theory.

We also performed TAO–DFT calculations with Q-Chem 5.2,^51^ acquiring the numerical grid containing 75 radial points in the Euler–Maclaurin quadrature and 302 angular points in the Lebedev grid. Geometries optimized at the UB3LYP-D3(BJ)/6-31G(d) level of theory were used for calculating single-point energies at the TAO-PBE/6-31G(d) level of theory. The general gradient approximation (GGA) functional introduced by Perdew, Burke, and Ernzerhof (PBE) in its TAO implementation was employed in this study.^37^ For comparison, also UPBE/6-31G(d)//UB3LYP/6-31(d) computations were performed in this study.

## Results and Discussion

### Dimerization Energy of [*n*]-Cyclacenes

To determine the dimerization energies of [*n*]-cyclacenes, we considered cyclacenes ranging from 6 to 20 rings. We optimized the geometries of the [*n*]-cyclacene and its corresponding dimer product, and the zero-point energy (ZPE)-corrected dimerization energy was obtained according to eq 1.
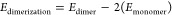
1

The dimerization energies obtained for [*n*]-cyclacenes using spin-restricted and spin-unrestricted treatment with the B3LYP and M06-2X functionals are significantly negative, which indicates that the dimerization of cyclacenes is a highly exothermic reaction (Table S1).

**Figure 2:**
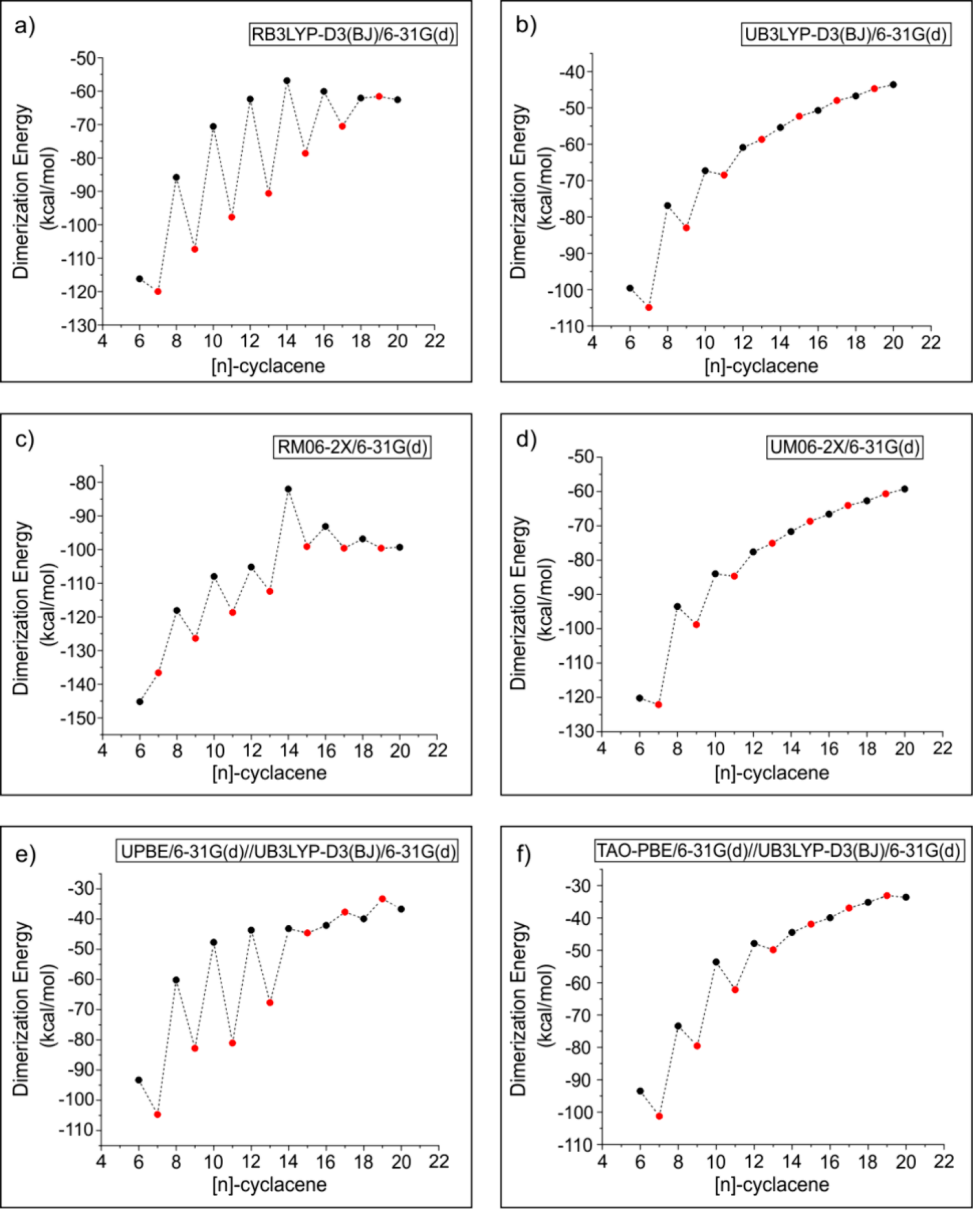
Dimerization energies for cyclacenes calculated at (a) RB3LYP-D3(BJ)/6-31G(d) + ZPE, (b) UB3LYP-D3(BJ)/6-31(d) + ZPE, (c) RM06-2*X*/6-31G(d) + ZPE, (d) UM06-2*X*/6-31G(d) + ZPE, (e) UPBE/6-31G(d)//UB3LYP-D3(BJ)/6-31G(d), and (f) the TAO-PBE/6-31G(d)//UB3LYP-D3BJ/6-31(d) level of theory as a function of the number of fused benzene rings. Dimerization energies are marked with black dots and red dots for even *n* and odd *n* [*n*]-cyclacenes, respectively.

The dimerization energies of [*n*]-cycalcenes computed using the spin-unrestricted wave function are consistently less exothermic than those calculated using the restricted wave function (Figure 2). This difference arises from the consideration of open-shell structures with biradical character.^30^

We split our discussion into two categories: spin-restricted and spin-unrestricted solutions. As shown in Figure 2a,c, the dimerization energies calculated at the RB3LYP-D3(BJ)/6-31G(d) and RM06-2X/6-31G(d) levels of theory exhibit an oscillatory pattern with an increasing number of fused rings. The dimerization reaction of even-numbered cyclacenes is less exothermic than that of cyclacenes with similar sizes but odd numbers of rings. However, the dimerization reaction is more exothermic for [6]-cyclacene than [7]-cyclacene at the RM06-2X/6-31G(d) level of theory. The dimerization of even *n* [*n*]-cyclacenes, starting from [6]-cyclacene, becomes drastically less exothermic with increasing system size, peaking at [14]-cyclacene. Subsequently, the reaction becomes more exothermic. In contrast, the dimerization energies for odd cyclacenes increase monotonically. As a result, the dimerization energy values of the smallest ([6]- and [7]-cyclacenes: −116.2 and −120.0 kcal/mol) and the largest ([19]- and [20]-cyclacenes: −61.6 and −62.6 kcal/mol) systems investigated are close (Table S1). However, the differences between the two sets are much larger. For example, the dimerization energy of the next even-numbered [8]-cyclacene is −85.8 kcal/mol, slightly less exothermic than that of [13]-cyclacene (−90.6 kcal/mol). For [10]-cyclacene, the dimerization energy increases to −70.6 kcal/mol, similar to that of [17]-cyclacene (−70.5 kcal/mol) (Table S1). Initially, the oscillatory behavior of dimerization energies increases with size until [14]-cyclacene; afterward, it is significantly damped (Figure 2a,c).

Similar to the situation observed in larger acenes, where open-shell treatment leads to improved outcomes attributed to the emerging polyradical character,^29,52^ we also performed broken-symmetry calculations for cyclacenes and their dimers.^30^ In contrast to the spin-restricted solutions, we observed an oscillatory pattern in the dimerization energies only for the smaller cyclacenes (*n* ≤ 11) (Figure 2b,d). For larger cyclacenes ranging from 12 to 20, we obtained a monotonic change in the dimerization energy, indicating that the dimerization process becomes less exothermic with increasing size. Additionally, the dimerization energy values were observed to approach a limit as the size of the cyclacene increases.

The dimerization energies obtained at the TAO-PBE level of theory (Figure 2f) show a pattern similar to that observed at the UB3LYP/6-31G(d) level of theory (Figure 2b). However, the oscillations are more pronounced and extend up to *n* = 12 while the dimerization energies are consistently less exothermic upon employing the TAO-PBE/6-31G(d) level of theory (Table S1). On the other hand, single-point dimerization energies calculated at the UPBE/6-31G(d)//UB3LYP-D3(BJ)/6-31G(d) level of theory show more fluctuation, also for the larger [*n*]-cyclacenes (Figure 2e). For a comparison of single-point dimerization energies calculated using various functionals, the reader is referred to Figure S1. Note that for *n* = 10 and *n* = 12, the cyclacenes, and for *n* = 6 and *n* = 7, the dimers do not give the symmetry-broken solutions at the PBE/6-31G(d)//UB3LYP-D3(BJ)/6-31G(d) level of theory.

The oscillatory behavior of dimerization energies with the [*n*]-cyclacene size was similarly observed before for the singlet–triplet gap and the heat of formation.^1,31^ This phenomenon has been elucidated within the framework of the cryptoannulenic effect.^53−56^ Cyclacene structures without Clar’s sextet imply that the system should consist of two (the top and bottom) peripheral circuits. These peripheral circuits can be seen as two trannulenes connected through a C–C rung bond.^57^ An [*n*]-cyclacene in this sense consists of two fused [2n]-trannulenes. These [2n]-trannulenes, or peripheral circuits, can be divided into two categories: 2n = 4k and 2n = 4k+2. The [2n]-trannulene units of even-numbered cyclacenes belong to the 4k type, whereas those of odd-numbered [*n*]-cyclacenes belong to the 4k+2-type trannulene. As Schleyer and co-workers have demonstrated, trannulenes follow the Hückel rule perfectly: 4k+2 trannulenes are aromatic, while 4k trannulene species are antiaromatic.^58^ Choi and Kim^50^ have demonstrated that even-numbered [*n*]-cyclacenes exhibit greater stability in terms of building unit energy, shorter C–C bond lengths, smaller bond-length alternations, and significantly more negative magnetic property values such as magnetic susceptibility, magnetic susceptibility exaltation, and nucleus-independent chemical shift (NICS), indicating aromaticity. Conversely, for odd *n* [*n*]-cyclacenes, a comparison of the magnetic values with [2n]-trannulenes reveals that they are almost nonaromatic due to the cancellation of the aromaticity of [4k+2] trannulene moieties based on magnetic criteria. Furthermore, Su and co-workers investigated the aromaticity of [5]- to [10]-cyclacene.^59^ Computed NICS values and anisotropy of the current-induced density (ACID) diagrams revealed that both cyclacenes are aromatic, despite being open-shell molecules, as confirmed by the presence of diatropic current in two individual [2n]-trannulene units.^59^ The NICS values at the center of the even *n* [*n*]-cyclacenes are more negative (−34 to −31 for *n* = 6 to 10) than those of odd *n* [*n*]-cyclacenes (−8 to −4 for *n* = 5 to 9). If the increased aromaticity of even *n* [*n*]-cyclacenes based on magnetic criteria also causes increased thermodynamic stability, then the dimerization process of cyclacenes with similar sizes exhibits greater exothermicity for odd cyclacenes compared to even ones.

Apart from the cryptoannulenic effect, the strain energy is expected to play a crucial role in the stability and reactivity of cyclacenes. Several theoretical studies have analyzed cyclacene strain, which demonstrates that the strain energy decreases with the increasing size of [*n*]-cyclacene.^34,60−64^ For example, at the B3LYP/6-31G(d) level of theory, the strain amounts to 1324.3 × *n*^–1^ kcal/mol, which translates to 221 kcal/mol for [6]-cyclacene and 66 kcal/mol for [20]-cyclacene.^64^ Therefore, smaller cyclacenes with higher strain exhibit greater reactivity, resulting in higher exothermicity.

### Comparison with Acene Dimerization

Acenes are a class of PAHs, composed of linearly fused benzene rings with the general formula C_4*n*+2_H_2*n*+4_. In the limit of infinite size, cyclacenes become indistinguishable from acenes. Therefore, it is interesting to compare the dimerization energies of cyclacenes to those of acenes.

Bendikov and co-workers computationally investigated the thermal dimerization of [*n*]-acenes for values of *n* ranging from 1 to 9.^29^ The dimerization process was examined at the most reactive central rings. At the M06-2X/6-31G(d) + ZPE level of theory, dimerization is endothermic for *n* = 1 and 2, whereas, from anthracene onward, it becomes exothermic. For acenes longer than hexacene, the dimerization computed using the spin-unrestricted wave functions were observed always more exothermic than those obtained using the spin-restricted wave function similar to the case of cyclacenes observed in our study. However, for acenes, the dimerization energy becomes more exothermic with increasing acene length, unlike the case of cyclacens, where it becomes less exothermic with an increase in size of cyclacenes. This is because the reactivity of acenes^26^ increases with length, whereas in the case of cyclacenes, we expect it to decrease due to the decrease in strain energy. The dimerization energy calculated at UM06-2X/6-31G(d) + ZPE for acenes longer than heptacene was observed to remain constant at approximately −46 kcal/mol.^29^ In our case, for [20]-cyclacene, the dimerization energy calculated at the same level of theory is −59.3 kcal/mol while we expect it to converge to −46 kcal/mol for *n* → ∞.

## Conclusions

Our computational investigations reveal that the dimerization of [*n*]-cyclacene (for 6 ≤ *n* ≤ 20) is a highly exothermic process, implying a highly reactive nature of [*n*]-cyclacenes despite their aromatic character identified by earlier studies based on magnetic criteria.^50,59^ The dimerization energies, calculated using the spin-restricted wave function, are influenced by both the cryptoannulenic effect and strain. However, the cryptoannulenic effect emerges as the dominant factor, leading to the observed oscillatory behavior in the dimerization energy values in a spin-restricted treatment. Conversely, in the case of spin-unrestricted calculations, the cryptoannulenic effect predominates for smaller cyclacenes (*n* ≤ 10), after which the strain energy becomes the determining factor. As a result, we observe a decrease in the exothermicity for dimerization as the cyclacene size increases. The TAO-PBE description of the dimerization process is qualitatively similar to UB3LYP but differs significantly from that of UPBE.

## Supporting Information Available

Plot of dimerization energies (UB3LYP, UPBE, and TAO-PBE) in a single graph, dimerization energies, total energies, and Cartesian coordinates (PDF)
