# Supplementary material for: Computational Studies of Dimerization of [n]-Cyclacenes
Source: J Phys Chem A. 2024 Aug 12;128(33):6847–52. doi: 10.1021/acs.jpca.4c02833 (PMC11345815; doi:10.1021/acs.jpca.4c02833)
Supplement: Supplementary file 1 — jp4c02833_si_001.pdf [file jp4c02833_si_001.pdf]

Supporting Information

Computational Studies of Dimerization of [n]-Cyclacenes

Ankit Somani, Divanshu Gupta, and Holger F. Bettinger*

Institut für Organische Chemie,

Eberhard Karls Universität Tübingen

Auf der Morgenstelle 18, 72076, Tübingen, Germany

E-mail: holger.bettinger@uni-tuebingen.de

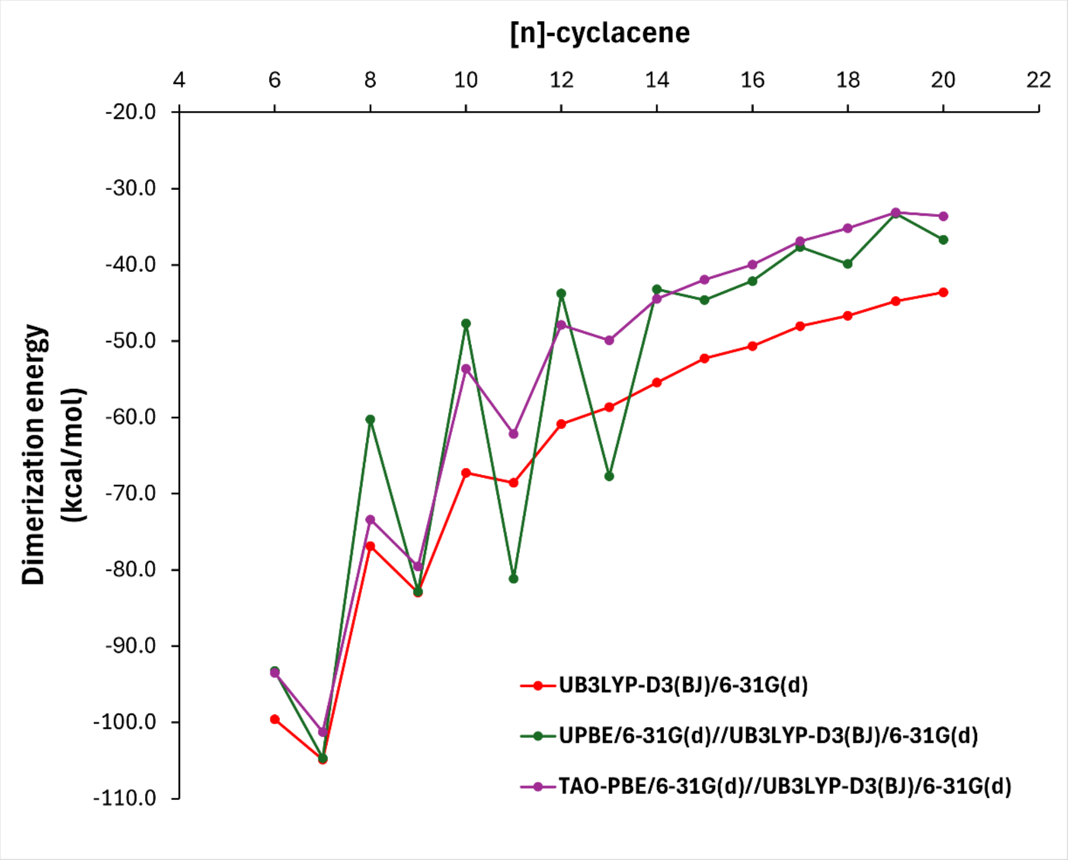

Figure S1. Dimerization energies for cyclacenes calculated at UB3LYP-D3(BJ)/6-31G(d) + ZPE, UPBE/6-31G(d)//UB3LYP-D3(BJ)/6-31(d), and TAO-PBE/6-31G(d)//UB3LYP-D3BJ/6-31(d) level of theory as a function of the number of fused benzene rings.

Table S1. Calculated dimerization energies (kcal/mol) at different functional using 6-31G(d) basis-set for [n]-cyclacene ranging between 6 to 20.

[n]	RB3LYP-D3(BJ) /6-31G(d)	UB3LYP-D3(BJ) /6-31G(d)	RM06-2X/6-31G(d)	UM06-2X/6-31G(d)	UPBE/6-31G(d)// UB3LYP-D3BJ/6-31G(d)[a]	TAO-PBE6-31G(d)// UB3LYP-D3BJ/6-31G(d)[a]
6	-116.2	-99.6	-145.2	-120.2	-93.3	-93.5
7	-120.0	-104.9	-136.6	-122.1	-104.7	-101.3
8	-85.8	-76.9	-118.1	-93.5	-60.2	-73.4
9	-107.3	-83.0	-126.4	-98.8	-82.8	-79.5
10	-70.6	-67.3	-108.0	-84.0	-47.7	-53.6
11	-97.7	-68.5	-118.7	-84.7	-81.1	-62.2
12	-62.4	-60.9	-105.2	-77.6	-43.7	-47.9
13	-90.6	-58.7	-112.4	-75.1	-67.7	-49.9
14	-56.9	-55.4	-82.0	-71.7	-43.2	-44.5
15	-78.6	-52.3	-99.1	-68.7	-44.6	-41.9
16	-60.1	-50.7	-93.1	-66.6	-42.1	-40.0
17	-70.5	-48.0	-99.6	-64.1	-37.7	-36.9
18	-62.1	-46.7	-96.8	-62.7	-39.9	-35.2
19	-61.6	-44.7	-99.6	-60.7	-33.3	-33.1
20	-62.6	-43.6	-99.3	-59.3	-36.7	-33.6

[a] = single-point energies without ZPE

Table S2. Calculated zero-point corrected energies (ZPE) of [n]-cyclacene (for 6 ≤ n ≤ 20), their corresponding dimers, and relative dimerization energies (kcal/mol) at B3LYP-D3(BJ)/6-31G(d) level of theory. The values in bold belong to RB3LYP and the values in parenthesis belong to the spin-unrestricted solution (UB3LYP).

[n]-cyclacene	Symmetry	E+ZPE (Hartree)	E+ZPE (Dimer) (Hartree)	Dimerization Energy (kcal/mol)
6	$\boldsymbol{D_{6h}}$ (D_{6h})	**-921.3036** *(-921.3177)*	**-1842.7924** *(-1842.7941)*	**-116.2** *(-99.6)*
7	$\boldsymbol{C_{2v}}$ (C_{2v})	**-1074.9456** *(-1074.9600)*	**-2150.0824** *(-2150.0871)*	**-120.0** *(-104.9)*
8	$\boldsymbol{D_{8h}}$ (D_{8h})	**-1228.6048** *(-1228.6182)*	**-2457.3464** *(-2457.3589)*	**-85.8** *(-76.9)*
9	$\boldsymbol{C_{2v}}$ (C_1)	**-1382.2149** *(-1382.2432)*	**-2764.6008** *(-2764.6186)*	**-107.3** *(-83.0)*
10	$\boldsymbol{D_{10h}}$ (D_{10h})	**-1535.8656** *(-1535.8819)*	**-3071.8437** *(-3071.8710)*	**-70.6** *(-67.3)*
11	$\boldsymbol{C_{2v}}$ (C_{2v})	**-1689.4631** *(-1689.5031)*	**-3379.0819** *(-3379.1153)*	**-97.7** *(-68.5)*
12	$\boldsymbol{D_{12h}}$ (D_{12h})	**-1843.1073** *(-1843.1296)*	**-3686.3141** *(-3686.3562)*	**-62.4** *(-60.9)*
13	$\boldsymbol{C_{2v}}$ (C_{2v})	**-1996.6995** *(-1996.7493)*	**-3993.5433** *(-3993.5921)*	**-90.6** *(-58.7)*
14	$\boldsymbol{D_{14h}}$ (D_{14h})	**-2150.3388** *(-2150.3689)*	**-4300.7683** *(-4300.8261)*	**-56.9** *(-55.4)*
15	$\boldsymbol{C_{2v}}$ (C_{2v})	**-2303.9355** *(-2303.9870)*	**-4607.9962** *(-4608.0573)*	**-78.6** *(-52.3)*
16	$\boldsymbol{D_{16h}}$ (D_{16h})	**-2457.5642** *(-2457.6031)*	**-4915.2242** *(-4915.2870)*	**-60.1** *(-50.7)*
17	$\boldsymbol{C_{2v}}$ (C_{2v})	**-2611.1675** *(-2611.2193)*	**-5222.4473** *(-5222.5151)*	**-70.5** *(-48.0)*
18	$\boldsymbol{D_{18h}}$ (D_{18h})	**-2764.7858** *(-2764.8337)*	**-5529.6705** *(-5529.7418)*	**-62.1** *(-46.7)*
19	$\boldsymbol{C_{2v}}$ (C_{2v})	**-2918.3961** *(-2918.4482)*	**-5836.8904** *(-5836.9677)*	**-61.6** *(-44.7)*
20	$\boldsymbol{D_{20h}}$ (C_1)	**-3072.0052** *(-3072.0615)*	**-6144.1102** *(-6144.1925)*	**-62.6** *(-43.6)*

Table S3. Calculated Gibbs free energies (298.15 K) (G) of [n]-cyclacene (for 6 ≤ n ≤ 20), their corresponding dimers, and relative Gibbs free (ΔG) dimerization energies (kcal/mol) at B3LYP-D3(BJ)/6-31G(d) level of theory. The values in bold belong to RB3LYP and the values in parenthesis belong to the spin-unrestricted solution (UB3LYP).

[n]-cyclacene	Symmetry	G (Hartree)	G (Dimer) (Hartree)	(ΔG) (kcal/mol)
6	$\boldsymbol{D_{6h}}$<br>(D_{6h})	**-921.3397**<br>*(-921.3539)*	**-1842.8427**<br>*(-1842.8446)*	**-102.5**<br>*(-85.9)*
7	$\boldsymbol{C_{2v}}$<br>(C_{2v})	**-1074.9857**<br>*(-1075.0012)*	**-2150.1381**<br>*(-2150.1431)*	**-104.6**<br>*(-88.3)*
8	$\boldsymbol{D_{8h}}$<br>(D_{8h})	**-1228.6457**<br>*(-1228.6592)*	**-2457.4076**<br>*(-2457.4204)*	**-72.9**<br>*(-64.0)*
9	$\boldsymbol{C_{2v}}$<br>(C_1)	**-1382.2606**<br>*(-1382.2897)*	**-2764.6676**<br>*(-2764.6857)*	**-91.9**<br>*(-66.7)*
10	$\boldsymbol{D_{10h}}$<br>(D_{10h})	**-1535.9118**<br>*(-1535.9284)*	**-3071.9162**<br>*(-3071.9438)*	**-58.1**<br>*(-54.6)*
11	$\boldsymbol{C_{2v}}$<br>(C_{2v})	**-1689.5148**<br>*(-1689.5553)*	**-3379.1603**<br>*(-3379.1939)*	**-82.0**<br>*(-52.3)*
12	$\boldsymbol{D_{12h}}$<br>(D_{12h})	**-1843.1593**<br>*(-1843.1817)*	**-3686.3984**<br>*(-3686.4407)*	**-50.1**<br>*(-48.5)*
13	$\boldsymbol{C_{2v}}$<br>(C_{2v})	**-1996.7577**<br>*(-1996.8074)*	**-3993.6336**<br>*(-3993.6826)*	**-74.2**<br>*(-42.5)*
14	$\boldsymbol{D_{14h}}$<br>(D_{14h})	**-2150.3967**<br>*(-2150.4270)*	**-4300.8648**<br>*(-4300.9226)*	**-44.8**<br>*(-43.0)*
15	$\boldsymbol{C_{2v}}$<br>(C_{2v})	**-2303.9997**<br>*(-2303.0512)*	**-4608.0988**<br>*(-4608.1600)*	**-62.4**<br>*(-36.1)*
16	$\boldsymbol{D_{16h}}$<br>(D_{16h})	**-2457.6280**<br>*(-2457.6671)*	**-4915.3330**<br>*(-4915.3958)*	**-48.3**<br>*(-38.7)*
17	$\boldsymbol{C_{2v}}$<br>(C_{2v})	**-2611.2379**<br>*(-2611.2897)*	**-5222.5622**<br>*(-5222.6300)*	**-54.2**<br>*(-31.8)*
18	$\boldsymbol{D_{18h}}$<br>(D_{18h})	**-2764.8560**<br>*(-2764.9038)*	**-5529.7916**<br>*(-5529.8630)*	**-49.9**<br>*(-34.8)*
19	$\boldsymbol{C_{2v}}$<br>(C_{2v})	**-2918.4752**<br>*(-2918.5248)*	**-5836.0177**<br>*(-5837.0951)*	**-42.2**<br>*(-28.6)*
20	$\boldsymbol{D_{20h}}$<br>(C_1)	**-3072.0818**<br>*(-3072.1413)*	**-6144.2439**<br>*(-6144.3263)*	**-50.4**<br>*(-27.4)*

Table S4. Calculated zero-point corrected energies (ZPE) of [n]-cyclacene (for 6 ≤ n ≤ 20), their corresponding dimers, and relative dimerization energies (kcal/mol) at M06-2X/6-31G(d) level of theory. The values in bold belong to RM06-2X and the values in parenthesis belong to the spin-unrestricted solution (UM06-2X).

[n]-cyclacene	Symmetry	E+ZPE (Hartree)	E+ZPE (Dimer) (Hartree)	Dimerization Energy (kcal/mol)
6	***D_{6h}***<br>*(D_{6h})*	**-920.7980**<br>*(-920.8179)*	**-1841.8273**<br>*(-1841.8273)*	**-145.2**<br>*(-120.2)*
7	***C_{2v}***<br>*(C_{2v})*	**-1074.3672**<br>*(-1074.3788)*	**-2148.9521**<br>*(-2148.9521)*	**-136.6**<br>*(-122.1)*
8	***D_{8h}***<br>*(D_{8h})*	**-1227.9330**<br>*(-1227.9553)*	**-2456.0542**<br>*(-2456.0596)*	**-118.1**<br>*(-93.5)*
9	***C_{2v}***<br>*(C_{2v})*	**-1381.4720**<br>*(-1381.4995)*	**-2763.1455**<br>*(-2763.1565)*	**-126.4**<br>*(-98.8)*
10	***D_{10h}***<br>*(D_{10h})*	**-1535.0268**<br>*(-1535.0559)*	**-3070.2257**<br>*(-3070.2456)*	**-108.0**<br>*(-84.0)*
11	***C_{2v}***<br>*(C_{2v})*	**-1688.5555**<br>*(-1688.5959)*	**-3377.3001**<br>*(-3377.3268)*	**-118.7**<br>*(-84.7)*
12	***C_2***<br>*(D_{12h})*	**-1842.1005**<br>*(-1842.1400)*	**-3684.3687**<br>*(-3684.4037)*	**-105.2**<br>*(-77.6)*
13	***C_1***<br>*(C_{2v})*	**-1995.6273**<br>*(-1995.6780)*	**-3991.4337**<br>*(-3991.4757)*	**-112.4**<br>*(-75.1)*
14	***C_1***<br>*(D_{14h})*	**-2149.1650**<br>*(-2149.2155)*	**-4298.4607**<br>*(-4298.5453)*	**-82.0**<br>*(-71.7)*
15	***C_1***<br>*(C_{2v})*	**-2302.6835**<br>*(-2302.7514)*	**-4605.5250**<br>*(-4605.6122)*	**-99.1**<br>*(-68.7)*
16	***C_1***<br>*(C_1)*	**-2456.2195**<br>*(-2456.2856)*	**-4912.5873**<br>*(-4912.6774)*	**-93.1**<br>*(-66.6)*
17	***C_1***<br>*(C_{2v})*	**-2609.7439**<br>*(-2609.8194)*	**-5219.6464**<br>*(-5219.7410)*	**-99.6**<br>*(-64.1)*
18	***D_2***<br>*(C_1)*	**-2763.2748**<br>*(-2763.3517)*	**-5526.7039**<br>*(-5526.8033)*	**-96.8**<br>*(-62.7)*
19	***C_1***<br>*(C_{2v})*	**-2916.8003**<br>*(-2916.8839)*	**-5833.7594**<br>*(-5833.8646)*	**-99.6**<br>*(-60.7)*
20	***D_{20}***<br>*(D_{10d})*	**-3070.3279**<br>*(-3070.4152)*	**-6140.8140**<br>*(-6140.9249)*	**-99.3**<br>*(-59.3)*

Table S5. Calculated Gibbs free energies (298.15 K) (G) of [n]-cyclacene (for 6 ≤ n ≤ 20), their corresponding dimers, and relative Gibbs free (ΔG) dimerization energies (kcal/mol) at M06-2X/6-31G(d) level of theory. The values in bold belong to RM06-2X and the values in parenthesis belong to the spin-unrestricted solution (UM06-2X).

[n]-cyclacene	Symmetry	G (Hartree)	G (Dimer) (Hartree)	(ΔG) (kcal/mol)
6	***D_{6h}***<br>*(D_{6h})*	**-920.8340**<br>*(-920.8540)*	**-1841.8775**<br>*(-1841.8775)*	**-131.4**<br>*(-106.4)*
7	***C_{2v}***<br>*(C_{2v})*	**-1074.4072**<br>*(-1074.4053)*	**-2149.0076**<br>*(-2149.0076)*	**-121.2**<br>*(-123.6)*
8	***D_{8h}***<br>*(D_{8h})*	**-1227.9739**<br>*(-1227.9962)*	**-2456.1152**<br>*(-2456.1220)*	**-105.0**<br>*(-81.3)*
9	***C_{2v}***<br>*(C_{2v})*	**-1381.5182**<br>*(-1381.5452)*	**-2763.2120**<br>*(-2763.2232)*	**-110.2**<br>*(-83.3)*
10	***D_{10h}***<br>*(D_{10h})*	**-1535.0731**<br>*(-1535.1021)*	**-3070.2979**<br>*(-3070.3180)*	**-95.2**<br>*(-71.4)*
11	***C_{2v}***<br>*(C_{2v})*	**-1688.6068**<br>*(-1688.6479)*	**-3377.3782**<br>*(-3377.4050)*	**-103.3**<br>*(-68.5)*
12	***C_2***<br>*(D_{12h})*	**-1842.1549**<br>*(-1842.1918)*	**-3684.4526**<br>*(-3684.4877)*	**-86.6**<br>*(-65.3)*
13	***C_1***<br>*(C_{2v})*	**-1995.6851**<br>*(-1995.7358)*	**-3991.5236**<br>*(-3991.5655)*	**-96.3**<br>*(-58.9)*
14	***C_1***<br>*(D_{14h})*	**-2149.2282**<br>*(-2149.2732)*	**-4298.5570**<br>*(-4298.6412)*	**-63.1**<br>*(-59.5)*
15	***C_1***<br>*(C_{2v})*	**-2302.7477**<br>*(-2302.8153)*	**-4605.6275**<br>*(-4605.7141)*	**-82.9**<br>*(-52.4)*
16	***C_1***<br>*(C_1)*	**-2456.2866**<br>*(-2456.3526)*	**-4912.6957**<br>*(-4912.7854)*	**-76.9**<br>*(-50.3)*
17	***C_1***<br>*(C_{2v})*	**-2609.8141**<br>*(-2609.8894)*	**-5219.7610**<br>*(-5219.8552)*	**-83.3**<br>*(-47.9)*
18	***D_2***<br>*(C_1)*	**-2763.3468**<br>*(-2763.4248)*	**-5526.8246**<br>*(-5526.9235)*	**-82.2**<br>*(-46.4)*
19	***C_1***<br>*(C_{2v})*	**-2916.8769**<br>*(-2916.9595)*	**-5833.8863**<br>*(-5833.9917)*	**-83.1**<br>*(-45.6)*
20	***D_{20}***<br>*(D_{10d})*	**-3070.4041**<br>*(-3070.4917)*	**-6140.9473**<br>*(-6141.0575)*	**-87.3**<br>*(-59.3)*

Table S6. Calculated single-point energies of [n]-cyclacene (for 6 ≤ n ≤ 20), their corresponding dimers, and relative dimerization energies (kcal/mol) at the UPBE/6-31G(d)//UB3LYP-D3(BJ)/6-31G(d) level of theory.

[n]-cyclacene	Monomer (Hartree)	Dimer (Hartree)	Dimerization Energy (kcal/mol)
6	-920.3507	-1840.8500	-93.3
7	-1073.8244	-2147.8156	-104.7
8	-1227.3300	-2454.7560	-60.2
9	-1380.7787	-2761.6894	-82.8
10	-1534.2701	-3068.6162	-47.7
11	-1687.7028	-3375.5349	-81.1
12	-1841.1912	-3682.4521	-43.7
13	-1994.6271	-3989.3621	-67.7
14	-2148.1017	-4296.2722	-43.2
15	-2301.5528	-4603.1767	-44.6
16	-2454.0073	-4910.0817	-42.1
17	-2608.4616	-5216.9832	-37.7
18	-2761.9107	-5523.8849	-39.9
19	-2915.3660	-5830.7850	-33.3
20	-3068.8129	-6137.6843	-36.7

Table S7. Calculated single-point energies of [n]-cyclacene (for $6 \leq n \leq 20$), their corresponding dimers, and relative dimerization energies (kcal/mol) at the TAO-PBE/6-31G(d)//UB3LYP-D3(BJ)/6-31G(d) level of theory.

[n]-cyclacene	Monomer (Hartree)	Dimer (Hartree)	Dimerization Energy (kcal/mol)
6	-920.3349	-1840.8188	-93.5
7	-1073.8093	-2147.7800	-101.3
8	-1227.3025	-2454.7219	-73.4
9	-1380.7620	-2761.6507	-79.5
10	-1534.2447	-3068.5749	-53.6
11	-1687.6950	-3375.4891	-62.2
12	-1841.1630	-3682.4024	-47.9
13	-1994.6144	-3989.3082	-49.9
14	-2148.0713	-4296.2136	-44.5
15	-2301.5240	-4603.1148	-41.9
16	-2454.9756	-4910.0149	-40.0
17	-2608.4275	-5216.9138	-36.9
18	-2761.8777	-5523.8114	-35.2
19	-2915.3275	-5830.7077	-33.1
20	-3068.7749	-6137.6033	-33.6

Table S8. Cartesian coordinates of the optimized geometries of [n]-cyclacene at B3LYP-D3(BJ)/6-31G(d) level of theory.

RB3LYP-D3(BJ)/6-31G(d)				UB3LYP-D3(BJ)/6-31G(d)			
Cyc6_R				Cyc6_U			
C	0.00000000	2.39403100	0.72527000	C	0.00000000	2.39875200	0.72525800
C	1.20637900	2.08951000	1.40158800	C	1.20702800	2.09063400	1.40518900
C	2.07329200	1.19701600	0.72527000	C	2.07738000	1.19937600	0.72525800
C	2.07329200	1.19701600	-0.72527000	C	2.07738000	1.19937600	-0.72525800
C	1.20637900	2.08951000	-1.40158800	C	1.20702800	2.09063400	-1.40518900
C	0.00000000	2.39403100	-0.72527000	C	0.00000000	2.39875200	-0.72525800
C	2.41275800	0.00000000	1.40158800	C	2.41405600	0.00000000	1.40518900
C	2.41275800	0.00000000	-1.40158800	C	2.41405600	0.00000000	-1.40518900
C	2.07329200	-1.19701600	-0.72527000	C	2.07738000	-1.19937600	-0.72525800
C	2.07329200	-1.19701600	0.72527000	C	2.07738000	-1.19937600	0.72525800
C	1.20637900	-2.08951000	1.40158800	C	1.20702800	-2.09063400	1.40518900
H	1.18669500	-2.05541600	2.48851800	H	1.18371600	-2.05025600	2.49165500
C	0.00000000	-2.39403100	0.72527000	C	0.00000000	-2.39875200	0.72525800
C	0.00000000	-2.39403100	-0.72527000	C	0.00000000	-2.39875200	-0.72525800
C	1.20637900	-2.08951000	-1.40158800	C	1.20702800	-2.09063400	-1.40518900
H	2.37339000	0.00000000	2.48851800	H	2.36743200	0.00000000	2.49165500
H	1.18669500	2.05541600	2.48851800	H	1.18371600	2.05025600	2.49165500
H	1.18669500	2.05541600	-2.48851800	H	1.18371600	2.05025600	-2.49165500
H	2.37339000	0.00000000	-2.48851800	H	2.36743200	0.00000000	-2.49165500
H	1.18669500	-2.05541600	-2.48851800	H	1.18371600	-2.05025600	-2.49165500
C	-1.20637900	-2.08951000	1.40158800	C	-1.20702800	-2.09063400	1.40518900
C	-2.07329200	-1.19701600	0.72527000	C	-2.07738000	-1.19937600	0.72525800
C	-2.07329200	-1.19701600	-0.72527000	C	-2.07738000	-1.19937600	-0.72525800
C	-1.20637900	-2.08951000	-1.40158800	C	-1.20702800	-2.09063400	-1.40518900
C	-2.41275800	0.00000000	1.40158800	C	-2.41405600	0.00000000	1.40518900
C	-2.41275800	0.00000000	-1.40158800	C	-2.41405600	0.00000000	-1.40518900
C	-2.07329200	1.19701600	-0.72527000	C	-2.07738000	1.19937600	-0.72525800
C	-2.07329200	1.19701600	0.72527000	C	-2.07738000	1.19937600	0.72525800
C	-1.20637900	2.08951000	1.40158800	C	-1.20702800	2.09063400	1.40518900
H	-1.18669500	2.05541600	2.48851800	H	-1.18371600	2.05025600	2.49165500
C	-1.20637900	2.08951000	-1.40158800	C	-1.20702800	2.09063400	-1.40518900
H	-2.37339000	0.00000000	2.48851800	H	-2.36743200	0.00000000	2.49165500
H	-2.37339000	0.00000000	-2.48851800	H	-2.36743200	0.00000000	-2.49165500
H	-1.18669500	2.05541600	-2.48851800	H	-1.18371600	2.05025600	-2.49165500
H	-1.18669500	-2.05541600	2.48851800	H	-1.18371600	-2.05025600	2.49165500
H	-1.18669500	-2.05541600	-2.48851800	H	-1.18371600	-2.05025600	-2.49165500
Cyc7_R				Cyc7_U			
C	0.00000000	0.73509000	2.77630400	C	0.64160800	-2.70796500	0.72823300
C	1.20660300	1.41557000	2.50624300	C	1.76013800	-2.16994800	1.41011000
C	0.00000000	-0.73509000	2.77630400	C	0.64164400	-2.70792500	-0.72829800
C	-1.20660300	1.41557000	2.50624300	C	-0.59926400	-2.72893800	1.41018100
C	2.16939300	0.73506100	1.73045300	C	2.51763100	-1.18686600	0.72826600
H	1.18247300	2.50137600	2.45612000	H	1.73047500	-2.13347600	2.49641300
C	1.20660300	-1.41557000	2.50624300	C	1.76002700	-2.16992900	-1.41018400
C	-1.20660300	-1.41557000	2.50624300	C	-0.59938500	-2.72880400	-1.41021400
C	-2.16939300	0.73506100	1.73045300	C	-1.71735900	-2.19005100	0.72825800
H	-1.18247300	2.50137600	2.45612000	H	-0.58914800	-2.68289200	2.49646800
C	2.16939300	-0.73506100	1.73045300	C	2.51764400	-1.18682000	-0.72830600
C	2.71063200	1.41562000	0.61881600	C	2.79453700	0.02307700	1.41011300
H	1.18247300	-2.50137600	2.45612000	H	1.73038800	-2.13340000	-2.49648200

C	-2.16939300	-0.73506100	1.73045300	C	-1.71735400	-2.19003400	-0.72828700
H	-1.18247300	-2.50137600	2.45612000	H	-0.58921200	-2.68273900	-2.49651100
C	-2.71063200	1.41562000	0.61881600	C	-2.50758900	-1.23306900	1.41013800
C	2.71063200	-1.41562000	0.61881600	C	2.79448400	0.02303900	-1.41013100
C	2.70504000	0.73507200	-0.61759200	C	2.49779400	1.22812200	0.72831000
H	2.65662500	2.50144200	0.60650700	H	2.74727000	0.02263200	2.49640000
C	-2.71063200	-1.41562000	0.61881600	C	-2.50765600	-1.23291600	-1.41016000
C	-2.70504000	0.73507200	-0.61759200	C	-2.78329800	-0.02292800	0.72829300
H	-2.65662500	2.50144200	0.60650700	H	-2.46525600	-1.21227600	2.49643900
C	2.70504000	-0.73507200	-0.61759200	C	2.49776500	1.22816700	-0.72828800
H	2.65662500	-2.50144200	0.60650700	H	2.74718700	0.02264400	-2.49641300
C	2.17405000	1.41561900	-1.73416600	C	1.72416700	2.19873400	1.41020200
C	-2.70504000	-0.73507200	-0.61759200	C	-2.78331400	-0.02294300	-0.72827700
H	-2.65662500	-2.50144200	0.60650700	H	-2.46529400	-1.21214600	-2.49645300
C	-2.17405000	1.41561900	-1.73416600	C	-2.52766000	1.19152400	1.41016700
C	2.17405000	-1.41561900	-1.73416600	C	1.72423900	2.19874900	-1.41017300
H	2.13059500	2.50142700	-1.69948100	H	1.69519700	2.16172300	2.49651000
C	1.20409200	0.73508000	-2.50094200	C	0.59688300	2.71783500	0.72832100
H	2.13059500	-2.50142700	-1.69948100	H	1.69525400	2.16180800	-2.49647900
C	1.20409200	-0.73508000	-2.50094200	C	0.59684800	2.71787400	-0.72828000
C	0.00000000	1.41557300	-2.78191200	C	-0.64425800	2.71862700	1.41025500
C	0.00000000	-1.41557300	-2.78191200	C	-0.64418100	2.71874800	-1.41020000
C	-2.17405000	-1.41561900	-1.73416600	C	-2.52765600	1.19168100	-1.41012000
C	-1.20409200	0.73508000	-2.50094200	C	-1.75321500	2.16141700	0.72833000
H	0.00000000	2.50138900	-2.72640000	H	-0.63340900	2.67295700	2.49655400
C	-1.20409200	-0.73508000	-2.50094200	C	-1.75323500	2.16140600	-0.72825800
H	0.00000000	-2.50138900	-2.72640000	H	-0.63342200	2.67311200	-2.49649800
H	-2.13059500	2.50142700	-1.69948100	H	-2.48495700	1.17135400	2.49645300
H	-2.13059500	-2.50142700	-1.69948100	H	-2.48499500	1.17150000	-2.49640800
**Cyc8_R**				**Cyc8_U**			
C	0.00000000	3.16219500	0.72912200	C	0.00000000	3.16570400	0.72877900
C	-1.21536500	2.93415100	1.40594600	C	-1.21592000	2.93549000	1.40788400
C	-2.23600900	2.23600900	0.72912200	C	-2.23849100	2.23849100	0.72877900
C	-2.23600900	2.23600900	-0.72912200	C	-2.23849100	2.23849100	-0.72877900
C	-1.21536500	2.93415100	-1.40594600	C	-1.21592000	2.93549000	-1.40788400
C	0.00000000	3.16219500	-0.72912200	C	0.00000000	3.16570400	-0.72877900
C	-2.93415100	1.21536500	1.40594600	C	-2.93549000	1.21592000	1.40788400
C	-2.93415100	1.21536500	-1.40594600	C	-2.93549000	1.21592000	-1.40788400
C	-3.16219500	0.00000000	-0.72912200	C	-3.16570400	0.00000000	-0.72877900
C	-3.16219500	0.00000000	0.72912200	C	-3.16570400	0.00000000	0.72877900
C	-2.93415100	-1.21536500	1.40594600	C	-2.93549000	-1.21592000	1.40788400
H	-2.90363300	-1.20272400	2.49310400	H	-2.90036900	-1.20137200	2.49479800
C	-2.93415100	-1.21536500	-1.40594600	C	-2.93549000	-1.21592000	-1.40788400
H	-2.90363300	1.20272400	2.49310400	H	-2.90036900	1.20137200	2.49479800
H	-1.20272400	2.90363300	2.49310400	H	-1.20137200	2.90036900	2.49479800
H	-1.20272400	2.90363300	-2.49310400	H	-1.20137200	2.90036900	-2.49479800
H	-2.90363300	1.20272400	-2.49310400	H	-2.90036900	1.20137200	-2.49479800
H	-2.90363300	-1.20272400	-2.49310400	H	-2.90036900	-1.20137200	-2.49479800
C	-2.23600900	-2.23600900	0.72912200	C	-2.23849100	-2.23849100	0.72877900
C	-1.21536500	-2.93415100	1.40594600	C	-1.21592000	-2.93549000	1.40788400
C	0.00000000	-3.16219500	0.72912200	C	0.00000000	-3.16570400	0.72877900
C	0.00000000	-3.16219500	-0.72912200	C	0.00000000	-3.16570400	-0.72877900
C	-1.21536500	-2.93415100	-1.40594600	C	-1.21592000	-2.93549000	-1.40788400
C	-2.23600900	-2.23600900	-0.72912200	C	-2.23849100	-2.23849100	-0.72877900
C	1.21536500	-2.93415100	1.40594600	C	1.21592000	-2.93549000	1.40788400
C	1.21536500	-2.93415100	-1.40594600	C	1.21592000	-2.93549000	-1.40788400
C	2.23600900	-2.23600900	-0.72912200	C	2.23849100	-2.23849100	-0.72877900
C	2.23600900	-2.23600900	0.72912200	C	2.23849100	-2.23849100	0.72877900

C	2.93415100	-1.21536500	1.40594600	C	2.93549000	-1.21592000	1.40788400
H	2.90363300	-1.20272400	2.49310400	H	2.90036900	-1.20137200	2.49479800
C	2.93415100	-1.21536500	-1.40594600	C	2.93549000	-1.21592000	-1.40788400
H	1.20272400	-2.90363300	2.49310400	H	1.20137200	-2.90036900	2.49479800
H	-1.20272400	-2.90363300	2.49310400	H	-1.20137200	-2.90036900	2.49479800
H	-1.20272400	-2.90363300	-2.49310400	H	-1.20137200	-2.90036900	-2.49479800
H	1.20272400	-2.90363300	-2.49310400	H	1.20137200	-2.90036900	-2.49479800
H	2.90363300	-1.20272400	-2.49310400	H	2.90036900	-1.20137200	-2.49479800
C	3.16219500	0.00000000	0.72912200	C	3.16570400	0.00000000	0.72877900
C	3.16219500	0.00000000	-0.72912200	C	3.16570400	0.00000000	-0.72877900
H	2.90363300	1.20272400	2.49310400	H	2.90036900	1.20137200	2.49479800
C	2.93415100	1.21536500	1.40594600	C	2.93549000	1.21592000	1.40788400
C	2.93415100	1.21536500	-1.40594600	C	2.93549000	1.21592000	-1.40788400
H	2.90363300	1.20272400	-2.49310400	H	2.90036900	1.20137200	-2.49479800
C	2.23600900	2.23600900	0.72912200	C	2.23849100	2.23849100	0.72877900
C	1.21536500	2.93415100	1.40594600	C	1.21592000	2.93549000	1.40788400
C	1.21536500	2.93415100	-1.40594600	C	1.21592000	2.93549000	-1.40788400
C	2.23600900	2.23600900	-0.72912200	C	2.23849100	2.23849100	-0.72877900
H	1.20272400	2.90363300	2.49310400	H	1.20137200	2.90036900	2.49479800
H	1.20272400	2.90363300	-2.49310400	H	1.20137200	2.90036900	-2.49479800
**Cyc9_R**				**Cyc9_U**			
C	-3.49356500	-0.73462400	0.61608800	C	-0.61776200	3.50301400	0.72773400
C	-3.07662800	-1.41473700	1.77650400	C	-1.78391200	3.08940900	1.40831100
C	-3.49356500	0.73462400	0.61608800	C	-0.61776200	3.50301400	-0.72773400
C	-3.49845300	-1.41473900	-0.61694700	C	0.61951700	3.51297900	1.40830900
C	-2.28046900	-0.73462900	2.71806100	C	-2.72547400	2.28665300	0.72773800
H	-3.03742800	-2.50115000	1.75387800	H	-1.76614900	3.05863000	2.49533700
C	-3.07662800	1.41473700	1.77650400	C	-1.78391200	3.08940900	-1.40831100
C	-3.49845300	1.41473900	-0.61694700	C	0.61951700	3.51297900	-1.40830900
C	-3.07223700	-0.73462100	-1.77398200	C	1.77881200	3.08055200	0.72773200
H	-3.45390100	-2.50115500	-0.60908900	H	0.61334400	3.47798600	2.49533700
C	-2.28046900	0.73462900	2.71806100	C	-2.72547400	2.28665300	-0.72773800
C	-1.21516600	-1.41472400	3.33899000	C	-3.35297400	1.22023300	1.40830900
H	-3.03742800	2.50115000	1.75387800	H	-1.76614900	3.05863000	-2.49533700
C	-3.07223700	0.73462100	-1.77398200	C	1.77881200	3.08055200	-0.72773200
H	-3.45390100	2.50115500	-0.60908900	H	0.61334400	3.47798600	-2.49533700
C	-2.28366300	-1.41473500	-2.72187900	C	2.73326000	2.29318000	1.40831600
C	-1.21516600	1.41472400	3.33899000	C	-3.35297400	1.22023300	-1.40830900
C	0.00000000	-0.73462200	3.54824700	C	-3.55795500	0.00000000	0.72773400
H	-1.19970800	-2.50114300	3.29654100	H	-3.31971200	1.20812100	2.49534200
C	0.00000000	0.73462200	3.54824700	C	-3.55795500	0.00000000	-0.72773400
H	-1.19970800	2.50114300	3.29654100	H	-3.31971200	1.20812100	-2.49534200
C	1.21516600	-1.41472400	3.33899000	C	-3.35297400	-1.22023300	1.40830900
C	1.21516600	1.41472400	3.33899000	C	-3.35297400	-1.22023300	-1.40830900
C	-2.28366300	1.41473500	-2.72187900	C	2.73326000	2.29318000	-1.40831600
H	1.19970800	-2.50114300	3.29654100	H	-3.31971200	-1.20812100	2.49534200
C	2.28046900	-0.73462900	2.71806100	C	-2.72547400	-2.28665300	0.72773800
H	1.19970800	2.50114300	3.29654100	H	-3.31971200	-1.20812100	-2.49534200
C	2.28046900	0.73462900	2.71806100	C	-2.72547400	-2.28665300	-0.72773800
C	3.07662800	1.41473700	1.77650400	C	-1.78391200	-3.08940900	-1.40831100
C	3.07662800	-1.41473700	1.77650400	C	-1.78391200	-3.08940900	1.40831100
C	3.49356500	-0.73462400	0.61608800	C	-0.61776200	-3.50301400	0.72773400
H	3.03742800	-2.50115000	1.75387800	H	-1.76614900	-3.05863000	2.49533700
C	3.49356500	0.73462400	0.61608800	C	-0.61776200	-3.50301400	-0.72773400
H	3.03742800	2.50115000	1.75387800	H	-1.76614900	-3.05863000	-2.49533700
C	3.49845300	-1.41473900	-0.61694700	C	0.61951700	-3.51297900	1.40830900
C	3.49845300	1.41473900	-0.61694700	C	0.61951700	-3.51297900	-1.40830900
C	3.07223700	-0.73462100	-1.77398200	C	1.77881200	-3.08055200	0.72773200

H	3.45390100	-2.50115500	-0.60908900	H	0.61334400	-3.47798600	2.49533700
C	3.07223700	0.73462100	-1.77398200	C	1.77881200	-3.08055200	-0.72773200
H	3.45390100	2.50115500	-0.60908900	H	0.61334400	-3.47798600	-2.49533700
C	2.28366300	-1.41473500	-2.72187900	C	2.73326000	-2.29318000	1.40831600
C	2.28366300	1.41473500	-2.72187900	C	2.73326000	-2.29318000	-1.40831600
H	2.25464900	-2.50115500	-2.68727300	H	2.70614200	-2.27044200	2.49534900
C	1.21343700	-0.73463300	-3.33429400	C	3.34340900	-1.21673700	0.72774200
H	2.25464900	2.50115500	-2.68727300	H	2.70614200	-2.27044200	-2.49534900
C	1.21343700	0.73463300	-3.33429400	C	3.34340900	-1.21673700	-0.72774200
C	0.00000000	1.41474000	-3.55333700	C	3.56821200	0.00000000	-1.40831900
C	0.00000000	-1.41474000	-3.55333700	C	3.56821200	0.00000000	1.40831900
C	-1.21343700	-0.73463300	-3.33429400	C	3.34340900	1.21673700	0.72774200
H	0.00000000	-2.50115300	-3.50806400	H	3.53270400	0.00000000	2.49534700
C	-1.21343700	0.73463300	-3.33429400	C	3.34340900	1.21673700	-0.72774200
H	0.00000000	2.50115300	-3.50806400	H	3.53270400	0.00000000	-2.49534700
H	-2.25464900	-2.50115500	-2.68727300	H	2.70614200	2.27044200	2.49534900
H	-2.25464900	2.50115500	-2.68727300	H	2.70614200	2.27044200	-2.49534900
**Cyc10_R**				**Cyc10_U**			
C	0.00000000	3.93629300	0.73105600	C	0.00000000	3.94121100	0.72960900
C	0.00000000	3.93629300	-0.73105600	C	0.00000000	3.94121100	-0.72960900
C	-1.21973900	3.75397100	1.40768000	C	-1.22073100	3.75702300	1.40894600
C	-1.21973900	3.75397100	-1.40768000	C	-1.22073100	3.75702300	-1.40894600
H	-1.21090000	3.72676600	2.49489300	H	-1.21099700	3.72706400	2.49603200
H	-1.21090000	3.72676600	-2.49489300	H	-1.21099700	3.72706400	-2.49603200
C	-3.74363700	1.21638100	0.73105600	C	-3.74831400	1.21790100	0.72960900
C	-3.74363700	1.21638100	-0.73105600	C	-3.74831400	1.21790100	-0.72960900
C	-1.21973900	-3.75397100	1.40768000	C	-1.22073100	-3.75702300	1.40894600
C	-2.31369500	-3.18452800	0.73105600	C	-2.31658600	-3.18850700	0.72960900
C	-3.19331800	-2.32008200	1.40768000	C	-3.19591500	-2.32196800	1.40894600
C	-2.31369500	-3.18452800	-0.73105600	C	-2.31658600	-3.18850700	-0.72960900
C	-3.19331800	-2.32008200	-1.40768000	C	-3.19591500	-2.32196800	-1.40894600
C	-3.74363700	-1.21638100	-0.73105600	C	-3.74831400	-1.21790100	-0.72960900
C	-3.74363700	-1.21638100	0.73105600	C	-3.74831400	-1.21790100	0.72960900
C	0.00000000	-3.93629300	0.73105600	C	0.00000000	-3.94121100	0.72960900
C	-1.21973900	-3.75397100	-1.40768000	C	-1.22073100	-3.75702300	-1.40894600
H	-3.17017600	-2.30326800	-2.49489300	H	-3.17043000	-2.30345200	-2.49603200
C	-3.94715900	0.00000000	-1.40768000	C	-3.95036800	0.00000000	-1.40894600
C	-3.94715900	0.00000000	1.40768000	C	-3.95036800	0.00000000	1.40894600
H	-3.91855300	0.00000000	-2.49489300	H	-3.91886700	0.00000000	-2.49603200
H	-3.91855300	0.00000000	2.49489300	H	-3.91886700	0.00000000	2.49603200
C	0.00000000	-3.93629300	-0.73105600	C	0.00000000	-3.94121100	-0.72960900
H	-1.21090000	-3.72676600	2.49489300	H	-1.21099700	-3.72706400	2.49603200
H	-3.17017600	-2.30326800	2.49489300	H	-3.17043000	-2.30345200	2.49603200
H	-1.21090000	-3.72676600	-2.49489300	H	-1.21099700	-3.72706400	-2.49603200
C	-3.19331800	2.32008200	1.40768000	C	-3.19591500	2.32196800	1.40894600
C	-2.31369500	3.18452800	0.73105600	C	-2.31658600	3.18850700	0.72960900
C	-2.31369500	3.18452800	-0.73105600	C	-2.31658600	3.18850700	-0.72960900
C	-3.19331800	2.32008200	-1.40768000	C	-3.19591500	2.32196800	-1.40894600
H	-3.17017600	2.30326800	2.49489300	H	-3.17043000	2.30345200	2.49603200
H	-3.17017600	2.30326800	-2.49489300	H	-3.17043000	2.30345200	-2.49603200
C	1.21973900	-3.75397100	1.40768000	C	1.22073100	-3.75702300	1.40894600
C	1.21973900	-3.75397100	-1.40768000	C	1.22073100	-3.75702300	-1.40894600
H	1.21090000	-3.72676600	2.49489300	H	1.21099700	-3.72706400	2.49603200
H	1.21090000	-3.72676600	-2.49489300	H	1.21099700	-3.72706400	-2.49603200
C	3.74363700	-1.21638100	0.73105600	C	3.74831400	-1.21790100	0.72960900
C	3.74363700	-1.21638100	-0.73105600	C	3.74831400	-1.21790100	-0.72960900
C	1.21973900	3.75397100	1.40768000	C	1.22073100	3.75702300	1.40894600
C	2.31369500	3.18452800	0.73105600	C	2.31658600	3.18850700	0.72960900

C	3.19331800	2.32008200	1.40768000	C	3.19591500	2.32196800	1.40894600
C	2.31369500	3.18452800	-0.73105600	C	2.31658600	3.18850700	-0.72960900
C	3.19331800	2.32008200	-1.40768000	C	3.19591500	2.32196800	-1.40894600
C	3.74363700	1.21638100	-0.73105600	C	3.74831400	1.21790100	-0.72960900
C	3.74363700	1.21638100	0.73105600	C	3.74831400	1.21790100	0.72960900
C	1.21973900	3.75397100	-1.40768000	C	1.22073100	3.75702300	-1.40894600
H	3.17017600	2.30326800	-2.49489300	H	3.17043000	2.30345200	-2.49603200
C	3.94715900	0.00000000	-1.40768000	C	3.95036800	0.00000000	-1.40894600
C	3.94715900	0.00000000	1.40768000	C	3.95036800	0.00000000	1.40894600
H	3.91855300	0.00000000	-2.49489300	H	3.91886700	0.00000000	-2.49603200
H	3.91855300	0.00000000	2.49489300	H	3.91886700	0.00000000	2.49603200
H	1.21090000	3.72676600	2.49489300	H	1.21099700	3.72706400	2.49603200
H	3.17017600	2.30326800	2.49489300	H	3.17043000	2.30345200	2.49603200
H	1.21090000	3.72676600	-2.49489300	H	1.21099700	3.72706400	-2.49603200
C	3.19331800	-2.32008200	1.40768000	C	3.19591500	-2.32196800	1.40894600
C	2.31369500	-3.18452800	0.73105600	C	2.31658600	-3.18850700	0.72960900
C	2.31369500	-3.18452800	-0.73105600	C	2.31658600	-3.18850700	-0.72960900
C	3.19331800	-2.32008200	-1.40768000	C	3.19591500	-2.32196800	-1.40894600
H	3.17017600	-2.30326800	2.49489300	H	3.17043000	-2.30345200	2.49603200
H	3.17017600	-2.30326800	-2.49489300	H	3.17043000	-2.30345200	-2.49603200
**Cyc11_R**				**Cyc11_U**			
C	-4.28030300	0.73412200	-0.61541500	C	-0.80136100	4.26009000	0.72815100
C	-4.28030300	-0.73412200	-0.61541500	C	-0.80136100	4.26009000	-0.72815100
C	-4.28456100	1.41419100	0.61602700	C	-1.97304800	3.86983100	1.40824400
C	-3.93745200	1.41419100	-1.79817400	C	0.43202300	4.32247200	1.40825600
C	-4.28456100	-1.41419100	0.61602700	C	-1.97304800	3.86983100	-1.40824400
C	-3.93745200	-1.41419100	-1.79817400	C	0.43202300	4.32247200	-1.40825600
C	-3.93353800	-0.73412200	1.79638700	C	-2.97668800	3.15020700	-0.72814500
C	-3.27135300	-1.41419100	2.83464400	C	-3.75125200	2.18835400	-1.40826900
C	-2.33790300	-0.73412200	3.63784900	C	-4.20672500	1.04045700	-0.72814700
C	-2.33790300	0.73412200	3.63784900	C	-4.20672500	1.04045700	0.72814700
C	-1.21951300	1.41419100	4.15328000	C	-4.33866900	-0.18743300	1.40825900
C	0.00000000	0.73412200	4.32431800	C	-4.10138500	-1.39938500	0.72814600
H	-1.20899800	2.50083900	4.11746800	H	-4.31054600	-0.18621600	2.49535800
C	-3.27135300	1.41419100	2.83464400	C	-3.75125200	2.18835400	1.40826900
C	-3.93353800	0.73412200	1.79638700	C	-2.97668800	3.15020700	0.72814500
H	-3.24314500	2.50083900	2.81020100	H	-3.72685500	2.17414500	2.49536200
C	-1.21951300	-1.41419100	4.15328000	C	-4.33866900	-0.18743300	-1.40825900
H	-3.24314500	-2.50083900	2.81020100	H	-3.72685500	2.17414500	-2.49536200
C	0.00000000	-0.73412200	4.32431800	C	-4.10138500	-1.39938500	-0.72814600
C	1.21951300	1.41419100	4.15328000	C	-3.54870600	-2.50376500	1.40825800
H	-1.20899800	-2.50083900	4.11746800	H	-4.31054600	-0.18621600	-2.49535800
H	-4.24761700	-2.50083900	0.61071500	H	-1.96021100	3.84470400	-2.49534300
H	-4.24761700	2.50083900	0.61071500	H	-1.96021100	3.84470400	2.49534300
C	1.21951300	-1.41419100	4.15328000	C	-3.54870600	-2.50376500	-1.40825800
C	2.33790300	0.73412200	3.63784900	C	-2.69403900	-3.39522900	0.72814700
C	3.27135300	1.41419100	2.83464400	C	-1.63205900	-4.02556900	1.40824900
C	3.93353800	0.73412200	1.79638700	C	-0.43108300	-4.31331900	0.72815100
C	4.28456100	1.41419100	0.61602700	C	0.80308900	-4.26915800	1.40825800
C	4.28030300	0.73412200	-0.61541500	C	1.96888900	-3.86167000	0.72815800
C	3.93745200	1.41419100	-1.79817400	C	2.98305600	-3.15693200	1.40826200
C	3.26810200	0.73412200	-2.83182600	C	3.74325300	-2.18371500	0.72813900
C	3.26810200	-0.73412200	-2.83182600	C	3.74325300	-2.18371500	-0.72813900
C	2.34022900	1.41419100	-3.64146700	C	4.21562400	-1.04266600	1.40826000
C	3.93745200	-1.41419100	-1.79817400	C	2.98305600	-3.15693200	-1.40826200
C	2.34022900	-1.41419100	-3.64146700	C	4.21562400	-1.04266600	-1.40826000
H	3.90350100	2.50083900	-1.78266900	H	2.96366500	-3.13638600	2.49535900
C	4.28030300	-0.73412200	-0.61541500	C	1.96888900	-3.86167000	-0.72815800

H	4.24761700	2.50083900	0.61071500	H	0.79785000	-4.24132300	2.49535100
C	4.28456100	-1.41419100	0.61602700	C	0.80308900	-4.26915800	-1.40825800
C	3.93353800	-0.73412200	1.79638700	C	-0.43108300	-4.31331900	-0.72815100
H	4.24761700	-2.50083900	0.61071500	H	0.79785000	-4.24132300	-2.49535100
C	3.27135300	-1.41419100	2.83464400	C	-1.63205900	-4.02556900	-1.40824900
H	3.90350100	-2.50083900	-1.78266900	H	2.96366500	-3.13638600	-2.49535900
C	2.33790300	-0.73412200	3.63784900	C	-2.69403900	-3.39522900	-0.72814700
H	3.24314500	-2.50083900	2.81020100	H	-1.62145300	-3.99942000	-2.49534800
H	3.24314500	2.50083900	2.81020100	H	-1.62145300	-3.99942000	2.49534800
H	1.20899800	-2.50083900	4.11746800	H	-3.52565900	-2.48752700	-2.49535300
H	1.20899800	2.50083900	4.11746800	H	-3.52565900	-2.48752700	2.49535300
C	1.21830100	-0.73412200	-4.14915300	C	4.32934200	0.18704200	-0.72814600
H	2.32005000	-2.50083900	-3.61006800	H	4.18831200	-1.03591500	-2.49535700
C	0.00000000	-1.41419100	-4.32862000	C	4.11011200	1.40238300	-1.40826200
C	1.21830100	0.73412200	-4.14915300	C	4.32934200	0.18704200	0.72814600
C	-1.21830100	-0.73412200	-4.14915300	C	3.54117200	2.49848000	-0.72814500
H	0.00000000	-2.50083900	-4.29129600	H	4.08343500	1.39329100	-2.49535800
C	0.00000000	1.41419100	-4.32862000	C	4.11011200	1.40238300	1.40826200
C	-1.21830100	0.73412200	-4.14915300	C	3.54117200	2.49848000	0.72814500
H	0.00000000	2.50083900	-4.29129600	H	4.08343500	1.39329100	2.49535800
C	-2.34022900	1.41419100	-3.64146700	C	2.69982900	3.40249400	1.40826200
C	-2.34022900	-1.41419100	-3.64146700	C	2.69982900	3.40249400	-1.40826200
H	2.32005000	2.50083900	-3.61006800	H	4.18831200	-1.03591500	2.49535700
C	-3.26810200	0.73412200	-2.83182600	C	1.62861700	4.01702500	0.72815400
H	-2.32005000	2.50083900	-3.61006800	H	2.68229900	3.38039200	2.49536000
C	-3.26810200	-0.73412200	-2.83182600	C	1.62861700	4.01702500	-0.72815400
H	-2.32005000	-2.50083900	-3.61006800	H	2.68229900	3.38039200	-2.49536000
H	-3.90350100	2.50083900	-1.78266900	H	0.42920400	4.29428700	2.49535000
H	-3.90350100	-2.50083900	-1.78266900	H	0.42920400	4.29428700	-2.49535000
**Cyc12_R**				**Cyc12_U**			
C	2.552928000	3.961860000	0.732078000	C	0.00000000	4.72066800	0.72971900
C	2.552928000	3.961860000	-0.732078000	C	0.00000000	4.72066800	-0.72971900
C	3.497728000	3.171906000	1.408780000	C	1.22389400	4.56763400	1.40890900
C	1.443168000	4.495815000	1.408780000	C	-1.22389400	4.56763400	1.40890900
C	3.497728000	3.171906000	-1.408780000	C	1.22389400	4.56763400	-1.40890900
C	1.443168000	4.495815000	-1.408780000	C	-1.22389400	4.56763400	-1.40890900
C	4.191830000	2.154607000	0.732078000	C	2.36033400	4.08821900	0.72971900
H	3.478922000	3.154852000	2.496052000	H	1.21704100	4.54205800	2.49617200
C	4.615075000	0.998087000	1.408780000	C	3.34374000	3.34374000	1.40890900
C	4.191830000	2.154607000	-0.732078000	C	2.36033400	4.08821900	-0.72971900
C	4.707535000	-0.229970000	0.732078000	C	4.08821900	2.36033400	0.72971900
H	4.590260000	0.992721000	2.496052000	H	3.32501700	3.32501700	2.49617200
C	4.615075000	0.998087000	-1.408780000	C	3.34374000	3.34374000	-1.40890900
C	4.707535000	-0.229970000	-0.732078000	C	4.08821900	2.36033400	-0.72971900
H	4.590260000	0.992721000	-2.496052000	H	3.32501700	3.32501700	-2.49617200
C	4.495815000	-1.443168000	-1.408780000	C	4.56763400	1.22389400	-1.40890900
C	4.495815000	-1.443168000	1.408780000	C	4.56763400	1.22389400	1.40890900
H	3.478922000	3.154852000	-2.496052000	H	1.21704100	4.54205800	-2.49617200
C	3.961860000	-2.552928000	-0.732078000	C	4.72066800	0.00000000	-0.72971900
H	4.471642000	-1.435409000	-2.496052000	H	4.54205800	1.21704100	-2.49617200
C	3.961860000	-2.552928000	0.732078000	C	4.72066800	0.00000000	0.72971900
H	4.471642000	-1.435409000	2.496052000	H	4.54205800	1.21704100	2.49617200
C	3.171906000	-3.497728000	1.408780000	C	4.56763400	-1.22389400	1.40890900
C	2.154607000	-4.191830000	0.732078000	C	4.08821900	-2.36033400	0.72971900
C	0.998087000	-4.615075000	1.408780000	C	3.34374000	-3.34374000	1.40890900
C	-0.229970000	-4.707535000	0.732078000	C	2.36033400	-4.08821900	0.72971900
H	0.992721000	-4.590260000	2.496052000	H	3.32501700	-3.32501700	2.49617200
C	-1.443168000	-4.495815000	1.408780000	C	1.22389400	-4.56763400	1.40890900

C	-0.229970000	-4.707535000	-0.732078000	C	2.36033400	-4.08821900	-0.72971900
C	-2.552928000	-3.961860000	0.732078000	C	0.00000000	-4.72066800	0.72971900
H	-1.435409000	-4.471642000	2.496052000	H	1.21704100	-4.54205800	2.49617200
C	-1.443168000	-4.495815000	-1.408780000	C	1.22389400	-4.56763400	-1.40890900
C	0.998087000	-4.615075000	-1.408780000	C	3.34374000	-3.34374000	-1.40890900
C	-2.552928000	-3.961860000	-0.732078000	C	0.00000000	-4.72066800	-0.72971900
H	-1.435409000	-4.471642000	-2.496052000	H	1.21704100	-4.54205800	-2.49617200
C	-3.497728000	-3.171906000	-1.408780000	C	-1.22389400	-4.56763400	-1.40890900
C	-3.497728000	-3.171906000	1.408780000	C	-1.22389400	-4.56763400	1.40890900
C	2.154607000	-4.191830000	-0.732078000	C	4.08821900	-2.36033400	-0.72971900
H	0.992721000	-4.590260000	-2.496052000	H	3.32501700	-3.32501700	-2.49617200
H	-3.478922000	-3.154852000	-2.496052000	H	-1.21704100	-4.54205800	-2.49617200
C	-4.191830000	-2.154607000	-0.732078000	C	-2.36033400	-4.08821900	-0.72971900
H	-3.478922000	-3.154852000	2.496052000	H	-1.21704100	-4.54205800	2.49617200
C	-4.191830000	-2.154607000	0.732078000	C	-2.36033400	-4.08821900	0.72971900
C	3.171906000	-3.497728000	-1.408780000	C	4.56763400	-1.22389400	-1.40890900
C	-4.615075000	-0.998087000	1.408780000	C	-3.34374000	-3.34374000	1.40890900
C	-4.615075000	-0.998087000	-1.408780000	C	-3.34374000	-3.34374000	-1.40890900
H	3.154852000	-3.478922000	-2.496052000	H	4.54205800	-1.21704100	-2.49617200
H	3.154852000	-3.478922000	2.496052000	H	4.54205800	-1.21704100	2.49617200
C	0.229970000	4.707535000	-0.732078000	C	-2.36033400	4.08821900	-0.72971900
C	-0.998087000	4.615075000	-1.408780000	C	-3.34374000	3.34374000	-1.40890900
C	-2.154607000	4.191830000	-0.732078000	C	-4.08821900	2.36033400	-0.72971900
C	-2.154607000	4.191830000	0.732078000	C	-4.08821900	2.36033400	0.72971900
C	-3.171906000	3.497728000	1.408780000	C	-4.56763400	1.22389400	1.40890900
C	-3.961860000	2.552928000	0.732078000	C	-4.72066800	0.00000000	0.72971900
H	-3.154852000	3.478922000	2.496052000	H	-4.54205800	1.21704100	2.49617200
C	-0.998087000	4.615075000	1.408780000	C	-3.34374000	3.34374000	1.40890900
C	0.229970000	4.707535000	0.732078000	C	-2.36033400	4.08821900	0.72971900
H	-0.992721000	4.590260000	2.496052000	H	-3.32501700	3.32501700	2.49617200
C	-3.171906000	3.497728000	-1.408780000	C	-4.56763400	1.22389400	-1.40890900
H	-0.992721000	4.590260000	-2.496052000	H	-3.32501700	3.32501700	-2.49617200
C	-3.961860000	2.552928000	-0.732078000	C	-4.72066800	0.00000000	-0.72971900
C	-4.495815000	1.443168000	1.408780000	C	-4.56763400	-1.22389400	1.40890900
H	-3.154852000	3.478922000	-2.496052000	H	-4.54205800	1.21704100	-2.49617200
H	1.435409000	4.471642000	-2.496052000	H	-1.21704100	4.54205800	-2.49617200
H	1.435409000	4.471642000	2.496052000	H	-1.21704100	4.54205800	2.49617200
C	-4.495815000	1.443168000	-1.408780000	C	-4.56763400	-1.22389400	-1.40890900
C	-4.707535000	0.229970000	-0.732078000	C	-4.08821900	-2.36033400	-0.72971900
H	-4.590260000	-0.992721000	-2.496052000	H	-3.32501700	-3.32501700	-2.49617200
C	-4.707535000	0.229970000	0.732078000	C	-4.08821900	-2.36033400	0.72971900
H	-4.590260000	-0.992721000	2.496052000	H	-3.32501700	-3.32501700	2.49617200
H	-4.471642000	1.435409000	-2.496052000	H	-4.54205800	-1.21704100	-2.49617200
H	-4.471642000	1.435409000	2.496052000	H	-4.54205800	-1.21704100	2.49617200
**Cyc13_R**				**Cyc13_U**			
C	-0.62578200	-5.06873600	-0.73136200	C	-4.30695500	2.75561700	0.72865900
C	-0.62610300	-5.06870400	0.73158200	C	-4.30694200	2.75564800	-0.72880300
C	-1.84771000	-4.77065900	-1.40868800	C	-4.84933400	1.64769600	1.40809200
C	0.58174300	-5.08229800	-1.40869600	C	-3.52762400	3.71180400	1.40811400
C	-1.80071000	-4.78847100	1.40889900	C	-4.84929800	1.64772300	-1.40825500
C	0.63166500	-5.07623900	1.40887400	C	-3.52762600	3.71185400	-1.40824000
C	-2.91084600	-4.19798200	-0.73137800	C	-5.09486300	0.43882700	0.72864500
H	-1.83825600	-4.74832100	-2.49592900	H	-4.82684500	1.64002200	2.49532900
C	1.80240500	-4.77905300	-0.73139600	C	-2.53253200	4.44080400	0.72867400
H	0.57970800	-5.05807700	-2.49593700	H	-3.51130600	3.69458100	2.49534900
C	-2.91111100	-4.19763300	0.73157200	C	-5.09482700	0.43885200	-0.72882500
H	-1.79289600	-4.76544200	2.49614200	H	-4.82677600	1.64008700	-2.49549100
C	1.80208000	-4.77899500	0.73153200	C	-2.53252200	4.44083400	-0.72879100

H	0.62792800	-5.05215300	2.49611200	H	-3.51129700	3.69468000	-2.49547500
C	-3.85446800	-3.36639100	-1.40868500	C	-5.05990400	-0.79422900	1.40808300
C	2.87817100	-4.23042500	-1.40874400	C	-1.39816000	4.92541200	1.40812600
C	-3.82123400	-3.40403100	1.40886800	C	-5.05980100	-0.79420400	-1.40826800
C	2.91957500	-4.20183100	1.40887200	C	-1.39815300	4.92545000	-1.40824000
C	-4.52984400	-2.36527600	-0.73139300	C	-4.71507900	-1.97861600	0.72864400
H	-3.83559700	-3.35097200	-2.49593100	H	-5.03647300	-0.79056900	2.49531900
C	3.81834700	-3.39485800	-0.73143100	C	-0.17831200	5.10874300	0.72866700
H	2.86507800	-4.20992300	-2.49597900	H	-1.39170000	4.90263700	2.49536100
C	-4.53009200	-2.36503700	0.73154800	C	-4.71502600	-1.97858800	-0.72881300
H	-3.80368100	-3.38724100	2.49611200	H	-5.03628800	-0.79050500	-2.49550200
C	3.81819000	-3.39507400	0.73150100	C	-0.17829200	5.10874300	-0.72878300
H	2.90515000	-4.18221200	2.49610700	H	-1.39167500	4.90271400	-2.49547600
C	-4.97877600	-1.19034500	-1.40875600	C	-4.11083100	-3.05402900	1.40811000
C	4.51600700	-2.40920100	-1.40875600	C	1.05136900	5.01109200	1.40813100
C	-4.96685100	-1.23912000	1.40886700	C	-4.11071500	-3.05399700	-1.40824300
C	4.53945200	-2.36466900	1.40880000	C	1.05138000	5.01105000	-1.40824600
C	-5.11100800	0.01000400	-0.73146100	C	-3.25496600	-3.94238000	0.72868800
H	-4.95492200	-1.18540300	-2.49599100	H	-4.09185600	-3.03991200	2.49534600
C	-5.11100500	0.01032600	0.73146900	C	-3.25492400	-3.94236100	-0.72876200
H	-4.94338900	-1.23249800	2.49610400	H	-4.09166300	-3.03983800	-2.49547700
C	-4.97399200	1.21013900	1.40876200	C	-2.22011600	-4.61382600	-1.40818400
C	-4.96188300	1.25886600	-1.40886100	C	-2.22020200	-4.61382300	1.40818500
C	-4.52038900	2.38327400	0.73139800	C	-1.04957500	-5.00293400	-0.72868500
H	-4.95015300	1.20510300	2.49599700	H	-2.20986300	-4.59254600	-2.49541900
C	-4.52064100	2.38303500	-0.73154400	C	-1.04960200	-5.00291700	0.72876900
H	-4.93845100	1.25214900	-2.49609800	H	-2.20999800	-4.59254900	2.49542000
C	-3.80765400	3.41919700	-1.40886700	C	0.17864400	-5.11685900	1.40826900
C	-2.89437700	4.20917100	-0.73157500	C	1.39603500	-4.91773800	0.72884500
H	-3.79017100	3.40233400	-2.49611100	H	0.17779700	-5.09321600	2.49550600
C	-3.84103000	3.38169300	1.40868600	C	0.17871700	-5.11693700	-1.40810500
C	-2.89410900	4.20952200	0.73137600	C	1.39606400	-4.91779400	-0.72861500
C	-1.78163500	4.79558400	-1.40890400	C	2.53663000	-4.44803500	1.40832800
H	-3.82221700	3.36620000	2.49593200	H	0.17790900	-5.09335600	-2.49534200
C	-1.82870300	4.77796900	1.40868300	C	2.53671900	-4.44815700	-1.40805300
H	-1.77391300	4.77252000	-2.49614700	H	2.52486500	-4.42739900	2.49556300
C	-0.60592200	5.07114600	-0.73158900	C	3.52213500	-3.70610800	0.72888200
H	-1.81933700	4.75559700	2.49592400	H	2.52500900	-4.42762300	-2.49529100
C	-0.60560100	5.07118300	0.73135500	C	3.52217200	-3.70617700	-0.72859300
C	0.65186600	5.07368000	-1.40888100	C	4.31376400	-2.76007400	1.40831600
C	0.60196900	5.07995000	1.40868800	C	4.31384300	-2.76018400	-1.40804200
C	1.82109100	4.77179000	-0.73153800	C	4.84143500	-1.64509200	0.72884700
H	0.64803300	5.04960500	-2.49611900	H	4.29372100	-2.74720900	2.49555100
C	1.82141500	4.77185300	0.73139000	C	4.84145900	-1.64515600	-0.72861600
H	0.59983800	5.05574300	2.49593000	H	4.29385100	-2.74741700	-2.49527800
C	2.89499100	4.21895500	1.40874100	C	5.10266700	-0.43959500	-1.40809000
C	2.93628400	4.19018800	-1.40887400	C	5.10264300	-0.43950100	1.40825900
C	3.83183600	3.37965100	0.73143100	C	5.05154500	0.79290900	-0.72868300
H	2.88181600	4.19850900	2.49597600	H	5.07901400	-0.43760200	-2.49532400
C	3.83168300	3.37986500	-0.73150000	C	5.05154200	0.79297200	0.72878200
H	2.92178200	4.17062200	-2.49611000	H	5.07897600	-0.43741800	2.49549300
C	4.54884200	2.34659800	-1.40879600	C	4.72243600	1.98183000	1.40818900
C	4.52556500	2.39122500	1.40876000	C	4.72241700	1.98172700	-1.40816000
C	4.95991400	-1.23267800	0.73147700	C	2.21678800	4.60672800	-0.72878300
H	4.51748300	-2.35413500	2.49604000	H	1.04654000	4.98788500	-2.49548300
C	4.96005900	-1.23238200	-0.73146500	C	2.21677600	4.60677300	0.72868000
H	4.49494300	-2.39699100	-2.49600000	H	1.04650200	4.98796400	2.49536800
C	5.11934700	0.01498300	1.40880700	C	3.26025600	3.94882900	-1.40822300
C	5.11925300	-0.03534400	-1.40880000	C	3.26027000	3.94892800	1.40814500
C	4.96492700	1.21264600	0.73147200	C	4.10424200	3.04921300	-0.72874200

H	5.09505100	0.01416900	2.49604800	H	3.24519500	3.93052200	-2.49545800
C	4.96479000	1.21294200	-0.73147100	C	4.10424400	3.04927800	0.72871000
H	5.09496600	-0.03443400	-2.49604100	H	3.24520300	3.93071100	2.49538100
H	4.52683400	2.33615000	-2.49603600	H	4.70056600	1.97270600	2.49542500
H	4.50444800	2.37910100	2.49600400	H	4.70054100	1.97250400	-2.49539500
**Cyc14_R**				**Cyc14_U**			
C	-0.578730000	5.461208000	0.732681000	C	0.00000000	5.50170700	0.72956800
C	-0.578730000	5.461208000	-0.732681000	C	0.00000000	5.50170700	-0.72956800
C	0.651851000	5.460088000	1.409445000	C	1.22588100	5.37093500	1.40857500
C	-1.781746000	5.202197000	1.409445000	C	-1.22588100	5.37093500	1.40857500
C	0.651851000	5.460088000	-1.409445000	C	1.22588100	5.37093500	-1.40857500
C	-1.781746000	5.202197000	-1.409445000	C	-1.22588100	5.37093500	-1.40857500
C	1.848112000	5.171481000	0.732681000	C	2.38710100	4.95686700	0.72956800
H	0.649145000	5.437422000	2.496766000	H	1.22087900	5.34902200	2.49596500
C	2.956341000	4.636542000	1.409445000	C	3.43484200	4.30715500	1.40857500
C	1.848112000	5.171481000	-0.732681000	C	2.38710100	4.95686700	-0.72956800
C	3.908912000	3.857477000	0.732681000	C	4.30140800	3.43025800	0.72956800
H	2.944069000	4.617295000	2.496766000	H	3.42082800	4.28958200	2.49596500
C	2.956341000	4.636542000	-1.409445000	C	3.43484200	4.30715500	-1.40857500
C	3.908912000	3.857477000	-0.732681000	C	4.30140800	3.43025800	-0.72956800
H	2.944069000	4.617295000	-2.496766000	H	3.42082800	4.28958200	-2.49596500
C	4.675291000	2.894671000	-1.409445000	C	4.96349000	2.39029100	-1.40857500
C	4.675291000	2.894671000	1.409445000	C	4.96349000	2.39029100	1.40857500
H	0.649145000	5.437422000	-2.496766000	H	1.22087900	5.34902200	-2.49596500
C	5.195505000	1.779454000	-0.732681000	C	5.36376800	1.22424500	-0.72956800
H	4.655884000	2.882655000	-2.496766000	H	4.94323900	2.38053900	-2.49596500
C	5.195505000	1.779454000	0.732681000	C	5.36376800	1.22424500	0.72956800
H	4.655884000	2.882655000	2.496766000	H	4.94323900	2.38053900	2.49596500
C	5.468243000	0.579476000	1.409445000	C	5.50905800	0.00000000	1.40857500
C	5.453064000	-0.651013000	0.732681000	C	5.36376800	-1.22424500	0.72956800
C	5.178142000	-1.850492000	1.409445000	C	4.96349000	-2.39029100	1.40857500
C	4.630577000	-2.952538000	0.732681000	C	4.30140800	-3.43025800	0.72956800
H	5.156646000	-1.842810000	2.496766000	H	4.94323900	-2.38053900	2.49596500
C	3.862446000	-3.913947000	1.409445000	C	3.43484200	-4.30715500	1.40857500
C	4.630577000	-2.952538000	-0.732681000	C	4.30140800	-3.43025800	-0.72956800
C	2.890948000	-4.669277000	0.732681000	C	2.38710100	-4.95686700	0.72956800
H	3.846412000	-3.897700000	2.496766000	H	3.42082800	-4.28958200	2.49596500
C	3.862446000	-3.913947000	-1.409445000	C	3.43484200	-4.30715500	-1.40857500
C	5.178142000	-1.850492000	-1.409445000	C	4.96349000	-2.39029100	-1.40857500
C	2.890948000	-4.669277000	-0.732681000	C	2.38710100	-4.95686700	-0.72956800
H	3.846412000	-3.897700000	-2.496766000	H	3.42082800	-4.28958200	-2.49596500
C	1.781746000	-5.202197000	-1.409445000	C	1.22588100	-5.37093500	-1.40857500
C	1.781746000	-5.202197000	1.409445000	C	1.22588100	-5.37093500	1.40857500
C	5.453064000	-0.651013000	-0.732681000	C	5.36376800	-1.22424500	-0.72956800
H	5.156646000	-1.842810000	-2.496766000	H	4.94323900	-2.38053900	-2.49596500
H	1.774349000	-5.180602000	-2.496766000	H	1.22087900	-5.34902200	-2.49596500
C	0.578730000	-5.461208000	-0.732681000	C	0.00000000	-5.50170700	-0.72956800
H	1.774349000	-5.180602000	2.496766000	H	1.22087900	-5.34902200	2.49596500
C	0.578730000	-5.461208000	0.732681000	C	0.00000000	-5.50170700	0.72956800
C	5.468243000	0.579476000	-1.409445000	C	5.50905800	0.00000000	-1.40857500
C	-0.651851000	-5.460088000	1.409445000	C	-1.22588100	-5.37093500	1.40857500
C	-0.651851000	-5.460088000	-1.409445000	C	-1.22588100	-5.37093500	-1.40857500
H	5.445543000	0.577070000	-2.496766000	H	5.48658200	0.00000000	-2.49596500
H	5.445543000	0.577070000	2.496766000	H	5.48658200	0.00000000	2.49596500
C	-2.890948000	4.669277000	-0.732681000	C	-2.38710100	4.95686700	-0.72956800
C	-3.862446000	3.913947000	-1.409445000	C	-3.43484200	4.30715500	-1.40857500
C	-4.630577000	2.952538000	-0.732681000	C	-4.30140800	3.43025800	-0.72956800
C	-4.630577000	2.952538000	0.732681000	C	-4.30140800	3.43025800	0.72956800

C	-5.178142000	1.850492000	1.409445000	C	-4.96349000	2.39029100	1.40857500
C	-5.453064000	0.651013000	0.732681000	C	-5.36376800	1.22424500	0.72956800
H	-5.156646000	1.842810000	2.496766000	H	-4.94323900	2.38053900	2.49596500
C	-3.862446000	3.913947000	1.409445000	C	-3.43484200	4.30715500	1.40857500
C	-2.890948000	4.669277000	0.732681000	C	-2.38710100	4.95686700	0.72956800
H	-3.846412000	3.897700000	2.496766000	H	-3.42082800	4.28958200	2.49596500
C	-5.178142000	1.850492000	-1.409445000	C	-4.96349000	2.39029100	-1.40857500
H	-3.846412000	3.897700000	-2.496766000	H	-3.42082800	4.28958200	-2.49596500
C	-5.453064000	0.651013000	-0.732681000	C	-5.36376800	1.22424500	-0.72956800
C	-5.468243000	-0.579476000	1.409445000	C	-5.50905800	0.00000000	1.40857500
H	-5.156646000	1.842810000	-2.496766000	H	-4.94323900	2.38053900	-2.49596500
H	-1.774349000	5.180602000	-2.496766000	H	-1.22087900	5.34902200	-2.49596500
H	-1.774349000	5.180602000	2.496766000	H	-1.22087900	5.34902200	2.49596500
C	-5.468243000	-0.579476000	-1.409445000	C	-5.50905800	0.00000000	-1.40857500
C	-1.848112000	-5.171481000	0.732681000	C	-2.38710100	-4.95686700	0.72956800
H	-0.649145000	-5.437422000	2.496766000	H	-1.22087900	-5.34902200	2.49596500
C	-2.956341000	-4.636542000	1.409445000	C	-3.43484200	-4.30715500	1.40857500
C	-1.848112000	-5.171481000	-0.732681000	C	-2.38710100	-4.95686700	-0.72956800
C	-3.908912000	-3.857477000	0.732681000	C	-4.30140800	-3.43025800	0.72956800
H	-2.944069000	-4.617295000	2.496766000	H	-3.42082800	-4.28958200	2.49596500
C	-2.956341000	-4.636542000	-1.409445000	C	-3.43484200	-4.30715500	-1.40857500
C	-3.908912000	-3.857477000	-0.732681000	C	-4.30140800	-3.43025800	-0.72956800
H	-2.944069000	-4.617295000	-2.496766000	H	-3.42082800	-4.28958200	-2.49596500
C	-4.675291000	-2.894671000	-1.409445000	C	-4.96349000	-2.39029100	-1.40857500
C	-4.675291000	-2.894671000	1.409445000	C	-4.96349000	-2.39029100	1.40857500
H	-0.649145000	-5.437422000	-2.496766000	H	-1.22087900	-5.34902200	-2.49596500
C	-5.195505000	-1.779454000	-0.732681000	C	-5.36376800	-1.22424500	-0.72956800
H	-4.655884000	-2.882655000	-2.496766000	H	-4.94323900	-2.38053900	-2.49596500
C	-5.195505000	-1.779454000	0.732681000	C	-5.36376800	-1.22424500	0.72956800
H	-4.655884000	-2.882655000	2.496766000	H	-4.94323900	-2.38053900	2.49596500
H	-5.445543000	-0.577070000	-2.496766000	H	-5.48658200	0.00000000	-2.49596500
H	-5.445543000	-0.577070000	2.496766000	H	-5.48658200	0.00000000	2.49596500
**Cyc15_R**				**Cyc15_U**			
C	5.57751600	1.89596900	-0.73073300	C	-5.65296200	-1.66740300	0.72921900
C	5.57775400	1.89528500	0.73066800	C	-5.65297800	-1.66739900	-0.72910800
C	5.86116300	0.67597500	-1.40710500	C	-5.18886100	-2.80940100	1.40823100
C	5.07885700	3.00023800	-1.40710500	C	-5.88347400	-0.45646700	1.40825500
C	5.85637800	0.71611100	1.40704300	C	-5.18887900	-2.80937900	-1.40814000
C	5.05817400	3.03497700	1.40704300	C	-5.88352400	-0.45645500	-1.40812800
C	5.86709600	-0.53565600	-0.73073500	C	-4.48558500	-3.82177400	0.72919800
H	5.84251000	0.67424900	-2.49460900	H	-5.17077500	-2.79960400	2.49555800
C	4.32323200	3.99916000	-0.73073200	C	-5.84272600	0.77555600	0.72923700
H	5.06257200	2.99109900	-2.49461200	H	-5.86293500	-0.45487600	2.49558200
C	5.86701400	-0.53638000	0.73067600	C	-4.48558500	-3.82176200	-0.72912900
H	5.83782800	0.71342200	2.49454900	H	-5.17080500	-2.79957400	-2.49546700
C	4.32372900	3.99864200	0.73068000	C	-5.84276000	0.77555800	-0.72910500
H	5.04240000	3.02497900	2.49454900	H	-5.86302400	-0.45486500	-2.49545600
C	5.62947100	-1.76543600	-1.40711900	C	-3.59704100	-4.67619100	1.40821200
C	3.41837900	4.80494800	-1.40710400	C	-5.56045400	1.97549700	1.40825800
C	5.64140100	-1.72683100	1.40704300	C	-3.59703700	-4.67615100	-1.40816200
C	3.38536300	4.82830100	1.40703900	C	-5.56052700	1.97551200	-1.40813200
C	5.14153400	-2.87449000	-0.73076600	C	-2.54280000	-5.31499700	0.72918600
H	5.61177400	-1.75940000	-2.49462800	H	-3.58451300	-4.65988000	2.49553900
C	2.32181100	5.41021300	-0.73073700	C	-5.02180600	3.08426400	0.72922000
H	3.40716600	4.78989100	-2.49460700	H	-5.54107200	1.96861800	2.49558600
C	5.14114000	-2.87511500	0.73065800	C	-2.54279500	-5.31497900	-0.72914900
H	5.62338000	-1.72175600	2.49454800	H	-3.58449800	-4.65981200	-2.49548800
C	2.32249300	5.40995900	0.73066800	C	-5.02184600	3.08426800	-0.72911300

H	3.37499600	4.81273100	2.49454500	H	-5.54120500	1.96863400	-2.49546100
C	4.45034500	-3.87056800	1.40701200	C	-1.38359400	-5.73421700	-1.40817600
C	4.42374200	-3.90096500	-1.40714400	C	-1.38359400	-5.73425000	1.40820600
C	3.52607400	-4.71582800	0.73062700	C	-0.16069900	-5.88923000	-0.72914600
H	4.43596600	-3.85861000	2.49451900	H	-1.37877600	-5.71426100	-2.49550300
C	3.52666800	-4.71540400	-0.73077300	C	-0.16070200	-5.88924100	0.72917400
H	4.41006600	-3.88830000	-2.49465000	H	-1.37878400	-5.71431800	2.49553400
C	2.49023100	-5.34438800	1.40700800	C	1.06882500	-5.80106900	-1.40817700
C	2.45351900	-5.36130400	-1.40713100	C	1.06882500	-5.80107400	1.40820800
C	1.30288000	-5.74089800	-0.73075600	C	2.24913500	-5.44558400	0.72917700
H	2.44612400	-5.34414100	-2.49463700	H	1.06511200	-5.78092800	2.49553400
C	1.30217600	-5.74105700	0.73066400	C	2.24914200	-5.44558900	-0.72914400
H	2.48199900	-5.32760300	2.49451300	H	1.06511900	-5.78091700	-2.49550400
C	0.05986600	-5.89496000	-1.40710200	C	3.33664100	-4.86521700	1.40821400
C	0.10029700	-5.89444300	1.40705700	C	3.33665400	-4.86524700	-1.40818000
C	-1.14573300	-5.77424500	-0.73070500	C	4.27047200	-4.06055600	0.72918300
H	0.06007100	-5.87632100	-2.49461100	H	3.32503500	-4.84828900	2.49554100
C	-1.14643800	-5.77413600	0.73071400	C	4.27048700	-4.06057400	-0.72915600
H	0.09958600	-5.87583800	2.49456600	H	3.32506700	-4.84833600	-2.49550800
C	-2.34411600	-5.40994100	-1.40703800	C	5.02790700	-3.08801500	1.40820100
C	-3.39663400	-4.80973800	-0.73064100	C	5.55358400	-1.97304500	0.72915700
H	-2.33635000	-5.39292400	-2.49454300	H	5.01042400	-3.07724700	2.49552900
C	-2.30694700	-5.42590400	1.40710100	C	5.02793800	-3.08806200	-1.40818600
C	-4.34352500	-3.98988200	-1.40700700	C	5.84981000	-0.77646700	1.40817100
C	-3.39722800	-4.80934800	0.73075800	C	5.55359900	-1.97306400	-0.72916500
H	-2.30001600	-5.40856800	2.49460600	H	5.01048700	-3.07732300	-2.49551500
C	-5.06109900	-3.01357100	-0.73063500	C	5.87631400	0.45593000	0.72913100
H	-4.32949500	-3.97753100	-2.49451500	H	5.82941800	-0.77375000	2.49549800
C	-4.31607100	-4.01957000	1.40714900	C	5.84983100	-0.77651000	-1.40820700
C	-5.59239800	-1.87930800	-1.40700600	C	5.65997300	1.66950100	1.40814000
C	-5.06148600	-3.01296300	0.73079000	C	5.87631700	0.45591200	-0.72920100
H	-4.30273300	-4.00655200	2.49465500	H	5.82945700	-0.77381700	-2.49553500
C	-5.85027900	-0.69543300	-0.73063200	C	5.18263700	2.80600700	0.72910500
H	-5.57454400	-1.87374300	-2.49451100	H	5.64024600	1.66368500	2.49546900
C	-5.57936600	-1.91757900	1.40715600	C	5.65996400	1.66945400	-1.40824500
C	-5.87371600	0.55688200	-1.40699900	C	4.49113100	3.82649600	1.40811200
C	-5.85034700	-0.69471200	0.73077800	C	5.18262100	2.80598400	-0.72924100
H	-5.56182200	-1.91106800	2.49466400	H	5.64022800	1.66361300	-2.49557300
C	-5.62723700	1.74319500	-0.73063100	C	3.59264900	4.67041900	0.72907900
H	-5.85511900	0.55469600	-2.49450500	H	4.47551500	3.81319000	2.49544100
C	-5.87734500	0.51663300	1.40714800	C	4.49108900	3.82643200	-1.40826900
C	-5.13883000	2.89658000	-1.40702100	C	2.54591800	5.32147900	1.40811300
C	-5.62698900	1.74388800	0.73076900	C	3.59262100	4.67038600	-0.72924500
H	-5.85862700	0.51541700	2.49465100	H	4.47543600	3.81308300	-2.49559700
C	-4.43082200	3.87984400	-0.73067500	C	1.38191500	5.72720800	0.72909600
H	-5.12280200	2.88700300	-2.49452600	H	2.53710500	5.30301300	2.49544100
C	-5.15851600	2.86130700	1.40712700	C	2.54587000	5.32140600	-1.40827400
C	-4.43032400	3.88038900	0.73073700	C	1.38188800	5.72717600	-0.72923500
C	-3.51532600	4.73465800	-1.40705200	C	0.16091700	5.89649600	1.40814500
H	-5.14196500	2.85261700	2.49463300	H	2.53701100	5.30288700	-2.49560000
C	-3.54767900	4.71046000	1.40709000	C	0.16087400	5.89644500	-1.40825000
C	-2.46862700	5.34495600	-0.73069800	C	-1.06750600	5.79399000	0.72913700
H	-3.50454400	4.71935400	-2.49455800	H	0.16038200	5.87607000	2.49547500
C	-2.46794800	5.34525500	0.73070700	C	-1.06753400	5.79397200	-0.72919900
H	-3.53604800	4.69572400	2.49459200	H	0.16029700	5.87597800	-2.49558000
C	-1.28471500	5.75382700	-1.40708100	C	-2.25186700	5.45225800	1.40819000
C	-1.32411900	5.74489500	1.40706100	C	-2.25191700	5.45224200	-1.40820300
C	-0.08033500	5.88614200	-0.73072400	C	-3.33253100	4.85921200	0.72917800
H	-1.28107900	5.73548100	-2.49458200	H	-2.24404300	5.43335600	2.49551900
C	-0.07959800	5.88616900	0.73068000	C	-3.33256500	4.85921000	-0.72914400

H	-1.31949400	5.72680400	2.49456700	H	-2.24414100	5.43332300	-2.49553300
C	1.16756100	5.77866000	-1.40710200	C	-4.27566500	4.06550400	1.40823600
H	1.16343100	5.76043100	-2.49460800	H	-4.26081100	4.05139700	2.49556400
C	1.12792600	5.78658200	1.40704100	C	-4.27573600	4.06551800	-1.40816000
H	1.12478700	5.76816700	2.49454300	H	-4.26094500	4.05141500	-2.49548900
**Cyc16_R**				**Cyc16_U**			
C	3.419633000	-5.256967000	0.733059000	C	0.00000000	6.28332500	0.72947700
C	3.419633000	-5.256967000	-0.733059000	C	0.00000000	6.28332500	-0.72947700
C	4.383664000	-4.493077000	1.409891000	C	-1.22712600	6.16918000	1.40826700
C	2.330551000	-5.828618000	1.409891000	C	1.22712600	6.16918000	1.40826700
C	4.383664000	-4.493077000	-1.409891000	C	-1.22712600	6.16918000	-1.40826700
C	2.330551000	-5.828618000	-1.409891000	C	1.22712600	6.16918000	-1.40826700
C	5.171083000	-3.548167000	-0.733059000	C	-2.40452500	5.80503600	-0.72947700
C	5.769404000	-2.473507000	-1.409891000	C	-3.49456000	5.22997800	-1.40826700
C	6.135282000	-1.299191000	-0.733059000	C	-4.44298200	4.44298200	-0.72947700
C	6.135282000	-1.299191000	0.733059000	C	-4.44298200	4.44298200	0.72947700
C	6.276804000	-0.077367000	1.409891000	C	-5.22997800	3.49456000	1.40826700
C	6.165441000	1.147575000	0.733059000	C	-5.80503600	2.40452500	0.72947700
H	6.256088000	-0.077111000	2.497246000	H	-5.21379300	3.48374500	2.49574000
C	5.769404000	-2.473507000	1.409891000	C	-3.49456000	5.22997800	1.40826700
C	5.171083000	-3.548167000	0.733059000	C	-2.40452500	5.80503600	0.72947700
H	5.750362000	-2.465343000	2.497246000	H	-3.48374500	5.21379300	2.49574000
C	6.276804000	-0.077367000	-1.409891000	C	-5.22997800	3.49456000	-1.40826700
H	5.750362000	-2.465343000	-2.497246000	H	-3.48374500	5.21379300	-2.49574000
C	6.165441000	1.147575000	-0.733059000	C	-5.80503600	2.40452500	-0.72947700
C	5.828618000	2.330551000	1.409891000	C	-6.16918000	1.22712600	1.40826700
H	6.256088000	-0.077111000	-2.497246000	H	-5.21379300	3.48374500	-2.49574000
H	4.369196000	-4.478248000	-2.497246000	H	-1.22332900	6.15008900	-2.49574000
H	4.369196000	-4.478248000	2.497246000	H	-1.22332900	6.15008900	2.49574000
C	5.828618000	2.330551000	-1.409891000	C	-6.16918000	1.22712600	-1.40826700
C	5.256967000	3.419633000	0.733059000	C	-6.28332500	0.00000000	0.72947700
C	4.493077000	4.383664000	1.409891000	C	-6.16918000	-1.22712600	1.40826700
C	3.548167000	5.171083000	0.733059000	C	-5.80503600	-2.40452500	0.72947700
C	2.473507000	5.769404000	1.409891000	C	-5.22997800	-3.49456000	1.40826700
H	2.465343000	5.750362000	2.497246000	H	-5.21379300	-3.48374500	2.49574000
C	1.299191000	6.135282000	0.733059000	C	-4.44298200	-4.44298200	0.72947700
C	3.548167000	5.171083000	-0.733059000	C	-5.80503600	-2.40452500	-0.72947700
C	2.473507000	5.769404000	-1.409891000	C	-5.22997800	-3.49456000	-1.40826700
H	2.465343000	5.750362000	-2.497246000	H	-5.21379300	-3.48374500	-2.49574000
C	1.299191000	6.135282000	-0.733059000	C	-4.44298200	-4.44298200	-0.72947700
C	4.493077000	4.383664000	-1.409891000	C	-6.16918000	-1.22712600	-1.40826700
C	5.256967000	3.419633000	-0.733059000	C	-6.28332500	0.00000000	-0.72947700
H	4.478248000	4.369196000	-2.497246000	H	-6.15008900	-1.22332900	-2.49574000
H	4.478248000	4.369196000	2.497246000	H	-6.15008900	-1.22332900	2.49574000
H	5.809380000	2.322859000	-2.497246000	H	-6.15008900	1.22332900	-2.49574000
H	5.809380000	2.322859000	2.497246000	H	-6.15008900	1.22332900	2.49574000
C	1.147575000	-6.165441000	-0.733059000	C	2.40452500	5.80503600	-0.72947700
C	-0.077367000	-6.276804000	-1.409891000	C	3.49456000	5.22997800	-1.40826700
C	-1.299191000	-6.135282000	-0.733059000	C	4.44298200	4.44298200	-0.72947700
C	-1.299191000	-6.135282000	0.733059000	C	4.44298200	4.44298200	0.72947700
C	-0.077367000	-6.276804000	1.409891000	C	3.49456000	5.22997800	1.40826700
H	-0.077111000	-6.256088000	2.497246000	H	3.48374500	5.21379300	2.49574000
C	1.147575000	-6.165441000	0.733059000	C	2.40452500	5.80503600	0.72947700
C	-2.473507000	-5.769404000	1.409891000	C	5.22997800	3.49456000	1.40826700
C	-3.548167000	-5.171083000	0.733059000	C	5.80503600	2.40452500	0.72947700
H	-2.465343000	-5.750362000	2.497246000	H	5.21379300	3.48374500	2.49574000
C	-2.473507000	-5.769404000	-1.409891000	C	5.22997800	3.49456000	-1.40826700
C	-3.548167000	-5.171083000	-0.733059000	C	5.80503600	2.40452500	-0.72947700

H	-2.465343000	-5.750362000	-2.497246000	H	5.21379300	3.48374500	-2.49574000
C	-4.493077000	-4.383664000	-1.409891000	C	6.16918000	1.22712600	-1.40826700
C	-4.493077000	-4.383664000	1.409891000	C	6.16918000	1.22712600	1.40826700
H	-0.077111000	-6.256088000	-2.497246000	H	3.48374500	5.21379300	-2.49574000
C	-5.256967000	-3.419633000	-0.733059000	C	6.28332500	0.00000000	-0.72947700
H	-4.478248000	-4.369196000	-2.497246000	H	6.15008900	1.22332900	-2.49574000
C	-5.256967000	-3.419633000	0.733059000	C	6.28332500	0.00000000	0.72947700
H	-4.478248000	-4.369196000	2.497246000	H	6.15008900	1.22332900	2.49574000
C	-5.828618000	-2.330551000	1.409891000	C	6.16918000	-1.22712600	1.40826700
C	-6.165441000	-1.147575000	0.733059000	C	5.80503600	-2.40452500	0.72947700
C	-6.276804000	0.077367000	1.409891000	C	5.22997800	-3.49456000	1.40826700
C	-6.135282000	1.299191000	0.733059000	C	4.44298200	-4.44298200	0.72947700
H	-6.256088000	0.077111000	2.497246000	H	5.21379300	-3.48374500	2.49574000
C	-5.769404000	2.473507000	1.409891000	C	3.49456000	-5.22997800	1.40826700
C	-6.135282000	1.299191000	-0.733059000	C	4.44298200	-4.44298200	-0.72947700
C	-5.171083000	3.548167000	0.733059000	C	2.40452500	-5.80503600	0.72947700
H	-5.750362000	2.465343000	2.497246000	H	3.48374500	-5.21379300	2.49574000
C	-5.769404000	2.473507000	-1.409891000	C	3.49456000	-5.22997800	-1.40826700
C	-6.276804000	0.077367000	-1.409891000	C	5.22997800	-3.49456000	-1.40826700
C	-5.171083000	3.548167000	-0.733059000	C	2.40452500	-5.80503600	-0.72947700
H	-5.750362000	2.465343000	-2.497246000	H	3.48374500	-5.21379300	-2.49574000
C	-4.383664000	4.493077000	-1.409891000	C	1.22712600	-6.16918000	-1.40826700
C	-4.383664000	4.493077000	1.409891000	C	1.22712600	-6.16918000	1.40826700
C	-6.165441000	-1.147575000	-0.733059000	C	5.80503600	-2.40452500	-0.72947700
H	-6.256088000	0.077111000	-2.497246000	H	5.21379300	-3.48374500	-2.49574000
H	-4.369196000	4.478248000	-2.497246000	H	1.22332900	-6.15008900	-2.49574000
C	-3.419633000	5.256967000	-0.733059000	C	0.00000000	-6.28332500	-0.72947700
H	-4.369196000	4.478248000	2.497246000	H	1.22332900	-6.15008900	2.49574000
C	-3.419633000	5.256967000	0.733059000	C	0.00000000	-6.28332500	0.72947700
C	-5.828618000	-2.330551000	-1.409891000	C	6.16918000	-1.22712600	-1.40826700
C	-2.330551000	5.828618000	1.409891000	C	-1.22712600	-6.16918000	1.40826700
C	-2.330551000	5.828618000	-1.409891000	C	-1.22712600	-6.16918000	-1.40826700
H	-5.809380000	-2.322859000	-2.497246000	H	6.15008900	-1.22332900	-2.49574000
H	-5.809380000	-2.322859000	2.497246000	H	6.15008900	-1.22332900	2.49574000
H	-2.322859000	5.809380000	2.497246000	H	-1.22332900	-6.15008900	2.49574000
C	-1.147575000	6.165441000	0.733059000	C	-2.40452500	-5.80503600	0.72947700
H	-2.322859000	5.809380000	-2.497246000	H	-1.22332900	-6.15008900	-2.49574000
C	-1.147575000	6.165441000	-0.733059000	C	-2.40452500	-5.80503600	-0.72947700
H	2.322859000	-5.809380000	-2.497246000	H	1.22332900	6.15008900	-2.49574000
H	2.322859000	-5.809380000	2.497246000	H	1.22332900	6.15008900	2.49574000
C	0.077367000	6.276804000	-1.409891000	C	-3.49456000	-5.22997800	-1.40826700
H	0.077111000	6.256088000	-2.497246000	H	-3.48374500	-5.21379300	-2.49574000
C	0.077367000	6.276804000	1.409891000	C	-3.49456000	-5.22997800	1.40826700
H	0.077111000	6.256088000	2.497246000	H	-3.48374500	-5.21379300	2.49574000
**Cyc17_R**				**Cyc17_U**			
C	6.18458200	-2.51864400	-1.40659100	C	-2.92387200	6.00574700	1.40820400
C	6.53188900	-1.35123500	-0.73063400	C	-1.76871700	6.43448000	0.72939800
C	5.60204700	-3.61855800	-0.73070100	C	-3.97389900	5.36106600	0.72939200
H	6.16973400	-2.51293000	-2.49417100	H	-2.91595000	5.98949800	2.49558500
C	6.67737900	-0.11512000	-1.40648400	C	-0.55673800	6.65648000	1.40821000
C	6.53201700	-1.35081400	0.73050600	C	-1.76873400	6.43447000	-0.72942000
C	6.57913600	1.09887800	-0.73049800	C	0.67529300	6.63905900	0.72940600
H	6.66154200	-0.11516300	-2.49406600	H	-0.55522100	6.63847800	2.49559300
C	6.67693500	-0.14148900	1.40648600	C	-0.55674600	6.65647300	-1.40822700
C	6.17476300	-2.54309200	1.40637700	C	-2.92387800	6.00573300	-1.40823000
C	6.57908300	1.09930900	0.73064100	C	0.67527500	6.63905900	-0.72941700
H	6.66114400	-0.14083500	2.49406900	H	-0.55525400	6.63846300	-2.49561000
C	6.27697700	2.27923200	1.40659400	C	1.88562100	6.40826000	-1.40822300

C	6.26802300	2.30401500	-1.40637200	C	1.88562900	6.40825700	1.40821900
C	5.60237300	-3.61820700	0.73044400	C	-3.97391100	5.36104700	-0.72942000
C	4.85608100	-4.58143400	-1.40661300	C	-4.89630600	4.54415400	1.40820700
H	6.16032500	-2.53679600	2.49396100	H	-2.91597900	5.98946400	-2.49561100
H	6.26195300	2.27409000	2.49417500	H	1.88051400	6.39094600	-2.49560600
C	5.73715700	3.40073700	0.73071400	C	3.02814200	5.94693400	-0.72941000
H	6.25330400	2.29826400	-2.49395400	H	1.88054600	6.39093700	2.49560200
C	5.73744800	3.40036200	-0.73043500	C	3.02815700	5.94692300	0.72941200
C	4.83826900	-4.60068000	1.40637700	C	-4.89630500	4.54412800	-1.40823300
C	5.01166400	4.41148700	-1.40634600	C	4.07336500	5.29444600	1.40822000
C	5.02871400	4.39154700	1.40663400	C	4.07336400	5.29446200	-1.40821200
C	3.91597200	-5.39621800	0.73047800	C	-5.64263100	3.56371900	-0.72942000
H	4.82721300	-4.58963300	2.49396100	H	-4.88307800	4.53183100	-2.49561500
C	3.91551200	-5.39650100	-0.73064300	C	-5.64262300	3.56374300	0.72939900
C	2.84866600	-6.03642000	1.40643600	C	-6.20759100	2.46872700	-1.40822100
C	2.87228000	-6.02516700	-1.40655600	C	-6.20758800	2.46875100	1.40820700
C	1.70093800	-6.44582600	-0.73060100	C	-6.54926900	1.28493000	0.72940600
H	2.86512300	-6.01102800	-2.49413900	H	-6.19073700	2.46206700	2.49558700
C	0.50112400	-6.65564500	-1.40650600	C	-6.68046700	0.05978700	1.40821900
C	1.70144900	-6.44568100	0.73053500	C	-6.54927400	1.28490400	-0.72941100
C	-0.74310100	-6.62505500	-0.73056600	C	-6.57135800	-1.16751800	0.72941600
H	0.49962900	-6.63995900	-2.49409100	H	-6.66236600	0.05964500	2.49560100
C	-1.93777800	-6.38753900	-1.40647100	C	-6.25099900	-2.35729300	1.40822800
C	-0.74259200	-6.62510800	0.73057600	C	-6.57135700	-1.16754300	-0.72940400
H	-1.93359100	-6.37249300	-2.49405400	H	-6.23407900	-2.35088600	2.49560900
C	-3.08702400	-5.90979500	-0.73052400	C	-5.70572300	-3.46222000	0.72942500
C	0.47501400	-6.65759000	1.40648100	C	-6.68047200	0.05976300	-1.40821500
C	-1.96275300	-6.37984800	1.40653000	C	-6.25100100	-2.35731700	-1.40820900
H	0.47422200	-6.64194800	2.49406500	H	-6.66237700	0.05958300	-2.49559700
H	-1.95779000	-6.36488500	2.49411700	H	-6.23407500	-2.35094600	-2.49559000
C	-3.08648900	-5.90992100	0.73061400	C	-5.70571700	-3.46224300	-0.72940100
H	2.84239500	-6.02215800	2.49401800	H	-6.19075400	2.46200700	-2.49560100
H	4.84432000	-4.57087600	-2.49419700	H	-4.88306900	4.53189300	2.49558800
H	5.00010700	4.40084700	-2.49392800	H	4.06232700	5.28006700	2.49560100
C	4.12061200	5.24186000	-0.73043400	C	4.97209800	4.45155500	0.72941200
H	5.01652400	4.38139500	2.49421600	H	4.06231200	5.28011000	-2.49559300
C	4.12014400	5.24215600	0.73068600	C	4.97208900	4.45157400	-0.72940200
C	-4.13578700	-5.24059900	1.40655100	C	-4.97699900	-4.45581000	-1.40820500
H	-4.12563400	-5.22828800	2.49413000	H	-4.96350800	-4.44375800	-2.49558600
C	-5.01402100	-4.39662100	0.73064300	C	-4.06932400	-5.28905200	-0.72939200
C	-4.11533700	-5.25697400	-1.40642700	C	-4.97699700	-4.45578700	1.40823100
H	-4.10595900	-5.24438000	-2.49401200	H	-4.96351800	-4.44370400	2.49561300
C	-5.01446200	-4.39637800	-0.73049400	C	-4.06933600	-5.28903500	0.72941800
C	3.10180700	5.91041800	1.40657900	C	5.71105000	3.46560200	-1.40820600
C	1.94747600	6.37572800	0.73060900	C	6.24460300	2.35496600	-0.72940200
H	3.09410000	5.89657200	2.49416100	H	5.69558200	3.45622400	-2.49558700
C	0.75659300	6.63147000	1.40648800	C	6.57744000	1.16861800	-1.40820900
C	1.94799800	6.37553900	-0.73052100	C	6.24460800	2.35494400	0.72941000
C	-0.48788900	6.64865700	0.73053800	C	6.67372000	-0.05976400	-0.72940600
H	0.75448300	6.61588700	2.49407400	H	6.55959200	1.16546400	-2.49558800
C	-0.48736000	6.64864400	-0.73060200	C	6.67371800	-0.05978700	0.72940700
C	-1.69081000	6.45719200	1.40643000	C	6.55550300	-1.28622800	-1.40821300
C	0.73060800	6.63434800	-1.40649400	C	6.57743800	1.16859900	1.40821400
C	-1.71603200	6.45039100	-1.40656500	C	6.55549900	-1.28624600	1.40821100
H	0.72923300	6.61876300	-2.49407700	H	6.55959300	1.16541200	2.49559400
C	-2.85756200	6.02395300	0.73048900	C	6.20154200	-2.46643900	-0.72940900
H	-1.68721500	6.44200300	2.49401300	H	6.53771200	-1.28272100	-2.49559300
C	-2.85700300	6.02400900	-0.73064200	C	6.20153500	-2.46646100	0.72940300
H	-1.71165100	6.43524700	-2.49415000	H	6.53770000	-1.28277300	2.49559100
C	-3.93126200	5.39553500	-1.40658200	C	5.64824800	-3.56739400	1.40820700

C	-3.91023900	5.41119400	1.40639100	C	5.64824900	-3.56737500	-1.40821500
C	-4.84180600	4.58580700	0.73047600	C	4.89177700	-4.53998000	-0.72941600
H	-3.90136200	5.39827400	2.49397600	H	5.63294500	-3.55768100	-2.49559700
C	-4.84133300	4.58598800	-0.73066200	C	4.89176600	-4.53999900	0.72940700
C	-5.60194300	3.63405300	1.40636200	C	3.97800600	-5.36656500	-1.40821800
C	-5.61594100	3.61180400	-1.40659200	C	3.97800100	-5.36657900	1.40821000
C	-6.17188100	2.52807600	-0.73067000	C	2.92119400	-6.00009800	0.72940600
H	-5.60239500	3.60349100	-2.49417300	H	3.96720300	-5.35203600	2.49559200
C	-6.54205500	1.33974900	-1.40652800	C	1.77056300	-6.44080500	1.40821800
C	-6.17218000	2.52772500	0.73045900	C	2.92120900	-6.00008700	-0.72941000
C	-6.66870900	0.12836800	-0.73054900	C	0.55628400	-6.64990100	0.72941200
H	-6.52640300	1.33684600	-2.49410400	H	1.76575900	-6.42336100	2.49560000
C	-6.53682800	1.36558800	1.40642000	C	1.77057100	-6.44079800	-1.40821800
C	-6.66872400	0.12795000	0.73057800	C	0.55630200	-6.64989800	-0.72940700
H	-6.52135600	1.36204400	2.49400300	H	1.76579100	-6.42334400	-2.49560000
C	-6.58874500	-1.08741800	1.40654900	C	-0.67584600	-6.64529200	-1.40821000
C	-6.58448500	-1.11341800	-1.40640200	C	-0.67585500	-6.64529200	1.40822200
H	-5.58893600	3.62517000	2.49394500	H	3.96722700	-5.35200000	-2.49560000
H	-6.57301800	-1.08513300	2.49412500	H	-0.67400500	-6.62729600	-2.49559200
C	-6.26450300	-2.28909200	0.73068100	C	-1.88367100	-6.40169800	-0.72939900
H	-6.56890200	-1.11046700	-2.49398500	H	-0.67403900	-6.62729600	2.49560400
C	-6.26477000	-2.28870700	-0.73044800	C	-1.88368800	-6.40169100	0.72941600
H	-3.92158800	5.38283800	-2.49416100	H	5.63293300	-3.55773100	2.49558900
C	3.07867200	5.92255000	-1.40640700	C	5.71105000	3.46558400	1.40821600
H	3.07187200	5.90857100	-2.49399100	H	5.69559600	3.45617800	2.49559700
C	-5.75062600	-3.39340300	1.40658600	C	-3.03098500	-5.95239200	-1.40820500
H	-5.73680400	-3.38565300	2.49416800	H	-3.02278200	-5.93630900	-2.49558700
C	-5.73745200	-3.41611300	-1.40637300	C	-3.03099000	-5.95238100	1.40822700
H	-5.72413100	-3.40773200	-2.49395600	H	-3.02280900	-5.93628000	2.49560900
**Cyc18_R**				**Cyc18_U**			
C	-1.22534700	6.94928700	1.41023800	C	-1.22791700	6.96386600	1.40807000
C	0.00000000	7.05134500	0.73330600	C	0.00000000	7.06512300	0.72948600
C	1.22534700	6.94928700	1.41023800	C	1.22791700	6.96386600	1.40807000
C	0.00000000	7.05134500	-0.73330600	C	0.00000000	7.06512300	-0.72948600
C	1.22534700	6.94928700	-1.41023800	C	1.22791700	6.96386600	-1.40807000
C	2.41170200	6.62609700	-0.73330600	C	2.41641400	6.63904400	-0.72948600
C	2.41170200	6.62609700	0.73330600	C	2.41641400	6.63904400	0.72948600
C	-2.41170200	6.62609700	0.73330600	C	-2.41641400	6.63904400	0.72948600
C	-1.22534700	6.94928700	-1.41023800	C	-1.22791700	6.96386600	-1.40807000
H	1.22206500	6.93067500	-2.49761500	H	1.22494200	6.94699300	-2.49560000
C	3.52824500	6.11110000	-1.41023800	C	3.53564700	6.12392100	-1.40807000
C	3.52824500	6.11110000	1.41023800	C	3.53564700	6.12392100	1.40807000
H	3.51879600	6.09473300	-2.49761500	H	3.52708100	6.10908300	-2.49560000
H	3.51879600	6.09473300	2.49761500	H	3.52708100	6.10908300	2.49560000
C	-2.41170200	6.62609700	-0.73330600	C	-2.41641400	6.63904400	-0.72948600
H	-1.22206500	6.93067500	2.49761500	H	-1.22494200	6.94699300	2.49560000
H	1.22206500	6.93067500	2.49761500	H	1.22494200	6.94699300	2.49560000
H	-1.22206500	6.93067500	-2.49761500	H	-1.22494200	6.94699300	-2.49560000
C	4.53251800	5.40164400	0.73330600	C	4.54137400	5.41219800	0.72948600
C	4.53251800	5.40164400	-0.73330600	C	4.54137400	5.41219800	-0.72948600
C	-3.52824500	6.11110000	-1.41023800	C	-3.53564700	6.12392100	-1.40807000
C	-4.53251800	5.40164400	-0.73330600	C	-4.54137400	5.41219800	-0.72948600
C	-5.40558600	4.53582500	-1.41023800	C	-5.41692600	4.54534100	-1.40807000
C	-6.10664400	3.52567300	-0.73330600	C	-6.11857600	3.53256100	-0.72948600
C	-6.63093200	2.41346200	-1.41023800	C	-6.64484400	2.41852500	-1.40807000
C	-6.94422000	1.22445300	-0.73330600	C	-6.95778800	1.22684600	-0.72948600
C	-6.94422000	1.22445300	0.73330600	C	-6.95778800	1.22684600	0.72948600
C	-7.05649100	0.00000000	1.41023800	C	-7.07129500	0.00000000	1.40807000

C	-6.63093200	2.41346200	1.41023800	C	-6.64484400	2.41852500	1.40807000
C	-6.10664400	3.52567300	0.73330600	C	-6.11857600	3.53256100	0.72948600
C	-7.05649100	0.00000000	-1.41023800	C	-7.07129500	0.00000000	-1.40807000
C	-5.40558600	4.53582500	1.41023800	C	-5.41692600	4.54534100	1.40807000
H	-7.03759200	0.00000000	2.49761500	H	-7.05416200	0.00000000	2.49560000
H	-7.03759200	0.00000000	-2.49761500	H	-7.05416200	0.00000000	-2.49560000
C	-4.53251800	5.40164400	0.73330600	C	-4.54137400	5.41219800	0.72948600
C	-3.52824500	6.11110000	1.41023800	C	-3.53564700	6.12392100	1.40807000
H	-6.61317300	2.40699800	-2.49761500	H	-6.62874400	2.41266500	-2.49560000
H	-6.61317300	2.40699800	2.49761500	H	-6.62874400	2.41266500	2.49560000
H	-5.39110800	4.52367700	2.49761500	H	-5.40380100	4.53432800	2.49560000
H	-5.39110800	4.52367700	-2.49761500	H	-5.40380100	4.53432800	-2.49560000
H	-3.51879600	6.09473300	-2.49761500	H	-3.52708100	6.10908300	-2.49560000
H	-3.51879600	6.09473300	2.49761500	H	-3.52708100	6.10908300	2.49560000
C	5.40558600	4.53582500	1.41023800	C	5.41692600	4.54534100	1.40807000
C	5.40558600	4.53582500	-1.41023800	C	5.41692600	4.54534100	-1.40807000
H	5.39110800	4.52367700	2.49761500	H	5.40380100	4.53432800	2.49560000
H	5.39110800	4.52367700	-2.49761500	H	5.40380100	4.53432800	-2.49560000
C	-6.94422000	-1.22445300	-0.73330600	C	-6.95778800	-1.22684600	-0.72948600
C	-6.63093200	-2.41346200	-1.41023800	C	-6.64484400	-2.41852500	-1.40807000
C	-6.94422000	-1.22445300	0.73330600	C	-6.95778800	-1.22684600	0.72948600
C	-6.63093200	-2.41346200	1.41023800	C	-6.64484400	-2.41852500	1.40807000
H	-6.61317300	-2.40699800	-2.49761500	H	-6.62874400	-2.41266500	-2.49560000
H	-6.61317300	-2.40699800	2.49761500	H	-6.62874400	-2.41266500	2.49560000
C	6.10664400	3.52567300	-0.73330600	C	6.11857600	3.53256100	-0.72948600
C	6.63093200	2.41346200	-1.41023800	C	6.64484400	2.41852500	-1.40807000
C	6.94422000	1.22445300	-0.73330600	C	6.95778800	1.22684600	-0.72948600
C	7.05649100	0.00000000	-1.41023800	C	7.07129500	0.00000000	-1.40807000
C	6.94422000	-1.22445300	-0.73330600	C	6.95778800	-1.22684600	-0.72948600
C	6.94422000	-1.22445300	0.73330600	C	6.95778800	-1.22684600	0.72948600
C	7.05649100	0.00000000	1.41023800	C	7.07129500	0.00000000	1.40807000
C	6.63093200	-2.41346200	-1.41023800	C	6.64484400	-2.41852500	-1.40807000
C	6.63093200	-2.41346200	1.41023800	C	6.64484400	-2.41852500	1.40807000
C	6.10664400	-3.52567300	0.73330600	C	6.11857600	-3.53256100	0.72948600
C	6.10664400	-3.52567300	-0.73330600	C	6.11857600	-3.53256100	-0.72948600
C	5.40558600	-4.53582500	-1.41023800	C	5.41692600	-4.54534100	-1.40807000
C	4.53251800	-5.40164400	-0.73330600	C	4.54137400	-5.41219800	-0.72948600
C	3.52824500	-6.11110000	-1.41023800	C	3.53564700	-6.12392100	-1.40807000
C	2.41170200	-6.62609700	-0.73330600	C	2.41641400	-6.63904400	-0.72948600
C	2.41170200	-6.62609700	0.73330600	C	2.41641400	-6.63904400	0.72948600
C	3.52824500	-6.11110000	1.41023800	C	3.53564700	-6.12392100	1.40807000
C	1.22534700	-6.94928700	1.41023800	C	1.22791700	-6.96386600	1.40807000
C	1.22534700	-6.94928700	-1.41023800	C	1.22791700	-6.96386600	-1.40807000
C	0.00000000	-7.05134500	-0.73330600	C	0.00000000	-7.06512300	-0.72948600
C	0.00000000	-7.05134500	0.73330600	C	0.00000000	-7.06512300	0.72948600
C	4.53251800	-5.40164400	0.73330600	C	4.54137400	-5.41219800	0.72948600
H	1.22206500	-6.93067500	-2.49761500	H	1.22494200	-6.94699300	-2.49560000
C	-1.22534700	-6.94928700	-1.41023800	C	-1.22791700	-6.96386600	-1.40807000
C	-1.22534700	-6.94928700	1.41023800	C	-1.22791700	-6.96386600	1.40807000
H	-1.22206500	-6.93067500	-2.49761500	H	-1.22494200	-6.94699300	-2.49560000
H	-1.22206500	-6.93067500	2.49761500	H	-1.22494200	-6.94699300	2.49560000
H	3.51879600	-6.09473300	2.49761500	H	3.52708100	-6.10908300	2.49560000
H	1.22206500	-6.93067500	2.49761500	H	1.22494200	-6.94699300	2.49560000
H	3.51879600	-6.09473300	-2.49761500	H	3.52708100	-6.10908300	-2.49560000
C	5.40558600	-4.53582500	1.41023800	C	5.41692600	-4.54534100	1.40807000
H	7.03759200	0.00000000	-2.49761500	H	7.05416200	0.00000000	-2.49560000
H	7.03759200	0.00000000	2.49761500	H	7.05416200	0.00000000	2.49560000
H	6.61317300	-2.40699800	2.49761500	H	6.62874400	-2.41266500	2.49560000
H	6.61317300	-2.40699800	-2.49761500	H	6.62874400	-2.41266500	-2.49560000
H	5.39110800	-4.52367700	-2.49761500	H	5.40380100	-4.53432800	-2.49560000

H	5.39110800	-4.52367700	2.49761500	H	5.40380100	-4.53432800	2.49560000
C	6.10664400	3.52567300	0.73330600	C	6.11857600	3.53256100	0.72948600
C	6.63093200	2.41346200	1.41023800	C	6.64484400	2.41852500	1.40807000
C	6.94422000	1.22445300	0.73330600	C	6.95778800	1.22684600	0.72948600
H	6.61317300	2.40699800	-2.49761500	H	6.62874400	2.41266500	-2.49560000
H	6.61317300	2.40699800	2.49761500	H	6.62874400	2.41266500	2.49560000
C	-2.41170200	-6.62609700	-0.73330600	C	-2.41641400	-6.63904400	-0.72948600
C	-3.52824500	-6.11110000	-1.41023800	C	-3.53564700	-6.12392100	-1.40807000
C	-2.41170200	-6.62609700	0.73330600	C	-2.41641400	-6.63904400	0.72948600
C	-3.52824500	-6.11110000	1.41023800	C	-3.53564700	-6.12392100	1.40807000
H	-3.51879600	-6.09473300	-2.49761500	H	-3.52708100	-6.10908300	-2.49560000
H	-3.51879600	-6.09473300	2.49761500	H	-3.52708100	-6.10908300	2.49560000
C	-4.53251800	-5.40164400	-0.73330600	C	-4.54137400	-5.41219800	-0.72948600
C	-5.40558600	-4.53582500	-1.41023800	C	-5.41692600	-4.54534100	-1.40807000
C	-6.10664400	-3.52567300	-0.73330600	C	-6.11857600	-3.53256100	-0.72948600
C	-6.10664400	-3.52567300	0.73330600	C	-6.11857600	-3.53256100	0.72948600
C	-5.40558600	-4.53582500	1.41023800	C	-5.41692600	-4.54534100	1.40807000
C	-4.53251800	-5.40164400	0.73330600	C	-4.54137400	-5.41219800	0.72948600
H	-5.39110800	-4.52367700	-2.49761500	H	-5.40380100	-4.53432800	-2.49560000
H	-5.39110800	-4.52367700	2.49761500	H	-5.40380100	-4.53432800	2.49560000
**Cyc19_R**				**Cyc19_U**			
C	1.22701100	1.40655100	7.35168500	C	7.43783800	-0.61514600	1.40817100
C	0.00000000	0.73078800	7.44630500	C	7.43185200	0.61669300	0.72948600
C	-1.22701100	1.40655100	7.35168500	C	7.23463200	1.83264900	1.40817400
C	0.00000000	-0.73078800	7.44630500	C	7.43185200	0.61669300	-0.72948600
C	-1.22701100	-1.40655100	7.35168500	C	7.23463200	1.83264900	-1.40817400
C	-2.41827500	-0.73080100	7.04288000	C	6.82873900	2.99570000	-0.72948200
C	-2.41827500	0.73080100	7.04288000	C	6.82873900	2.99570000	0.72948200
C	2.41827500	0.73080100	7.04288000	C	7.22922800	-1.82920100	0.72948300
C	1.22701100	-1.40655100	7.35168500	C	7.43783800	-0.61514600	-1.40817100
H	-1.22463800	-2.49411500	7.33755900	H	7.21890900	1.82866700	-2.49557100
C	-3.54815000	-1.40656500	6.55505000	C	6.24721400	4.08164400	-1.40816500
C	-3.54815000	1.40656500	6.55505000	C	6.24721400	4.08164400	1.40816500
H	-3.54136300	-2.49412600	6.54252400	H	6.23367100	4.07277700	-2.49556400
H	-3.54136300	2.49412600	6.54252400	H	6.23367100	4.07277700	2.49556400
C	2.41827500	-0.73080100	7.04288000	C	7.22922800	-1.82920100	-0.72948300
H	1.22463800	2.49411500	7.33755900	H	7.42170400	-0.61381200	2.49556900
H	-1.22463800	2.49411500	7.33755900	H	7.21890900	1.82866700	2.49557100
H	1.22463800	-2.49411500	7.33755900	H	7.42170400	-0.61381200	-2.49556900
C	-4.57463700	0.73077100	5.87631400	C	5.48549000	5.04976700	0.72948600
C	-4.57463700	-0.73077100	5.87631400	C	5.48549000	5.04976700	-0.72948600
C	3.54815000	-1.40656500	6.55505000	C	6.83468600	-2.99615200	-1.40817400
C	4.57463700	-0.73077100	5.87631400	C	6.24301900	-4.07660500	-0.72948800
C	5.48514000	-1.40654500	5.04832800	C	5.49077300	-5.05210800	-1.40817600
C	6.23579400	-0.73080200	4.07318000	C	4.58028900	-5.88181600	-0.72947800
C	6.82804100	-1.40654100	2.99433500	C	3.55218800	-6.56036500	-1.40817100
C	7.22122500	-0.73077900	1.82825800	C	2.42175000	-7.04981000	-0.72948400
C	7.22122500	0.73077900	1.82825800	C	2.42175000	-7.04981000	0.72948400
C	7.43091900	1.40656700	0.61553800	C	1.22906300	-7.35794000	1.40817000
C	6.82804100	1.40654100	2.99433500	C	3.55218800	-6.56036500	1.40817100
C	6.23579400	0.73080200	4.07318000	C	4.58028900	-5.88181600	0.72947800
C	7.43091900	-1.40656700	0.61553800	C	1.22906300	-7.35794000	-1.40817000
C	5.48514000	1.40654500	5.04832800	C	5.49077300	-5.05210800	1.40817600
H	7.41666500	2.49413500	0.61447500	H	1.22638900	-7.34193300	2.49556600
H	7.41666500	-2.49413500	0.61447500	H	1.22638900	-7.34193300	-2.49556600
C	4.57463700	0.73077100	5.87631400	C	6.24301900	-4.07660500	0.72948800
C	3.54815000	1.40656500	6.55505000	C	6.83468600	-2.99615200	1.40817400
H	6.81493800	-2.49410700	2.98857400	H	3.54448600	-6.54612500	-2.49556800

H	6.81493800	2.49410700	2.98857400	H	3.54448600	-6.54612500	2.49556800
H	5.47460000	2.49413000	5.03873100	H	5.47887700	-5.04116700	2.49557300
H	5.47460000	-2.49413000	5.03873100	H	5.47887700	-5.04116700	-2.49557300
H	3.54136300	-2.49412600	6.54252400	H	6.81983700	-2.98963200	-2.49557100
H	3.54136300	2.49412600	6.54252400	H	6.81983700	-2.98963200	2.49557100
C	-5.48514000	1.40654500	5.04832800	C	4.58269200	5.88786000	1.40817200
C	-5.48514000	-1.40654500	5.04832800	C	4.58269200	5.88786000	-1.40817200
H	-5.47460000	2.49413000	5.03873100	H	4.57272200	5.87503400	2.49556900
H	-5.47460000	-2.49413000	5.03873100	H	4.57272200	5.87503400	-2.49556900
C	-6.23579400	-0.73080200	4.07318000	C	3.54785500	6.55610300	-0.72948600
C	-6.82804100	-1.40654100	2.99433500	C	2.42189200	7.05575500	-1.40817200
C	-7.22122500	-0.73077900	1.82825800	C	1.22619000	7.35197300	-0.72948800
C	-7.43091900	-1.40656700	0.61553800	C	-0.00096800	7.45928100	-1.40817600
C	-7.42393600	-0.73084200	-0.61506900	C	-1.22809800	7.35165500	-0.72948800
C	-7.42393600	0.73084200	-0.61506900	C	-1.22809800	7.35165500	0.72948800
C	-7.43091900	1.40656700	0.61553800	C	-0.00096800	7.45928100	1.40817600
C	-7.22824000	-1.40656300	-1.83010300	C	-2.42372400	7.05512700	-1.40817200
C	-7.22824000	1.40656300	-1.83010300	C	-2.42372400	7.05512700	1.40817200
C	-6.82180900	0.73080000	-2.99168700	C	-3.54955700	6.55518300	0.72948600
C	-6.82180900	-0.73080000	-2.99168700	C	-3.54955700	6.55518300	-0.72948600
C	-6.24206100	-1.40656200	-4.07721300	C	-4.58422000	5.88667200	-1.40817200
C	-5.48030500	-0.73081700	-5.04381900	C	-5.48680100	5.04834400	-0.72948600
C	-4.57932100	-1.40656100	-5.88207100	C	-6.24827400	4.08002400	-1.40816500
C	-3.54494500	-0.73080600	-6.54887900	C	-6.82951700	2.99392900	-0.72948200
C	-3.54494500	0.73080600	-6.54887900	C	-6.82951700	2.99392900	0.72948200
C	-4.57932100	1.40656100	-5.88207100	C	-6.24827400	4.08002400	1.40816500
C	-2.42064100	1.40654900	-7.04925700	C	-7.23510800	1.83077200	1.40817400
C	-2.42064100	-1.40654900	-7.04925700	C	-7.23510800	1.83077200	-1.40817400
C	-1.22584600	-0.73080000	-7.34425400	C	-7.43201100	0.61476500	-0.72948600
C	-1.22584600	0.73080000	-7.34425400	C	-7.43201100	0.61476500	0.72948600
C	-5.48030500	0.73081700	-5.04381900	C	-5.48680100	5.04834400	0.72948600
H	-2.41602300	-2.49412500	-7.03580700	H	-7.21938300	1.82679400	-2.49557100
C	0.00000000	-1.40655900	-7.45276100	C	-7.43767800	-0.61707600	-1.40817100
C	0.00000000	1.40655900	-7.45276100	C	-7.43767800	-0.61707600	1.40817100
H	0.00000000	-2.49412400	-7.43852800	H	-7.42154400	-0.61573800	-2.49556900
H	0.00000000	2.49412400	-7.43852800	H	-7.42154400	-0.61573800	2.49556900
H	-4.57053700	2.49410800	-5.87076300	H	-6.23472900	4.07116000	2.49556400
H	-2.41602300	2.49412500	-7.03580700	H	-7.21938300	1.82679400	2.49557100
H	-4.57053700	-2.49410800	-5.87076300	H	-6.23472900	4.07116000	-2.49556400
C	-6.24206100	1.40656200	-4.07721300	C	-4.58422000	5.88667200	1.40817200
H	-7.41666500	-2.49413500	0.61447500	H	-0.00096600	7.44310500	-2.49557400
H	-7.41666500	2.49413500	0.61447500	H	-0.00096600	7.44310500	2.49557400
H	-7.21438800	2.49410300	-1.82656700	H	-2.41848200	7.03985400	2.49557100
H	-7.21438800	-2.49410300	-1.82656700	H	-2.41848200	7.03985400	-2.49557100
H	-6.23011300	-2.49413000	-4.06939400	H	-4.57424800	5.87384800	-2.49556900
H	-6.23011300	2.49413000	-4.06939400	H	-4.57424800	5.87384800	2.49556900
C	-6.23579400	0.73080200	4.07318000	C	3.54785500	6.55610300	0.72948600
C	-6.82804100	1.40654100	2.99433500	C	2.42189200	7.05575500	1.40817200
C	-7.22122500	0.73077900	1.82825800	C	1.22619000	7.35197300	0.72948800
H	-6.81493800	-2.49410700	2.98857400	H	2.41665500	7.04048100	-2.49557100
H	-6.81493800	2.49410700	2.98857400	H	2.41665500	7.04048100	2.49557100
C	1.22584600	-0.73080000	-7.34425400	C	-7.22875200	-1.83107700	-0.72948300
C	2.42064100	-1.40654900	-7.04925700	C	-6.83390700	-2.99792500	-1.40817400
C	1.22584600	0.73080000	-7.34425400	C	-7.22875200	-1.83107700	0.72948300
C	2.42064100	1.40654900	-7.04925700	C	-6.83390700	-2.99792500	1.40817400
H	2.41602300	-2.49412500	-7.03580700	H	-6.81906000	-2.99140000	-2.49557100
H	2.41602300	2.49412500	-7.03580700	H	-6.81906000	-2.99140000	2.49557100
C	3.54494500	-0.73080600	-6.54887900	C	-6.24196000	-4.07822400	-0.72948800
C	4.57932100	-1.40656100	-5.88207100	C	-5.48946100	-5.05353200	-1.40817600
C	5.48030500	-0.73081700	-5.04381900	C	-4.57876200	-5.88300400	-0.72947800

C	6.24206100	-1.40656200	-4.07721300	C	-3.55048500	-6.56128600	-1.40817100
H	6.23011300	-2.49413000	-4.06939400	H	-3.54278600	-6.54704400	-2.49556800
C	5.48030500	0.73081700	-5.04381900	C	-4.57876200	-5.88300400	0.72947800
C	6.24206100	1.40656200	-4.07721300	C	-3.55048500	-6.56128600	1.40817100
H	6.23011300	2.49413000	-4.06939400	H	-3.54278600	-6.54704400	2.49556800
C	4.57932100	1.40656100	-5.88207100	C	-5.48946100	-5.05353200	1.40817600
C	3.54494500	0.73080600	-6.54887900	C	-6.24196000	-4.07822400	0.72948800
H	4.57053700	-2.49410800	-5.87076300	H	-5.47756800	-5.04258800	-2.49557300
H	4.57053700	2.49410800	-5.87076300	H	-5.47756800	-5.04258800	2.49557300
C	6.82180900	-0.73080000	-2.99168700	C	-2.41992000	-7.05043800	-0.72948400
C	7.22824000	-1.40656300	-1.83010300	C	-1.22715300	-7.35825800	-1.40817000
C	7.42393600	-0.73084200	-0.61506900	C	0.00096700	-7.45393600	-0.72948100
C	7.42393600	0.73084200	-0.61506900	C	0.00096700	-7.45393600	0.72948100
C	7.22824000	1.40656300	-1.83010300	C	-1.22715300	-7.35825800	1.40817000
C	6.82180900	0.73080000	-2.99168700	C	-2.41992000	-7.05043800	0.72948400
H	7.21438800	-2.49410300	-1.82656700	H	-1.22448300	-7.34225000	-2.49556600
H	7.21438800	2.49410300	-1.82656700	H	-1.22448300	-7.34225000	2.49556600
**Cyc20_R**				**Cyc20_U**			
C	1.22584400	7.73967600	1.41062200	C	4.76118000	-6.24471500	1.40813800
C	0.00000000	7.83162400	0.73347900	C	0.18125500	-7.84989500	1.40814700
C	0.00000000	7.83162400	-0.73347900	C	0.18125400	-7.84991500	-1.40816000
C	1.22584400	7.73967600	-1.41062200	C	-1.04828200	-7.77608400	-0.72953200
H	1.22312900	7.72253500	2.49801700	C	-1.04827900	-7.77608400	0.72953200
H	1.22312900	7.72253500	-2.49801700	C	5.67543600	-5.41925600	0.72953100
C	-1.22584400	7.73967600	-1.41062200	C	4.76119300	-6.24473300	-1.40815500
C	-2.42010500	7.44831700	-0.73347900	H	0.18089800	-7.83452300	-2.49560400
C	-2.42010500	7.44831700	0.73347900	C	-2.25353200	-7.52194000	-1.40814300
C	-3.55753900	6.98206300	1.41062200	C	-2.25353600	-7.52196100	1.40815700
C	-4.60331300	6.33591700	0.73347900	H	-2.24911200	-7.50716400	-2.49560500
C	-5.54099600	5.54099600	1.41062200	H	-2.24913100	-7.50724000	2.49560100
C	-6.33591700	4.60331300	0.73347900	C	5.67543500	-5.41925300	-0.72953600
C	-6.98206300	3.55753900	1.41062200	H	4.75178300	-6.23240100	2.49559900
C	-6.33591700	4.60331300	-0.73347900	H	0.18090000	-7.83444900	2.49560800
C	-5.54099600	5.54099600	-1.41062200	H	4.75183200	-6.23246400	-2.49560000
C	-4.60331300	6.33591700	-0.73347900	C	-3.40013200	-7.07192100	0.72952800
C	-6.98206300	3.55753900	-1.41062200	C	-3.40013200	-7.07192400	-0.72952500
C	-3.55753900	6.98206300	-1.41062200	C	7.40989900	2.59830500	1.40814700
H	-6.96660000	3.54966000	2.49801700	C	7.71940300	1.40607100	0.72953300
H	-6.96660000	3.54966000	-2.49801700	C	7.84991700	0.18125400	1.40816000
C	-1.22584400	7.73967600	1.41062200	C	7.71940400	1.40607100	-0.72953000
H	-5.52872500	5.52872500	-2.49801700	C	7.84989700	0.18125500	-1.40814800
H	-5.52872500	5.52872500	2.49801700	C	7.77608600	-1.04827900	-0.72953200
H	-3.54966000	6.96660000	2.49801700	C	7.77608600	-1.04828200	0.72953200
H	-3.54966000	6.96660000	-2.49801700	C	7.40992200	2.59831500	-1.40815300
H	-1.22312900	7.72253500	-2.49801700	C	7.52196300	-2.25353700	-1.40815700
H	-1.22312900	7.72253500	2.49801700	C	7.52194200	-2.25353200	1.40814300
C	-7.44831700	2.42010500	-0.73347900	H	7.39534500	2.59320600	2.49560800
C	-7.73967600	1.22584400	-1.41062200	H	7.39542400	2.59323700	-2.49559800
C	-7.83162400	0.00000000	-0.73347900	C	7.07192600	-3.40013200	0.72952500
C	-7.73967600	-1.22584400	-1.41062200	C	7.07192300	-3.40013200	-0.72952800
H	-7.72253500	-1.22312900	-2.49801700	C	6.45782700	-4.46787700	-1.40814200
C	-7.44831700	-2.42010500	-0.73347900	C	6.45785000	-4.46789400	1.40814800
C	-6.98206300	-3.55753900	-1.41062200	H	7.83445100	0.18090000	-2.49560800
C	-6.33591700	-4.60331300	-0.73347900	H	7.83452400	0.18089800	2.49560400
C	-5.54099600	-5.54099600	-1.41062200	H	7.50716700	-2.24911200	2.49560500
C	-4.60331300	-6.33591700	-0.73347900	H	7.50724300	-2.24913200	-2.49560200
C	-3.55753900	-6.98206300	-1.41062200	H	6.44510100	-4.45906200	-2.49560400
C	-2.42010500	-7.44831700	-0.73347900	H	6.44517200	-4.45911500	2.49559400

C	-1.22584400	-7.73967600	-1.41062200	C	-6.45784900	4.46789300	1.40814800
C	0.00000000	-7.83162400	-0.73347900	C	-5.67543500	5.41925500	0.72953100
C	0.00000000	-7.83162400	0.73347900	C	-4.76117900	6.24471400	1.40813800
C	1.22584400	-7.73967600	1.41062200	C	-5.67543500	5.41925300	-0.72953600
C	-1.22584400	-7.73967600	1.41062200	C	-4.76119200	6.24473200	-1.40815500
C	-2.42010500	-7.44831700	0.73347900	C	-3.72291300	6.90743800	-0.72953000
C	2.42010500	-7.44831700	0.73347900	C	-3.72291600	6.90743800	0.72952400
C	1.22584400	-7.73967600	-1.41062200	C	-7.07192500	3.40013200	0.72952500
H	-1.22312900	-7.72253500	-2.49801700	C	-6.45782700	4.46787700	-1.40814200
C	-3.55753900	-6.98206300	1.41062200	H	-4.75183100	6.23246300	-2.49560000
H	-3.54966000	-6.96660000	-2.49801700	C	-2.59830500	7.40989700	-1.40814600
H	-3.54966000	-6.96660000	2.49801700	C	-2.59831500	7.40992100	1.40815300
C	2.42010500	-7.44831700	-0.73347900	H	-2.59320600	7.39534300	-2.49560800
H	1.22312900	-7.72253500	2.49801700	H	-2.59323700	7.39542200	2.49559800
H	-1.22312900	-7.72253500	2.49801700	C	-7.07192300	3.40013200	-0.72952800
H	1.22312900	-7.72253500	-2.49801700	H	-6.44517200	4.45911500	2.49559400
C	3.55753900	-6.98206300	-1.41062200	H	-4.75178200	6.23240000	2.49559900
C	4.60331300	-6.33591700	-0.73347900	H	-6.44510000	4.45906100	-2.49560400
C	4.60331300	-6.33591700	0.73347900	C	-4.46787700	-6.45782600	1.40814200
C	5.54099600	-5.54099600	1.41062200	C	-7.40989900	-2.59830500	1.40814700
C	6.33591700	-4.60331300	0.73347900	C	-7.40992300	-2.59831500	-1.40815300
C	6.98206300	-3.55753900	1.41062200	C	-7.71940500	-1.40607100	-0.72953000
C	6.33591700	-4.60331300	-0.73347900	C	-7.71940300	-1.40607100	0.72953300
C	6.98206300	-3.55753900	-1.41062200	C	-4.46789400	-6.45784800	-1.40814800
C	5.54099600	-5.54099600	-1.41062200	C	-7.84989700	-0.18125600	-1.40814800
C	3.55753900	-6.98206300	1.41062200	C	-7.84991700	-0.18125400	1.40816000
H	6.96660000	-3.54966000	-2.49801700	H	-4.45906100	-6.44509900	2.49560400
H	6.96660000	-3.54966000	2.49801700	H	-4.45911500	-6.44517000	-2.49559400
H	5.52872500	-5.52872500	2.49801700	C	-7.77608600	1.04828200	0.72953200
H	5.52872500	-5.52872500	-2.49801700	C	-7.77608700	1.04827900	-0.72953200
H	3.54966000	-6.96660000	-2.49801700	C	-7.52196300	2.25353700	-1.40815700
H	3.54966000	-6.96660000	2.49801700	C	-7.52194200	2.25353200	1.40814300
C	7.44831700	-2.42010500	-0.73347900	H	-7.39542500	-2.59323700	-2.49559800
C	7.73967600	-1.22584400	-1.41062200	H	-7.39534600	-2.59320600	2.49560800
C	7.83162400	0.00000000	-0.73347900	H	-7.83452500	-0.18089800	2.49560400
C	7.73967600	1.22584400	-1.41062200	H	-7.83445200	-0.18090000	-2.49560800
C	7.44831700	2.42010500	-0.73347900	H	-7.50724300	2.24913200	-2.49560200
C	7.44831700	2.42010500	0.73347900	H	-7.50716700	2.24911200	2.49560500
C	6.98206300	3.55753900	1.41062200	C	4.46789400	6.45784900	-1.40814800
C	6.33591700	4.60331300	0.73347900	C	4.46787700	6.45782600	1.40814200
C	5.54099600	5.54099600	1.41062200	H	4.45911500	6.44517100	-2.49559400
C	6.33591700	4.60331300	-0.73347900	H	4.45906200	6.44509900	2.49560400
C	5.54099600	5.54099600	-1.41062200	C	-6.90744000	-3.72291600	-0.72952400
H	5.52872500	5.52872500	-2.49801700	C	-6.24471600	-4.76117900	-1.40813800
C	6.98206300	3.55753900	-1.41062200	C	-5.41925600	-5.67543500	-0.72953100
H	6.96660000	3.54966000	-2.49801700	C	-5.41925400	-5.67543400	0.72953600
H	6.96660000	3.54966000	2.49801700	C	-6.24473300	-4.76119200	1.40815500
H	5.52872500	5.52872500	2.49801700	C	-6.90744000	-3.72291400	0.72953000
C	7.73967600	1.22584400	1.41062200	H	-6.23240200	-4.75178300	-2.49559900
H	7.72253500	1.22312900	2.49801700	H	-6.23246400	-4.75183200	2.49560000
H	7.72253500	1.22312900	-2.49801700	C	-1.40607100	7.71940100	-0.72953300
C	7.83162400	0.00000000	0.73347900	C	-0.18125400	7.84991500	-1.40816000
C	7.73967600	-1.22584400	1.41062200	C	-0.18125500	7.84989500	1.40814800
C	7.44831700	-2.42010500	0.73347900	C	-1.40607100	7.71940300	0.72953000
H	7.72253500	-1.22312900	-2.49801700	H	-0.18089800	7.83452300	-2.49560400
H	7.72253500	-1.22312900	2.49801700	H	-0.18090000	7.83445000	2.49560800
C	-4.60331300	-6.33591700	0.73347900	C	1.04828200	7.77608400	-0.72953200
C	-5.54099600	-5.54099600	1.41062200	C	2.25353200	7.52194100	-1.40814300
H	-5.52872500	-5.52872500	-2.49801700	C	3.40013200	7.07192400	-0.72952500
H	-5.52872500	-5.52872500	2.49801700	C	3.40013200	7.07192200	0.72952800

C	-7.44831700	-2.42010500	0.73347900	C	2.25353700	7.52196100	1.40815700
C	-7.73967600	-1.22584400	1.41062200	C	1.04827900	7.77608500	0.72953200
H	-7.72253500	-1.22312900	2.49801700	H	2.24911200	7.50716600	-2.49560500
C	-6.98206300	-3.55753900	1.41062200	H	2.24913200	7.50724100	2.49560100
C	-6.33591700	-4.60331300	0.73347900	C	5.41925600	5.67543500	-0.72953100
H	-6.96660000	-3.54966000	-2.49801700	C	6.24471500	4.76117900	-1.40813800
H	-6.96660000	-3.54966000	2.49801700	C	6.90743900	3.72291600	-0.72952400
C	-7.44831700	2.42010500	0.73347900	C	6.90743900	3.72291300	0.72953000
C	-7.73967600	1.22584400	1.41062200	C	6.24473300	4.76119200	1.40815500
C	-7.83162400	0.00000000	0.73347900	C	5.41925400	5.67543400	0.72953600
H	-7.72253500	1.22312900	-2.49801700	H	6.23240100	4.75178200	-2.49559900
H	-7.72253500	1.22312900	2.49801700	H	6.23246400	4.75183200	2.49560000
C	4.60331300	6.33591700	-0.73347900	C	1.40607100	-7.71940100	-0.72953300
C	3.55753900	6.98206300	-1.41062200	C	2.59830500	-7.40989800	-1.40814600
C	2.42010500	7.44831700	-0.73347900	C	3.72291400	-6.90743800	-0.72953000
C	2.42010500	7.44831700	0.73347900	C	3.72291600	-6.90743800	0.72952400
C	3.55753900	6.98206300	1.41062200	C	2.59831500	-7.40992100	1.40815300
C	4.60331300	6.33591700	0.73347900	C	1.40607100	-7.71940200	0.72953000
H	3.54966000	6.96660000	-2.49801700	H	2.59320600	-7.39534400	-2.49560800
H	3.54966000	6.96660000	2.49801700	H	2.59323700	-7.39542300	2.49559800

Table S9. Cartesian coordinates of the optimized geometries of [n]-cyclacene at M06-2X/6-31G(d) level of theory.

RM06-2X/6-31G(d)				UM06-2X/6-31G(d)			
**Cyc6_R**				**Cyc6_U**			
C	0.00000000	2.38827400	0.72435300	C	0.00000000	2.39219900	0.72413400
C	1.20241100	2.08263700	1.39980000	C	1.20205500	2.08202100	1.40405100
C	2.06830600	1.19413700	0.72435300	C	0.00000000	2.39219900	-0.72413400
C	2.06830600	1.19413700	-0.72435300	C	-1.20205500	2.08202100	1.40405100
C	1.20241100	2.08263700	-1.39980000	C	2.07170500	1.19609900	0.72413400
C	0.00000000	2.38827400	-0.72435300	H	1.17951800	2.04298500	2.49053600
C	2.40482300	0.00000000	1.39980000	C	1.20205500	2.08202100	-1.40405100
C	2.40482300	0.00000000	-1.39980000	C	-1.20205500	2.08202100	-1.40405100
C	2.06830600	-1.19413700	-0.72435300	C	-2.07170500	1.19609900	0.72413400
C	2.06830600	-1.19413700	0.72435300	H	-1.17951800	2.04298500	2.49053600
C	1.20241100	-2.08263700	1.39980000	C	2.07170500	1.19609900	-0.72413400
H	1.18414600	-2.05100000	2.48691900	C	2.40411100	0.00000000	1.40405100
C	0.00000000	-2.38827400	0.72435300	C	2.40411100	0.00000000	-1.40405100
C	0.00000000	-2.38827400	-0.72435300	C	2.07170500	-1.19609900	0.72413400
C	1.20241100	-2.08263700	-1.39980000	H	2.35903600	0.00000000	2.49053600
H	2.36829100	0.00000000	2.48691900	H	1.17951800	2.04298500	-2.49053600
H	1.18414600	2.05100000	2.48691900	C	2.07170500	-1.19609900	-0.72413400
H	1.18414600	2.05100000	-2.48691900	H	2.35903600	0.00000000	-2.49053600
H	2.36829100	0.00000000	-2.48691900	C	-2.07170500	1.19609900	-0.72413400
H	1.18414600	-2.05100000	-2.48691900	H	-1.17951800	2.04298500	-2.49053600
C	-1.20241100	-2.08263700	1.39980000	C	1.20205500	-2.08202100	1.40405100
C	-2.06830600	-1.19413700	0.72435300	C	1.20205500	-2.08202100	-1.40405100
C	-2.06830600	-1.19413700	-0.72435300	C	0.00000000	-2.39219900	-0.72413400
C	-1.20241100	-2.08263700	-1.39980000	H	1.17951800	-2.04298500	-2.49053600
C	-2.40482300	0.00000000	1.39980000	H	1.17951800	-2.04298500	2.49053600
C	-2.40482300	0.00000000	-1.39980000	C	0.00000000	-2.39219900	0.72413400
C	-2.06830600	1.19413700	-0.72435300	C	-1.20205500	-2.08202100	1.40405100
C	-2.06830600	1.19413700	0.72435300	C	-1.20205500	-2.08202100	-1.40405100
C	-1.20241100	2.08263700	1.39980000	C	-2.07170500	-1.19609900	0.72413400
H	-1.18414600	2.05100000	2.48691900	H	-1.17951800	-2.04298500	2.49053600
C	-1.20241100	2.08263700	-1.39980000	C	-2.07170500	-1.19609900	-0.72413400
H	-2.36829100	0.00000000	2.48691900	H	-1.17951800	-2.04298500	-2.49053600
H	-2.36829100	0.00000000	-2.48691900	C	-2.40411100	0.00000000	1.40405100
H	-1.18414600	2.05100000	-2.48691900	C	-2.40411100	0.00000000	-1.40405100
H	-1.18414600	-2.05100000	2.48691900	H	-2.35903600	0.00000000	2.49053600
H	-1.18414600	-2.05100000	-2.48691900	H	-2.35903600	0.00000000	-2.49053600
**Cyc7_R**				**Cyc7_U**			
C	0.00000000	0.73347000	2.76892500	C	0.00000000	2.76892500	0.73347000
C	-1.20291100	1.41382900	2.49799600	C	-1.20296000	2.49797200	1.41382900
C	0.00000000	-0.73347000	2.76892500	C	0.00000000	2.76892500	-0.73347000
C	1.20291100	1.41382900	2.49799600	C	1.20296000	2.49797200	1.41382900
C	-2.16453300	0.73344800	1.72616300	C	-2.16483300	1.72639600	0.73347000
H	-1.18018500	2.49992800	2.45072700	H	-1.18020400	2.45071800	2.49992800
C	-1.20291100	-1.41382900	2.49799600	C	-1.20296000	2.49797200	-1.41382900
C	1.20291100	-1.41382900	2.49799600	C	1.20296000	2.49797200	-1.41382900
C	2.16453300	0.73344800	1.72616300	C	2.16483300	1.72639600	0.73347000
H	1.18018500	2.49992800	2.45072700	H	1.18020400	2.45071800	2.49992800
C	-2.16453300	-0.73344800	1.72616300	C	-2.16483300	1.72639600	-0.73347000
C	-2.70305200	1.41382200	0.61694500	C	-2.70302700	0.61694800	1.41382900
H	-1.18018500	-2.49992800	2.45072700	H	-1.18020400	2.45071800	-2.49992800

C	2.16453300	-0.73344800	1.72616300	C	2.16483300	1.72639600	-0.73347000
H	1.18018500	-2.49992800	2.45072700	H	1.18020400	2.45071800	-2.49992800
C	2.70305200	1.41382200	0.61694500	C	2.70302700	0.61694800	1.41382900
C	-2.70305200	-1.41382200	0.61694500	C	-2.70302700	0.61694800	-1.41382900
C	-2.69917900	0.73347300	-0.61610400	C	-2.69950200	-0.61614400	0.73347000
H	-2.65209900	2.49993700	0.60535900	H	-2.65189300	0.60527700	2.49992800
C	2.70305200	-1.41382200	0.61694500	C	2.70302700	0.61694800	-1.41382900
C	2.69917900	0.73347300	-0.61610400	C	2.69950200	-0.61614400	0.73347000
H	2.65209900	2.49993700	0.60535900	H	2.65189300	0.60527700	2.49992800
C	-2.69917900	-0.73347300	-0.61610400	C	-2.69950200	-0.61614400	-0.73347000
H	-2.65209900	-2.49993700	0.60535900	H	-2.65189300	0.60527700	-2.49992800
C	-2.16778300	1.41387700	-1.72877000	C	-2.16765900	-1.72865000	1.41382900
C	2.69917900	-0.73347300	-0.61610400	C	2.69950200	-0.61614400	-0.73347000
H	2.65209900	-2.49993700	0.60535900	H	2.65189300	0.60527700	-2.49992800
C	2.16778300	1.41387700	-1.72877000	C	2.16765900	-1.72865000	1.41382900
C	-2.16778300	-1.41387700	-1.72877000	C	-2.16765900	-1.72865000	-1.41382900
H	-2.12691900	2.49998400	-1.69618200	H	-2.12665300	-1.69594900	2.49992800
C	-1.20117900	0.73343500	-2.49424200	C	-1.20139100	-2.49471500	0.73347000
H	-2.12691900	-2.49998400	-1.69618200	H	-2.12665300	-1.69594900	-2.49992800
C	-1.20117900	-0.73343500	-2.49424200	C	-1.20139100	-2.49471500	-0.73347000
C	0.00000000	1.41381200	-2.77275900	C	0.00000000	-2.77254000	1.41382900
C	0.00000000	-1.41381200	-2.77275900	C	0.00000000	-2.77254000	-1.41382900
C	2.16778300	-1.41387700	-1.72877000	C	2.16765900	-1.72865000	-1.41382900
C	1.20117900	0.73343500	-2.49424200	C	1.20139100	-2.49471500	0.73347000
H	0.00000000	2.49993100	-2.72064600	H	0.00000000	-2.72009200	2.49992800
C	1.20117900	-0.73343500	-2.49424200	C	1.20139100	-2.49471500	-0.73347000
H	0.00000000	-2.49993100	-2.72064600	H	0.00000000	-2.72009200	-2.49992800
H	2.12691900	2.49998400	-1.69618200	H	2.12665300	-1.69594900	2.49992800
H	2.12691900	-2.49998400	-1.69618200	H	2.12665300	-1.69594900	-2.49992800

**Cyc8_R**				**Cyc8_U**			
				C	1.21191400	2.92581900	1.40712000
C	-1.21146400	-2.92473400	1.40469100	C	0.00000000	3.15874500	0.72720400
C	0.00000000	-3.15394200	0.72830500	H	1.19762800	2.89133000	2.49400000
H	-1.19937200	-2.89554100	2.49199100	C	2.23357000	2.23357000	0.72720400
C	-2.23017300	-2.23017300	0.72830500	C	0.00000000	3.15874500	-0.72720400
C	0.00000000	-3.15394200	-0.72830500	C	-1.21191400	2.92581900	1.40712000
C	1.21146400	-2.92473400	1.40469100	C	2.23357000	2.23357000	-0.72720400
C	-2.23017300	-2.23017300	-0.72830500	C	2.92581900	1.21191400	1.40712000
C	-2.92473400	-1.21146400	1.40469100	C	1.21191400	2.92581900	-1.40712000
C	-1.21146400	-2.92473400	-1.40469100	C	-1.21191400	2.92581900	-1.40712000
C	1.21146400	-2.92473400	-1.40469100	C	-2.23357000	2.23357000	0.72720400
C	2.23017300	-2.23017300	0.72830500	H	-1.19762800	2.89133000	2.49400000
H	1.19937200	-2.89554100	2.49199100	C	2.92581900	1.21191400	-1.40712000
C	-2.92473400	-1.21146400	-1.40469100	C	3.15874500	0.00000000	0.72720400
C	-3.15394200	0.00000000	0.72830500	H	2.89133000	1.19762800	2.49400000
H	-2.89554100	-1.19937200	2.49199100	H	1.19762800	2.89133000	-2.49400000
H	-1.19937200	-2.89554100	-2.49199100	C	-2.23357000	2.23357000	-0.72720400
C	2.23017300	-2.23017300	-0.72830500	H	-1.19762800	2.89133000	-2.49400000
H	1.19937200	-2.89554100	-2.49199100	C	-2.92581900	1.21191400	1.40712000
C	2.92473400	-1.21146400	1.40469100	C	3.15874500	0.00000000	-0.72720400
C	-3.15394200	0.00000000	-0.72830500	H	2.89133000	1.19762800	-2.49400000
H	-2.89554100	-1.19937200	-2.49199100	C	2.92581900	-1.21191400	1.40712000
C	-2.92473400	1.21146400	1.40469100	C	-2.92581900	1.21191400	-1.40712000
C	2.92473400	-1.21146400	-1.40469100	H	-2.89133000	1.19762800	2.49400000
H	2.89554100	-1.19937200	2.49199100	C	-3.15874500	0.00000000	0.72720400
C	3.15394200	0.00000000	0.72830500	C	2.92581900	-1.21191400	-1.40712000
C	-2.92473400	1.21146400	-1.40469100	C	2.23357000	-2.23357000	0.72720400
C	-2.23017300	2.23017300	0.72830500	H	2.89133000	-1.19762800	2.49400000

H	-2.89554100	1.19937200	2.49199100	H	-2.89133000	1.19762800	-2.49400000
H	2.89554100	-1.19937200	-2.49199100	C	-3.15874500	0.00000000	-0.72720400
C	3.15394200	0.00000000	-0.72830500	C	-2.92581900	-1.21191400	1.40712000
C	2.92473400	1.21146400	1.40469100	C	-2.92581900	-1.21191400	-1.40712000
C	2.92473400	1.21146400	-1.40469100	C	2.23357000	-2.23357000	-0.72720400
C	-2.23017300	2.23017300	-0.72830500	H	2.89133000	-1.19762800	-2.49400000
H	-2.89554100	1.19937200	-2.49199100	C	-2.23357000	-2.23357000	0.72720400
C	2.23017300	2.23017300	0.72830500	H	-2.89133000	-1.19762800	2.49400000
H	2.89554100	1.19937200	2.49199100	C	-2.23357000	-2.23357000	-0.72720400
C	2.23017300	2.23017300	-0.72830500	H	-2.89133000	-1.19762800	-2.49400000
H	2.89554100	1.19937200	-2.49199100	C	-1.21191400	-2.92581900	-1.40712000
C	1.21146400	2.92473400	-1.40469100	C	-1.21191400	-2.92581900	1.40712000
C	1.21146400	2.92473400	1.40469100	C	0.00000000	-3.15874500	0.72720400
C	0.00000000	3.15394200	0.72830500	H	-1.19762800	-2.89133000	2.49400000
H	1.19937200	2.89554100	2.49199100	C	0.00000000	-3.15874500	-0.72720400
C	0.00000000	3.15394200	-0.72830500	H	-1.19762800	-2.89133000	-2.49400000
H	1.19937200	2.89554100	-2.49199100	C	1.21191400	-2.92581900	1.40712000
C	-1.21146400	2.92473400	1.40469100	C	1.21191400	-2.92581900	-1.40712000
C	-1.21146400	2.92473400	-1.40469100	H	1.19762800	-2.89133000	2.49400000
H	-1.19937200	2.89554100	2.49199100	H	1.19762800	-2.89133000	-2.49400000
H	-1.19937200	2.89554100	-2.49199100				
**Cyc9_R**				**Cyc9_U**			
C	-0.61478500	3.48537800	0.73234200	C	0.00000000	0.72640100	-3.54956600
C	-1.77181900	3.06791800	1.41289100	C	1.21619800	1.40756400	-3.34234700
C	-0.61478500	3.48537800	-0.73234200	C	2.28091100	0.72642100	-2.71907400
C	0.61523900	3.48823900	1.41285100	C	2.28091100	-0.72642100	-2.71907400
C	-2.71254300	2.27546100	0.73236500	C	1.21619800	-1.40756400	-3.34234700
H	-1.75024900	3.03048300	2.49951800	C	0.00000000	-0.72640100	-3.54956600
C	-1.77181900	3.06791800	-1.41289100	C	3.07884100	1.40760000	-1.77815100
C	0.61523900	3.48823900	-1.41285100	C	3.07884100	-1.40760000	-1.77815100
C	1.77024000	3.06514900	0.73232900	C	3.49364200	-0.72640000	-0.61626100
H	0.60775200	3.44565200	2.49947700	C	3.49364200	0.72640000	-0.61626100
C	-2.71254300	2.27546100	-0.73236500	C	3.50061600	1.40758400	0.61744000
C	-3.33045200	1.21187100	1.41286700	H	3.46572300	2.49447600	0.61130600
H	-1.75024900	3.03048300	-2.49951800	C	3.50061600	-1.40758400	0.61744000
C	1.77024000	3.06514900	-0.73232900	H	3.04806900	2.49448800	-1.76040800
H	0.60775200	3.44565200	-2.49947700	H	1.20406700	2.49445500	-3.30901900
C	2.71480000	2.27734200	1.41287900	H	1.20406700	-2.49445500	-3.30901900
C	-3.33045200	1.21187100	-1.41286700	H	3.04806900	-2.49448800	-1.76040800
C	-3.54108600	0.00000000	0.73234700	H	3.46572300	-2.49447600	0.61130600
H	-3.28996000	1.19714500	2.49950100	C	3.07247800	0.72638900	1.77448800
C	-3.54108600	0.00000000	-0.73234700	C	2.28548600	1.40757800	2.72452500
H	-3.28996000	1.19714500	-2.49950100	C	1.21376100	0.72642000	3.33573000
C	-3.33045200	-1.21187100	1.41286700	C	1.21376100	-0.72642000	3.33573000
C	-3.33045200	-1.21187100	-1.41286700	C	2.28548600	-1.40757800	2.72452500
C	2.71480000	2.27734200	-1.41287900	C	3.07247800	-0.72638900	1.77448800
H	-3.28996000	-1.19714500	2.49950100	C	0.00000000	1.40758500	3.55693100
C	-2.71254300	-2.27546100	0.73236500	C	0.00000000	-1.40758500	3.55693100
H	-3.28996000	-1.19714500	-2.49950100	C	-1.21376100	-0.72642000	3.33573000
C	-2.71254300	-2.27546100	-0.73236500	C	-1.21376100	0.72642000	3.33573000
C	-1.77181900	-3.06791800	-1.41289100	C	-2.28548600	1.40757800	2.72452500
C	-1.77181900	-3.06791800	1.41289100	H	-2.26270900	2.49446900	2.69732700
C	-0.61478500	-3.48537800	0.73234200	C	-2.28548600	-1.40757800	2.72452500
H	-1.75024900	-3.03048300	2.49951800	H	0.00000000	2.49446500	3.52119300
C	-0.61478500	-3.48537800	-0.73234200	H	2.26270900	2.49446900	2.69732700
H	-1.75024900	-3.03048300	-2.49951800	H	2.26270900	-2.49446900	2.69732700
C	0.61523900	-3.48823900	1.41285100	H	0.00000000	-2.49446500	3.52119300
C	0.61523900	-3.48823900	-1.41285100	H	-2.26270900	-2.49446900	2.69732700

C	1.77024000	-3.06514900	0.73232900	C	-3.07247800	0.72638900	1.77448800
H	0.60775200	-3.44565200	2.49947700	C	-3.50061600	1.40758400	0.61744000
C	1.77024000	-3.06514900	-0.73232900	C	-3.49364200	0.72640000	-0.61626100
H	0.60775200	-3.44565200	-2.49947700	C	-3.49364200	-0.72640000	-0.61626100
C	2.71480000	-2.27734200	1.41287900	C	-3.50061600	-1.40758400	0.61744000
C	2.71480000	-2.27734200	-1.41287900	C	-3.07247800	-0.72638900	1.77448800
H	2.68180000	-2.24968800	2.49951400	C	-3.07884100	1.40760000	-1.77815100
C	3.32775500	-1.21085700	0.73235700	C	-3.07884100	-1.40760000	-1.77815100
H	2.68180000	-2.24968800	-2.49951400	C	-2.28091100	-0.72642100	-2.71907400
C	3.32775500	-1.21085700	-0.73235700	C	-2.28091100	0.72642100	-2.71907400
C	3.54429300	0.00000000	-1.41287800	C	-1.21619800	1.40756400	-3.34234700
C	3.54429300	0.00000000	1.41287800	H	-1.20406700	2.49445500	-3.30901900
C	3.32775500	1.21085700	0.73235700	C	-1.21619800	-1.40756400	-3.34234700
H	3.50085600	0.00000000	2.49949700	H	-3.04806900	2.49448800	-1.76040800
C	3.32775500	1.21085700	-0.73235700	H	-3.46572300	2.49447600	0.61130600
H	3.50085600	0.00000000	-2.49949700	H	-3.46572300	-2.49447600	0.61130600
H	2.68180000	2.24968800	2.49951400	H	-3.04806900	-2.49448800	-1.76040800
H	2.68180000	2.24968800	-2.49951400	H	-1.20406700	-2.49445500	-3.30901900
**Cyc10_R**				**Cyc10_U**			
C	0.00000000	3.92631700	0.73017000	C	0.00000000	3.93271300	0.72804500
C	0.00000000	3.92631700	-0.73017000	C	0.00000000	3.93271300	-0.72804500
C	-1.21589300	3.74213300	1.40685200	C	-1.21729300	3.74644300	1.40785000
C	1.21589300	3.74213300	1.40685200	C	1.21729300	3.74644300	1.40785000
C	-1.21589300	3.74213300	-1.40685200	C	-1.21729300	3.74644300	-1.40785000
C	1.21589300	3.74213300	-1.40685200	C	1.21729300	3.74644300	-1.40785000
H	-1.20730500	3.71570300	2.49428400	H	-1.20761200	3.71664900	2.49497400
C	-2.30783100	3.17645700	0.73017000	C	-2.31159100	3.18163200	0.72804500
H	-1.20730500	3.71570300	-2.49428400	C	2.31159100	3.18163200	0.72804500
C	-2.30783100	3.17645700	-0.73017000	H	1.20761200	3.71664900	2.49497400
C	-3.18324800	2.31276500	1.40685200	H	-1.20761200	3.71664900	-2.49497400
C	-3.73415000	1.21329900	0.73017000	C	-2.31159100	3.18163200	-0.72804500
C	-3.73415000	1.21329900	-0.73017000	C	2.31159100	3.18163200	-0.72804500
C	-3.93471100	0.00000000	1.40685200	H	1.20761200	3.71664900	-2.49497400
C	-3.93471100	0.00000000	-1.40685200	C	-3.18691400	2.31542900	1.40785000
C	-3.18324800	2.31276500	-1.40685200	C	-3.18691400	2.31542900	-1.40785000
C	-3.73415000	-1.21329900	-0.73017000	C	-3.74023200	1.21527500	0.72804500
C	-3.18324800	-2.31276500	-1.40685200	H	-3.16157000	2.29701500	2.49497400
C	-2.30783100	-3.17645700	-0.73017000	C	-3.74023200	1.21527500	-0.72804500
C	-2.30783100	-3.17645700	0.73017000	C	-3.93924300	0.00000000	1.40785000
C	-1.21589300	-3.74213300	1.40685200	C	-3.93924300	0.00000000	-1.40785000
C	0.00000000	-3.92631700	0.73017000	C	-3.74023200	-1.21527500	0.72804500
H	-1.20730500	-3.71570300	2.49428400	H	-3.90791600	0.00000000	2.49497400
C	-3.18324800	-2.31276500	1.40685200	C	-3.74023200	-1.21527500	-0.72804500
C	-3.73415000	-1.21329900	0.73017000	H	-3.90791600	0.00000000	-2.49497400
H	-3.16076600	-2.29643100	2.49428400	H	-3.16157000	2.29701500	-2.49497400
C	-1.21589300	-3.74213300	-1.40685200	C	-3.18691400	-2.31542900	-1.40785000
H	-3.16076600	-2.29643100	-2.49428400	C	-2.31159100	-3.18163200	-0.72804500
C	0.00000000	-3.92631700	-0.73017000	H	-3.16157000	-2.29701500	-2.49497400
C	1.21589300	-3.74213300	1.40685200	C	-2.31159100	-3.18163200	0.72804500
H	-1.20730500	-3.71570300	-2.49428400	C	-1.21729300	-3.74644300	-1.40785000
H	-3.90692100	0.00000000	-2.49428400	C	-1.21729300	-3.74644300	1.40785000
H	-3.90692100	0.00000000	2.49428400	C	-3.18691400	-2.31542900	1.40785000
C	1.21589300	-3.74213300	-1.40685200	C	0.00000000	-3.93271300	0.72804500
H	-3.16076600	2.29643100	2.49428400	H	-1.20761200	-3.71664900	2.49497400
H	-3.16076600	2.29643100	-2.49428400	C	0.00000000	-3.93271300	-0.72804500
H	1.20730500	-3.71570300	2.49428400	C	1.21729300	-3.74644300	1.40785000
C	2.30783100	-3.17645700	0.73017000	H	-3.16157000	-2.29701500	2.49497400
H	1.20730500	-3.71570300	-2.49428400	H	-1.20761200	-3.71664900	-2.49497400

C	2.30783100	-3.17645700	-0.73017000	C	1.21729300	-3.74644300	-1.40785000
C	2.30783100	3.17645700	0.73017000	H	1.20761200	-3.71664900	2.49497400
H	1.20730500	3.71570300	2.49428400	C	2.31159100	-3.18163200	0.72804500
C	3.18324800	2.31276500	1.40685200	H	1.20761200	-3.71664900	-2.49497400
C	2.30783100	3.17645700	-0.73017000	C	2.31159100	-3.18163200	-0.72804500
C	3.73415000	1.21329900	0.73017000	C	3.18691400	-2.31542900	1.40785000
H	3.16076600	2.29643100	2.49428400	C	3.18691400	-2.31542900	-1.40785000
C	3.18324800	2.31276500	-1.40685200	C	3.18691400	2.31542900	1.40785000
C	3.73415000	1.21329900	-0.73017000	C	3.74023200	1.21527500	0.72804500
H	3.16076600	2.29643100	-2.49428400	H	3.16157000	2.29701500	2.49497400
C	3.93471100	0.00000000	-1.40685200	C	3.18691400	2.31542900	-1.40785000
C	3.73415000	-1.21329900	-0.73017000	C	3.74023200	1.21527500	-0.72804500
C	3.73415000	-1.21329900	0.73017000	C	3.93924300	0.00000000	1.40785000
C	3.93471100	0.00000000	1.40685200	H	3.16157000	2.29701500	-2.49497400
C	3.18324800	-2.31276500	1.40685200	C	3.93924300	0.00000000	-1.40785000
C	3.18324800	-2.31276500	-1.40685200	C	3.74023200	-1.21527500	-0.72804500
H	1.20730500	3.71570300	-2.49428400	H	3.90791600	0.00000000	-2.49497400
H	3.90692100	0.00000000	-2.49428400	C	3.74023200	-1.21527500	0.72804500
H	3.90692100	0.00000000	2.49428400	H	3.90791600	0.00000000	2.49497400
H	3.16076600	-2.29643100	2.49428400	H	3.16157000	-2.29701500	2.49497400
H	3.16076600	-2.29643100	-2.49428400	H	3.16157000	-2.29701500	-2.49497400
	**Cyc11_R**				**Cyc11_U**		
C	4.27310000	0.73191900	0.61406200	C	-0.61535300	4.28207100	-0.72685200
C	4.27310000	-0.73191900	0.61406200	C	-0.61535300	4.28207100	0.72685200
C	4.27568800	1.41231000	-0.61450800	C	0.61636600	4.28844900	-1.40713300
C	3.92888600	1.41232000	1.79345200	C	-1.79880000	3.94053300	-1.40717500
C	4.27568800	-1.41231000	-0.61450800	C	0.61636600	4.28844900	1.40713300
C	3.92888600	-1.41232000	1.79345200	C	-1.79880000	3.94053300	1.40717500
C	3.92680100	-0.73193800	-1.79255900	C	1.79636900	3.93508400	0.72686700
C	3.26388300	-1.41234700	-2.82696200	C	2.83543400	3.27356100	1.40725000
C	2.33317700	-0.73192200	-3.62881400	C	3.63660700	2.33808500	0.72682900
C	2.33317700	0.73192200	-3.62881400	C	3.63660700	2.33808500	-0.72682900
C	1.21645300	1.41233500	-4.14092200	C	4.15360300	1.22010300	-1.40732600
C	0.00000000	0.73192300	-4.31296200	C	4.32234800	0.00000000	-0.72680900
H	1.20631900	2.49927100	-4.10628600	H	4.12645400	1.21185200	-2.49448200
C	3.26388300	1.41234700	-2.82696200	C	2.83543400	3.27356100	-1.40725000
C	3.92680100	0.73193800	-1.79255900	C	1.79636900	3.93508400	-0.72686700
H	3.23653000	2.49928300	-2.80327300	H	2.81710900	3.25166000	-2.49441500
C	1.21645300	-1.41233500	-4.14092200	C	4.15360300	1.22010300	1.40732600
H	3.23653000	-2.49928300	-2.80327300	H	2.81710900	3.25166000	2.49441500
C	0.00000000	-0.73192300	-4.31296200	C	4.32234800	0.00000000	0.72680900
C	-1.21645300	1.41233500	-4.14092200	C	4.15360300	-1.22010200	-1.40732600
H	1.20631900	-2.49927100	-4.10628600	H	4.12645400	1.21185200	2.49448200
H	4.23971300	-2.49924200	-0.60933700	H	0.61243200	4.26020300	2.49429300
H	4.23971300	2.49924200	-0.60933700	H	0.61243200	4.26020300	-2.49429300
C	-1.21645300	-1.41233500	-4.14092200	C	4.15360300	-1.22010200	1.40732600
C	-2.33317700	0.73192200	-3.62881400	C	3.63660700	-2.33808400	-0.72682900
C	-3.26388300	1.41234700	-2.82696200	C	2.83543500	-3.27356100	-1.40725000
C	-3.92680100	0.73193800	-1.79255900	C	1.79637000	-3.93508400	-0.72686700
C	-4.27568800	1.41231000	-0.61450800	C	0.61636700	-4.28844900	-1.40713300
C	-4.27310000	0.73191900	0.61406200	C	-0.61535300	-4.28207100	-0.72685200
C	-3.92888600	1.41232000	1.79345200	C	-1.79880000	-3.94053300	-1.40717500
C	-3.26183400	0.73193200	2.82518100	C	-2.83118700	-3.26862800	-0.72685600
C	-3.26183400	-0.73193200	2.82518100	C	-2.83118700	-3.26862800	0.72685600
C	-2.33450700	1.41232800	3.63101000	C	-3.64197400	-2.34143500	-1.40727900
C	-3.92888600	-1.41232000	1.79345200	C	-1.79880000	-3.94053300	1.40717500
C	-2.33450700	-1.41232800	3.63101000	C	-3.64197400	-2.34143500	1.40727900
H	-3.89603500	2.49926100	1.77844000	H	-1.78731900	-3.91458300	-2.49434400

C	-4.27310000	-0.73191900	0.61406200	C	-0.61535300	-4.28207100	0.72685200
H	-4.23971300	2.49924200	-0.60933700	H	0.61243300	-4.26020300	-2.49429300
C	-4.27568800	-1.41231000	-0.61450800	C	0.61636700	-4.28844900	1.40713300
C	-3.92680100	-0.73193800	-1.79255900	C	1.79637000	-3.93508400	0.72686700
H	-4.23971300	-2.49924200	-0.60933700	H	0.61243300	-4.26020300	2.49429300
C	-3.26388300	-1.41234700	-2.82696200	C	2.83543500	-3.27356100	1.40725000
H	-3.89603500	-2.49926100	1.77844000	H	-1.78731900	-3.91458300	2.49434400
C	-2.33317700	-0.73192200	-3.62881400	C	3.63660700	-2.33808400	0.72682900
H	-3.23653000	-2.49928300	-2.80327300	H	2.81710900	-3.25166000	2.49441500
H	-3.23653000	2.49928300	-2.80327300	H	2.81710900	-3.25166000	-2.49441500
H	-1.20631900	-2.49927100	-4.10628600	H	4.12645400	-1.21185100	2.49448200
H	-1.20631900	2.49927100	-4.10628600	H	4.12645400	-1.21185100	-2.49448200
C	-1.21576200	-0.73191000	4.13870000	C	-4.14774900	-1.21832800	0.72679400
H	-2.31496400	-2.49926000	3.60052800	H	-3.61815000	-2.32557800	2.49443400
C	0.00000000	-1.41232200	4.31574100	C	-4.32906700	0.00000000	1.40732800
C	-1.21576200	0.73191000	4.13870000	C	-4.14774900	-1.21832800	-0.72679400
C	1.21576200	-0.73191000	4.13870000	C	-4.14774900	1.21832700	0.72679400
H	0.00000000	-2.49925500	4.27949200	H	-4.30053200	0.00000000	2.49447700
C	0.00000000	1.41232200	4.31574100	C	-4.32906700	0.00000000	-1.40732800
C	1.21576200	0.73191000	4.13870000	C	-4.14774900	1.21832700	-0.72679400
H	0.00000000	2.49925500	4.27949200	H	-4.30053200	0.00000000	-2.49447700
C	2.33450700	1.41232800	3.63101000	C	-3.64197400	2.34143400	-1.40727900
C	2.33450700	-1.41232800	3.63101000	C	-3.64197400	2.34143400	1.40727900
H	-2.31496400	2.49926000	3.60052800	H	-3.61815000	-2.32557800	-2.49443400
C	3.26183400	0.73193200	2.82518100	C	-2.83118800	3.26862800	-0.72685600
H	2.31496400	2.49926000	3.60052800	H	-3.61815100	2.32557700	-2.49443400
C	3.26183400	-0.73193200	2.82518100	C	-2.83118800	3.26862800	0.72685600
H	2.31496400	-2.49926000	3.60052800	H	-3.61815100	2.32557700	2.49443400
H	3.89603500	2.49926100	1.77844000	H	-1.78732000	3.91458300	-2.49434400
H	3.89603500	-2.49926100	1.77844000	H	-1.78732000	3.91458300	2.49434400
**Cyc12_R**				**Cyc12_U**			
C	-0.73126900	3.32511200	-3.32486600	C	0.00000000	4.71036500	0.72804800
C	0.73124800	3.32487000	-3.32512700	C	0.00000000	4.71036500	-0.72804800
C	-1.40812500	0.01118100	4.70845300	C	1.22054500	4.55513800	1.40779800
C	-0.73123900	-1.21719400	4.54183500	C	-1.22054500	4.55513800	1.40779800
C	-1.40815600	-2.34454500	4.08330600	C	1.22054500	4.55513800	-1.40779800
C	0.73123900	-1.21686700	4.54190300	C	-1.22054500	4.55513800	-1.40779800
C	1.40812600	-2.36407100	4.07224700	C	2.35518300	4.07929600	0.72804800
C	0.73123700	-3.32486100	3.32512500	H	1.21366900	4.52947500	2.49507300
C	-0.73127900	-3.32510100	3.32485900	C	-2.35518300	4.07929600	0.72804800
C	-0.73123900	1.21686700	4.54190300	H	-1.21366900	4.52947500	2.49507300
C	1.40812500	-0.01118100	4.70845300	C	2.35518300	4.07929600	-0.72804800
H	2.49564800	-2.35167800	4.05072900	H	1.21366900	4.52947500	-2.49507300
C	1.40812200	-4.08330700	2.34457300	C	-2.35518300	4.07929600	-0.72804800
C	-1.40816400	-4.07219700	2.36404500	H	-1.21366900	4.52947500	-2.49507300
H	2.49564600	-4.06174600	2.33208900	C	3.33459200	3.33459200	1.40779800
H	-2.49568500	-4.05066500	2.35163300	C	4.07929600	2.35518300	0.72804800
C	0.73123900	1.21719400	4.54183500	H	3.31580600	3.31580600	2.49507300
H	-2.49564900	0.01122500	4.68354700	C	3.33459200	3.33459200	-1.40779800
H	-2.49568000	-2.33204400	4.06173600	C	4.07929600	2.35518300	-0.72804800
H	2.49564900	-0.01122500	4.68354700	C	4.55513800	1.22054500	1.40779800
C	-0.73125900	-4.54186200	1.21685200	H	3.31580600	3.31580600	-2.49507300
C	0.73122000	-4.54181300	1.21720400	C	4.55513800	1.22054500	-1.40779800
C	-1.40815700	4.07220900	-2.36405600	C	4.71036500	0.00000000	-0.72804800
C	-0.73125800	4.54187000	-1.21685800	H	4.52947500	1.21366900	-2.49507300
C	-1.40812700	4.70842000	-0.01116200	C	4.71036500	0.00000000	0.72804800
C	0.73122100	4.54182300	-1.21720800	H	4.52947500	1.21366900	2.49507300
C	1.40812500	4.70843200	0.01115700	C	4.55513800	-1.22054500	-1.40779800

C	0.73125900	4.54186200	1.21685200	C	4.55513800	-1.22054500	1.40779800
C	-0.73122000	4.54181300	1.21720400	C	4.07929600	-2.35518300	0.72804800
C	1.40812600	4.08332000	-2.34457700	H	4.52947500	-1.21366900	2.49507300
C	1.40816400	4.07219700	2.36404500	C	3.33459200	-3.33459200	1.40779800
C	-1.40812200	4.08330700	2.34457300	C	4.07929600	-2.35518300	-0.72804800
H	-2.49567800	4.05067700	-2.35164800	C	2.35518300	-4.07929600	0.72804800
H	2.49565000	4.06176300	-2.33209200	H	3.31580600	-3.31580600	2.49507300
C	-0.73123700	3.32486100	3.32512500	C	1.22054500	-4.55513800	1.40779800
C	0.73127900	3.32510100	3.32485900	C	2.35518300	-4.07929600	-0.72804800
C	1.40815600	2.34454500	4.08330600	C	0.00000000	-4.71036500	0.72804800
C	-1.40812600	2.36407100	4.07224700	H	1.21366900	-4.52947500	2.49507300
H	2.49564900	4.68352400	0.01118300	C	1.22054500	-4.55513800	-1.40779800
H	-2.49565000	4.68350500	-0.01118900	C	3.33459200	-3.33459200	-1.40779800
H	-2.49564600	4.06174600	2.33208900	C	0.00000000	-4.71036500	-0.72804800
H	2.49568500	4.05066500	2.35163300	C	-1.22054500	-4.55513800	1.40779800
H	2.49568000	2.33204400	4.06173600	H	1.21366900	-4.52947500	-2.49507300
H	-2.49564800	2.35167800	4.05072900	H	3.31580600	-3.31580600	-2.49507300
C	-1.40813700	-2.36406700	-4.07223100	C	-1.22054500	-4.55513800	-1.40779800
C	-0.73124600	-1.21686300	-4.54188400	H	-1.21366900	-4.52947500	-2.49507300
C	-1.40812600	-0.01117400	-4.70843500	C	-2.35518300	-4.07929600	-0.72804800
C	0.73123300	-1.21720000	-4.54183200	H	-1.21366900	-4.52947500	2.49507300
C	1.40812600	0.01117400	-4.70843500	C	-2.35518300	-4.07929600	0.72804800
C	0.73124600	1.21686300	-4.54188400	C	-3.33459200	-3.33459200	-1.40779800
C	-0.73123300	1.21720000	-4.54183200	C	-3.33459200	-3.33459200	1.40779800
C	-0.73124800	-3.32487000	-3.32512700	H	4.52947500	-1.21366900	-2.49507300
C	1.40814500	-2.34456000	-4.08332100	C	-4.07929600	-2.35518300	0.72804800
H	2.49564900	0.01121100	-4.68353600	H	-3.31580600	-3.31580600	2.49507300
C	1.40813700	2.36406700	-4.07223100	C	-4.07929600	-2.35518300	-0.72804800
C	-1.40814500	2.34456000	-4.08332100	H	-3.31580600	-3.31580600	-2.49507300
H	2.49565900	2.35167400	-4.05071700	C	-3.33459200	3.33459200	-1.40779800
H	-2.49566900	2.33205900	-4.06174600	C	-4.07929600	2.35518300	-0.72804800
C	0.73126900	-3.32511200	-3.32486600	H	-3.31580600	3.31580600	-2.49507300
H	-2.49565900	-2.35167400	-4.05071700	C	-4.07929600	2.35518300	0.72804800
H	-2.49564900	-0.01121100	-4.68353600	C	-4.55513800	1.22054500	-1.40779800
H	2.49566900	-2.33205900	-4.06174600	C	-4.55513800	1.22054500	1.40779800
C	1.40812700	-4.70842000	-0.01116200	C	-3.33459200	3.33459200	1.40779800
C	-1.40812500	-4.70843200	0.01115700	C	-4.71036500	0.00000000	0.72804800
C	-0.73122100	-4.54182300	-1.21720800	H	-4.52947500	1.21366900	2.49507300
C	0.73125800	-4.54187000	-1.21685800	C	-4.71036500	0.00000000	-0.72804800
C	1.40815700	-4.07220900	-2.36405600	C	-4.55513800	-1.22054500	1.40779800
C	-1.40812600	-4.08332000	-2.34457700	H	-3.31580600	3.31580600	2.49507300
H	-2.49564900	-4.68352400	0.01118300	H	-4.52947500	1.21366900	-2.49507300
H	2.49565000	-4.68350500	-0.01118900	C	-4.55513800	-1.22054500	-1.40779800
H	2.49567800	-4.05067700	-2.35164800	H	-4.52947500	-1.21366900	2.49507300
H	-2.49565000	-4.06176300	-2.33209200	H	-4.52947500	-1.21366900	-2.49507300

**Cyc13_R**				**Cyc13_U**			
C	2.63777700	-4.35454100	-0.73179000	C	4.18634200	2.91535400	0.72730700
C	2.63774600	-4.35450600	0.73174700	C	4.18634200	2.91535400	-0.72730700
C	4.00713000	3.14469900	-1.41205500	C	3.37033400	3.83621500	1.40731300
C	3.13662800	4.01037300	-0.73169600	C	4.76804500	1.83108100	1.40732800
C	2.08645300	4.64626200	-1.41202100	C	3.37033400	3.83621500	-1.40731300
C	3.13665100	4.01035700	0.73185000	C	4.76804500	1.83108100	-1.40732800
C	2.08649300	4.64619600	1.41222200	C	2.35130500	4.52573300	0.72729300
C	0.91342400	5.00825500	0.73189200	H	3.35430300	3.81828400	2.49462600
C	0.91341700	5.00830500	-0.73165400	C	5.06267900	0.63646400	0.72735100
C	4.64143800	2.09358100	-0.73174000	H	4.74540400	1.82278800	2.49464100
C	4.00721300	3.14467700	1.41215300	C	2.35130500	4.52573300	-0.72729300
H	2.07382900	4.61799100	2.49933000	H	3.35430300	3.81828400	-2.49462600

C	-0.31190000	5.08350100	1.41223800	C	5.06267900	0.63646400	-0.72735100
C	-0.31192700	5.08363300	-1.41198000	H	4.74540400	1.82278800	-2.49464100
H	-0.30998400	5.05253800	2.49934500	C	1.20089100	4.96204200	1.40730600
H	-0.31002500	5.05276400	-2.49908800	C	5.07341700	-0.59389500	1.40731200
C	4.64147800	2.09358300	0.73180000	C	1.20089100	4.96204200	-1.40730600
H	3.98271900	3.12556400	-2.49915900	C	5.07341700	-0.59389500	-1.40731200
H	2.07377800	4.61808800	-2.49912900	C	-0.02176600	5.09949100	0.72732800
H	3.98284900	3.12552800	2.49925800	H	1.19540700	4.93851600	2.49461700
C	-1.51893400	4.85921800	-0.73165500	C	4.77863200	-1.78846300	0.72737600
C	-1.51892800	4.85914400	0.73190100	H	5.04950200	-0.59083900	2.49463300
C	3.60502100	-3.59843500	-1.41211100	C	-0.02176600	5.09949100	-0.72732800
C	3.60494600	-3.59837900	1.41209500	H	1.19540700	4.93851600	-2.49461700
C	4.35970800	-2.63012600	0.73177600	C	4.77863200	-1.78846300	-0.72737600
C	4.35972500	-2.63014700	-0.73178100	H	5.04950200	-0.59083900	-2.49463300
C	4.86476000	-1.51117100	1.41210600	C	-1.24330900	4.95151600	1.40732500
C	4.86476100	-1.51117000	-1.41210900	C	4.21584400	-2.88258100	1.40728600
C	5.08289300	-0.30302100	-0.73177000	C	-1.24330900	4.95151600	-1.40732500
C	5.08291600	-0.30301500	0.73177700	C	4.21584400	-2.88258100	-1.40728600
C	5.00987700	0.92247300	1.41211300	C	-2.38999500	4.50545900	0.72736100
C	5.00979800	0.92249300	-1.41209200	H	-1.23707300	4.92821400	2.49463700
H	3.58298400	-3.57648700	2.49920100	C	-2.38999500	4.50545900	-0.72736100
H	3.58313500	-3.57657200	-2.49921900	H	-1.23707300	4.92821400	-2.49463700
H	4.83514900	-1.50198000	-2.49921600	C	-3.40296000	3.80701600	-1.40729700
H	4.83513200	-1.50200100	2.49921100	C	-3.40296000	3.80701600	1.40729700
H	4.97936100	0.91683100	2.49921800	C	-4.21116800	2.87932700	-0.72737700
H	4.97923100	0.91687700	-2.49919600	H	-3.38673000	3.78921200	-2.49461900
C	-3.13795300	-4.01210600	-1.41225600	C	-4.21116800	2.87932700	0.72737700
C	-2.08557700	-4.64427800	-0.73188500	H	-3.38673000	3.78921200	2.49461900
C	-0.91380200	-5.01055100	-1.41219400	C	-4.78368100	1.79029800	1.40732900
C	-2.08561600	-4.64430300	0.73167100	C	-5.06806400	0.59318000	0.72738200
C	-0.91388000	-5.01054400	1.41202500	H	-4.76117500	1.78179900	2.49465400
C	0.31173800	-5.08136800	0.73171200	C	-4.78368100	1.79029800	-1.40732900
C	0.31176800	-5.08138700	-0.73183500	C	-5.06806400	0.59318000	-0.72738200
C	-4.00535600	-3.14327600	-0.73191000	C	-5.06827100	-0.63718600	1.40733700
C	-3.13806800	-4.01217900	1.41200300	H	-4.76117500	1.78179900	-2.49465400
H	-0.90835500	-4.98013400	2.49913500	C	-5.06827100	-0.63718600	-1.40733700
C	1.51951400	-4.86125800	1.41207200	H	-5.04433100	-0.63449300	2.49465300
C	1.51958700	-4.86131000	-1.41214600	C	-4.76305600	-1.82917800	0.72734500
H	1.51026400	-4.83175100	2.49918000	H	-5.04433100	-0.63449300	-2.49465300
H	1.51038700	-4.83182000	-2.49925500	C	-4.76305600	-1.82917800	-0.72734500
C	-4.00541500	-3.14332800	0.73164800	C	-4.19105100	-2.91851600	1.40734700
H	-3.11888200	-3.98773400	-2.49936200	C	-4.19105100	-2.91851600	-1.40734700
H	-0.90823800	-4.98013300	-2.49930200	C	-3.36671200	-3.83200200	0.72732300
H	-3.11906900	-3.98788400	2.49911200	H	-4.17107200	-2.90498500	2.49465600
C	-2.63896500	4.35650800	-1.41200800	C	-3.36671200	-3.83200200	-0.72732300
C	-3.60345300	3.59690300	-0.73170300	H	-4.17107200	-2.90498500	-2.49465600
C	-4.36165100	2.63139100	-1.41207400	C	-2.35388900	-4.53060900	-1.40732300
C	-3.60345800	3.59683400	0.73185300	C	-2.35388900	-4.53060900	1.40732300
C	-4.36166700	2.63128500	1.41214600	C	-1.19954900	-4.95631300	-0.72730700
C	-4.86269900	1.51052400	0.73177800	H	-2.34272600	-4.50918600	-2.49463500
C	-4.86267000	1.51058400	-0.73179800	C	-1.19954900	-4.95631300	0.72730700
C	-2.63894900	4.35637500	1.41222400	H	-2.34272600	-4.50918600	2.49463500
C	-5.08510000	0.30311400	1.41207500	C	0.02185000	-5.10481400	1.40730900
C	-5.08500500	0.30320200	-1.41218500	C	0.02185000	-5.10481400	-1.40730900
H	-2.62295700	4.33006500	-2.49911500	C	3.39949700	-3.80313500	-0.72736000
H	-2.62292600	4.32985100	2.49933100	H	4.19581100	-2.86900400	-2.49460700
C	-5.00753500	-0.92201900	-0.73186400	C	3.39949700	-3.80313500	0.72736000
C	-5.00759500	-0.92208000	0.73168000	H	4.19581100	-2.86900400	2.49460700
C	-4.64342500	-2.09448500	1.41198300	C	2.39270200	-4.51042400	-1.40729800
C	-4.64329500	-2.09439400	-1.41222800	C	2.39270200	-4.51042400	1.40729800

H	-4.33511000	2.61522000	2.49925100	C	1.24196000	-4.94612500	-0.72733100
H	-4.33510300	2.61538800	-2.49918100	H	2.38107500	-4.48923200	-2.49461200
H	-5.05402800	0.30137500	-2.49929100	C	1.24196000	-4.94612500	0.72733100
H	-5.05418500	0.30122700	2.49918100	H	2.38107500	-4.48923200	2.49461200
H	-4.61520100	-2.08176800	2.49909000	H	0.02142800	-5.08067800	2.49462000
H	-4.61500100	-2.08160000	-2.49933200	H	0.02142800	-5.08067800	-2.49462000
**Cyc14_R**				**Cyc14_U**			
C	-4.38379400	-3.28535500	0.73286400	C	0.00000000	5.48917900	0.72791600
C	-4.38379400	-3.28535500	-0.73286300	C	0.00000000	5.48917900	-0.72791600
C	-5.02306000	-2.22067000	1.40782700	C	1.22257100	5.35643300	1.40755900
C	-3.55986400	-4.17424700	1.40855600	C	-1.22257100	5.35643300	1.40755900
C	-5.02306000	-2.22067000	-1.40782600	C	1.22257100	5.35643300	-1.40755900
C	-3.55986400	-4.17424700	-1.40855500	C	-1.22257100	5.35643300	-1.40755900
C	-5.37577800	-1.05460700	0.73364400	C	2.38166600	4.94558000	0.72791600
H	-5.01082900	-2.21555700	2.49562000	H	1.21751400	5.33427700	2.49494200
C	-2.52215800	-4.86255800	0.73186900	C	-2.38166600	4.94558000	0.72791600
H	-3.54734700	-4.16021300	2.49624000	H	-1.21751400	5.33427700	2.49494200
C	-5.37577800	-1.05460700	-0.73364400	C	2.38166600	4.94558000	-0.72791600
H	-5.01082900	-2.21555700	-2.49561900	H	1.21751400	5.33427700	-2.49494200
C	-2.52215800	-4.86255800	-0.73186800	C	-2.38166600	4.94558000	-0.72791600
H	-3.54734700	-4.16021300	-2.49623900	H	-1.21751400	5.33427700	-2.49494200
C	-5.49275800	0.17365000	1.40725400	C	3.42556800	4.29552600	1.40755900
C	-5.29931800	1.38636600	0.73385000	C	4.29161300	3.42244700	0.72791600
H	-5.48276900	0.17321200	2.49509900	H	3.41139800	4.27775800	2.49494200
C	-5.49275800	0.17364900	-1.40725300	C	3.42556800	4.29552600	-1.40755900
C	-5.29931800	1.38636600	-0.73385000	C	4.29161300	3.42244700	-0.72791600
C	-4.87430700	2.53366300	1.40745300	C	4.95008900	2.38383700	1.40755900
H	-5.48276800	0.17321200	-2.49509800	H	3.41139800	4.27775800	-2.49494200
C	-4.87430700	2.53366300	-1.40745300	C	4.95008900	2.38383700	-1.40755900
C	-4.16922400	3.55111200	-0.73333600	C	5.35155400	1.22145700	-0.72791600
H	-4.86459400	2.52871200	-2.49528400	H	4.92961400	2.37397700	-2.49494200
C	-4.16922400	3.55111200	0.73333500	C	5.35155400	1.22145700	0.72791600
H	-4.86459400	2.52871300	2.49528400	H	4.92961400	2.37397700	2.49494200
C	-3.29112700	4.39068500	-1.40815500	C	5.49418400	0.00000000	-1.40755900
C	-3.29112700	4.39068600	1.40815400	C	5.49418400	0.00000000	1.40755900
C	-2.21174800	5.00910000	0.73236800	C	5.35155400	-1.22145700	0.72791600
H	-3.28160700	4.37838800	2.49590300	H	5.47145800	0.00000000	2.49494200
C	-1.05873900	5.37848400	1.40878700	C	4.95008900	-2.38383700	1.40755900
C	-2.21174800	5.00910000	-0.73236900	C	5.35155400	-1.22145700	-0.72791600
C	0.18287700	5.47356900	0.73137500	C	4.29161300	-3.42244700	0.72791600
H	-1.05438800	5.35800100	2.49640600	H	4.92961400	-2.37397700	2.49494200
C	1.38180200	5.30335700	1.40909600	C	3.42556800	-4.29552600	1.40755900
C	0.18287700	5.47356900	-0.73137700	C	4.29161300	-3.42244700	-0.72791600
C	2.54110700	4.85550000	0.73057400	C	2.38166600	-4.94558000	0.72791600
H	1.37568100	5.27897300	2.49658900	H	3.41139800	-4.27775800	2.49494200
C	1.38180100	5.30335600	-1.40909700	C	3.42556800	-4.29552600	-1.40755900
C	-1.05873900	5.37848300	-1.40878800	C	4.95008900	-2.38383700	-1.40755900
C	2.54110700	4.85549900	-0.73057500	C	2.38166600	-4.94558000	-0.72791600
C	3.54906600	4.17852100	1.40918100	C	1.22257100	-5.35643300	1.40755900
H	1.37568000	5.27897200	-2.49659100	H	3.41139800	-4.27775800	-2.49494200
H	-1.05438800	5.35800000	-2.49640700	H	4.92961400	-2.37397700	-2.49494200
C	3.54906500	4.17852100	-1.40918200	C	1.22257100	-5.35643300	-1.40755900
H	3.53122400	4.15737300	-2.49658100	H	1.21751400	-5.33427700	-2.49494200
C	4.39736000	3.27649500	-0.73004100	C	0.00000000	-5.48917900	-0.72791600
H	3.53122400	4.15737400	2.49657900	H	1.21751400	-5.33427700	2.49494200
C	4.39736000	3.27649500	0.73004000	C	0.00000000	-5.48917900	0.72791600
C	5.01308700	2.22443900	-1.40920500	C	-1.22257100	-5.35643300	-1.40755900
C	5.01308700	2.22443900	1.40920400	C	-1.22257100	-5.35643300	1.40755900

H	-3.28160700	4.37838700	-2.49590300	H	5.47145800	0.00000000	-2.49494200
C	5.38467400	1.04892500	0.72981000	C	-2.38166600	-4.94558000	0.72791600
H	4.98685000	2.21279200	2.49655400	H	-1.21751400	-5.33427700	2.49494200
C	5.38467400	1.04892500	-0.72981000	C	-2.38166600	-4.94558000	-0.72791600
H	4.98685000	2.21279100	-2.49655500	H	-1.21751400	-5.33427700	-2.49494200
C	-1.39391800	-5.30104700	-1.40900900	C	-3.42556800	4.29552600	-1.40755900
C	-0.16200000	-5.47550900	-0.73091200	C	-4.29161300	3.42244700	-0.72791600
H	-1.38756000	-5.27850100	-2.49655700	H	-3.41139800	4.27775800	-2.49494200
C	-0.16200000	-5.47550900	0.73091300	C	-4.29161300	3.42244700	0.72791600
C	1.04654400	-5.37867700	-1.40918000	C	-4.95008900	2.38383700	-1.40755900
C	1.04654400	-5.37867700	1.40918100	C	-4.95008900	2.38383700	1.40755900
C	-1.39391800	-5.30104700	1.40901000	C	-3.42556800	4.29552600	1.40755900
C	2.22972100	-5.00567200	0.73027300	C	-5.35155400	1.22145700	0.72791600
H	1.04162000	-5.35260400	2.49661800	H	-4.92961400	2.37397700	2.49494200
C	2.22972100	-5.00567200	-0.73027200	C	-5.35155400	1.22145700	-0.72791600
C	3.27978200	-4.39141100	1.40917600	C	-5.49418400	0.00000000	1.40755900
H	-1.38756000	-5.27850100	2.49655900	H	-3.41139800	4.27775800	2.49494200
H	1.04162000	-5.35260500	-2.49661700	H	-4.92961400	2.37397700	-2.49494200
C	3.27978200	-4.39141100	-1.40917500	C	-5.49418400	0.00000000	-1.40755900
C	4.18153000	-3.54673400	0.72991700	C	-5.35155400	-1.22145700	0.72791600
H	3.26292000	-4.36867100	2.49654500	H	-5.47145800	0.00000000	2.49494200
C	4.18153000	-3.54673400	-0.72991600	C	-5.35155400	-1.22145700	-0.72791600
H	3.26292000	-4.36867100	-2.49654400	H	-5.47145800	0.00000000	-2.49494200
C	5.48245700	-0.17155900	1.40920600	C	-3.42556800	-4.29552600	1.40755900
C	5.30751400	-1.38616000	0.72979200	C	-4.29161300	-3.42244700	0.72791600
H	5.45343900	-0.17055800	2.49654000	H	-3.41139800	-4.27775800	2.49494200
C	5.48245700	-0.17155900	-1.40920700	C	-3.42556800	-4.29552600	-1.40755900
C	5.30751400	-1.38616000	-0.72979200	C	-4.29161300	-3.42244700	-0.72791600
C	4.86352400	-2.53325400	1.40919100	C	-4.95008900	-2.38383700	1.40755900
H	5.45343900	-0.17055800	-2.49654000	H	-3.41139800	-4.27775800	-2.49494200
C	4.86352400	-2.53325400	-1.40919000	C	-4.95008900	-2.38383700	-1.40755900
H	4.83785100	-2.51977400	-2.49653000	H	-4.92961400	-2.37397700	-2.49494200
H	4.83785100	-2.51977400	2.49653000	H	-4.92961400	-2.37397700	2.49494200
**Cyc15_R**				**Cyc15_U**			
C	-1.25085600	5.74209300	0.73222300	C	5.46094000	2.17922600	0.72766500
C	-1.24941300	5.74254600	-0.73199700	C	5.46094000	2.17922600	-0.72766500
C	5.09217400	-2.94731100	1.40896800	C	4.89228400	3.26947400	1.40733300
C	4.38793900	-3.91060600	0.73216800	C	5.79965700	0.99720500	1.40737400
C	3.45267100	-4.76306000	1.40883000	C	4.89228400	3.26947400	-1.40733300
C	4.38704600	-3.91178300	-0.73200900	C	5.79965700	0.99720500	-1.40737400
C	3.51004200	-4.72102500	-1.40876300	C	4.10198900	4.21145400	0.72765800
C	2.41614700	-5.35731600	-0.73209300	H	4.87480200	3.25779600	2.49474200
C	2.41750700	-5.35671600	0.73208800	C	5.87542900	-0.23004600	0.72765800
C	5.59990000	-1.78817200	0.73229100	H	5.77898300	0.99364700	2.49478400
C	5.12769600	-2.88572300	-1.40867900	C	4.10198900	4.21145400	-0.72765800
H	3.49801900	-4.70454900	-2.49646100	H	4.87480200	3.25779600	-2.49474200
C	1.28593500	-5.74009600	-1.40890400	C	5.87542900	-0.23004600	-0.72765800
C	1.21650100	-5.75530800	1.40874400	H	5.77898300	0.99364700	-2.49478400
H	1.28165000	-5.72010600	-2.49660300	C	3.13921800	4.97628400	1.40736800
H	1.21204200	-5.73540000	2.49644700	C	5.70378500	-1.44764900	1.40735900
C	5.59956100	-1.78969400	-0.73192900	C	3.13921800	4.97628500	-1.40736900
H	5.07434200	-2.93725700	2.49667200	C	5.70378500	-1.44764900	-1.40735900
H	3.44049100	-4.74662200	2.49653200	C	2.03415200	5.51554800	0.72765100
H	5.11011800	-2.87560900	-2.49638000	H	3.12809000	4.95864000	2.49477900
C	0.02941500	-5.87682500	0.73199700	C	5.27355200	-2.59952800	0.72766200
C	0.02785900	-5.87675700	-0.73223400	H	5.68348700	-1.44250100	2.49476900
C	2.39928100	5.37106000	1.40872300	C	2.03415200	5.51554800	-0.72765100
C	3.43117500	4.77181400	0.73194900	H	3.12809100	4.95864100	-2.49477900

C	4.37712400	3.93130900	1.40866400	C	5.27355200	-2.59952800	-0.72766200
C	3.43235900	4.77089000	-0.73224700	H	5.68348600	-1.44250100	-2.49476900
C	4.32933300	3.98381300	-1.40895100	C	0.84351000	5.82265200	-1.40733300
C	5.07698700	2.96283900	-0.73221100	C	0.84351000	5.82265200	1.40733300
C	5.07621800	2.96419100	0.73198200	C	-0.38534300	5.86602100	-0.72765500
C	2.33409300	5.39961500	-1.40889800	H	0.84050800	5.80187500	-2.49474300
C	5.57633100	1.87899000	-1.40890000	C	-0.38534300	5.86602100	0.72765500
C	5.59851400	1.81164100	1.40878100	H	0.84050800	5.80187400	2.49474300
H	2.39118900	5.35232100	2.49642700	C	-1.59801200	5.66261600	-1.40735200
H	2.32577500	5.38091000	-2.49659700	C	-1.59801200	5.66261600	1.40735200
C	5.84355200	0.64373000	0.73210800	C	-2.73830200	5.20255900	0.72766600
C	5.84378400	0.64216000	-0.73212400	H	-1.59231900	5.64243200	2.49476200
C	5.85869300	-0.55114600	-1.40874500	C	-2.73830200	5.20255900	-0.72766600
C	5.85145400	-0.62161300	1.40892500	H	-1.59231900	5.64243200	-2.49476200
H	4.31414100	3.97000300	-2.49664700	C	-3.76336800	4.52347900	1.40739900
H	4.36201700	3.91750800	2.49636600	C	-3.76336800	4.52347900	-1.40739900
H	5.57918300	1.80522500	2.49648400	C	-4.61780500	3.63923600	0.72766600
H	5.55703100	1.87257800	-2.49660200	H	-3.75000700	4.50739700	2.49481000
H	5.83853200	-0.54920800	-2.49644800	C	-4.61780400	3.63923600	-0.72766600
H	5.83117400	-0.61956300	2.49662900	H	-3.75000600	4.50739600	-2.49480900
C	-5.59412200	1.82451100	1.40879400	C	-5.27786400	2.60182800	1.40733400
C	-5.10567500	2.91331800	0.73211700	C	-5.69874500	1.44649300	0.72765100
C	-4.36816600	3.94156700	1.40885900	H	-5.25903700	2.59254900	2.49474500
C	-5.10491600	2.91465600	-0.73210300	C	-5.27786400	2.60182800	-1.40733400
C	-4.41540300	3.88864000	-1.40879800	C	-5.87981300	0.23026700	1.40736200
C	-3.47769000	4.73831700	-0.73200300	C	-5.69874500	1.44649300	-0.72765100
C	-3.47890100	4.73734500	0.73218000	H	-5.25903600	2.59254900	-2.49474500
C	-5.84944800	0.58514400	0.73204500	C	-5.79438600	-0.99637300	0.72764800
C	-5.61583500	1.75694700	-1.40886500	H	-5.85893000	0.22944500	2.49477300
H	-4.40020000	3.87510200	-2.49649700	C	-5.87981200	0.23026600	-1.40736200
C	-2.45164300	5.34762700	-1.40870300	C	-5.46511800	-2.18107000	1.40733900
C	-2.38685500	5.37664900	1.40893800	C	-5.79438600	-0.99637300	-0.72764800
H	-2.44326900	5.32906500	-2.49640200	H	-5.85893000	0.22944500	-2.49477300
H	-2.37838400	5.35801500	2.49663900	C	-4.88814400	-3.26691600	0.72766000
C	-5.84934400	0.58665000	-0.73217600	H	-5.44565100	-2.17330200	2.49474800
H	-5.57467000	1.81831200	2.49649600	C	-5.46511800	-2.18107000	-1.40733900
H	-4.35293000	3.92801500	2.49656100	C	-4.10557900	-4.21529300	1.40736100
H	-5.59648700	1.75077300	-2.49656900	C	-4.88814400	-3.26691600	-0.72766000
C	-1.22984300	-5.75266000	1.40876000	H	-5.44565100	-2.17330200	-2.49474800
C	-2.36381600	-5.38088400	0.73211200	C	-3.13689900	-4.97266700	0.72767400
C	-3.46374700	-4.75520200	1.40882900	H	-4.09091000	-4.20021000	2.49477200
C	-2.36530500	-5.38032600	-0.73212800	C	-4.10557900	-4.21529400	-1.40736100
C	-3.40625200	-4.79681600	-1.40882000	C	-2.03603700	-5.52049000	1.40737400
C	-4.34965100	-3.95343700	-0.73209000	C	-3.13689900	-4.97266700	-0.72767400
C	-4.34854200	-3.95450500	0.73209300	H	-4.09091100	-4.20021000	-2.49477200
C	-1.16044900	-5.76704100	-1.40890000	C	-0.84300400	-5.81796600	0.72765100
C	-5.06333700	-2.99702200	-1.40878600	H	-2.02878000	-5.50083000	2.49478300
C	-5.09897400	-2.93558200	1.40881100	C	-2.03603700	-5.52049100	-1.40737400
H	-1.22572000	-5.73270300	2.49646600	C	-0.84300400	-5.81796600	-0.72765100
H	-1.15632200	-5.74702300	-2.49660000	C	0.38548100	-5.87080300	1.40732500
C	-5.58167000	-1.84421800	0.73210100	H	-2.02878000	-5.50083100	-2.49478300
C	-5.58219300	-1.84282400	-0.73207800	C	0.38548100	-5.87080300	-1.40732500
C	-5.84500000	-0.67882400	-1.40884800	C	1.59650100	-5.65765900	0.72765300
C	-5.85272700	-0.60815400	1.40879100	H	0.38410700	-5.84990900	2.49473400
H	-3.39432200	-4.78025300	-2.49652100	C	1.59650100	-5.65765900	-0.72765300
H	-3.45183000	-4.73852200	2.49653000	H	0.38410800	-5.84991000	-2.49473400
H	-5.08129800	-2.92517300	2.49650900	C	2.74033000	-5.20649200	1.40737500
H	-5.04572100	-2.98673900	-2.49648600	C	2.74033000	-5.20649200	-1.40737500
H	-5.82473700	-0.67664100	-2.49654700	C	3.76004200	-4.51938900	0.72765600
H	-5.83247700	-0.60582300	2.49649400	H	2.73059600	-5.18799300	2.49478700

C	-0.06430900	5.88206000	-1.40876100	C	3.76004200	-4.51938900	-0.72765600
C	1.19467400	5.75403800	-0.73211600	H	2.73059600	-5.18799300	-2.49478700
C	1.19325000	5.75430600	0.73210600	C	4.62154500	-3.64200100	1.40735000
C	0.00684100	5.88217600	1.40887700	H	4.60508700	-3.62902900	2.49476100
H	-0.06427900	5.86170600	-2.49646400	C	4.62154500	-3.64200000	-1.40735000
H	0.00708600	5.86169800	2.49658000	H	4.60508700	-3.62902800	-2.49476100
**Cyc16_R**				**Cyc16_U**			
C	-1.08098700	-6.16810000	-0.73241800	C	6.19884100	-0.93098400	0.72784000
C	-1.08188100	-6.16795300	0.73227300	C	6.19885100	-0.93097400	-0.72783600
C	5.74613500	2.50316300	-1.40877200	C	-4.22124700	-4.64043200	1.40745600
C	5.12645400	3.59747700	-0.73209100	C	-5.04180400	-3.72509300	0.72785000
C	4.35094500	4.51152600	-1.40883500	C	-5.67572000	-2.67186600	1.40746100
C	5.12677400	3.59686500	0.73253100	C	-5.04179400	-3.72510400	-0.72785400
C	4.30855300	4.55181700	1.40919800	C	-5.67572600	-2.67186800	-1.40745600
C	3.36003600	5.28478300	0.73243200	C	-6.08336400	-1.51214000	-0.72785000
C	3.35947700	5.28509300	-0.73221200	C	-6.08336100	-1.51215700	0.72784600
C	6.11258600	1.36182800	-0.73209800	C	-3.23239800	-5.37075700	0.72783300
C	5.72219300	2.55647200	1.40926100	C	-4.22123300	-4.64042800	-1.40746800
H	4.29515700	4.53777700	2.49690500	H	-5.65787400	-2.66345500	-2.49491000
C	2.23861000	5.85383000	1.40906200	C	-6.26596800	-0.29646200	-1.40747700
C	2.29310700	5.83252400	-1.40902900	C	-6.26598100	-0.29646900	1.40746400
H	2.23163200	5.83566200	2.49677000	H	-6.24620500	-0.29550600	-2.49491800
H	2.28604000	5.81422700	-2.49674100	H	-6.24639100	-0.29552400	2.49492400
C	6.11253000	1.36101200	0.73253500	C	-3.23241300	-5.37075500	-0.72782800
H	5.72833700	2.49545100	-2.49648000	H	-4.20799600	-4.62586000	2.49491100
H	4.33752500	4.49748700	-2.49654400	H	-5.65768600	-2.66336900	2.49489600
H	5.70418800	2.54843200	2.49696600	H	-4.20786700	-4.62573300	-2.49490500
C	1.08107500	6.16804400	-0.73238100	C	-6.19884100	0.93098400	0.72784000
C	1.08180200	6.16791000	0.73230000	C	-6.19885100	0.93097400	-0.72783600
C	2.50320000	-5.74618000	-1.40884200	C	4.64043300	-4.22124600	1.40745600
C	3.59752500	-5.12641300	-0.73213500	C	3.72509400	-5.04180400	0.72785000
C	4.51154600	-4.35091000	-1.40880500	C	2.67186600	-5.67571900	1.40746100
C	3.59681000	-5.12677900	0.73251100	C	3.72510400	-5.04179400	-0.72785400
C	4.55180400	-4.30855400	1.40922900	C	2.67186800	-5.67572600	-1.40745600
C	5.28476400	-3.36004100	0.73252700	C	1.51214000	-6.08336400	-0.72785000
C	5.28518000	-3.35940300	-0.73214000	C	1.51215700	-6.08336100	0.72784600
C	2.55642200	-5.72219100	1.40918800	C	4.64042800	-4.22123300	-1.40746800
C	5.85387300	-2.23862600	1.40919600	C	0.29646200	-6.26596800	-1.40747700
C	5.83258700	-2.29306400	-1.40888600	C	0.29646900	-6.26598100	1.40746400
H	2.49552400	-5.72838600	-2.49655200	H	4.62586000	-4.20799600	2.49491100
H	2.54838600	-5.70418200	2.49689300	H	4.62573300	-4.20786700	-2.49490500
C	6.16815100	-1.08100700	-0.73218900	C	-0.93098400	-6.19884100	0.72784000
C	6.16794600	-1.08181400	0.73249400	C	-0.93097400	-6.19885100	-0.72783600
C	6.26457200	0.17198700	1.40922900	C	-2.12403700	-5.90247800	-1.40742200
C	6.26625000	0.11368000	-1.40884300	C	-2.12402500	-5.90244200	1.40744200
H	4.53767900	-4.29514000	2.49693900	H	2.66345500	-5.65787400	-2.49491100
H	4.49767400	-4.33748700	-2.49652000	H	2.66336900	-5.65768600	2.49489600
H	5.81444100	-2.28593000	-2.49659800	H	0.29552400	-6.24639100	2.49492400
H	5.83558600	-2.23167500	2.49690600	H	0.29550600	-6.24620500	-2.49491800
H	6.24490500	0.17140800	2.49693600	H	-2.11740300	-5.88401500	-2.49487700
H	6.24679800	0.11337200	-2.49655300	H	-2.11732100	-5.88379600	2.49487700
C	-5.74616400	-2.50317800	-1.40915800	C	4.22124700	4.64043200	1.40745600
C	-5.12642500	-3.59749100	-0.73246600	C	5.04180400	3.72509400	0.72785000
C	-4.35089000	-4.51152900	-1.40913000	C	5.67572000	2.67186600	1.40746100
C	-5.12682700	-3.59681200	0.73217700	C	5.04179400	3.72510400	-0.72785400
C	-4.30859500	-4.55183100	1.40890000	C	5.67572600	2.67186800	-1.40745600
C	-3.36009500	-5.28476400	0.73221900	C	6.08336400	1.51214000	-0.72785000
C	-3.35940600	-5.28515400	-0.73245000	C	6.08336200	1.51215700	0.72784600

C	-6.11251400	-1.36180100	-0.73251300	C	3.23239800	5.37075700	0.72783300
C	-5.72224200	-2.55645700	1.40886900	C	4.22123300	4.64042800	-1.40746800
H	-4.29521400	-4.53770800	2.49661000	H	5.65787400	2.66345500	-2.49491000
C	-2.23867800	-5.85390400	1.40892300	C	6.26596800	0.29646200	-1.40747700
C	-2.29303300	-5.83255600	-1.40915600	C	6.26598100	0.29646900	1.40746400
H	-2.23178900	-5.83559900	2.49663400	H	6.24620500	0.29550600	-2.49491800
H	-2.28584500	-5.81440700	-2.49686700	H	6.24639200	0.29552400	2.49492400
C	-6.11257200	-1.36095200	0.73213400	C	3.23241300	5.37075500	-0.72782800
H	-5.72835500	-2.49546200	-2.49686800	H	4.20799600	4.62586000	2.49491100
H	-4.33744500	-4.49764200	-2.49684300	H	5.65768600	2.66336900	2.49489600
H	-5.70426100	-2.54845300	2.49657500	H	4.20786700	4.62573300	-2.49490500
C	-0.11364500	6.26614600	-1.40908800	C	-5.90244200	2.12402500	1.40744200
C	-4.51152300	4.35090100	-1.40916800	C	-2.67186600	5.67571900	1.40746100
C	-4.55187800	4.30857800	1.40886100	C	-2.67186800	5.67572600	-1.40745600
C	-5.28482600	3.36007900	0.73211100	C	-1.51214000	6.08336400	-0.72785000
C	-5.28509900	3.35946600	-0.73253300	C	-1.51215700	6.08336100	0.72784600
C	-0.17202200	6.26456000	1.40898800	C	-5.90247900	2.12403700	-1.40742200
C	-5.85392100	2.23866300	1.40877600	C	-0.29646200	6.26596800	-1.40747700
C	-5.83253200	2.29306600	-1.40931200	C	-0.29646900	6.26598100	1.40746400
H	-0.11327200	6.24665200	-2.49679600	H	-5.88379600	2.11732100	2.49487700
H	-0.17150700	6.24493900	2.49669800	H	-5.88401600	2.11740300	-2.49487700
C	-6.16804600	1.08105200	-0.73261800	C	0.93098400	6.19884100	0.72784000
C	-6.16798200	1.08186500	0.73206800	C	0.93097400	6.19885100	-0.72783600
C	-6.26459000	-0.17194700	1.40880900	C	2.12403700	5.90247800	-1.40742200
C	-6.26615600	-0.11367900	-1.40927300	C	2.12402500	5.90244200	1.40744200
H	-4.53784900	4.29521700	2.49656800	H	-2.66345500	5.65787400	-2.49491100
H	-4.49746300	4.33745400	-2.49687500	H	-2.66336900	5.65768600	2.49489600
H	-5.81422900	2.28594600	-2.49702200	H	-0.29552400	6.24639100	2.49492400
H	-5.83574600	2.23174500	2.49648500	H	-0.29550600	6.24620500	-2.49491800
H	-6.24501600	-0.17134200	2.49651700	H	2.11740300	5.88401500	-2.49487700
H	-6.24666600	-0.11337300	-2.49698200	H	2.11732100	5.88379600	2.49487700
C	0.17191500	-6.26453400	1.40905700	C	5.90247900	-2.12403700	-1.40742200
C	1.36094400	-6.11253100	0.73243100	C	5.37075500	-3.23241300	-0.72782800
C	1.36185300	-6.11252000	-0.73222100	C	5.37075700	-3.23239800	0.72783300
C	0.11371200	-6.26620500	-1.40901800	C	5.90244200	-2.12402500	1.40744200
H	0.17125300	-6.24490800	2.49676400	H	5.88401600	-2.11740300	-2.49487700
H	0.11346400	-6.24676100	-2.49673000	H	5.88379600	-2.11732100	2.49487700
C	-3.59688400	5.12678900	0.73219300	C	-3.72510400	5.04179400	-0.72785400
C	-2.55652000	5.72219700	1.40894200	C	-4.64042800	4.22123300	-1.40746800
C	-1.36102400	6.11251400	0.73224500	C	-5.37075500	3.23241300	-0.72782800
C	-1.36178000	6.11252400	-0.73238500	C	-5.37075700	3.23239800	0.72783300
C	-2.50314100	5.74607300	-1.40908800	C	-4.64043300	4.22124700	1.40745600
C	-3.59745500	5.12643100	-0.73242700	C	-3.72509400	5.04180400	0.72785000
H	-2.54851400	5.70421600	2.49664800	H	-4.62573300	4.20786700	-2.49490500
H	-2.49538700	5.72825600	-2.49679400	H	-4.62586000	4.20799600	2.49491100
**Cyc17_R**				**Cyc17_U**			
C	3.60566900	-5.60144600	-1.40818800	C	6.66351700	0.07976200	-1.40730400
C	4.54445700	-4.86375900	-0.73206600	C	6.53119100	1.30176900	-0.72783300
C	5.38564500	-3.92053700	-1.40878900	C	6.56030300	-1.14503700	-0.72781800
C	4.54579200	-4.86259800	0.73215800	H	6.64493700	0.07953700	-2.49477900
C	5.34553800	-3.97611000	1.40836800	C	6.18478900	2.48105300	-1.40731400
C	5.99594600	-2.89248600	0.73171700	C	6.53119000	1.30176800	0.72784400
C	5.99484200	-2.89381700	-0.73250800	C	5.61971900	3.57257200	-0.72784100
C	2.48051400	-6.17691400	-0.73147300	H	6.16754200	2.47413200	-2.49479200
C	3.54777200	-5.63796500	1.40894600	C	6.18478700	2.48105100	1.40732600
H	5.33142700	-3.96555600	2.49614000	C	6.66351500	0.07976100	1.40731400
C	6.42148100	-1.77700500	1.40796600	C	5.61971800	3.57257100	0.72785300
C	6.43848200	-1.71032000	-1.40914200	H	6.16753800	2.47412900	2.49480400
H	6.40473800	-1.77236400	2.49574000	C	4.87038900	4.54688700	1.40732900

H	6.42096100	-1.70568000	-2.49691700	C	4.87039200	4.54688800	-1.40731800
C	2.48192300	-6.17607000	0.73276800	C	6.56030200	-1.14503700	0.72782700
H	3.59598300	-5.58659800	-2.49597100	C	6.24212100	-2.33229000	-1.40730000
H	5.37099800	-3.90994300	-2.49656400	H	6.64493400	0.07953500	2.49478900
H	3.53834200	-5.62277500	2.49671600	H	4.85680100	4.53419900	2.49480700
C	6.63566100	-0.53289700	-0.73278400	C	3.94904100	5.36042300	0.72784000
C	6.63633000	-0.53146700	0.73141200	H	4.85680500	4.53420100	-2.49479500
C	-5.24502900	-4.10727900	-1.40877400	C	3.94904200	5.36042400	-0.72783000
C	-4.42412600	-4.97394600	-0.73218600	C	6.24211900	-2.33229100	1.40730700
C	-3.40712500	-5.72474200	-1.40841000	C	2.89838500	5.99834600	-1.40732100
C	-4.42322800	-4.97478000	0.73204900	C	2.89838300	5.99834500	1.40733100
C	-3.46654900	-5.68856000	1.40875500	C	5.70328300	-3.43701700	0.72783700
C	-2.32721800	-6.23610100	0.73253500	H	6.22476400	-2.32580800	2.49478300
C	-2.32849600	-6.23592700	-0.73170500	C	5.70328300	-3.43701700	-0.72783200
C	-5.28785500	-4.05265400	1.40832300	C	4.97772300	-4.42911200	1.40734000
C	-1.17714900	-6.55603200	1.40908300	C	4.97772500	-4.42911100	-1.40733700
C	-1.10897100	-6.56866500	-1.40800800	C	4.07616500	-5.26457000	-0.72784500
H	-5.23080700	-4.09651800	-2.49655100	H	4.96394500	-4.41683200	-2.49481500
H	-5.27412000	-4.04171400	2.49609400	C	3.04114300	-5.92748000	-1.40733200
C	0.08159600	-6.65577400	-0.73149400	C	4.07616400	-5.26457100	0.72784700
C	0.08298300	-6.65529500	0.73272100	C	1.89868000	-6.38089900	-0.72783400
C	1.27101200	-6.53858200	1.40922800	H	3.03269300	-5.91099800	-2.49481100
C	1.33885700	-6.52569800	-1.40789300	C	0.69408700	-6.62529600	-1.40733000
H	-3.45760800	-5.67308600	2.49652600	C	1.89867900	-6.38090000	0.72783100
H	-3.39776600	-5.70976700	-2.49619300	H	0.69216900	-6.60691000	-2.49480900
H	-1.10579400	-6.55136400	-2.49579600	C	-0.53500700	-6.63566200	-0.72783500
H	-1.17420500	-6.53813000	2.49685300	C	3.04114100	-5.92748100	1.40733200
H	1.26739900	-6.52086200	2.49699400	C	0.69408600	-6.62529700	1.40732500
H	1.33549000	-6.50844700	-2.49567900	H	3.03269000	-5.91100100	2.49481000
C	-2.51520600	6.16872000	-1.40818000	H	0.69216500	-6.60691300	2.49480400
C	-3.57345000	5.61610200	-0.73184900	C	-0.53500800	-6.63566200	0.72782800
C	-4.57376200	4.84363600	-1.40852700	H	4.96394100	-4.41683400	2.49481900
C	-3.57485200	5.61513500	0.73237300	H	6.22476800	-2.32580600	-2.49477600
C	-4.52374400	4.89051000	1.40859400	H	2.89034300	5.98170000	-2.49479900
C	-5.36221200	3.94481000	0.73196000	C	1.74542500	6.42427800	-0.72782600
C	-5.36110600	3.94612200	-0.73228600	H	2.89034000	5.98169800	2.49480900
C	-1.30329500	6.52752900	-0.73163700	C	1.74542400	6.42427800	0.72783500
C	-2.45126000	6.19388700	1.40895400	C	-1.74658900	-6.42866900	1.40730700
H	-4.51172400	4.87745000	2.49636500	H	-1.74174300	-6.41080700	2.49478500
C	-5.98538200	2.92651200	1.40829000	C	-2.89650300	-5.99455900	0.72782900
C	-6.01468200	2.86440400	-1.40888600	C	-1.74658700	-6.42866800	-1.40731500
H	-5.96960400	2.91885000	2.49606500	H	-1.74174000	-6.41080400	-2.49479200
H	-5.99845700	2.85663700	-2.49666700	C	-2.89650200	-5.99455800	-0.72783800
C	-1.30482900	6.52690900	0.73259200	C	0.53533900	6.63987600	1.40730800
H	-2.50859600	6.15236800	-2.49597000	C	-0.69365700	6.62086500	0.72783300
H	-4.56149600	4.83071200	-2.49630900	H	0.53385400	6.62146500	2.49478600
H	-2.44457800	6.17718200	2.49672200	C	-1.89993800	6.38501600	1.40734100
C	6.62185700	0.73104100	-1.40924700	C	-0.69365600	6.62086500	-0.72782800
C	4.40096500	5.00110700	-1.40844400	C	-3.03917100	5.92352800	0.72784200
C	4.45296900	4.95470400	1.40867100	H	-1.89467300	6.36729500	2.49481800
C	3.43353800	5.70232300	0.73248100	C	-3.03917000	5.92352800	-0.72784200
C	3.43469100	5.70187300	-0.73176700	C	-4.07888800	5.26800600	1.40733200
C	6.63007700	0.66225200	1.40789500	C	-1.89993600	6.38501700	-1.40733800
C	2.36185000	6.22801300	1.40912200	C	-4.07888600	5.26800700	-1.40733400
C	2.29709700	6.25298600	-1.40801800	H	-1.89467000	6.36729800	-2.49481500
H	6.60378200	0.72920600	-2.49702500	C	-4.97457900	4.42627200	0.72783700
H	6.61279800	0.66031700	2.49567200	H	-4.06755500	5.25336400	2.49481200
C	1.14283300	6.55723100	-0.73145200	C	-4.97457800	4.42627300	-0.72784100
C	1.14133900	6.55710300	0.73275700	H	-4.06755100	5.25336600	-2.49481400
C	-0.04801500	6.66062900	1.40914600	C	-5.70722100	3.43937200	-1.40731300

C	-0.11684800	6.66030900	-1.40798100	C	-5.70722300	3.43937000	1.40730700
H	4.44143100	4.94128100	2.49644600	C	-6.23819000	2.33085100	0.72783000
H	4.38902000	4.98808000	-2.49623300	H	-5.69130100	3.42976900	2.49478400
H	2.29076400	6.23660400	-2.49580500	C	-6.23818900	2.33085100	-0.72783700
H	2.35573800	6.21101100	2.49688700	C	-6.56467900	1.14585900	1.40731700
H	-0.04780100	6.64257800	2.49691300	C	-6.56467700	1.14586000	-1.40732600
H	-0.11662100	6.64263000	-2.49576700	C	-6.65911400	-0.07963400	-0.72783000
C	-6.39490200	-1.86898700	1.40804300	H	-6.54639100	1.14265700	-2.49480200
C	-5.92173500	-3.04114800	0.73175600	C	-6.53536900	-1.30253800	-1.40730100
C	-5.92209400	-3.03984900	-0.73244200	C	-6.65911500	-0.07963500	0.72782000
C	-6.37429800	-1.93515700	-1.40909300	C	-6.18055600	-2.47934300	-0.72784500
H	-6.37823300	-1.86379700	2.49581700	H	-6.51717200	-1.29890500	-2.49477700
H	-6.35694100	-1.93022300	-2.49686500	C	-6.53537100	-1.30253900	1.40729000
C	5.26216400	4.07723400	0.73196400	C	-6.18055700	-2.47934300	0.72783400
C	5.94290700	3.01206100	1.40817800	H	-6.51717500	-1.29890600	2.49476600
C	6.38025600	1.90140200	0.73155000	C	-5.62320800	-3.57481800	1.40731800
C	6.38014300	1.90004000	-0.73270700	C	-5.62320700	-3.57481700	-1.40733000
C	5.91039300	3.07363300	-1.40898300	H	-6.54639500	1.14265500	2.49479300
C	5.26273900	4.07622800	-0.73226800	H	-5.60757600	-3.56487300	2.49479500
H	5.92742000	3.00384400	2.49595400	C	-4.86705300	-4.54383100	0.72783500
H	5.89423500	3.06561500	-2.49676400	H	-5.60757300	-3.56487100	-2.49480600
C	-6.42546000	1.74170900	0.73171300	C	-4.86705200	-4.54383100	-0.72784600
C	-6.63851800	0.56697700	1.40802000	H	-5.69129700	3.42977200	-2.49479000
C	-6.62072900	-0.69682300	0.73148600	C	0.53534100	6.63987700	-1.40730100
C	-6.62035500	-0.69528000	-0.73272500	H	0.53385800	6.62146800	-2.49477900
C	-6.64316700	0.49833700	-1.40910100	C	-3.95158400	-5.36397000	1.40732700
C	-6.42467900	1.74320300	-0.73252000	H	-3.94060200	-5.34902700	2.49480500
H	-6.62104500	0.56554200	2.49579600	C	-3.95158200	-5.36396800	-1.40733700
H	-6.62508400	0.49692600	-2.49687500	H	-3.94059900	-5.34902500	-2.49481500
**Cyc18_R**				**Cyc18_U**			
C	-1.19274400	6.94594400	1.40905600	C	-1.22466700	6.94370700	1.40729200
C	-0.00043500	7.04285200	0.73243900	C	-0.00000400	7.04674900	0.72783300
C	1.25500500	6.93474800	1.40897400	C	1.22465900	6.94370800	1.40729200
C	0.00043500	7.04285200	-0.73243900	C	-0.00000400	7.04674900	-0.72783300
C	1.19274400	6.94594400	-1.40905600	C	1.22465900	6.94370800	-1.40729200
C	2.40949200	6.61828800	-0.73255200	C	2.41072400	6.62164300	-0.72786000
C	2.40870100	6.61855000	0.73236100	C	2.41072400	6.62164300	0.72786000
C	-2.40949200	6.61828800	0.73255200	C	-2.41073200	6.62164100	0.72786000
C	-1.25500500	6.93474800	-1.40897400	C	-1.22466700	6.94370700	-1.40729200
H	1.18999300	6.92883500	-2.49682600	H	1.22159900	6.92638200	-2.49482400
C	3.49670300	6.11935600	-1.40919100	C	3.52631900	6.10612800	-1.40731800
C	3.55161000	6.08778700	1.40884100	C	3.52631900	6.10612800	1.40731800
H	3.48823600	6.10430000	-2.49696900	H	3.51757000	6.09096900	-2.49485200
H	3.54285600	6.07297900	2.49661400	H	3.51757000	6.09096900	2.49485200
C	-2.40870100	6.61855000	-0.73236100	C	-2.41073200	6.62164100	-0.72786000
H	-1.18999300	6.92883500	2.49682600	H	-1.22160700	6.92638000	2.49482400
H	1.25172800	6.91765300	2.49674600	H	1.22159900	6.92638200	2.49482400
H	-1.25172800	6.91765300	-2.49674600	H	-1.22160700	6.92638000	-2.49482400
C	4.52746700	5.39597700	0.73219400	C	4.53089900	5.39812300	0.72785600
C	4.52800400	5.39523300	-0.73272800	C	4.53089900	5.39812300	-0.72785600
C	-3.55161000	6.08778700	-1.40884100	C	-3.52632600	6.10612400	-1.40731800
C	-4.52746700	5.39597700	-0.73219400	C	-4.53090600	5.39811800	-0.72785600
C	-5.41996900	4.50647800	-1.40872100	C	-5.40324800	4.53250800	-1.40732500
C	-6.10016800	3.52240000	-0.73216700	C	-6.10548900	3.52386500	-0.72786700
C	-6.63431400	2.38115700	-1.40878400	C	-6.62905600	2.41201000	-1.40730300
C	-6.93654800	1.22367600	-0.73228700	C	-6.94357100	1.22395300	-0.72784600
C	-6.93661200	1.22257400	0.73260500	C	-6.94357100	1.22395300	0.72784600
C	-7.04792200	0.03139100	1.40900500	C	-7.05488700	-0.00000400	1.40730700
C	-6.61217600	2.44008800	1.40928300	C	-6.62905600	2.41201000	1.40730300

C	-6.10039500	3.52138500	0.73278200	C	-6.10548900	3.52386500	0.72786700
C	-7.04792200	-0.03139100	-1.40900500	C	-7.05488700	-0.00000400	-1.40730700
C	-5.37888700	4.55445600	1.40935600	C	-5.40324800	4.53250800	1.40732500
H	-7.03054200	0.03094500	2.49677700	H	-7.03724200	-0.00000400	2.49483800
H	-7.03054200	-0.03094500	-2.49677700	H	-7.03724200	-0.00000400	-2.49483800
C	-4.52800400	5.39523300	0.73272800	C	-4.53090600	5.39811800	0.72785600
C	-3.49670300	6.11935600	1.40919100	C	-3.52632600	6.10612400	1.40731800
H	-6.61814200	2.37559200	-2.49656100	H	-6.61248000	2.40597200	-2.49483600
H	-6.59581200	2.43381800	2.49705400	H	-6.61248000	2.40597200	2.49483600
H	-5.36565000	4.54303700	2.49712500	H	-5.38972900	4.52118500	2.49485800
H	-5.40670400	4.49570900	-2.49650000	H	-5.38972900	4.52118500	-2.49485800
H	-3.54285600	6.07297900	-2.49661400	H	-3.51757700	6.09096500	-2.49485200
H	-3.48823600	6.10430000	2.49696900	H	-3.51757700	6.09096500	2.49485200
C	5.41996900	4.50647800	1.40872100	C	5.40324300	4.53251400	1.40732500
C	5.37888700	4.55445600	-1.40935600	C	5.40324300	4.53251400	-1.40732500
H	5.40670400	4.49570900	2.49650000	H	5.38972400	4.52119100	2.49485800
H	5.36565000	4.54303700	-2.49712500	H	5.38972400	4.52119100	-2.49485800
C	-6.93661200	-1.22257400	-0.73260500	C	-6.94356900	-1.22396100	-0.72784600
C	-6.61217600	-2.44008800	-1.40928300	C	-6.62905300	-2.41201800	-1.40730300
C	-6.93654800	-1.22367600	0.73228700	C	-6.94356900	-1.22396100	0.72784600
C	-6.63431400	-2.38115700	1.40878400	C	-6.62905300	-2.41201800	1.40730300
H	-6.59581200	-2.43381800	-2.49705400	H	-6.61247700	-2.40597900	-2.49483600
H	-6.61814200	-2.37559200	2.49656100	H	-6.61247700	-2.40597900	2.49483600
C	6.10039500	3.52138500	-0.73278200	C	6.10548500	3.52387200	-0.72786700
C	6.61217600	2.44008800	-1.40928300	C	6.62905300	2.41201800	-1.40730300
C	6.93661200	1.22257400	-0.73260500	C	6.94356900	1.22396100	-0.72784600
C	7.04792200	0.03139100	-1.40900500	C	7.05488700	0.00000400	-1.40730700
C	6.93654800	-1.22367600	-0.73228700	C	6.94357100	-1.22395300	-0.72784600
C	6.93661200	-1.22257400	0.73260500	C	6.94357100	-1.22395300	0.72784600
C	7.04792200	-0.03139100	1.40900500	C	7.05488700	0.00000400	1.40730700
C	6.63431400	-2.38115700	-1.40878400	C	6.62905600	-2.41201000	-1.40730300
C	6.61217600	-2.44008800	1.40928300	C	6.62905600	-2.41201000	1.40730300
C	6.10039500	-3.52138500	0.73278200	C	6.10548900	-3.52386500	0.72786700
C	6.10016800	-3.52240000	-0.73216700	C	6.10548900	-3.52386500	-0.72786700
C	5.41996900	-4.50647800	-1.40872100	C	5.40324800	-4.53250800	-1.40732500
C	4.52746700	-5.39597700	-0.73219400	C	4.53090600	-5.39811800	-0.72785600
C	3.55161000	-6.08778700	-1.40884100	C	3.52632600	-6.10612400	-1.40731800
C	2.40870100	-6.61855000	-0.73236100	C	2.41073200	-6.62164000	-0.72786000
C	2.40949200	-6.61828800	0.73255200	C	2.41073200	-6.62164000	0.72786000
C	3.49670300	-6.11935600	1.40919100	C	3.52632600	-6.10612400	1.40731800
C	1.19274400	-6.94594400	1.40905600	C	1.22466700	-6.94370700	1.40729200
C	1.25500500	-6.93474800	-1.40897400	C	1.22466700	-6.94370700	-1.40729200
C	-0.00043500	-7.04285200	-0.73243900	C	0.00000400	-7.04674900	-0.72783300
C	0.00043500	-7.04285200	0.73243900	C	0.00000400	-7.04674900	0.72783300
C	4.52800400	-5.39523300	0.73272800	C	4.53090600	-5.39811800	0.72785600
H	1.25172800	-6.91765300	-2.49674600	H	1.22160700	-6.92638000	-2.49482400
C	-1.19274400	-6.94594400	-1.40905600	C	-1.22465900	-6.94370800	-1.40729200
C	-1.25500500	-6.93474800	1.40897400	C	-1.22465900	-6.94370800	1.40729200
H	-1.18999300	-6.92883500	-2.49682600	H	-1.22159900	-6.92638200	-2.49482400
H	-1.25172800	-6.91765300	2.49674600	H	-1.22159900	-6.92638200	2.49482400
H	3.48823600	-6.10430000	2.49696900	H	3.51757700	-6.09096500	2.49485200
H	1.18999300	-6.92883500	2.49682600	H	1.22160700	-6.92638000	2.49482400
H	3.54285600	-6.07297900	-2.49661400	H	3.51757700	-6.09096500	-2.49485200
C	5.37888700	-4.55445600	1.40935600	C	5.40324800	-4.53250800	1.40732500
H	7.03054200	0.03094500	-2.49677700	H	7.03724200	0.00000400	-2.49483800
H	7.03054200	-0.03094500	2.49677700	H	7.03724200	0.00000400	2.49483800
H	6.59581200	-2.43381800	2.49705400	H	6.61248000	-2.40597200	2.49483600
H	6.61814200	-2.37559200	-2.49656100	H	6.61248000	-2.40597200	-2.49483600
H	5.40670400	-4.49570900	-2.49650000	H	5.38972900	-4.52118500	-2.49485800
H	5.36565000	-4.54303700	2.49712500	H	5.38972900	-4.52118500	2.49485800

C	6.10016800	3.52240000	0.73216700	C	6.10548500	3.52387200	0.72786700
C	6.63431400	2.38115700	1.40878400	C	6.62905300	2.41201800	1.40730300
C	6.93654800	1.22367600	0.73228700	C	6.94356900	1.22396100	0.72784600
H	6.59581200	2.43381800	-2.49705400	H	6.61247700	2.40597900	-2.49483600
H	6.61814200	2.37559200	2.49656100	H	6.61247700	2.40597900	2.49483600
C	-2.40949200	-6.61828800	-0.73255200	C	-2.41072400	-6.62164300	-0.72786000
C	-3.49670300	-6.11935600	-1.40919100	C	-3.52631900	-6.10612800	-1.40731800
C	-2.40870100	-6.61855000	0.73236100	C	-2.41072400	-6.62164300	0.72786000
C	-3.55161000	-6.08778700	1.40884100	C	-3.52631900	-6.10612800	1.40731800
H	-3.48823600	-6.10430000	-2.49696900	H	-3.51757000	-6.09096900	-2.49485200
H	-3.54285600	-6.07297900	2.49661400	H	-3.51757000	-6.09096900	2.49485200
C	-4.52800400	-5.39523300	-0.73272800	C	-4.53089900	-5.39812300	-0.72785600
C	-5.37888700	-4.55445600	-1.40935600	C	-5.40324300	-4.53251500	-1.40732500
C	-6.10039500	-3.52138500	-0.73278200	C	-6.10548500	-3.52387200	-0.72786700
C	-6.10016800	-3.52240000	0.73216700	C	-6.10548500	-3.52387200	0.72786700
C	-5.41996900	-4.50647800	1.40872100	C	-5.40324300	-4.53251500	1.40732500
C	-4.52746700	-5.39597700	0.73219400	C	-4.53089900	-5.39812300	0.72785600
H	-5.36565000	-4.54303700	-2.49712500	H	-5.38972400	-4.52119100	-2.49485800
H	-5.40670400	-4.49570900	2.49650000	H	-5.38972400	-4.52119100	2.49485800
**Cyc19_R**				**Cyc19_U**			
C	-1.22099000	-7.33375400	1.40910700	C	-1.22496900	1.40719700	-7.34215100
C	0.03784800	-7.43012800	0.73215100	C	0.00000000	0.72791500	-7.43980400
C	1.22839500	-7.33356000	1.40785800	C	1.22496900	1.40719700	-7.34215100
C	0.03563500	-7.43011000	-0.73218600	C	0.00000000	-0.72791500	-7.43980400
C	1.29347100	-7.32135200	-1.40912800	C	1.22496900	-1.40719700	-7.34215100
C	2.44826700	-7.01629100	-0.73337100	C	2.41520900	-0.72796100	-7.03642700
C	2.45052200	-7.01641400	0.73099600	C	2.41520900	0.72796100	-7.03642700
C	-2.37873500	-7.04005200	0.73336600	C	-2.41520900	0.72796100	-7.03642700
C	-1.15581000	-7.34523200	-1.40788100	C	-1.22496900	-1.40719700	-7.34215100
H	1.29033100	-7.30531000	-2.49693400	H	1.22222100	-2.49474300	-7.32572100
C	3.60291500	-6.50533000	-1.41016800	C	3.54201300	-1.40723900	-6.54622700
C	3.54549800	-6.53940500	1.40683600	C	3.54201300	1.40723900	-6.54622700
H	3.59467800	-6.49068400	-2.49796600	H	3.53412200	-2.49477500	-6.53165400
H	3.53844200	-6.52605000	2.49468800	H	3.53412200	2.49477500	-6.53165400
C	-2.38099800	-7.04016600	-0.73100100	C	-2.41520900	-0.72796100	-7.03642700
H	-1.21800600	-7.31767200	2.49693700	H	-1.22222100	2.49474300	-7.32572100
H	1.22610900	-7.31806600	2.49569900	H	1.22222100	2.49474300	-7.32572100
H	-1.15370500	-7.32971400	-2.49569700	H	-1.22222100	-2.49474300	-7.32572100
C	4.59864900	-5.84269400	0.73022200	C	4.56847400	0.72792300	-5.87060800
C	4.59642400	-5.84264000	-0.73414900	C	4.56847400	-0.72792300	-5.87060800
C	-7.42009600	-0.61651300	1.40905000	C	-3.54201300	-1.40723900	-6.54622700
C	-7.20389000	-1.86123500	0.73345900	C	-4.56847400	-0.72792300	-5.87060800
C	-6.81614000	-2.99027800	1.41033900	C	-5.47517600	-1.40721500	-5.04124700
C	-7.20502300	-1.86083800	-0.73089100	C	-6.22671200	-0.72792600	-4.06897200
C	-6.79072300	-3.05413100	-1.40664200	C	-6.81473100	-1.40717000	-2.98994500
C	-6.20940400	-4.09695500	-0.73001200	C	-7.20951000	-0.72788800	-1.82623400
C	-6.20741900	-4.09706100	0.73438700	C	-7.20951000	0.72788800	-1.82623400
C	-7.41501700	-0.68574000	-1.40786400	C	-7.41517400	1.40721900	-0.61470500
C	-5.42966800	-5.09042000	-1.40629900	C	-6.81473100	1.40717000	-2.98994500
C	-5.47330300	-5.03884200	1.41072800	C	-6.22671200	0.72792600	-4.06897200
H	-7.40403400	-0.61432700	2.49688200	C	-7.41517400	-1.40721900	-0.61470500
H	-7.39951100	-0.68513800	-2.49568300	C	-5.47517600	1.40721500	-5.04124700
C	-4.53836500	-5.88763900	0.73419800	H	-7.39865100	2.49475500	-0.61334700
C	-4.54061900	-5.88773500	-0.73017200	H	-7.39865100	-2.49475500	-0.61334700
C	-3.48064900	-6.57399400	-1.40681100	C	-4.56847400	0.72792300	-5.87060800
C	-3.53834700	-6.54049300	1.41018900	C	-3.54201300	1.40723900	-6.54622700
H	-6.77689600	-3.04854100	-2.49447000	H	-6.79950500	-2.49470600	-2.98326900
H	-6.80088900	-2.98291200	2.49816000	H	-6.79950500	2.49470600	-2.98326900

H	-5.46081400	-5.02716500	2.49854900	H	-5.46292900	2.49480600	-5.03003300
H	-5.41886800	-5.08043200	-2.49413000	H	-5.46292900	-2.49480600	-5.03003300
H	-3.47378100	-6.56059000	-2.49463800	H	-3.53412200	-2.49477500	-6.53165400
H	-3.53022500	-6.52573600	2.49801300	H	-3.53412200	2.49477500	-6.53165400
C	4.56792000	5.86948700	1.41100200	C	5.47517600	1.40721500	-5.04124700
C	3.50619000	6.55315800	0.73445200	C	5.47517600	-1.40721500	-5.04124700
C	2.41230200	7.03273900	1.41023400	H	5.46292900	2.49480600	-5.03003300
C	3.50867700	6.55378700	-0.72993800	H	5.46292900	-2.49480600	-5.03003300
C	2.35041100	7.05638900	-1.40680000	C	6.22671200	-0.72792600	-4.06897200
C	1.18894800	7.33522700	-0.73118900	C	6.81473100	-1.40717000	-2.98994500
C	1.18648600	7.33489100	0.73315900	C	7.20951000	-0.72788800	-1.82623400
C	5.44693500	5.06133300	0.73464400	C	7.41517400	-1.40721900	-0.61470500
C	4.51680600	5.91360400	-1.40601200	C	7.41104600	-0.72796400	0.61413600
H	2.34611900	7.04206500	-2.49462700	C	7.41104600	0.72796400	0.61413600
C	-0.06953900	7.43452600	-1.40844600	C	7.41517400	1.40721900	-0.61470500
C	-0.00395600	7.43476400	1.40856200	C	7.21279000	-1.40725400	1.82686500
H	-0.06867000	7.41853300	-2.49625800	C	7.21279000	1.40725400	1.82686500
H	-0.00465400	7.41868300	2.49639800	C	6.81036600	0.72792700	2.98795900
C	5.44916200	5.06173700	-0.72974500	C	6.81036600	-0.72792700	2.98795900
H	4.55736700	5.85575600	2.49882100	C	6.22928600	-1.40723600	4.07073300
H	2.40642500	7.01683500	2.49805900	C	5.47179800	-0.72797200	5.03837200
H	4.50800500	5.90212700	-2.49384600	C	4.57070900	-1.40723800	5.87386000
C	5.47972700	-5.03657600	1.40635300	C	3.54009900	-0.72797300	6.54313700
C	7.42141600	-0.61244700	1.40784000	C	3.54009900	0.72797300	6.54313700
C	7.42580700	-0.54327700	-1.40907700	C	4.57070900	1.40723800	5.87386000
C	7.41288500	0.65035900	-0.73207500	C	2.41648600	1.40719600	7.04061800
C	7.41279400	0.65087700	0.73227000	C	2.41648600	-1.40719600	7.04061800
C	5.52293800	-4.98465400	-1.41067100	C	1.22434000	-0.72795000	7.33870500
C	7.20059300	1.89576100	-1.40761700	C	1.22434000	0.72795000	7.33870500
C	7.21663000	1.82828000	1.40935800	C	5.47179800	0.72797200	5.03837200
H	5.46876200	-5.02664800	2.49420700	H	2.41105400	-2.49476400	7.02484300
H	5.51038600	-4.97316800	-2.49846800	C	0.00000000	-1.40722000	7.44418200
C	6.79816700	3.02022600	0.73377100	C	0.00000000	1.40722000	7.44418200
C	6.79955000	3.02008700	-0.73061600	H	0.00000000	-2.49475500	7.42750600
C	6.19439600	4.12894000	-1.40633600	H	0.00000000	2.49475500	7.42750600
C	6.22954400	4.06987500	1.41061600	H	4.56046200	2.49473100	5.86067400
H	7.40973800	-0.54132800	-2.49688400	H	2.41105400	2.49476400	7.02484300
H	7.40587000	-0.61194600	2.49568400	H	4.56046200	-2.49473100	5.86067400
H	7.20094300	1.82342200	2.49718800	C	6.22928600	1.40723600	4.07073300
H	7.18558600	1.89266400	-2.49543600	H	7.39865100	-2.49475500	-0.61334700
H	6.18198900	4.12130600	-2.49417100	H	7.39865100	2.49475500	-0.61334700
H	6.21558900	4.06006000	2.49843900	H	7.19672300	2.49473500	1.82279300
C	-6.26941000	4.00815100	-1.41071700	H	7.19672300	-2.49473500	1.82279300
C	-6.23506800	4.06764000	1.40623700	H	6.21538300	-2.49477500	4.06165200
H	-6.25528700	3.99846600	-2.49851400	H	6.21538300	2.49477500	4.06165200
H	-6.22264200	4.06015500	2.49409800	C	6.22671200	0.72792600	-4.06897200
C	7.22195500	-1.79005200	-0.73343300	C	6.81473100	1.40717000	-2.98994500
C	6.84542600	-2.92289700	-1.41029600	C	7.20951000	0.72788800	-1.82623400
C	6.24767200	-4.03563000	-0.73433700	H	6.79950500	-2.49470600	-2.98326900
C	6.24960800	-4.03545400	0.73006100	H	6.79950500	2.49470600	-2.98326900
C	6.82054800	-2.98690700	1.40668900	C	-1.22434000	-0.72795000	7.33870500
C	7.22305500	-1.78959300	0.73091600	C	-2.41648600	-1.40719600	7.04061800
H	6.83015800	-2.91574000	-2.49809300	C	-1.22434000	0.72795000	7.33870500
H	6.80659200	-2.98141600	2.49454000	C	-2.41648600	1.40719600	7.04061800
C	-1.25893600	7.32287100	-0.73305300	H	-2.41105400	-2.49476400	7.02484300
C	-2.48169800	7.00865800	-1.41015900	H	-2.41105400	2.49476400	7.02484300
C	-2.42007800	7.03279500	1.40687800	C	-3.54009900	-0.72797300	6.54313700
C	-1.26140900	7.32310300	0.73129500	C	-4.57070900	-1.40723800	5.87386000
H	-2.47563600	6.99287400	-2.49796000	C	-5.47179800	-0.72797200	5.03837200
H	-2.41564100	7.01844900	2.49473000	C	-6.22928600	-1.40723600	4.07073300

C	-3.57079900	6.51826000	-0.73441700	H	-6.21538300	-2.49477500	4.06165200
C	-4.62568500	5.82412700	-1.41102000	C	-5.47179800	0.72797200	5.03837200
C	-5.49669600	5.00731700	-0.73470800	C	-6.22928600	1.40723600	4.07073300
C	-5.49901900	5.00772700	0.72968000	H	-6.21538300	2.49477500	4.06165200
C	-4.57512800	5.86873500	1.40600000	C	-4.57070900	1.40723800	5.87386000
C	-3.57333100	6.51883500	0.72997300	C	-3.54009900	0.72797300	6.54313700
H	-4.61494200	5.81052200	-2.49881400	H	-4.56046200	-2.49473100	5.86067400
H	-4.56623500	5.85732200	2.49385900	H	-4.56046200	2.49473100	5.86067400
C	-6.82767800	2.95294400	-0.73389200	C	-6.81036600	-0.72792700	2.98795900
C	-7.23430100	1.75689700	-1.40946500	C	-7.21279000	-1.40725400	1.82686500
C	-7.41887400	0.57763600	-0.73235000	C	-7.41104600	-0.72796400	0.61413600
C	-7.41902500	0.57716500	0.73199300	C	-7.41104600	0.72796400	0.61413600
C	-7.21914500	1.82463700	1.40750400	C	-7.21279000	1.40725400	1.82686500
C	-6.82919400	2.95284900	0.73049400	C	-6.81036600	0.72792700	2.98795900
H	-7.21852800	1.75216700	-2.49727000	H	-7.19672300	-2.49473500	1.82279300
H	-7.20415900	1.82174500	2.49534900	H	-7.19672300	2.49473500	1.82279300
**Cyc20_R**				**Cyc20_U**			
C	1.19205600	7.73729100	1.40891000	C	-4.60369800	6.33644700	1.40714600
C	0.00052800	7.82437600	0.73248100	C	0.00000000	7.83228000	1.40714600
C	-0.00052800	7.82437600	-0.73248100	C	0.00000000	7.83231500	-1.40718500
C	1.25724200	7.72696700	-1.40891000	C	1.22474400	7.73214800	-0.72794200
H	1.18977400	7.72199000	2.49673000	C	1.22456500	7.73217600	0.72794200
H	1.25468400	7.71170900	-2.49673000	C	-5.53555300	5.53568100	0.72794200
C	-1.19205600	7.73729100	-1.40891000	C	-4.60371900	6.33647600	-1.40718500
C	-2.41836800	7.44126100	-0.73248100	H	0.00000000	7.81658500	-2.49473800
C	-2.41736300	7.44158700	0.73248100	C	2.42030700	7.44894100	-1.40714600
C	-3.58347200	6.96027300	1.40891000	C	2.42031800	7.44897400	1.40718500
C	-4.59862600	6.33036400	0.73248100	H	2.41551200	7.43418200	-2.49475200
C	-5.55892700	5.51225900	1.40891000	H	2.41545800	7.43401400	2.49473800
C	-6.32974300	4.59948000	0.73248100	C	-5.53568100	5.53555300	-0.72794200
C	-6.99023600	3.52466700	1.40891000	H	-4.59457700	6.32389300	2.49475200
C	-6.33036400	4.59862600	-0.73248100	H	0.00000000	7.81676100	2.49475200
C	-5.51225900	5.55892700	-1.40891000	H	-4.59447400	6.32375000	-2.49473800
C	-4.59948000	6.32974300	-0.73248100	C	3.55416600	6.97524300	0.72794200
C	-6.96027300	3.58347200	-1.40891000	C	3.55400400	6.97532500	-0.72794200
C	-3.52466700	6.99023600	-1.40891000	C	-7.44894100	-2.42030700	1.40714600
H	-6.97638900	3.51776800	2.49673000	C	-7.73214800	-1.22474400	0.72794200
H	-6.94655300	3.57632500	-2.49673000	C	-7.83231500	0.00000000	1.40718500
C	-1.25724200	7.72696700	1.40891000	C	-7.73217600	-1.22456500	-0.72794200
H	-5.50141900	5.54789000	-2.49673000	C	-7.83228000	0.00000000	-1.40714600
H	-5.54789000	5.50141900	2.49673000	C	-7.73217600	1.22456500	-0.72794200
H	-3.57632500	6.94655300	2.49673000	C	-7.73214800	1.22474400	0.72794200
H	-3.51776800	6.97638900	-2.49673000	C	-7.44897400	-2.42031800	-1.40718500
H	-1.18977400	7.72199000	-2.49673000	C	-7.44897400	2.42031800	-1.40718500
H	-1.25468400	7.71170900	2.49673000	C	-7.44894100	2.42030700	1.40714600
C	-7.44158700	2.41736300	-0.73248100	H	-7.43418200	-2.41551200	2.49475200
C	-7.72696700	1.25724200	-1.40891000	H	-7.43401400	-2.41545800	-2.49473800
C	-7.82437600	-0.00052800	-0.73248100	C	-6.97532500	3.55400400	0.72794200
C	-7.73729100	-1.19205600	-1.40891000	C	-6.97524300	3.55416600	-0.72794200
H	-7.72199000	-1.18977400	-2.49673000	C	-6.33644700	4.60369800	-1.40714600
C	-7.44126100	-2.41836800	-0.73248100	C	-6.33647600	4.60371900	1.40718500
C	-6.99023600	-3.52466700	-1.40891000	H	-7.81676100	0.00000000	-2.49475200
C	-6.32974300	-4.59948000	-0.73248100	H	-7.81658500	0.00000000	2.49473800
C	-5.55892700	-5.51225900	-1.40891000	H	-7.43418200	2.41551200	2.49475200
C	-4.59862600	-6.33036400	-0.73248100	H	-7.43401400	2.41545800	-2.49473800
C	-3.58347200	-6.96027300	-1.40891000	H	-6.32389300	4.59457700	-2.49475200
C	-2.41736300	-7.44158700	-0.73248100	H	-6.32375000	4.59447400	2.49473800
C	-1.25724200	-7.72696700	-1.40891000	C	6.33647600	-4.60371900	1.40718500

C	0.00052800	-7.82437600	-0.73248100	C	5.53555300	-5.53568100	0.72794200
C	-0.00052800	-7.82437600	0.73248100	C	4.60369800	-6.33644700	1.40714600
C	1.25724200	-7.72696700	1.40891000	C	5.53568100	-5.53555300	-0.72794200
C	-1.19205600	-7.73729100	1.40891000	C	4.60371900	-6.33647600	-1.40718500
C	-2.41836800	-7.44126100	0.73248100	C	3.55400400	-6.97532500	-0.72794200
C	2.41736300	-7.44158700	0.73248100	C	3.55416600	-6.97524300	0.72794200
C	1.19205600	-7.73729100	-1.40891000	C	6.97532500	-3.55400400	0.72794200
H	-1.25468400	-7.71170900	-2.49673000	C	6.33644700	-4.60369800	-1.40714600
C	-3.52466700	-6.99023600	1.40891000	H	4.59447400	-6.32375000	-2.49473800
H	-3.57632500	-6.94655300	-2.49673000	C	2.42030700	-7.44894100	-1.40714600
H	-3.51776800	-6.97638900	2.49673000	C	2.42031800	-7.44897400	1.40718500
C	2.41836800	-7.44126100	-0.73248100	H	2.41551200	-7.43418200	-2.49475200
H	1.25468400	-7.71170900	2.49673000	H	2.41545800	-7.43401400	2.49473800
H	-1.18977400	-7.72199000	2.49673000	C	6.97524300	-3.55416600	-0.72794200
H	1.18977400	-7.72199000	-2.49673000	H	6.32375000	-4.59447400	2.49473800
C	3.52466700	-6.99023600	-1.40891000	H	4.59457700	-6.32389300	2.49475200
C	4.59948000	-6.32974300	-0.73248100	H	6.32389300	-4.59457700	-2.49475200
C	4.59862600	-6.33036400	0.73248100	C	4.60369800	6.33644700	1.40714600
C	5.55892700	-5.51225900	1.40891000	C	7.44894100	2.42030700	1.40714600
C	6.32974300	-4.59948000	0.73248100	C	7.44897400	2.42031800	-1.40718500
C	6.99023600	-3.52466700	1.40891000	C	7.73217600	1.22456500	-0.72794200
C	6.33036400	-4.59862600	-0.73248100	C	7.73214800	1.22474400	0.72794200
C	6.96027300	-3.58347200	-1.40891000	C	4.60371900	6.33647600	-1.40718500
C	5.51225900	-5.55892700	-1.40891000	C	7.83228000	0.00000000	-1.40714600
C	3.58347200	-6.96027300	1.40891000	C	7.83231500	0.00000000	1.40718500
H	6.94655300	-3.57632500	-2.49673000	H	4.59457700	6.32389300	2.49475200
H	6.97638900	-3.51776800	2.49673000	H	4.59447400	6.32375000	-2.49473800
H	5.54789000	-5.50141900	2.49673000	C	7.73214800	-1.22474400	0.72794200
H	5.50141900	-5.54789000	-2.49673000	C	7.73217600	-1.22456500	-0.72794200
H	3.51776800	-6.97638900	-2.49673000	C	7.44897400	-2.42031800	-1.40718500
H	3.57632500	-6.94655300	2.49673000	C	7.44894100	-2.42030700	1.40714600
C	7.44158700	-2.41736300	-0.73248100	H	7.43401400	2.41545800	-2.49473800
C	7.72696700	-1.25724200	-1.40891000	H	7.43418200	2.41551200	2.49475200
C	7.82437600	0.00052800	-0.73248100	H	7.81658500	0.00000000	2.49473800
C	7.73729100	1.19205600	-1.40891000	H	7.81676100	0.00000000	-2.49475200
C	7.44126100	2.41836800	-0.73248100	H	7.43401400	-2.41545800	-2.49473800
C	7.44158700	2.41736300	0.73248100	H	7.43418200	-2.41551200	2.49475200
C	6.96027300	3.58347200	1.40891000	C	-4.60371900	-6.33647600	-1.40718500
C	6.33036400	4.59862600	0.73248100	C	-4.60369800	-6.33644700	1.40714600
C	5.51225900	5.55892700	1.40891000	H	-4.59447400	-6.32375000	-2.49473800
C	6.32974300	4.59948000	-0.73248100	H	-4.59457700	-6.32389300	2.49475200
C	5.55892700	5.51225900	-1.40891000	C	6.97524300	3.55416600	-0.72794200
H	5.54789000	5.50141900	-2.49673000	C	6.33644700	4.60369800	-1.40714600
C	6.99023600	3.52466700	-1.40891000	C	5.53568100	5.53555300	-0.72794200
H	6.97638900	3.51776800	-2.49673000	C	5.53555300	5.53568100	0.72794200
H	6.94655300	3.57632500	2.49673000	C	6.33647600	4.60371900	1.40718500
H	5.50141900	5.54789000	2.49673000	C	6.97532500	3.55400400	0.72794200
C	7.72696700	1.25724200	1.40891000	H	6.32389300	4.59457700	-2.49475200
H	7.71170900	1.25468400	2.49673000	H	6.32375000	4.59447400	2.49473800
H	7.72199000	1.18977400	-2.49673000	C	1.22474400	-7.73214800	-0.72794200
C	7.82437600	-0.00052800	0.73248100	C	0.00000000	-7.83231500	-1.40718500
C	7.73729100	-1.19205600	1.40891000	C	0.00000000	-7.83228000	1.40714600
C	7.44126100	-2.41836800	0.73248100	C	1.22456500	-7.73217600	0.72794200
H	7.71170900	-1.25468400	-2.49673000	H	0.00000000	-7.81658500	-2.49473800
H	7.72199000	-1.18977400	2.49673000	H	0.00000000	-7.81676100	2.49475200
C	-4.59948000	-6.32974300	0.73248100	C	-1.22474400	-7.73214800	-0.72794200
C	-5.51225900	-5.55892700	1.40891000	C	-2.42030700	-7.44894100	-1.40714600
H	-5.54789000	-5.50141900	-2.49673000	C	-3.55400400	-6.97532500	-0.72794200
H	-5.50141900	-5.54789000	2.49673000	C	-3.55416600	-6.97524300	0.72794200
C	-7.44158700	-2.41736300	0.73248100	C	-2.42031800	-7.44897400	1.40718500

C	-7.72696700	-1.25724200	1.40891000	C	-1.22456500	-7.73217600	0.72794200
H	-7.71170900	-1.25468400	2.49673000	H	-2.41551200	-7.43418200	-2.49475200
C	-6.96027300	-3.58347200	1.40891000	H	-2.41545800	-7.43401400	2.49473800
C	-6.33036400	-4.59862600	0.73248100	C	-5.53568100	-5.53555300	-0.72794200
H	-6.97638900	-3.51776800	-2.49673000	C	-6.33644700	-4.60369800	-1.40714600
H	-6.94655300	-3.57632500	2.49673000	C	-6.97524300	-3.55416600	-0.72794200
C	-7.44126100	2.41836800	0.73248100	C	-6.97532500	-3.55400400	0.72794200
C	-7.73729100	1.19205600	1.40891000	C	-6.33647600	-4.60371900	1.40718500
C	-7.82437600	0.00052800	0.73248100	C	-5.53555300	-5.53568100	0.72794200
H	-7.71170900	1.25468400	-2.49673000	H	-6.32389300	-4.59457700	-2.49475200
H	-7.72199000	1.18977400	2.49673000	H	-6.32375000	-4.59447400	2.49473800
C	4.59862600	6.33036400	-0.73248100	C	-1.22474400	7.73214800	-0.72794200
C	3.58347200	6.96027300	-1.40891000	C	-2.42030700	7.44894100	-1.40714600
C	2.41736300	7.44158700	-0.73248100	C	-3.55400400	6.97532500	-0.72794200
C	2.41836800	7.44126100	0.73248100	C	-3.55416600	6.97524300	0.72794200
C	3.52466700	6.99023600	1.40891000	C	-2.42031800	7.44897400	1.40718500
C	4.59948000	6.32974300	0.73248100	C	-1.22456500	7.73217600	0.72794200
H	3.57632500	6.94655300	-2.49673000	H	-2.41551200	7.43418200	-2.49475200
H	3.51776800	6.97638900	2.49673000	H	-2.41545800	7.43401400	2.49473800

Table S10. Cartesian coordinates of the optimized geometries of [n]-cyclacene dimer at B3LYP-D3(BJ)/6-31G(d) level of theory.

RB3LYP-D3(BJ)/6-31G(d)				UB3LYP-D3(BJ)/6-31G(d)			
Dcyc6_R				Dcyc6_U			
C	-1.50587800	1.17360000	0.72355900	C	-1.50547900	1.17380000	0.72296500
C	-2.49556800	1.83353300	1.40527100	C	-2.49621000	1.83340700	1.40481700
C	-3.70792500	2.21646100	0.72386200	C	-3.70789700	2.21550400	0.72344800
C	-3.70792500	2.21646100	-0.72386200	C	-3.70789700	2.21550400	-0.72344800
C	-2.49556800	1.83353300	-1.40527100	C	-2.49621000	1.83340700	-1.40481700
C	-1.50587800	1.17360000	-0.72355900	C	-1.50547900	1.17380000	-0.72296500
C	-4.90525100	1.97805800	1.40730500	C	-4.90659300	1.97703600	1.40711000
C	-4.90525100	1.97805800	-1.40730500	C	-4.90659300	1.97703600	-1.40711000
C	-5.88083300	1.19101800	-0.72439200	C	-5.88135100	1.19073600	-0.72436000
C	-5.88083300	1.19101800	0.72439200	C	-5.88135100	1.19073600	0.72436000
C	-6.22356700	0.00000000	1.40855900	C	-6.22906400	0.00000000	1.40872800
H	-6.15575500	0.00000000	2.49375000	H	-6.16393100	0.00000000	2.49406300
C	-5.88083300	-1.19101800	0.72439200	C	-5.88135100	-1.19073600	0.72436000
C	-5.88083300	-1.19101800	-0.72439200	C	-5.88135100	-1.19073600	-0.72436000
C	-6.22356700	0.00000000	-1.40855900	C	-6.22906400	0.00000000	-1.40872800
H	-4.87541100	1.91935200	2.49280100	H	-4.87663300	1.91832900	2.49260300
H	-2.53847300	1.74774900	2.48891000	H	-2.53909000	1.74759500	2.48845300
H	-2.53847300	1.74774900	-2.48891000	H	-2.53909000	1.74759500	-2.48845300
H	-4.87541100	1.91935200	-2.49280100	H	-4.87663300	1.91832900	-2.49260300
H	-6.15575500	0.00000000	-2.49375000	H	-6.16393100	0.00000000	-2.49406300
C	-4.90525100	-1.97805800	1.40730500	C	-4.90659300	-1.97703600	1.40711000
C	-3.70792500	-2.21646100	0.72386200	C	-3.70789700	-2.21550400	0.72344800
C	-3.70792500	-2.21646100	-0.72386200	C	-3.70789700	-2.21550400	-0.72344800
C	-4.90525100	-1.97805800	-1.40730500	C	-4.90659300	-1.97703600	-1.40711000
C	-2.49556800	-1.83353300	1.40527100	C	-2.49621000	-1.83340700	1.40481700
C	-2.49556800	-1.83353300	-1.40527100	C	-2.49621000	-1.83340700	-1.40481700
C	-1.50587800	-1.17360000	-0.72355900	C	-1.50547900	-1.17380000	-0.72296500
C	-1.50587800	-1.17360000	0.72355900	C	-1.50547900	-1.17380000	0.72296500
C	-0.78134500	0.00000000	1.39415600	C	-0.78135400	0.00000000	1.39391000
H	-1.07898000	0.00000000	2.44596800	H	-1.07938300	0.00000000	2.44559600
C	-0.78134500	0.00000000	-1.39415600	C	-0.78135400	0.00000000	-1.39391000
H	-2.53847300	-1.74774900	2.48891000	H	-2.53909000	-1.74759500	2.48845300
H	-2.53847300	-1.74774900	-2.48891000	H	-2.53909000	-1.74759500	-2.48845300
H	-1.07898000	0.00000000	-2.44596800	H	-1.07938300	0.00000000	-2.44559600
H	-4.87541100	-1.91935200	2.49280100	H	-4.87663300	-1.91832900	2.49260300
H	-4.87541100	-1.91935200	-2.49280100	H	-4.87663300	-1.91832900	-2.49260300
C	3.70792500	-2.21646100	0.72386200	C	3.70789700	-2.21550400	0.72344800
C	4.90525100	-1.97805800	1.40730500	C	4.90659300	-1.97703600	1.40711000
C	5.88083300	-1.19101800	0.72439200	C	5.88135100	-1.19073600	0.72436000
C	5.88083300	-1.19101800	-0.72439200	C	5.88135100	-1.19073600	-0.72436000
C	4.90525100	-1.97805800	-1.40730500	C	4.90659300	-1.97703600	-1.40711000
C	3.70792500	-2.21646100	-0.72386200	C	3.70789700	-2.21550400	-0.72344800
C	6.22356700	0.00000000	1.40855900	C	6.22906400	0.00000000	1.40872800
C	6.22356700	0.00000000	-1.40855900	C	6.22906400	0.00000000	-1.40872800
C	5.88083300	1.19101800	-0.72439200	C	5.88135100	1.19073600	-0.72436000
C	5.88083300	1.19101800	0.72439200	C	5.88135100	1.19073600	0.72436000
C	4.90525100	1.97805800	1.40730500	C	4.90659300	1.97703600	1.40711000
H	4.87541100	1.91935200	2.49280100	H	4.87663300	1.91832900	2.49260300
C	3.70792500	2.21646100	0.72386200	C	3.70789700	2.21550400	0.72344800
C	3.70792500	2.21646100	-0.72386200	C	3.70789700	2.21550400	-0.72344800
C	4.90525100	1.97805800	-1.40730500	C	4.90659300	1.97703600	-1.40711000
H	6.15575500	0.00000000	2.49375000	H	6.16393100	0.00000000	2.49406300

H	4.87541100	-1.91935200	2.49280100	H	4.87663300	-1.91832900	2.49260300
H	4.87541100	-1.91935200	-2.49280100	H	4.87663300	-1.91832900	-2.49260300
H	6.15575500	0.00000000	-2.49375000	H	6.16393100	0.00000000	-2.49406300
H	4.87541100	1.91935200	-2.49280100	H	4.87663300	1.91832900	-2.49260300
C	2.49556800	1.83353300	1.40527100	C	2.49621000	1.83340700	1.40481700
C	1.50587800	1.17360000	0.72355900	C	1.50547900	1.17380000	0.72296500
C	1.50587800	1.17360000	-0.72355900	C	1.50547900	1.17380000	-0.72296500
C	2.49556800	1.83353300	-1.40527100	C	2.49621000	1.83340700	-1.40481700
C	0.78134500	0.00000000	1.39415600	C	0.78135400	0.00000000	1.39391000
C	0.78134500	0.00000000	-1.39415600	C	0.78135400	0.00000000	-1.39391000
C	1.50587800	-1.17360000	-0.72355900	C	1.50547900	-1.17380000	-0.72296500
C	1.50587800	-1.17360000	0.72355900	C	1.50547900	-1.17380000	0.72296500
C	2.49556800	-1.83353300	1.40527100	C	2.49621000	-1.83340700	1.40481700
H	2.53847300	-1.74774900	2.48891000	H	2.53909000	-1.74759500	2.48845300
C	2.49556800	-1.83353300	-1.40527100	C	2.49621000	-1.83340700	-1.40481700
H	1.07898000	0.00000000	2.44596800	H	1.07938300	0.00000000	2.44559600
H	1.07898000	0.00000000	-2.44596800	H	1.07938300	0.00000000	-2.44559600
H	2.53847300	-1.74774900	-2.48891000	H	2.53909000	-1.74759500	-2.48845300
H	2.53847300	1.74774900	2.48891000	H	2.53909000	1.74759500	2.48845300
H	2.53847300	1.74774900	-2.48891000	H	2.53909000	1.74759500	-2.48845300
**Dcyc7_R**				**Dcyc7_U**			
C	-5.95538300	2.09164400	0.72732100	C	-5.95742800	2.09127800	0.72607300
C	-4.77737700	2.51092300	1.40979400	C	-4.78609900	2.50566100	1.40766500
C	-3.56524400	2.44813100	0.72671000	C	-3.56469000	2.44735100	0.72341100
C	-3.56524400	2.44813100	-0.72671000	C	-3.56469000	2.44735100	-0.72341100
C	-4.77737700	2.51092300	-1.40979400	C	-4.78609900	2.50566100	-1.40766500
C	-5.95538300	2.09164400	-0.72732100	C	-5.95742800	2.09127800	-0.72607300
C	-2.41116300	1.92005700	1.40626400	C	-2.41743600	1.91750800	1.40364300
C	-2.41116300	1.92005700	-1.40626400	C	-2.41743600	1.91750800	-1.40364300
C	-1.48632600	1.17811500	-0.72327100	C	-1.48466100	1.17905200	-0.72044100
C	-1.48632600	1.17811500	0.72327100	C	-1.48466100	1.17905200	0.72044100
C	-0.78341100	0.00000000	1.39693200	C	-0.78355500	0.00000000	1.39526000
H	-1.08580100	0.00000000	2.44712500	H	-1.08640900	0.00000000	2.44537100
C	-0.78341100	0.00000000	-1.39693200	C	-0.78355500	0.00000000	-1.39526000
H	-2.45273100	1.85517300	2.49132500	H	-2.46031800	1.85044100	2.48856300
H	-4.76705500	2.45100000	2.49549300	H	-4.77509700	2.44589800	2.49340000
H	-4.76705500	2.45100000	-2.49549300	H	-4.77509700	2.44589800	-2.49340000
H	-2.45273100	1.85517300	-2.49132500	H	-2.46031800	1.85044100	-2.48856300
H	-1.08580100	0.00000000	-2.44712500	H	-1.08640900	0.00000000	-2.44537100
C	-1.48632600	-1.17811500	0.72327100	C	-1.48466100	-1.17905200	0.72044100
C	-2.41116300	-1.92005700	1.40626400	C	-2.41743600	-1.91750800	1.40364300
C	-3.56524400	-2.44813100	0.72671000	C	-3.56469000	-2.44735100	0.72341100
C	-3.56524400	-2.44813100	-0.72671000	C	-3.56469000	-2.44735100	-0.72341100
C	-2.41116300	-1.92005700	-1.40626400	C	-2.41743600	-1.91750800	-1.40364300
C	-1.48632600	-1.17811500	-0.72327100	C	-1.48466100	-1.17905200	-0.72044100
C	-4.77737700	-2.51092300	1.40979400	C	-4.78609900	-2.50566100	1.40766500
C	-4.77737700	-2.51092300	-1.40979400	C	-4.78609900	-2.50566100	-1.40766500
C	-5.95538300	-2.09164400	-0.72732100	C	-5.95742800	-2.09127800	-0.72607300
C	-5.95538300	-2.09164400	0.72732100	C	-5.95742800	-2.09127800	0.72607300
C	-6.80258600	-1.19994200	1.41118400	C	-6.82256900	-1.20365100	1.41101500
H	-6.74813700	-1.17075200	2.49658000	H	-6.77709300	-1.18014500	2.49694400
C	-6.80258600	-1.19994200	-1.41118400	C	-6.82256900	-1.20365100	-1.41101500
H	-4.76705500	-2.45100000	2.49549300	H	-4.77509700	-2.44589800	2.49340000
H	-2.45273100	-1.85517300	2.49132500	H	-2.46031800	-1.85044100	2.48856300
H	-2.45273100	-1.85517300	-2.49132500	H	-2.46031800	-1.85044100	-2.48856300
H	-4.76705500	-2.45100000	-2.49549300	H	-4.77509700	-2.44589800	-2.49340000
H	-6.74813700	-1.17075200	-2.49658000	H	-6.77709300	-1.18014500	-2.49694400
C	-7.11763900	0.00000000	0.72786800	C	-7.12186600	0.00000000	0.72856000

C	-6.80258600	1.19994200	1.41118400	C	-6.82256900	1.20365100	1.41101500
C	-6.80258600	1.19994200	-1.41118400	C	-6.82256900	1.20365100	-1.41101500
C	-7.11763900	0.00000000	-0.72786800	C	-7.12186600	0.00000000	-0.72856000
H	-6.74813700	1.17075200	2.49658000	H	-6.77709300	1.18014500	2.49694400
H	-6.74813700	1.17075200	-2.49658000	H	-6.77709300	1.18014500	-2.49694400
C	1.48632600	1.17811500	0.72327100	C	1.48466100	1.17905200	0.72044100
C	2.41116300	1.92005700	1.40626400	C	2.41743600	1.91750800	1.40364300
C	3.56524400	2.44813100	0.72671000	C	3.56469000	2.44735100	0.72341100
C	3.56524400	2.44813100	-0.72671000	C	3.56469000	2.44735100	-0.72341100
C	2.41116300	1.92005700	-1.40626400	C	2.41743600	1.91750800	-1.40364300
C	1.48632600	1.17811500	-0.72327100	C	1.48466100	1.17905200	-0.72044100
C	4.77737700	2.51092300	1.40979400	C	4.78609900	2.50566100	1.40766500
C	4.77737700	2.51092300	-1.40979400	C	4.78609900	2.50566100	-1.40766500
C	5.95538300	2.09164400	-0.72732100	C	5.95742800	2.09127800	-0.72607300
C	5.95538300	2.09164400	0.72732100	C	5.95742800	2.09127800	0.72607300
C	6.80258600	1.19994200	1.41118400	C	6.82256900	1.20365100	1.41101500
H	6.74813700	1.17075200	2.49658000	H	6.77709300	1.18014500	2.49694400
C	6.80258600	1.19994200	-1.41118400	C	6.82256900	1.20365100	-1.41101500
H	4.76705500	2.45100000	2.49549300	H	4.77509700	2.44589800	2.49340000
H	2.45273100	1.85517300	2.49132500	H	2.46031800	1.85044100	2.48856300
H	2.45273100	1.85517300	-2.49132500	H	2.46031800	1.85044100	-2.48856300
H	4.76705500	2.45100000	-2.49549300	H	4.77509700	2.44589800	-2.49340000
H	6.74813700	1.17075200	-2.49658000	H	6.77709300	1.18014500	-2.49694400
C	7.11763900	0.00000000	0.72786800	C	7.12186600	0.00000000	0.72856000
C	6.80258600	-1.19994200	1.41118400	C	6.82256900	-1.20365100	1.41101500
C	5.95538300	-2.09164400	0.72732100	C	5.95742800	-2.09127800	0.72607300
C	5.95538300	-2.09164400	-0.72732100	C	5.95742800	-2.09127800	-0.72607300
C	6.80258600	-1.19994200	-1.41118400	C	6.82256900	-1.20365100	-1.41101500
C	7.11763900	0.00000000	-0.72786800	C	7.12186600	0.00000000	-0.72856000
C	4.77737700	-2.51092300	1.40979400	C	4.78609900	-2.50566100	1.40766500
C	4.77737700	-2.51092300	-1.40979400	C	4.78609900	-2.50566100	-1.40766500
C	3.56524400	-2.44813100	-0.72671000	C	3.56469000	-2.44735100	-0.72341100
C	3.56524400	-2.44813100	0.72671000	C	3.56469000	-2.44735100	0.72341100
C	2.41116300	-1.92005700	1.40626400	C	2.41743600	-1.91750800	1.40364300
H	2.45273100	-1.85517300	2.49132500	H	2.46031800	-1.85044100	2.48856300
C	2.41116300	-1.92005700	-1.40626400	C	2.41743600	-1.91750800	-1.40364300
H	4.76705500	-2.45100000	2.49549300	H	4.77509700	-2.44589800	2.49340000
H	6.74813700	-1.17075200	2.49658000	H	6.77709300	-1.18014500	2.49694400
H	6.74813700	-1.17075200	-2.49658000	H	6.77709300	-1.18014500	-2.49694400
H	4.76705500	-2.45100000	-2.49549300	H	4.77509700	-2.44589800	-2.49340000
H	2.45273100	-1.85517300	-2.49132500	H	2.46031800	-1.85044100	-2.48856300
C	1.48632600	-1.17811500	0.72327100	C	1.48466100	-1.17905200	0.72044100
C	0.78341100	0.00000000	1.39693200	C	0.78355500	0.00000000	1.39526000
C	0.78341100	0.00000000	-1.39693200	C	0.78355500	0.00000000	-1.39526000
C	1.48632600	-1.17811500	-0.72327100	C	1.48466100	-1.17905200	-0.72044100
H	1.08580100	0.00000000	2.44712500	H	1.08640900	0.00000000	2.44537100
H	1.08580100	0.00000000	-2.44712500	H	1.08640900	0.00000000	-2.44537100

**Dcyc8_R**				**Dcyc8_U**			
C	-3.44404600	2.62332600	0.72679000	C	-3.44377600	2.61593900	0.72183100
C	-4.62730800	2.87885700	1.40924600	C	-4.64273900	2.87000200	1.40575200
C	-5.87093000	2.74992700	0.72835400	C	-5.87241100	2.74604700	0.72538300
C	-5.87093000	2.74992700	-0.72835400	C	-5.87241100	2.74604700	-0.72538300
C	-4.62730800	2.87885700	-1.40924600	C	-4.64273900	2.87000200	-1.40575200
C	-3.44404600	2.62332600	-0.72679000	C	-3.44377600	2.61593900	-0.72183100
C	-6.94981400	2.17152700	1.41156800	C	-6.97446800	2.17365500	1.40984300
C	-6.94981400	2.17152700	-1.41156800	C	-6.97446800	2.17365500	-1.40984300
C	-7.72573000	1.20322000	-0.72874500	C	-7.73567200	1.20409400	-0.72870200
C	-7.72573000	1.20322000	0.72874500	C	-7.73567200	1.20409400	0.72870200

C	-7.99866500	0.00000000	1.41199700	C	-8.03999500	0.00000000	1.41115700
H	-7.94240700	0.00000000	2.49780100	H	-8.00572200	0.00000000	2.49783800
C	-7.99866500	0.00000000	-1.41199700	C	-8.03999500	0.00000000	-1.41115700
H	-6.91332000	2.12938900	2.49747700	H	-6.94487100	2.14135100	2.49627800
H	-4.62884800	2.82825800	2.49566100	H	-4.64347100	2.81800900	2.49211600
H	-4.62884800	2.82825800	-2.49566100	H	-4.64347100	2.81800900	-2.49211600
H	-6.91332000	2.12938900	-2.49747700	H	-6.94487100	2.14135100	-2.49627800
H	-7.94240700	0.00000000	-2.49780100	H	-8.00572200	0.00000000	-2.49783800
C	-7.72573000	-1.20322000	0.72874500	C	-7.73567200	-1.20409400	0.72870200
C	-6.94981400	-2.17152700	1.41156800	C	-6.97446800	-2.17365500	1.40984300
C	-5.87093000	-2.74992700	0.72835400	C	-5.87241100	-2.74604700	0.72538300
C	-5.87093000	-2.74992700	-0.72835400	C	-5.87241100	-2.74604700	-0.72538300
C	-6.94981400	-2.17152700	-1.41156800	C	-6.97446800	-2.17365500	-1.40984300
C	-7.72573000	-1.20322000	-0.72874500	C	-7.73567200	-1.20409400	-0.72870200
C	-4.62730800	-2.87885700	1.40924600	C	-4.64273900	-2.87000200	1.40575200
C	-4.62730800	-2.87885700	-1.40924600	C	-4.64273900	-2.87000200	-1.40575200
C	-3.44404600	-2.62332600	-0.72679000	C	-3.44377600	-2.61593900	-0.72183100
C	-3.44404600	-2.62332600	0.72679000	C	-3.44377600	-2.61593900	0.72183100
C	-2.35422400	-1.97362700	1.40685100	C	-2.35987800	-1.97488800	1.40266400
H	-2.39932400	-1.91486700	2.49221300	H	-2.40353900	-1.91743700	2.48816700
C	-2.35422400	-1.97362700	-1.40685100	C	-2.35987800	-1.97488800	-1.40266400
H	-4.62884800	-2.82825800	2.49566100	H	-4.64347100	-2.81800900	2.49211600
H	-6.91332000	-2.12938900	2.49747700	H	-6.94487100	-2.14135100	2.49627800
H	-6.91332000	-2.12938900	-2.49747700	H	-6.94487100	-2.14135100	-2.49627800
H	-4.62884800	-2.82825800	-2.49566100	H	-4.64347100	-2.81800900	-2.49211600
H	-2.39932400	-1.91486700	-2.49221300	H	-2.40353900	-1.91743700	-2.48816700
C	-1.47301800	-1.18439200	0.72416100	C	-1.46981000	-1.18524900	0.71878900
C	-1.47301800	-1.18439200	-0.72416100	C	-1.46981000	-1.18524900	-0.71878900
H	-1.08797400	0.00000000	2.44721700	H	-1.09049300	0.00000000	2.44450300
C	-0.78587600	0.00000000	1.39680700	C	-0.78617800	0.00000000	1.39472900
C	-0.78587600	0.00000000	-1.39680700	C	-0.78617800	0.00000000	-1.39472900
H	-1.08797400	0.00000000	-2.44721700	H	-1.09049300	0.00000000	-2.44450300
C	-1.47301800	1.18439200	0.72416100	C	-1.46981000	1.18524900	0.71878900
C	-2.35422400	1.97362700	1.40685100	C	-2.35987800	1.97488800	1.40266400
C	-2.35422400	1.97362700	-1.40685100	C	-2.35987800	1.97488800	-1.40266400
C	-1.47301800	1.18439200	-0.72416100	C	-1.46981000	1.18524900	-0.71878900
H	-2.39932400	1.91486700	2.49221300	H	-2.40353900	1.91743700	2.48816700
H	-2.39932400	1.91486700	-2.49221300	H	-2.40353900	1.91743700	-2.48816700
C	7.72573000	1.20322000	0.72874500	C	7.73567200	1.20409400	0.72870200
C	6.94981400	2.17152700	1.41156800	C	6.97446800	2.17365500	1.40984300
C	5.87093000	2.74992700	0.72835400	C	5.87241100	2.74604700	0.72538300
C	5.87093000	2.74992700	-0.72835400	C	5.87241100	2.74604700	-0.72538300
C	6.94981400	2.17152700	-1.41156800	C	6.97446800	2.17365500	-1.40984300
C	7.72573000	1.20322000	-0.72874500	C	7.73567200	1.20409400	-0.72870200
C	4.62730800	2.87885700	1.40924600	C	4.64273900	2.87000200	1.40575200
C	4.62730800	2.87885700	-1.40924600	C	4.64273900	2.87000200	-1.40575200
C	3.44404600	2.62332600	-0.72679000	C	3.44377600	2.61593900	-0.72183100
C	3.44404600	2.62332600	0.72679000	C	3.44377600	2.61593900	0.72183100
C	2.35422400	1.97362700	1.40685100	C	2.35987800	1.97488800	1.40266400
H	2.39932400	1.91486700	2.49221300	H	2.40353900	1.91743700	2.48816700
C	2.35422400	1.97362700	-1.40685100	C	2.35987800	1.97488800	-1.40266400
H	4.62884800	2.82825800	2.49566100	H	4.64347100	2.81800900	2.49211600
H	6.91332000	2.12938900	2.49747700	H	6.94487100	2.14135100	2.49627800
H	6.91332000	2.12938900	-2.49747700	H	6.94487100	2.14135100	-2.49627800
H	4.62884800	2.82825800	-2.49566100	H	4.64347100	2.81800900	-2.49211600
H	2.39932400	1.91486700	-2.49221300	H	2.40353900	1.91743700	-2.48816700
C	1.47301800	1.18439200	0.72416100	C	1.46981000	1.18524900	0.71878900
C	0.78587600	0.00000000	1.39680700	C	0.78617800	0.00000000	1.39472900
C	1.47301800	-1.18439200	0.72416100	C	1.46981000	-1.18524900	0.71878900
C	1.47301800	-1.18439200	-0.72416100	C	1.46981000	-1.18524900	-0.71878900

C	0.78587600	0.00000000	-1.39680700	C	0.78617800	0.00000000	-1.39472900
C	1.47301800	1.18439200	-0.72416100	C	1.46981000	1.18524900	-0.71878900
C	2.35422400	-1.97362700	1.40685100	C	2.35987800	-1.97488800	1.40266400
C	2.35422400	-1.97362700	-1.40685100	C	2.35987800	-1.97488800	-1.40266400
C	3.44404600	-2.62332600	-0.72679000	C	3.44377600	-2.61593900	-0.72183100
C	3.44404600	-2.62332600	0.72679000	C	3.44377600	-2.61593900	0.72183100
C	4.62730800	-2.87885700	1.40924600	C	4.64273900	-2.87000200	1.40575200
H	4.62884800	-2.82825800	2.49566100	H	4.64347100	-2.81800900	2.49211600
C	4.62730800	-2.87885700	-1.40924600	C	4.64273900	-2.87000200	-1.40575200
H	2.39932400	-1.91486700	2.49221300	H	2.40353900	-1.91743700	2.48816700
H	1.08797400	0.00000000	2.44721700	H	1.09049300	0.00000000	2.44450300
H	1.08797400	0.00000000	-2.44721700	H	1.09049300	0.00000000	-2.44450300
H	2.39932400	-1.91486700	-2.49221300	H	2.40353900	-1.91743700	-2.48816700
H	4.62884800	-2.82825800	-2.49566100	H	4.64347100	-2.81800900	-2.49211600
C	5.87093000	-2.74992700	0.72835400	C	5.87241100	-2.74604700	0.72538300
C	5.87093000	-2.74992700	-0.72835400	C	5.87241100	-2.74604700	-0.72538300
H	6.91332000	-2.12938900	2.49747700	H	6.94487100	-2.14135100	2.49627800
C	6.94981400	-2.17152700	1.41156800	C	6.97446800	-2.17365500	1.40984300
C	6.94981400	-2.17152700	-1.41156800	C	6.97446800	-2.17365500	-1.40984300
H	6.91332000	-2.12938900	-2.49747700	H	6.94487100	-2.14135100	-2.49627800
C	7.72573000	-1.20322000	0.72874500	C	7.73567200	-1.20409400	0.72870200
C	7.99866500	0.00000000	1.41199700	C	8.03999500	0.00000000	1.41115700
C	7.99866500	0.00000000	-1.41199700	C	8.03999500	0.00000000	-1.41115700
C	7.72573000	-1.20322000	-0.72874500	C	7.73567200	-1.20409400	-0.72870200
H	7.94240700	0.00000000	2.49780100	H	8.00572200	0.00000000	2.49783800
H	7.94240700	0.00000000	-2.49780100	H	8.00572200	0.00000000	-2.49783800
**Dcyc9_R**				**Dcyc9_U**			
C	-5.72939400	3.22737700	0.72917100	C	-5.72855300	3.22485900	0.72553000
C	-4.48299600	3.14229000	1.40971800	C	-4.49721500	3.13818900	1.40546900
C	-3.34242100	2.74274600	0.72764700	C	-3.33947600	2.74194100	0.72213700
C	-3.34242100	2.74274600	-0.72764700	C	-3.33947600	2.74194100	-0.72213700
C	-4.48299600	3.14229000	-1.40971800	C	-4.49721500	3.13818900	-1.40546900
C	-5.72939400	3.22737700	-0.72917100	C	-5.72855300	3.22485900	-0.72553000
C	-2.30152500	2.01868400	1.40721900	C	-2.30878300	2.01911900	1.40313200
C	-2.30152500	2.01868400	-1.40721900	C	-2.30878300	2.01911900	-1.40313200
C	-1.46149100	1.18785700	-0.72447100	C	-1.45881900	1.18936800	-0.71921900
C	-1.46149100	1.18785700	0.72447100	C	-1.45881900	1.18936800	0.71921900
C	-0.78697100	0.00000000	1.39832300	C	-0.78748900	0.00000000	1.39565200
H	-1.09169100	0.00000000	2.44801100	H	-1.09303600	0.00000000	2.44508700
C	-0.78697100	0.00000000	-1.39832300	C	-0.78748900	0.00000000	-1.39565200
H	-2.34308500	1.97091800	2.49325600	H	-2.35214800	1.96916600	2.48900200
H	-4.49110500	3.09852700	2.49634800	H	-4.50470700	3.09330600	2.49215700
H	-4.49110500	3.09852700	-2.49634800	H	-4.50470700	3.09330600	-2.49215700
H	-2.34308500	1.97091800	-2.49325600	H	-2.35214800	1.96916600	-2.48900200
H	-1.09169100	0.00000000	-2.44801100	H	-1.09303600	0.00000000	-2.44508700
C	-1.46149100	-1.18785700	0.72447100	C	-1.45881900	-1.18936800	0.71921900
C	-2.30152500	-2.01868400	1.40721900	C	-2.30878300	-2.01911900	1.40313200
C	-3.34242100	-2.74274600	0.72764700	C	-3.33947600	-2.74194100	0.72213700
C	-3.34242100	-2.74274600	-0.72764700	C	-3.33947600	-2.74194100	-0.72213700
C	-2.30152500	-2.01868400	-1.40721900	C	-2.30878300	-2.01911900	-1.40313200
C	-1.46149100	-1.18785700	-0.72447100	C	-1.45881900	-1.18936800	-0.71921900
C	-4.48299600	-3.14229000	1.40971800	C	-4.49721500	-3.13818900	1.40546900
C	-4.48299600	-3.14229000	-1.40971800	C	-4.49721500	-3.13818900	-1.40546900
C	-5.72939400	-3.22737700	-0.72917100	C	-5.72855300	-3.22485900	-0.72553000
C	-5.72939400	-3.22737700	0.72917100	C	-5.72855300	-3.22485900	0.72553000
C	-6.91246400	-2.92389400	1.41178500	C	-6.93164400	-2.92181400	1.40914300
H	-6.89160200	-2.88054500	2.49799700	H	-6.91321000	-2.88559000	2.49576000
C	-6.91246400	-2.92389400	-1.41178500	C	-6.93164400	-2.92181400	-1.40914300

H	-4.49110500	-3.09852700	2.49634800	H	-4.50470700	-3.09330600	2.49215700
H	-2.34308500	-1.97091800	2.49325600	H	-2.35214800	-1.96916600	2.48900200
H	-2.34308500	-1.97091800	-2.49325600	H	-2.35214800	-1.96916600	-2.48900200
H	-4.49110500	-3.09852700	-2.49634800	H	-4.50470700	-3.09330600	-2.49215700
H	-6.89160200	-2.88054500	-2.49799700	H	-6.91321000	-2.88559000	-2.49576000
C	-7.93791400	-2.22585700	0.73045100	C	-7.94496300	-2.22810100	0.72891400
C	-8.63117800	-1.21048000	1.41288500	C	-8.66557000	-1.21468200	1.41115200
C	-8.87637000	0.00000000	0.73048400	C	-8.88919200	0.00000000	0.73033500
C	-8.87637000	0.00000000	-0.73048400	C	-8.88919200	0.00000000	-0.73033500
C	-8.63117800	-1.21048000	-1.41288500	C	-8.66557000	-1.21468200	-1.41115200
C	-7.93791400	-2.22585700	-0.73045100	C	-7.94496300	-2.22810100	-0.72891400
C	-8.63117800	1.21048000	1.41288500	C	-8.66557000	1.21468200	1.41115200
C	-8.63117800	1.21048000	-1.41288500	C	-8.66557000	1.21468200	-1.41115200
C	-7.93791400	2.22585700	-0.73045100	C	-7.94496300	2.22810100	-0.72891400
C	-7.93791400	2.22585700	0.73045100	C	-7.94496300	2.22810100	0.72891400
C	-6.91246400	2.92389400	1.41178500	C	-6.93164400	2.92181400	1.40914300
H	-6.89160200	2.88054500	2.49799700	H	-6.91321000	2.88559000	2.49576000
C	-6.91246400	2.92389400	-1.41178500	C	-6.93164400	2.92181400	-1.40914300
H	-8.58413900	1.19125600	2.49891600	H	-8.63605300	1.20311400	2.49791300
H	-8.58413900	-1.19125600	2.49891600	H	-8.63605300	-1.20311400	2.49791300
H	-8.58413900	-1.19125600	-2.49891600	H	-8.63605300	-1.20311400	-2.49791300
H	-8.58413900	1.19125600	-2.49891600	H	-8.63605300	1.20311400	-2.49791300
H	-6.89160200	2.88054500	-2.49799700	H	-6.91321000	2.88559000	-2.49576000
C	3.34242100	2.74274600	0.72764700	C	3.33947600	2.74194100	0.72213700
C	4.48299600	3.14229000	1.40971800	C	4.49721500	3.13818900	1.40546900
C	5.72939400	3.22737700	0.72917100	C	5.72855300	3.22485900	0.72553000
C	5.72939400	3.22737700	-0.72917100	C	5.72855300	3.22485900	-0.72553000
C	4.48299600	3.14229000	-1.40971800	C	4.49721500	3.13818900	-1.40546900
C	3.34242100	2.74274600	-0.72764700	C	3.33947600	2.74194100	-0.72213700
C	6.91246400	2.92389400	1.41178500	C	6.93164400	2.92181400	1.40914300
C	6.91246400	2.92389400	-1.41178500	C	6.93164400	2.92181400	-1.40914300
C	7.93791400	2.22585700	-0.73045100	C	7.94496300	2.22810100	-0.72891400
C	7.93791400	2.22585700	0.73045100	C	7.94496300	2.22810100	0.72891400
C	8.63117800	1.21048000	1.41288500	C	8.66557000	1.21468200	1.41115200
H	8.58413900	1.19125600	2.49891600	H	8.63605300	1.20311400	2.49791300
C	8.63117800	1.21048000	-1.41288500	C	8.66557000	1.21468200	-1.41115200
H	6.89160200	2.88054500	2.49799700	H	6.91321000	2.88559000	2.49576000
H	4.49110500	3.09852700	2.49634800	H	4.50470700	3.09330600	2.49215700
H	4.49110500	3.09852700	-2.49634800	H	4.50470700	3.09330600	-2.49215700
H	6.89160200	2.88054500	-2.49799700	H	6.91321000	2.88559000	-2.49576000
H	8.58413900	1.19125600	-2.49891600	H	8.63605300	1.20311400	-2.49791300
C	8.87637000	0.00000000	0.73048400	C	8.88919200	0.00000000	0.73033500
C	8.63117800	-1.21048000	1.41288500	C	8.66557000	-1.21468200	1.41115200
C	7.93791400	-2.22585700	0.73045100	C	7.94496300	-2.22810100	0.72891400
C	7.93791400	-2.22585700	-0.73045100	C	7.94496300	-2.22810100	-0.72891400
C	8.63117800	-1.21048000	-1.41288500	C	8.66557000	-1.21468200	-1.41115200
C	8.87637000	0.00000000	-0.73048400	C	8.88919200	0.00000000	-0.73033500
C	6.91246400	-2.92389400	1.41178500	C	6.93164400	-2.92181400	1.40914300
C	6.91246400	-2.92389400	-1.41178500	C	6.93164400	-2.92181400	-1.40914300
C	5.72939400	-3.22737700	-0.72917100	C	5.72855300	-3.22485900	-0.72553000
C	5.72939400	-3.22737700	0.72917100	C	5.72855300	-3.22485900	0.72553000
C	4.48299600	-3.14229000	1.40971800	C	4.49721500	-3.13818900	1.40546900
H	4.49110500	-3.09852700	2.49634800	H	4.50470700	-3.09330600	2.49215700
C	4.48299600	-3.14229000	-1.40971800	C	4.49721500	-3.13818900	-1.40546900
H	6.89160200	-2.88054500	2.49799700	H	6.91321000	-2.88559000	2.49576000
H	8.58413900	-1.19125600	2.49891600	H	8.63605300	-1.20311400	2.49791300
H	8.58413900	-1.19125600	-2.49891600	H	8.63605300	-1.20311400	-2.49791300
H	6.89160200	-2.88054500	-2.49799700	H	6.91321000	-2.88559000	-2.49576000
H	4.49110500	-3.09852700	-2.49634800	H	4.50470700	-3.09330600	-2.49215700
C	3.34242100	-2.74274600	0.72764700	C	3.33947600	-2.74194100	0.72213700

C	2.30152500	-2.01868400	1.40721900	C	2.30878300	-2.01911900	1.40313200
C	1.46149100	-1.18785700	0.72447100	C	1.45881900	-1.18936800	0.71921900
C	1.46149100	-1.18785700	-0.72447100	C	1.45881900	-1.18936800	-0.71921900
C	2.30152500	-2.01868400	-1.40721900	C	2.30878300	-2.01911900	-1.40313200
C	3.34242100	-2.74274600	-0.72764700	C	3.33947600	-2.74194100	-0.72213700
C	0.78697100	0.00000000	1.39832300	C	0.78748900	0.00000000	1.39565200
C	0.78697100	0.00000000	-1.39832300	C	0.78748900	0.00000000	-1.39565200
C	1.46149100	1.18785700	-0.72447100	C	1.45881900	1.18936800	-0.71921900
C	1.46149100	1.18785700	0.72447100	C	1.45881900	1.18936800	0.71921900
C	2.30152500	2.01868400	1.40721900	C	2.30878300	2.01911900	1.40313200
H	2.34308500	1.97091800	2.49325600	H	2.35214800	1.96916600	2.48900200
C	2.30152500	2.01868400	-1.40721900	C	2.30878300	2.01911900	-1.40313200
H	1.09169100	0.00000000	2.44801100	H	1.09303600	0.00000000	2.44508700
H	2.34308500	-1.97091800	2.49325600	H	2.35214800	-1.96916600	2.48900200
H	2.34308500	-1.97091800	-2.49325600	H	2.35214800	-1.96916600	-2.48900200
H	1.09169100	0.00000000	-2.44801100	H	1.09303600	0.00000000	-2.44508700
H	2.34308500	1.97091800	-2.49325600	H	2.35214800	1.96916600	-2.48900200
**Dcyc10_R**				**Dcyc10_U**			
C	1.45277300	1.19225200	0.72434200	C	1.44994800	1.19277000	0.71901500
C	1.45277300	1.19225200	-0.72434200	C	1.44994800	1.19277000	-0.71901500
C	0.78891900	0.00000000	1.39832600	C	0.78933900	0.00000000	1.39618900
C	0.78891900	0.00000000	-1.39832600	C	0.78933900	0.00000000	-1.39618900
H	1.09347800	0.00000000	2.44802600	H	1.09551800	0.00000000	2.44543800
H	1.09347800	0.00000000	-2.44802600	H	1.09551800	0.00000000	-2.44543800
C	3.25808300	-2.83594900	0.72805200	C	3.25650600	-2.83087300	0.72243600
C	3.25808300	-2.83594900	-0.72805200	C	3.25650600	-2.83087300	-0.72243600
C	8.90210900	-2.27146200	1.41273700	C	8.93162000	-2.27573600	1.41021100
C	7.95519400	-3.05106000	0.73067700	C	7.95990500	-3.05237100	0.72859800
C	6.79653900	-3.49446400	1.41150100	C	6.81634900	-3.49318800	1.40835300
C	7.95519400	-3.05106000	-0.73067700	C	7.95990500	-3.05237100	-0.72859800
C	6.79653900	-3.49446400	-1.41150100	C	6.81634900	-3.49318800	-1.40835300
C	5.58068200	-3.58522300	-0.72974000	C	5.57951100	-3.58032100	-0.72562200
C	5.58068200	-3.58522300	0.72974000	C	5.57951100	-3.58032100	0.72562200
C	9.53666600	-1.21121000	0.73102300	C	9.55141700	-1.21383900	0.73008100
C	8.90210900	-2.27146200	-1.41273700	C	8.93162000	-2.27573600	-1.41021100
H	6.78644100	-3.45373500	-2.49813100	H	6.80689900	-3.45826100	-2.49521500
C	4.35583000	-3.33830100	-1.40932700	C	4.37103000	-3.33397100	-1.40526300
C	4.35583000	-3.33830100	1.40932700	C	4.37103000	-3.33397100	1.40526300
H	4.36686100	-3.30206700	-2.49631900	H	4.38200800	-3.29554400	-2.49219300
H	4.36686100	-3.30206700	2.49631900	H	4.38200800	-3.29554400	2.49219300
C	9.53666600	-1.21121000	-0.73102300	C	9.55141700	-1.21383900	-0.73008100
H	8.86723700	-2.24218100	2.49909100	H	8.90829700	-2.25694100	2.49714500
H	6.78644100	-3.45373500	2.49813100	H	6.80689900	-3.45826100	2.49521500
H	8.86723700	-2.24218100	-2.49909100	H	8.90829700	-2.25694100	-2.49714500
C	2.26309000	-2.05035400	1.40762700	C	2.26754100	-2.05333600	1.40333000
C	1.45277300	-1.19225200	0.72434200	C	1.44994800	-1.19277000	0.71901500
C	1.45277300	-1.19225200	-0.72434200	C	1.44994800	-1.19277000	-0.71901500
C	2.26309000	-2.05035400	-1.40762700	C	2.26754100	-2.05333600	-1.40333000
H	2.30475400	-2.00680600	2.49380000	H	2.30770300	-2.01121000	2.48963500
H	2.30475400	-2.00680600	-2.49380000	H	2.30770300	-2.01121000	-2.48963500
C	9.75935400	0.00000000	1.41319600	C	9.79698600	0.00000000	1.41061900
C	9.75935400	0.00000000	-1.41319600	C	9.79698600	0.00000000	-1.41061900
H	9.71216700	0.00000000	2.49943000	H	9.77062100	0.00000000	2.49760700
H	9.71216700	0.00000000	-2.49943000	H	9.77062100	0.00000000	-2.49760700
C	7.95519400	3.05106000	0.73067700	C	7.95990500	3.05237100	0.72859800
C	7.95519400	3.05106000	-0.73067700	C	7.95990500	3.05237100	-0.72859800
C	2.26309000	2.05035400	1.40762700	C	2.26754100	2.05333600	1.40333000
C	3.25808300	2.83594900	0.72805200	C	3.25650600	2.83087300	0.72243600

C	4.35583000	3.33830100	1.40932700	C	4.37103000	3.33397100	1.40526300
C	3.25808300	2.83594900	-0.72805200	C	3.25650600	2.83087300	-0.72243600
C	4.35583000	3.33830100	-1.40932700	C	4.37103000	3.33397100	-1.40526300
C	5.58068200	3.58522300	-0.72974000	C	5.57951100	3.58032100	-0.72562200
C	5.58068200	3.58522300	0.72974000	C	5.57951100	3.58032100	0.72562200
C	2.26309000	2.05035400	-1.40762700	C	2.26754100	2.05333600	-1.40333000
H	4.36686100	3.30206700	-2.49631900	H	4.38200800	3.29554400	-2.49219300
C	6.79653900	3.49446400	-1.41150100	C	6.81634900	3.49318800	-1.40835300
C	6.79653900	3.49446400	1.41150100	C	6.81634900	3.49318800	1.40835300
H	6.78644100	3.45373500	-2.49813100	H	6.80689900	3.45826100	-2.49521500
H	6.78644100	3.45373500	2.49813100	H	6.80689900	3.45826100	2.49521500
H	2.30475400	2.00680600	2.49380000	H	2.30770300	2.01121000	2.48963500
H	4.36686100	3.30206700	2.49631900	H	4.38200800	3.29554400	2.49219300
H	2.30475400	2.00680600	-2.49380000	H	2.30770300	2.01121000	-2.48963500
C	8.90210900	2.27146200	1.41273700	C	8.93162000	2.27573600	1.41021100
C	9.53666600	1.21121000	0.73102300	C	9.55141700	1.21383900	0.73008100
C	9.53666600	1.21121000	-0.73102300	C	9.55141700	1.21383900	-0.73008100
C	8.90210900	2.27146200	-1.41273700	C	8.93162000	2.27573600	-1.41021100
H	8.86723700	2.24218100	2.49909100	H	8.90829700	2.25694100	2.49714500
H	8.86723700	2.24218100	-2.49909100	H	8.90829700	2.25694100	-2.49714500
C	-9.53666600	1.21121000	0.73102300	C	-9.55141700	1.21383900	0.73008100
C	-9.53666600	1.21121000	-0.73102300	C	-9.55141700	1.21383900	-0.73008100
C	-9.75935400	0.00000000	1.41319600	C	-9.79698600	0.00000000	1.41061900
C	-9.75935400	0.00000000	-1.41319600	C	-9.79698600	0.00000000	-1.41061900
H	-9.71216700	0.00000000	2.49943000	H	-9.77062100	0.00000000	2.49760700
H	-9.71216700	0.00000000	-2.49943000	H	-9.77062100	0.00000000	-2.49760700
C	-7.95519400	-3.05106000	0.73067700	C	-7.95990500	-3.05237100	0.72859800
C	-7.95519400	-3.05106000	-0.73067700	C	-7.95990500	-3.05237100	-0.72859800
C	-2.26309000	-2.05035400	1.40762700	C	-2.26754100	-2.05333600	1.40333000
C	-3.25808300	-2.83594900	0.72805200	C	-3.25650600	-2.83087300	0.72243600
C	-4.35583000	-3.33830100	1.40932700	C	-4.37103000	-3.33397100	1.40526300
C	-3.25808300	-2.83594900	-0.72805200	C	-3.25650600	-2.83087300	-0.72243600
C	-4.35583000	-3.33830100	-1.40932700	C	-4.37103000	-3.33397100	-1.40526300
C	-5.58068200	-3.58522300	-0.72974000	C	-5.57951100	-3.58032100	-0.72562200
C	-5.58068200	-3.58522300	0.72974000	C	-5.57951100	-3.58032100	0.72562200
C	-1.45277300	-1.19225200	0.72434200	C	-1.44994800	-1.19277000	0.71901500
C	-2.26309000	-2.05035400	-1.40762700	C	-2.26754100	-2.05333600	-1.40333000
H	-4.36686100	-3.30206700	-2.49631900	H	-4.38200800	-3.29554400	-2.49219300
C	-6.79653900	-3.49446400	-1.41150100	C	-6.81634900	-3.49318800	-1.40835300
C	-6.79653900	-3.49446400	1.41150100	C	-6.81634900	-3.49318800	1.40835300
H	-6.78644100	-3.45373500	-2.49813100	H	-6.80689900	-3.45826100	-2.49521500
H	-6.78644100	-3.45373500	2.49813100	H	-6.80689900	-3.45826100	2.49521500
C	-1.45277300	-1.19225200	-0.72434200	C	-1.44994800	-1.19277000	-0.71901500
H	-2.30475400	-2.00680600	2.49380000	H	-2.30770300	-2.01121000	2.48963500
H	-4.36686100	-3.30206700	2.49631900	H	-4.38200800	-3.29554400	2.49219300
H	-2.30475400	-2.00680600	-2.49380000	H	-2.30770300	-2.01121000	-2.48963500
C	-8.90210900	-2.27146200	1.41273700	C	-8.93162000	-2.27573600	1.41021100
C	-9.53666600	-1.21121000	0.73102300	C	-9.55141700	-1.21383900	0.73008100
C	-9.53666600	-1.21121000	-0.73102300	C	-9.55141700	-1.21383900	-0.73008100
C	-8.90210900	-2.27146200	-1.41273700	C	-8.93162000	-2.27573600	-1.41021100
H	-8.86723700	-2.24218100	2.49909100	H	-8.90829700	-2.25694100	2.49714500
H	-8.86723700	-2.24218100	-2.49909100	H	-8.90829700	-2.25694100	-2.49714500
C	-0.78891900	0.00000000	1.39832600	C	-0.78933900	0.00000000	1.39618900
C	-0.78891900	0.00000000	-1.39832600	C	-0.78933900	0.00000000	-1.39618900
H	-1.09347800	0.00000000	2.44802600	H	-1.09551800	0.00000000	2.44543800
H	-1.09347800	0.00000000	-2.44802600	H	-1.09551800	0.00000000	-2.44543800
C	-3.25808300	2.83594900	0.72805200	C	-3.25650600	2.83087300	0.72243600
C	-3.25808300	2.83594900	-0.72805200	C	-3.25650600	2.83087300	-0.72243600
C	-8.90210900	2.27146200	1.41273700	C	-8.93162000	2.27573600	1.41021100
C	-7.95519400	3.05106000	0.73067700	C	-7.95990500	3.05237100	0.72859800

C	-6.79653900	3.49446400	1.41150100	C	-6.81634900	3.49318800	1.40835300
C	-7.95519400	3.05106000	-0.73067700	C	-7.95990500	3.05237100	-0.72859800
C	-6.79653900	3.49446400	-1.41150100	C	-6.81634900	3.49318800	-1.40835300
C	-5.58068200	3.58522300	-0.72974000	C	-5.57951100	3.58032100	-0.72562200
C	-5.58068200	3.58522300	0.72974000	C	-5.57951100	3.58032100	0.72562200
C	-8.90210900	2.27146200	-1.41273700	C	-8.93162000	2.27573600	-1.41021100
H	-6.78644100	3.45373500	-2.49813100	H	-6.80689900	3.45826100	-2.49521500
C	-4.35583000	3.33830100	-1.40932700	C	-4.37103000	3.33397100	-1.40526300
C	-4.35583000	3.33830100	1.40932700	C	-4.37103000	3.33397100	1.40526300
H	-4.36686100	3.30206700	-2.49631900	H	-4.38200800	3.29554400	-2.49219300
H	-4.36686100	3.30206700	2.49631900	H	-4.38200800	3.29554400	2.49219300
H	-8.86723700	2.24218100	2.49909100	H	-8.90829700	2.25694100	2.49714500
H	-6.78644100	3.45373500	2.49813100	H	-6.80689900	3.45826100	2.49521500
H	-8.86723700	2.24218100	-2.49909100	H	-8.90829700	2.25694100	-2.49714500
C	-2.26309000	2.05035400	1.40762700	C	-2.26754100	2.05333600	1.40333000
C	-1.45277300	1.19225200	0.72434200	C	-1.44994800	1.19277000	0.71901500
C	-1.45277300	1.19225200	-0.72434200	C	-1.44994800	1.19277000	-0.71901500
C	-2.26309000	2.05035400	-1.40762700	C	-2.26754100	2.05333600	-1.40333000
H	-2.30475400	2.00680600	2.49380000	H	-2.30770300	2.01121000	2.48963500
H	-2.30475400	2.00680600	-2.49380000	H	-2.30770300	2.01121000	-2.48963500
**Dcyc11_R**				**Dcyc11_U**			
C	-1.44496800	1.19498400	0.72449900	C	-1.44254900	1.19620600	0.71952900
C	-1.44496800	1.19498400	-0.72449900	C	-1.44254900	1.19620600	-0.71952900
C	-7.86860800	-3.70613300	0.73116300	C	-7.86945500	-3.70900900	0.72861600
C	-7.86860800	-3.70613300	-0.73116300	C	-7.86945500	-3.70900900	-0.72861600
C	-6.65033200	3.93953800	1.41134900	C	-6.66662400	3.93984000	1.40812900
C	-5.43476900	3.85994100	0.73036600	C	-5.43114700	3.85872800	0.72582300
C	-4.24192600	3.48976400	1.40956600	C	-4.25360300	3.49070800	1.40529900
C	-5.43476900	3.85994100	-0.73036600	C	-5.43114700	3.85872800	-0.72582300
C	-4.24192600	3.48976400	-1.40956600	C	-4.25360300	3.49070800	-1.40529900
C	-3.18549700	2.90721700	-0.72811400	C	-3.18099800	2.90722200	-0.72270600
C	-3.18549700	2.90721700	0.72811400	C	-3.18099800	2.90722200	0.72270600
C	-7.86860800	3.70613300	0.73116300	C	-7.86945500	3.70900900	0.72861600
C	-6.65033200	3.93953800	-1.41134900	C	-6.66662400	3.93984000	-1.40812900
H	-4.25441100	3.45842400	-2.49660000	H	-4.26617900	3.45807700	-2.49241600
C	-2.22769100	2.07760400	-1.40767500	C	-2.23219200	2.08060700	-1.40380400
C	-2.22769100	2.07760400	1.40767500	C	-2.23219200	2.08060700	1.40380400
H	-2.26618500	2.04047200	-2.49419900	H	-2.27184600	2.04236700	-2.49027700
H	-2.26618500	2.04047200	2.49419900	H	-2.27184600	2.04236700	2.49027700
C	-7.86860800	3.70613300	-0.73116300	C	-7.86945500	3.70900900	-0.72861600
H	-6.64751100	3.90324500	2.49814400	H	-6.66323800	3.90716500	2.49513300
H	-4.25441100	3.45842400	2.49660000	H	-4.26617900	3.45807700	2.49241600
H	-6.64751100	3.90324500	-2.49814400	H	-6.66323800	3.90716500	-2.49513300
C	-8.96302500	-3.15617100	1.41267500	C	-8.98562000	-3.16092200	1.40978100
C	-9.85319400	-2.29905700	0.73177700	C	-9.86285000	-2.30378600	0.73004600
C	-10.43501200	-1.21626700	1.41338700	C	-10.46511200	-1.21948300	1.41023600
C	-9.85319400	-2.29905700	-0.73177700	C	-9.86285000	-2.30378600	-0.73004600
C	-10.43501200	-1.21626700	-1.41338700	C	-10.46511200	-1.21948300	-1.41023600
C	-10.63424500	0.00000000	-0.73202200	C	-10.64892500	0.00000000	-0.73037000
C	-10.63424500	0.00000000	0.73202200	C	-10.64892500	0.00000000	0.73037000
C	-8.96302500	-3.15617100	-1.41267500	C	-8.98562000	-3.16092200	-1.40978100
C	-10.43501200	1.21626700	-1.41338700	C	-10.46511200	1.21948300	-1.41023600
C	-10.43501200	1.21626700	1.41338700	C	-10.46511200	1.21948300	1.41023600
H	-8.93919100	-3.12351600	2.49923300	H	-8.96837500	-3.13807500	2.49679500
H	-8.93919100	-3.12351600	-2.49923300	H	-8.96837500	-3.13807500	-2.49679500
C	-9.85319400	2.29905700	0.73177700	C	-9.86285000	2.30378600	0.73004600
C	-9.85319400	2.29905700	-0.73177700	C	-9.86285000	2.30378600	-0.73004600
C	-8.96302500	3.15617100	-1.41267500	C	-8.98562000	3.16092200	-1.40978100

C	-8.96302500	3.15617100	1.41267500	C	-8.98562000	3.16092200	1.40978100
H	-10.39443800	-1.20259600	-2.49986600	H	-10.44099800	-1.21157500	-2.49728700
H	-10.39443800	-1.20259600	2.49986600	H	-10.44099800	-1.21157500	2.49728700
H	-10.39443800	1.20259600	2.49986600	H	-10.44099800	1.21157500	2.49728700
H	-10.39443800	1.20259600	-2.49986600	H	-10.44099800	1.21157500	-2.49728700
H	-8.93919100	3.12351600	-2.49923300	H	-8.96837500	3.13807500	-2.49679500
H	-8.93919100	3.12351600	2.49923300	H	-8.96837500	3.13807500	2.49679500
C	-6.65033200	-3.93953800	-1.41134900	C	-6.66662400	-3.93984000	-1.40812900
C	-5.43476900	-3.85994100	-0.73036600	C	-5.43114700	-3.85872800	-0.72582300
C	-4.24192600	-3.48976400	-1.40956600	C	-4.25360300	-3.49070800	-1.40529900
C	-5.43476900	-3.85994100	0.73036600	C	-5.43114700	-3.85872800	0.72582300
C	-4.24192600	-3.48976400	1.40956600	C	-4.25360300	-3.49070800	1.40529900
C	-3.18549700	-2.90721700	0.72811400	C	-3.18099800	-2.90722200	0.72270600
C	-3.18549700	-2.90721700	-0.72811400	C	-3.18099800	-2.90722200	-0.72270600
C	-6.65033200	-3.93953800	1.41134900	C	-6.66662400	-3.93984000	1.40812900
C	-2.22769100	-2.07760400	1.40767500	C	-2.23219200	-2.08060700	1.40380400
C	-2.22769100	-2.07760400	-1.40767500	C	-2.23219200	-2.08060700	-1.40380400
H	-6.64751100	-3.90324500	-2.49814400	H	-6.66323800	-3.90716500	-2.49513300
H	-6.64751100	-3.90324500	2.49814400	H	-6.66323800	-3.90716500	2.49513300
C	-1.44496800	-1.19498400	-0.72449900	C	-1.44254900	-1.19620600	-0.71952900
C	-1.44496800	-1.19498400	0.72449900	C	-1.44254900	-1.19620600	0.71952900
C	-0.78994300	0.00000000	1.39913300	C	-0.79042500	0.00000000	1.39662100
C	-0.78994300	0.00000000	-1.39913300	C	-0.79042500	0.00000000	-1.39662100
H	-4.25441100	-3.45842400	2.49660000	H	-4.26617900	-3.45807700	2.49241600
H	-4.25441100	-3.45842400	-2.49660000	H	-4.26617900	-3.45807700	-2.49241600
H	-2.26618500	-2.04047200	-2.49419900	H	-2.27184600	-2.04236700	-2.49027700
H	-2.26618500	-2.04047200	2.49419900	H	-2.27184600	-2.04236700	2.49027700
H	-1.09544900	0.00000000	2.44858500	H	-1.09714500	0.00000000	2.44575300
H	-1.09544900	0.00000000	-2.44858500	H	-1.09714500	0.00000000	-2.44575300
C	10.63424500	0.00000000	0.73202200	C	10.64892500	0.00000000	0.73037000
C	10.63424500	0.00000000	-0.73202200	C	10.64892500	0.00000000	-0.73037000
C	3.18549700	-2.90721700	0.72811400	C	3.18099800	-2.90722200	0.72270600
C	3.18549700	-2.90721700	-0.72811400	C	3.18099800	-2.90722200	-0.72270600
C	6.65033200	3.93953800	1.41134900	C	6.66662400	3.93984000	1.40812900
C	7.86860800	3.70613300	0.73116300	C	7.86945500	3.70900900	0.72861600
C	8.96302500	3.15617100	1.41267500	C	8.98562000	3.16092200	1.40978100
C	7.86860800	3.70613300	-0.73116300	C	7.86945500	3.70900900	-0.72861600
C	8.96302500	3.15617100	-1.41267500	C	8.98562000	3.16092200	-1.40978100
C	9.85319400	2.29905700	-0.73177700	C	9.86285000	2.30378600	-0.73004600
C	9.85319400	2.29905700	0.73177700	C	9.86285000	2.30378600	0.73004600
C	5.43476900	3.85994100	0.73036600	C	5.43114700	3.85872800	0.72582300
C	6.65033200	3.93953800	-1.41134900	C	6.66662400	3.93984000	-1.40812900
H	8.93919100	3.12351600	-2.49923300	H	8.96837500	3.13807500	-2.49679500
C	10.43501200	1.21626700	-1.41338700	C	10.46511200	1.21948300	-1.41023600
C	10.43501200	1.21626700	1.41338700	C	10.46511200	1.21948300	1.41023600
H	10.39443800	1.20259600	-2.49986600	H	10.44099800	1.21157500	-2.49728700
H	10.39443800	1.20259600	2.49986600	H	10.44099800	1.21157500	2.49728700
C	5.43476900	3.85994100	-0.73036600	C	5.43114700	3.85872800	-0.72582300
H	6.64751100	3.90324500	2.49814400	H	6.66323800	3.90716500	2.49513300
H	8.93919100	3.12351600	2.49923300	H	8.96837500	3.13807500	2.49679500
H	6.64751100	3.90324500	-2.49814400	H	6.66323800	3.90716500	-2.49513300
C	2.22769100	-2.07760400	1.40767500	C	2.23219200	-2.08060700	1.40380400
C	1.44496800	-1.19498400	0.72449900	C	1.44254900	-1.19620600	0.71952900
C	0.78994300	0.00000000	1.39913300	C	0.79042500	0.00000000	1.39662100
C	1.44496800	-1.19498400	-0.72449900	C	1.44254900	-1.19620600	-0.71952900
C	0.78994300	0.00000000	-1.39913300	C	0.79042500	0.00000000	-1.39662100
C	1.44496800	1.19498400	-0.72449900	C	1.44254900	1.19620600	-0.71952900
C	1.44496800	1.19498400	0.72449900	C	1.44254900	1.19620600	0.71952900
C	2.22769100	-2.07760400	-1.40767500	C	2.23219200	-2.08060700	-1.40380400
C	2.22769100	2.07760400	-1.40767500	C	2.23219200	2.08060700	-1.40380400

C	2.22769100	2.07760400	1.40767500	C	2.23219200	2.08060700	1.40380400
H	2.26618500	-2.04047200	2.49419900	H	2.27184600	-2.04236700	2.49027700
H	2.26618500	-2.04047200	-2.49419900	H	2.27184600	-2.04236700	-2.49027700
C	3.18549700	2.90721700	0.72811400	C	3.18099800	2.90722200	0.72270600
C	3.18549700	2.90721700	-0.72811400	C	3.18099800	2.90722200	-0.72270600
C	4.24192600	3.48976400	-1.40956600	C	4.25360300	3.49070800	-1.40529900
C	4.24192600	3.48976400	1.40956600	C	4.25360300	3.49070800	1.40529900
H	1.09544900	0.00000000	-2.44858500	H	1.09714500	0.00000000	-2.44575300
H	1.09544900	0.00000000	2.44858500	H	1.09714500	0.00000000	2.44575300
H	2.26618500	2.04047200	2.49419900	H	2.27184600	2.04236700	2.49027700
H	2.26618500	2.04047200	-2.49419900	H	2.27184600	2.04236700	-2.49027700
H	4.25441100	3.45842400	-2.49660000	H	4.26617900	3.45807700	-2.49241600
H	4.25441100	3.45842400	2.49660000	H	4.26617900	3.45807700	2.49241600
C	4.24192600	-3.48976400	-1.40956600	C	4.25360300	-3.49070800	-1.40529900
C	5.43476900	-3.85994100	-0.73036600	C	5.43114700	-3.85872800	-0.72582300
C	6.65033200	-3.93953800	-1.41134900	C	6.66662400	-3.93984000	-1.40812900
C	5.43476900	-3.85994100	0.73036600	C	5.43114700	-3.85872800	0.72582300
C	6.65033200	-3.93953800	1.41134900	C	6.66662400	-3.93984000	1.40812900
C	7.86860800	-3.70613300	0.73116300	C	7.86945500	-3.70900900	0.72861600
C	7.86860800	-3.70613300	-0.73116300	C	7.86945500	-3.70900900	-0.72861600
C	4.24192600	-3.48976400	1.40956600	C	4.25360300	-3.49070800	1.40529900
C	8.96302500	-3.15617100	1.41267500	C	8.98562000	-3.16092200	1.40978100
C	8.96302500	-3.15617100	-1.41267500	C	8.98562000	-3.16092200	-1.40978100
H	4.25441100	-3.45842400	-2.49660000	H	4.26617900	-3.45807700	-2.49241600
H	4.25441100	-3.45842400	2.49660000	H	4.26617900	-3.45807700	2.49241600
C	9.85319400	-2.29905700	-0.73177700	C	9.86285000	-2.30378600	-0.73004500
C	9.85319400	-2.29905700	0.73177700	C	9.86285000	-2.30378600	0.73004600
C	10.43501200	-1.21626700	1.41338700	C	10.46511200	-1.21948300	1.41023600
C	10.43501200	-1.21626700	-1.41338700	C	10.46511200	-1.21948300	-1.41023600
H	6.64751100	-3.90324500	2.49814400	H	6.66323800	-3.90716500	2.49513300
H	6.64751100	-3.90324500	-2.49814400	H	6.66323800	-3.90716500	-2.49513300
H	8.93919100	-3.12351600	-2.49923300	H	8.96837500	-3.13807500	-2.49679500
H	8.93919100	-3.12351600	2.49923300	H	8.96837500	-3.13807500	2.49679500
H	10.39443800	-1.20259600	2.49986600	H	10.44099800	-1.21157500	2.49728700
H	10.39443800	-1.20259600	-2.49986600	H	10.44099800	-1.21157500	-2.49728700

**Dcyc12_R**				**Dcyc12_U**			
C	-5.29989600	4.07839900	0.73038300	C	-5.29767800	4.07449400	0.72614300
C	-5.29989600	4.07839900	-0.73038300	C	-5.29767800	4.07449400	-0.72614300
C	-4.14191900	-3.61066200	1.40935900	C	-4.15452800	-3.61062300	1.40538200
C	-5.29989600	-4.07839900	0.73038300	C	-5.29767800	-4.07449400	0.72614300
C	-6.49860600	-4.29056700	1.41119500	C	-6.51544200	-4.29175500	1.40795200
C	-5.29989600	-4.07839900	-0.73038300	C	-5.29767800	-4.07449400	-0.72614300
C	-6.49860600	-4.29056700	-1.41119500	C	-6.51544200	-4.29175500	-1.40795200
C	-7.73746800	-4.23189300	-0.73153800	C	-7.73862400	-4.23250200	-0.72879200
C	-7.73746800	-4.23189300	0.73153800	C	-7.73862400	-4.23250200	0.72879200
C	-3.12278300	-2.96619800	0.72836900	C	-3.12049500	-2.96286500	0.72312600
C	-4.14191900	-3.61066200	-1.40935900	C	-4.15452800	-3.61062300	-1.40538200
H	-6.50005700	-4.25830900	-2.49819100	H	-6.51607900	-4.26266700	-2.49508900
C	-8.91004900	-3.88151100	-1.41245700	C	-8.93176200	-3.88607100	-1.40943300
C	-8.91004900	-3.88151100	1.41245700	C	-8.93176200	-3.88607100	1.40943300
H	-8.89492400	-3.84903900	-2.49924000	H	-8.92026400	-3.86226200	-2.49655400
H	-8.89492400	-3.84903900	2.49924000	H	-8.92026400	-3.86226200	2.49655400
C	-3.12278300	-2.96619800	-0.72836900	C	-3.12049500	-2.96286500	-0.72312600
H	-4.15504400	-3.58363400	2.49657900	H	-4.16797000	-3.58192200	2.49259900
H	-6.50005700	-4.25830900	2.49819100	H	-6.51607900	-4.26266700	2.49508900
H	-4.15504400	-3.58363400	-2.49657900	H	-4.16797000	-3.58192200	-2.49259900
C	-9.96126900	-3.23208100	0.73212700	C	-9.97081200	-3.23698400	0.72993400
C	-9.96126900	-3.23208100	-0.73212700	C	-9.97081200	-3.23698400	-0.72993400

C	-4.14191900	3.61066200	1.40935900	C	-4.15452800	3.61062300	1.40538200
C	-3.12278300	2.96619800	0.72836900	C	-3.12049500	2.96286500	0.72312600
C	-2.19897400	2.09909900	1.40793200	C	-2.20241200	2.10270800	1.40401700
C	-3.12278300	2.96619800	-0.72836900	C	-3.12049500	2.96286500	-0.72312600
C	-2.19897400	2.09909900	-1.40793200	C	-2.20241200	2.10270800	-1.40401700
C	-1.43878000	1.19803100	-0.72436900	C	-1.43652000	1.19839800	-0.71951900
C	-1.43878000	1.19803100	0.72436900	C	-1.43652000	1.19839800	0.71951900
C	-4.14191900	3.61066200	-1.40935900	C	-4.15452800	3.61062300	-1.40538200
C	-0.79144200	0.00000000	-1.39909900	C	-0.79180200	0.00000000	-1.39713000
C	-0.79144200	0.00000000	1.39909900	C	-0.79180200	0.00000000	1.39713000
H	-4.15504400	3.58363400	2.49657900	H	-4.16797000	3.58192200	2.49259900
H	-4.15504400	3.58363400	-2.49657900	H	-4.16797000	3.58192200	-2.49259900
C	-1.43878000	-1.19803100	0.72436900	C	-1.43652000	-1.19839800	0.71951900
C	-1.43878000	-1.19803100	-0.72436900	C	-1.43652000	-1.19839800	-0.71951900
C	-2.19897400	-2.09909900	-1.40793200	C	-2.20241200	-2.10270800	-1.40401700
C	-2.19897400	-2.09909900	1.40793200	C	-2.20241200	-2.10270800	1.40401700
H	-2.23636900	2.06517500	-2.49457900	H	-2.23871300	2.06981200	-2.49075300
H	-2.23636900	2.06517500	2.49457900	H	-2.23871300	2.06981200	2.49075300
H	-1.09693500	0.00000000	2.44852600	H	-1.09872500	0.00000000	2.44617000
H	-1.09693500	0.00000000	-2.44852600	H	-1.09872500	0.00000000	-2.44617000
H	-2.23636900	-2.06517500	-2.49457900	H	-2.23871300	-2.06981200	-2.49075300
H	-2.23636900	-2.06517500	2.49457900	H	-2.23871300	-2.06981200	2.49075300
C	-10.79110700	2.32718500	1.41324900	C	-10.81946900	2.33215600	1.40973100
C	-9.96126900	3.23208100	0.73212700	C	-9.97081200	3.23698400	0.72993400
C	-8.91004900	3.88151100	1.41245700	C	-8.93176200	3.88607100	1.40943300
C	-9.96126900	3.23208100	-0.73212700	C	-9.97081200	3.23698400	-0.72993400
C	-8.91004900	3.88151100	-1.41245700	C	-8.93176200	3.88607100	-1.40943300
C	-7.73746800	4.23189300	-0.73153800	C	-7.73862400	4.23250200	-0.72879200
C	-7.73746800	4.23189300	0.73153800	C	-7.73862400	4.23250200	0.72879200
C	-11.32535800	1.21614100	0.73235600	C	-11.34279600	1.21906700	0.73009600
C	-10.79110700	2.32718500	-1.41324900	C	-10.81946900	2.33215600	-1.40973100
H	-8.89492400	3.84903900	-2.49924000	H	-8.92026400	3.86226200	-2.49655400
C	-6.49860600	4.29056700	-1.41119500	C	-6.51544200	4.29175500	-1.40795200
C	-6.49860600	4.29056700	1.41119500	C	-6.51544200	4.29175500	1.40795200
H	-6.50005700	4.25830900	-2.49819100	H	-6.51607900	4.26266700	-2.49508900
H	-6.50005700	4.25830900	2.49819100	H	-6.51607900	4.26266700	2.49508900
C	-11.32535800	1.21614100	-0.73235600	C	-11.34279600	1.21906700	-0.73009600
H	-10.75894900	2.30577800	2.49986600	H	-10.79910700	2.31872200	2.49686500
H	-8.89492400	3.84903900	2.49924000	H	-8.92026400	3.86226200	2.49655400
H	-10.75894900	2.30577800	-2.49986600	H	-10.79910700	2.31872200	-2.49686500
C	-10.79110700	-2.32718500	-1.41324900	C	-10.81946900	-2.33215600	-1.40973100
C	-10.79110700	-2.32718500	1.41324900	C	-10.81946900	-2.33215600	1.40973100
C	-11.32535800	-1.21614100	0.73235600	C	-11.34279600	-1.21906700	0.73009600
C	-11.32535800	-1.21614100	-0.73235600	C	-11.34279600	-1.21906700	-0.73009600
C	-11.51288300	0.00000000	-1.41352500	C	-11.54437300	0.00000000	-1.40971100
C	-11.51288300	0.00000000	1.41352500	C	-11.54437300	0.00000000	1.40971100
H	-10.75894900	-2.30577800	2.49986600	H	-10.79910700	-2.31872200	2.49686500
H	-10.75894900	-2.30577800	-2.49986600	H	-10.79910700	-2.31872200	-2.49686500
H	-11.47338600	0.00000000	-2.50009000	H	-11.52061200	0.00000000	-2.49685400
H	-11.47338600	0.00000000	2.50009000	H	-11.52061200	0.00000000	2.49685400
C	9.96126900	3.23208100	0.73212700	C	9.97081200	3.23698400	0.72993400
C	9.96126900	3.23208100	-0.73212700	C	9.97081200	3.23698400	-0.72993400
C	6.49860600	-4.29056700	1.41119500	C	6.51544200	-4.29175500	1.40795200
C	5.29989600	-4.07839900	0.73038300	C	5.29767800	-4.07449400	0.72614300
C	4.14191900	-3.61066200	1.40935900	C	4.15452800	-3.61062300	1.40538200
C	5.29989600	-4.07839900	-0.73038300	C	5.29767800	-4.07449400	-0.72614300
C	4.14191900	-3.61066200	-1.40935900	C	4.15452800	-3.61062300	-1.40538200
C	3.12278300	-2.96619800	-0.72836900	C	3.12049500	-2.96286500	-0.72312600
C	3.12278300	-2.96619800	0.72836900	C	3.12049500	-2.96286500	0.72312600
C	7.73746800	-4.23189300	0.73153800	C	7.73862400	-4.23250200	0.72879200

C	6.49860600	-4.29056700	-1.41119500	C	6.51544200	-4.29175500	-1.40795200
H	4.15504400	-3.58363400	-2.49657900	H	4.16797000	-3.58192200	-2.49259900
C	2.19897400	-2.09909900	-1.40793200	C	2.20241200	-2.10270800	-1.40401700
C	2.19897400	-2.09909900	1.40793200	C	2.20241200	-2.10270800	1.40401700
H	2.23636900	-2.06517500	-2.49457900	H	2.23871300	-2.06981200	-2.49075300
H	2.23636900	-2.06517500	2.49457900	H	2.23871300	-2.06981200	2.49075300
C	7.73746800	-4.23189300	-0.73153800	C	7.73862400	-4.23250200	-0.72879200
H	6.50005700	-4.25830900	2.49819100	H	6.51607900	-4.26266700	2.49508900
H	4.15504400	-3.58363400	2.49657900	H	4.16797000	-3.58192200	2.49259900
H	6.50005700	-4.25830900	-2.49819100	H	6.51607900	-4.26266700	-2.49508900
C	1.43878000	-1.19803100	0.72436900	C	1.43652000	-1.19839800	0.71951900
C	1.43878000	-1.19803100	-0.72436900	C	1.43652000	-1.19839800	-0.71951900
C	10.79110700	2.32718500	1.41324900	C	10.81946900	2.33215600	1.40973100
C	11.32535800	1.21614100	0.73235600	C	11.34279600	1.21906700	0.73009600
C	11.51288300	0.00000000	1.41352500	C	11.54437300	0.00000000	1.40971100
C	11.32535800	1.21614100	-0.73235600	C	11.34279600	1.21906700	-0.73009600
C	11.51288300	0.00000000	-1.41352500	C	11.54437300	0.00000000	-1.40971100
C	11.32535800	-1.21614100	-0.73235600	C	11.34279600	-1.21906700	-0.73009600
C	11.32535800	-1.21614100	0.73235600	C	11.34279600	-1.21906700	0.73009600
C	10.79110700	2.32718500	-1.41324900	C	10.81946900	2.33215600	-1.40973100
C	10.79110700	-2.32718500	-1.41324900	C	10.81946900	-2.33215600	-1.40973100
C	10.79110700	-2.32718500	1.41324900	C	10.81946900	-2.33215600	1.40973100
H	10.75894900	2.30577800	2.49986600	H	10.79910700	2.31872200	2.49686500
H	10.75894900	2.30577800	-2.49986600	H	10.79910700	2.31872200	-2.49686500
C	9.96126900	-3.23208100	0.73212700	C	9.97081200	-3.23698400	0.72993400
C	9.96126900	-3.23208100	-0.73212700	C	9.97081200	-3.23698400	-0.72993400
C	8.91004900	-3.88151100	-1.41245700	C	8.93176200	-3.88607100	-1.40943300
C	8.91004900	-3.88151100	1.41245700	C	8.93176200	-3.88607100	1.40943300
H	11.47338600	0.00000000	-2.50009000	H	11.52061200	0.00000000	-2.49685400
H	11.47338600	0.00000000	2.50009000	H	11.52061200	0.00000000	2.49685400
H	10.75894900	-2.30577800	2.49986600	H	10.79910700	-2.31872200	2.49686500
H	10.75894900	-2.30577800	-2.49986600	H	10.79910700	-2.31872200	-2.49686500
H	8.89492400	-3.84903900	-2.49924000	H	8.92026400	-3.86226200	-2.49655400
H	8.89492400	-3.84903900	2.49924000	H	8.92026400	-3.86226200	2.49655400
C	4.14191900	3.61066200	1.40935900	C	4.15452800	3.61062300	1.40538200
C	5.29989600	4.07839900	0.73038300	C	5.29767800	4.07449400	0.72614300
C	6.49860600	4.29056700	1.41119500	C	6.51544200	4.29175500	1.40795200
C	5.29989600	4.07839900	-0.73038200	C	5.29767800	4.07449400	-0.72614300
C	6.49860600	4.29056700	-1.41119500	C	6.51544200	4.29175500	-1.40795200
C	7.73746800	4.23189300	-0.73153800	C	7.73862400	4.23250200	-0.72879200
C	7.73746800	4.23189300	0.73153800	C	7.73862400	4.23250200	0.72879200
C	3.12278300	2.96619800	0.72836900	C	3.12049500	2.96286500	0.72312600
C	4.14191900	3.61066200	-1.40935900	C	4.15452800	3.61062300	-1.40538200
H	6.50005700	4.25830900	-2.49819100	H	6.51607900	4.26266700	-2.49508900
C	8.91004900	3.88151100	-1.41245700	C	8.93176200	3.88607100	-1.40943300
C	8.91004900	3.88151100	1.41245700	C	8.93176200	3.88607100	1.40943300
H	8.89492400	3.84903900	-2.49924000	H	8.92026400	3.86226200	-2.49655400
H	8.89492400	3.84903900	2.49924000	H	8.92026400	3.86226200	2.49655400
C	3.12278300	2.96619800	-0.72836900	C	3.12049500	2.96286500	-0.72312600
H	4.15504400	3.58363400	2.49657900	H	4.16797000	3.58192200	2.49259900
H	6.50005700	4.25830900	2.49819100	H	6.51607900	4.26266700	2.49508900
H	4.15504400	3.58363400	-2.49657900	H	4.16797000	3.58192200	-2.49259900
C	0.79144200	0.00000000	-1.39909900	C	0.79180200	0.00000000	-1.39713000
C	0.79144200	0.00000000	1.39909900	C	0.79180200	0.00000000	1.39713000
C	1.43878000	1.19803100	0.72436900	C	1.43652000	1.19839800	0.71951900
C	1.43878000	1.19803100	-0.72436900	C	1.43652000	1.19839800	-0.71951900
C	2.19897400	2.09909900	-1.40793200	C	2.20241200	2.10270800	-1.40401700
C	2.19897400	2.09909900	1.40793200	C	2.20241200	2.10270800	1.40401700
H	1.09693500	0.00000000	2.44852600	H	1.09872500	0.00000000	2.44617000
H	1.09693500	0.00000000	-2.44852600	H	1.09872500	0.00000000	-2.44617000

H	2.23636900	2.06517500	-2.49457900	H	2.23871300	2.06981200	-2.49075300
H	2.23636900	2.06517500	2.49457900	H	2.23871300	2.06981200	2.49075300
**Dcyc13_R**				**Dcyc13_U**			
C	9.94554500	4.01604500	0.73244300	C	9.95008500	4.02305200	0.72993300
C	9.94554500	4.01604500	-0.73244300	C	9.95008500	4.02305200	-0.72993300
C	10.94117700	-3.29921400	1.41316500	C	10.96229200	-3.30666200	1.40951700
C	9.94554500	-4.01604500	0.73244300	C	9.95008500	-4.02305200	0.72993300
C	8.79864700	-4.47545000	1.41236000	C	8.81517000	-4.48364500	1.40931200
C	9.94554500	-4.01604500	-0.73244300	C	9.95008500	-4.02305200	-0.72993300
C	8.79864700	-4.47545000	-1.41236000	C	8.81517000	-4.48364500	-1.40931200
C	7.58880000	-4.65488900	-0.73177700	C	7.58625700	-4.65919600	-0.72882200
C	7.58880000	-4.65488900	0.73177700	C	7.58625700	-4.65919600	0.72882200
C	11.71920600	-2.34287100	0.73277700	C	11.73086200	-2.34817700	0.72999800
C	10.94117700	-3.29921400	-1.41316500	C	10.96229200	-3.30666200	-1.40951700
H	8.78988600	-4.44469700	-2.49921100	H	8.80823000	-4.46061900	-2.49641500
C	6.35152900	-4.57223300	-1.41107000	C	6.36416200	-4.57787700	-1.40784400
C	6.35152900	-4.57223300	1.41107000	C	6.36416200	-4.57787700	1.40784400
H	6.35575600	-4.54384600	-2.49816700	H	6.36741800	-4.55187300	-2.49506800
H	6.35575600	-4.54384600	2.49816700	H	6.36741800	-4.55187300	2.49506800
C	11.71920600	-2.34287100	-0.73277700	C	11.73086200	-2.34817700	-0.72999800
H	10.91709500	-3.27407800	2.49993100	H	10.94623300	-3.28998800	2.49671000
H	8.78988600	-4.44469700	2.49921100	H	8.80823000	-4.46061900	2.49641500
H	10.91709500	-3.27407800	-2.49993100	H	10.94623300	-3.28998800	-2.49671000
C	5.17736100	-4.25283200	0.73062200	C	5.17173300	-4.25307300	0.72631600
C	5.17736100	-4.25283200	-0.73062200	C	5.17173300	-4.25307300	-0.72631600
C	10.94117700	3.29921400	1.41316500	C	10.96229200	3.30666200	1.40951700
C	10.94117700	3.29921400	-1.41316500	C	10.96229200	3.30666200	-1.40951700
C	11.71920600	2.34287100	-0.73277700	C	11.73086200	2.34817700	-0.72999800
C	11.71920600	2.34287100	0.73277700	C	11.73086200	2.34817700	0.72999800
C	12.21843800	1.21957900	-1.41356100	C	12.24253100	1.22250500	-1.40934400
C	12.21843800	1.21957900	1.41356100	C	12.24253100	1.22250500	1.40934400
C	12.38600700	0.00000000	0.73284800	C	12.40054900	0.00000000	0.72984900
C	12.38600700	0.00000000	-0.73284800	C	12.40054900	0.00000000	-0.72984900
C	12.21843800	-1.21957900	-1.41356100	C	12.24253100	-1.22250500	-1.40934400
C	12.21843800	-1.21957900	1.41356100	C	12.24253100	-1.22250500	1.40934400
H	10.91709500	3.27407800	-2.49993100	H	10.94623300	3.28998800	-2.49671000
H	10.91709500	3.27407800	2.49993100	H	10.94623300	3.28998800	2.49671000
H	12.18342200	1.20974500	2.50022400	H	12.22015900	1.21624100	2.49650500
H	12.18342200	1.20974500	-2.50022400	H	12.22015900	1.21624100	-2.49650500
H	12.18342200	-1.20974500	-2.50022400	H	12.22015900	-1.21624100	-2.49650500
H	12.18342200	-1.20974500	2.50022400	H	12.22015900	-1.21624100	2.49650500
C	4.05400800	3.70783000	1.40935400	C	4.06238200	3.71266200	1.40534700
C	5.17736100	4.25283200	0.73062200	C	5.17173300	4.25307300	0.72631600
C	6.35152900	4.57223300	1.41107000	C	6.36416200	4.57787700	1.40784400
C	5.17736100	4.25283200	-0.73062200	C	5.17173300	4.25307300	-0.72631600
C	6.35152900	4.57223300	-1.41107000	C	6.36416200	4.57787700	-1.40784400
C	7.58880000	4.65488900	-0.73177700	C	7.58625700	4.65919600	-0.72882200
C	7.58880000	4.65488900	0.73177700	C	7.58625700	4.65919600	0.72882200
C	3.06878800	3.01328500	0.72853500	C	3.06322900	3.01383700	0.72318800
C	4.05400800	3.70783000	-1.40935400	C	4.06238200	3.71266200	-1.40534700
H	6.35575600	4.54384600	-2.49816700	H	6.36741800	4.55187300	-2.49506800
C	8.79864700	4.47545000	-1.41236000	C	8.81517000	4.48364500	-1.40931200
C	8.79864700	4.47545000	1.41236000	C	8.81517000	4.48364500	1.40931200
H	8.78988600	4.44469700	-2.49921100	H	8.80823000	4.46061900	-2.49641500
H	8.78988600	4.44469700	2.49921100	H	8.80823000	4.46061900	2.49641500
C	3.06878800	3.01328500	-0.72853500	C	3.06322900	3.01383700	-0.72318800
H	4.06736800	3.68406600	2.49664000	H	4.07595600	3.68775500	2.49267100
H	6.35575600	4.54384600	2.49816700	H	6.36741800	4.55187300	2.49506800

H	4.06736800	3.68406600	-2.49664000	H	4.07595600	3.68775500	-2.49267100
C	4.05400800	-3.70783000	1.40935400	C	4.06238200	-3.71266200	1.40534700
C	3.06878800	-3.01328500	0.72853500	C	3.06322900	-3.01383700	0.72318800
C	2.17341300	-2.11731700	1.40806800	C	2.17597700	-2.12165700	1.40431400
C	3.06878800	-3.01328500	-0.72853500	C	3.06322900	-3.01383700	-0.72318800
C	2.17341300	-2.11731700	-1.40806800	C	2.17597700	-2.12165700	-1.40431400
C	1.43334100	-1.20017100	-0.72432300	C	1.43097800	-1.20107800	-0.71969800
C	1.43334100	-1.20017100	0.72432300	C	1.43097800	-1.20107800	0.71969800
C	4.05400800	-3.70783000	-1.40935400	C	4.06238200	-3.71266200	-1.40534700
C	0.79240400	0.00000000	-1.39949800	C	0.79279200	0.00000000	-1.39726400
C	0.79240400	0.00000000	1.39949800	C	0.79279200	0.00000000	1.39726400
H	4.06736800	-3.68406600	2.49664000	H	4.07595600	-3.68775500	2.49267100
H	4.06736800	-3.68406600	-2.49664000	H	4.07595600	-3.68775500	-2.49267100
C	1.43334100	1.20017100	0.72432300	C	1.43097800	1.20107800	0.71969800
C	1.43334100	1.20017100	-0.72432300	C	1.43097800	1.20107800	-0.71969800
C	2.17341300	2.11731700	-1.40806800	C	2.17597700	2.12165700	-1.40431400
C	2.17341300	2.11731700	1.40806800	C	2.17597700	2.12165700	1.40431400
H	2.20848900	-2.08721600	-2.49483400	H	2.21151900	-2.09147200	-2.49106600
H	2.20848900	-2.08721600	2.49483400	H	2.21151900	-2.09147200	2.49106600
H	1.09840400	0.00000000	2.44875900	H	1.09983700	0.00000000	2.44628100
H	1.09840400	0.00000000	-2.44875900	H	1.09983700	0.00000000	-2.44628100
H	2.20848900	2.08721600	-2.49483400	H	2.21151900	2.09147200	-2.49106600
H	2.20848900	2.08721600	2.49483400	H	2.21151900	2.09147200	2.49106600
C	-5.17736100	4.25283200	0.73062200	C	-5.17173300	4.25307300	0.72631600
C	-5.17736100	4.25283200	-0.73062200	C	-5.17173300	4.25307300	-0.72631600
C	-2.17341300	-2.11731700	1.40806800	C	-2.17597700	-2.12165700	1.40431400
C	-3.06878800	-3.01328500	0.72853500	C	-3.06322900	-3.01383700	0.72318800
C	-4.05400800	-3.70783000	1.40935400	C	-4.06238200	-3.71266200	1.40534700
C	-3.06878800	-3.01328500	-0.72853500	C	-3.06322900	-3.01383700	-0.72318800
C	-4.05400800	-3.70783000	-1.40935400	C	-4.06238200	-3.71266200	-1.40534700
C	-5.17736100	-4.25283200	-0.73062200	C	-5.17173300	-4.25307300	-0.72631600
C	-5.17736100	-4.25283200	0.73062200	C	-5.17173300	-4.25307300	0.72631600
C	-1.43334100	-1.20017100	0.72432300	C	-1.43097800	-1.20107800	0.71969800
C	-2.17341300	-2.11731700	-1.40806800	C	-2.17597700	-2.12165700	-1.40431400
H	-4.06736800	-3.68406600	-2.49664000	H	-4.07595600	-3.68775500	-2.49267100
C	-6.35152900	-4.57223300	-1.41107000	C	-6.36416200	-4.57787700	-1.40784400
C	-6.35152900	-4.57223300	1.41107000	C	-6.36416200	-4.57787700	1.40784400
H	-6.35575600	-4.54384600	-2.49816700	H	-6.36741800	-4.55187300	-2.49506800
H	-6.35575600	-4.54384600	2.49816700	H	-6.36741800	-4.55187300	2.49506800
C	-1.43334100	-1.20017100	-0.72432300	C	-1.43097800	-1.20107800	-0.71969800
H	-2.20848900	-2.08721600	2.49483400	H	-2.21151900	-2.09147200	2.49106600
H	-4.06736800	-3.68406600	2.49664000	H	-4.07595600	-3.68775500	2.49267100
H	-2.20848900	-2.08721600	-2.49483400	H	-2.21151900	-2.09147200	-2.49106600
C	-7.58880000	-4.65488900	0.73177700	C	-7.58625700	-4.65919600	0.72882200
C	-7.58880000	-4.65488900	-0.73177700	C	-7.58625700	-4.65919600	-0.72882200
C	-4.05400800	3.70783000	1.40935400	C	-4.06238200	3.71266200	1.40534700
C	-4.05400800	3.70783000	-1.40935400	C	-4.06238200	3.71266200	-1.40534700
C	-3.06878800	3.01328500	-0.72853500	C	-3.06322900	3.01383700	-0.72318800
C	-3.06878800	3.01328500	0.72853500	C	-3.06322900	3.01383700	0.72318800
C	-2.17341300	2.11731700	-1.40806800	C	-2.17597700	2.12165700	-1.40431400
C	-2.17341300	2.11731700	1.40806800	C	-2.17597700	2.12165700	1.40431400
C	-1.43334100	1.20017100	0.72432300	C	-1.43097800	1.20107800	0.71969800
C	-1.43334100	1.20017100	-0.72432300	C	-1.43097800	1.20107800	-0.71969800
C	-0.79240400	0.00000000	-1.39949800	C	-0.79279200	0.00000000	-1.39726400
C	-0.79240400	0.00000000	1.39949800	C	-0.79279200	0.00000000	1.39726400
H	-4.06736800	3.68406600	-2.49664000	H	-4.07595600	3.68775500	-2.49267100
H	-4.06736800	3.68406600	2.49664000	H	-4.07595600	3.68775500	2.49267100
H	-2.20848900	2.08721600	2.49483400	H	-2.21151900	2.09147200	2.49106600
H	-2.20848900	2.08721600	-2.49483400	H	-2.21151900	2.09147200	-2.49106600
H	-1.09840400	0.00000000	-2.44875900	H	-1.09983700	0.00000000	-2.44628100

H	-1.09840400	0.00000000	2.44875900	H	-1.09983700	0.00000000	2.44628100
C	-10.94117700	3.29921400	1.41316500	C	-10.96229200	3.30666200	1.40951700
C	-9.94554500	4.01604500	0.73244300	C	-9.95008500	4.02305200	0.72993300
C	-8.79864700	4.47545000	1.41236000	C	-8.81517000	4.48364500	1.40931200
C	-9.94554500	4.01604500	-0.73244300	C	-9.95008500	4.02305200	-0.72993300
C	-8.79864700	4.47545000	-1.41236000	C	-8.81517000	4.48364500	-1.40931200
C	-7.58880000	4.65488900	-0.73177700	C	-7.58625700	4.65919600	-0.72882200
C	-7.58880000	4.65488900	0.73177700	C	-7.58625700	4.65919600	0.72882200
C	-11.71920600	2.34287100	0.73277700	C	-11.73086200	2.34817700	0.72999800
C	-10.94117700	3.29921400	-1.41316500	C	-10.96229200	3.30666200	-1.40951700
H	-8.78988600	4.44469700	-2.49921100	H	-8.80823000	4.46061900	-2.49641500
C	-6.35152900	4.57223300	-1.41107000	C	-6.36416200	4.57787700	-1.40784400
C	-6.35152900	4.57223300	1.41107000	C	-6.36416200	4.57787700	1.40784400
H	-6.35575600	4.54384600	-2.49816700	H	-6.36741800	4.55187300	-2.49506800
H	-6.35575600	4.54384600	2.49816700	H	-6.36741800	4.55187300	2.49506800
C	-11.71920600	2.34287100	-0.73277700	C	-11.73086200	2.34817700	-0.72999800
H	-10.91709500	3.27407800	2.49993100	H	-10.94623300	3.28998800	2.49671000
H	-8.78988600	4.44469700	2.49921100	H	-8.80823000	4.46061900	2.49641500
H	-10.91709500	3.27407800	-2.49993100	H	-10.94623300	3.28998800	-2.49671000
C	-8.79864700	-4.47545000	1.41236000	C	-8.81517000	-4.48364500	1.40931200
C	-9.94554500	-4.01604500	0.73244300	C	-9.95008500	-4.02305200	0.72993300
C	-10.94117700	-3.29921400	1.41316500	C	-10.96229200	-3.30666200	1.40951700
C	-9.94554500	-4.01604500	-0.73244300	C	-9.95008500	-4.02305200	-0.72993300
C	-10.94117700	-3.29921400	-1.41316500	C	-10.96229200	-3.30666200	-1.40951700
C	-11.71920600	-2.34287100	-0.73277700	C	-11.73086200	-2.34817700	-0.72999800
C	-11.71920600	-2.34287100	0.73277700	C	-11.73086200	-2.34817700	0.72999800
C	-8.79864700	-4.47545000	-1.41236000	C	-8.81517000	-4.48364500	-1.40931200
C	-12.21843800	-1.21957900	-1.41356100	C	-12.24253100	-1.22250500	-1.40934400
C	-12.21843800	-1.21957900	1.41356100	C	-12.24253100	-1.22250500	1.40934400
H	-8.78988600	-4.44469700	2.49921100	H	-8.80823000	-4.46061900	2.49641500
H	-8.78988600	-4.44469700	-2.49921100	H	-8.80823000	-4.46061900	-2.49641500
C	-12.38600700	0.00000000	0.73284800	C	-12.40054900	0.00000000	0.72984900
C	-12.38600700	0.00000000	-0.73284800	C	-12.40054900	0.00000000	-0.72984900
C	-12.21843800	1.21957900	-1.41356100	C	-12.24253100	1.22250500	-1.40934400
C	-12.21843800	1.21957900	1.41356100	C	-12.24253100	1.22250500	1.40934400
H	-10.91709500	-3.27407800	-2.49993100	H	-10.94623300	-3.28998800	-2.49671000
H	-10.91709500	-3.27407800	2.49993100	H	-10.94623300	-3.28998800	2.49671000
H	-12.18342200	-1.20974500	2.50022400	H	-12.22015900	-1.21624100	2.49650500
H	-12.18342200	-1.20974500	-2.50022400	H	-12.22015900	-1.21624100	-2.49650500
H	-12.18342200	1.20974500	-2.50022400	H	-12.22015900	1.21624100	-2.49650500
H	-12.18342200	1.20974500	2.50022400	H	-12.22015900	1.21624100	2.49650500
**Dcyc14_R**				**Dcyc14_U**			
C	-7.44295200	4.98683600	0.72838900	C	-7.43677000	5.00291200	0.72910000
C	-7.44295200	4.98683600	-0.72838900	C	-7.43677000	5.00291200	-0.72910000
C	-3.99558200	-3.78155800	1.40521800	C	-3.98668400	-3.79148600	1.40547100
C	-5.07245800	-4.38115300	0.72606200	C	-5.06362700	-4.39358900	0.72646000
C	-6.24042400	-4.78978500	1.40668300	C	-6.22779500	-4.80676500	1.40786300
C	-5.07245800	-4.38115300	-0.72606200	C	-5.06362700	-4.39358900	-0.72646000
C	-6.24042400	-4.78978500	-1.40668300	C	-6.22779500	-4.80676500	-1.40786300
C	-7.44295200	-4.98683600	-0.72838900	C	-7.43677000	-5.00291200	-0.72910000
C	-7.44295200	-4.98683600	0.72838900	C	-7.43677000	-5.00291200	0.72910000
C	-3.02504400	-3.04472900	0.72375200	C	-3.01824900	-3.05125600	0.72343900
C	-3.99558200	-3.78155800	-1.40521800	C	-3.98668400	-3.79148600	-1.40547100
H	-6.24579300	-4.76459300	-2.49398300	H	-6.23253300	-4.78391800	-2.49516600
C	-8.69080600	-4.95196700	-1.40751000	C	-8.67695400	-4.97239300	-1.40916100
C	-8.69080600	-4.95196700	1.40751000	C	-8.67695400	-4.97239300	1.40916100
H	-8.68666800	-4.92843000	-2.49484200	H	-8.67347500	-4.95108600	-2.49642700
H	-8.68666800	-4.92843000	2.49484200	H	-8.67347500	-4.95108600	2.49642700

C	-3.02504400	-3.04472900	-0.72375200	C	-3.01824900	-3.05125600	-0.72343900
H	-4.00959300	-3.75870400	2.49258000	H	-4.00006200	-3.76936500	2.49285000
H	-6.24579300	-4.76459300	2.49398300	H	-6.23253300	-4.78391800	2.49516600
H	-4.00959300	-3.75870400	-2.49258000	H	-4.00006200	-3.76936500	-2.49285000
C	-9.86927900	-4.66702400	0.73039200	C	-9.86595200	-4.67840300	0.73011600
C	-9.86927900	-4.66702400	-0.73039200	C	-9.86595200	-4.67840300	-0.73011600
C	-3.99558200	3.78155800	1.40521800	C	-3.98668400	3.79148600	1.40547100
C	-3.02504400	3.04472900	0.72375200	C	-3.01824900	3.05125600	0.72343900
C	-2.15680300	2.13431800	1.40449300	C	-2.15451600	2.13667100	1.40439600
C	-3.02504400	3.04472900	-0.72375200	C	-3.01824900	3.05125600	-0.72343900
C	-2.15680300	2.13431800	-1.40449300	C	-2.15451600	2.13667100	-1.40439600
C	-1.42745400	1.20182100	-0.71980200	C	-1.42691200	1.20266400	-0.71969600
C	-1.42745400	1.20182100	0.71980200	C	-1.42691200	1.20266400	0.71969600
C	-3.99558200	3.78155800	-1.40521800	C	-3.98668400	3.79148600	-1.40547100
C	-0.79343000	0.00000000	-1.39807400	C	-0.79376100	0.00000000	-1.39759700
C	-0.79343000	0.00000000	1.39807400	C	-0.79376100	0.00000000	1.39759700
H	-4.00959300	3.75870400	2.49258000	H	-4.00006200	3.76936500	2.49285000
H	-4.00959300	3.75870400	-2.49258000	H	-4.00006200	3.76936500	-2.49285000
C	-1.42745400	-1.20182100	0.71980200	C	-1.42691200	-1.20266400	0.71969600
C	-1.42745400	-1.20182100	-0.71980200	C	-1.42691200	-1.20266400	-0.71969600
C	-2.15680300	-2.13431800	-1.40449300	C	-2.15451600	-2.13667100	-1.40439600
C	-2.15680300	-2.13431800	1.40449300	C	-2.15451600	-2.13667100	1.40439600
H	-2.18884400	2.10802500	-2.49151400	H	-2.18756200	2.10975200	-2.49138300
H	-2.18884400	2.10802500	2.49151400	H	-2.18756200	2.10975200	2.49138300
H	-1.10132200	0.00000000	2.44686200	H	-1.10114100	0.00000000	2.44654500
H	-1.10132200	0.00000000	-2.44686200	H	-1.10114100	0.00000000	-2.44654500
H	-2.18884400	-2.10802500	-2.49151400	H	-2.18756200	-2.10975200	-2.49138300
H	-2.18884400	-2.10802500	2.49151400	H	-2.18756200	-2.10975200	2.49138300
C	-12.69328000	2.36226200	1.40639900	C	-12.66936500	2.36641900	1.40907600
C	-11.93049000	3.34917300	0.73200300	C	-11.92416100	3.35265900	0.72999600
C	-10.99896600	4.12771100	1.40733700	C	-10.98276300	4.14150000	1.40930300
C	-11.93049000	3.34917300	-0.73200300	C	-11.92416100	3.35265900	-0.72999600
C	-10.99896600	4.12771100	-1.40733700	C	-10.98276300	4.14150000	-1.40930300
C	-9.86927900	4.66702400	-0.73039200	C	-9.86595200	4.67840300	-0.73011600
C	-9.86927900	4.66702400	0.73039200	C	-9.86595200	4.67840300	0.73011600
C	-13.13506800	1.22184300	0.73295000	C	-13.12359200	1.22191400	0.72964500
C	-12.69328000	2.36226200	-1.40639900	C	-12.66936500	2.36641900	-1.40907600
H	-10.98885200	4.11349400	-2.49480600	H	-10.97108600	4.12377000	-2.49655200
C	-8.69080600	4.95196700	-1.40751000	C	-8.67695400	4.97239300	-1.40916100
C	-8.69080600	4.95196700	1.40751000	C	-8.67695400	4.97239300	1.40916100
H	-8.68666800	4.92843000	-2.49484200	H	-8.67347500	4.95108600	-2.49642700
H	-8.68666800	4.92843000	2.49484200	H	-8.67347500	4.95108600	2.49642700
C	-13.13506800	1.22184300	-0.73295000	C	-13.12359200	1.22191400	-0.72964500
H	-12.68513900	2.35820200	2.49398800	H	-12.65002100	2.35555100	2.49631300
H	-10.98885200	4.11349400	2.49480600	H	-10.97108600	4.12377000	2.49655200
H	-12.68513900	2.35820200	-2.49398800	H	-12.65002100	2.35555100	-2.49631300
C	-10.99896600	-4.12771100	1.40733700	C	-10.98276300	-4.14150000	1.40930300
C	-11.93049000	-3.34917300	0.73200300	C	-11.92416100	-3.35265900	0.72999600
C	-12.69328000	-2.36226200	1.40639900	C	-12.66936500	-2.36641900	1.40907600
C	-11.93049000	-3.34917300	-0.73200300	C	-11.92416100	-3.35265900	-0.72999600
C	-12.69328000	-2.36226200	-1.40639900	C	-12.66936500	-2.36641900	-1.40907600
C	-10.99896600	-4.12771100	-1.40733700	C	-10.98276300	-4.14150000	-1.40930300
H	-10.98885200	-4.11349400	2.49480600	H	-10.97108600	-4.12377000	2.49655200
H	-10.98885200	-4.11349400	-2.49480600	H	-10.97108600	-4.12377000	-2.49655200
C	-13.13506800	-1.22184300	0.73295000	C	-13.12359200	-1.22191400	0.72964500
C	-13.13506800	-1.22184300	-0.73295000	C	-13.12359200	-1.22191400	-0.72964500
C	-13.32268000	0.00000000	-1.40578100	C	-13.29332700	0.00000000	-1.40895600
C	-13.32268000	0.00000000	1.40578100	C	-13.29332700	0.00000000	1.40895600
H	-12.68513900	-2.35820200	-2.49398800	H	-12.65002100	-2.35555100	-2.49631300
H	-12.68513900	-2.35820200	2.49398800	H	-12.65002100	-2.35555100	2.49631300

H	-13.31768100	0.00000000	-2.49341000	H	-13.27054800	0.00000000	-2.49618400
H	-13.31768100	0.00000000	2.49341000	H	-13.27054800	0.00000000	2.49618400
C	-6.24042400	4.78978500	-1.40668300	C	-6.22779500	4.80676500	-1.40786300
C	-5.07245800	4.38115300	-0.72606200	C	-5.06362700	4.39358900	-0.72646000
C	-5.07245800	4.38115300	0.72606200	C	-5.06362700	4.39358900	0.72646000
C	-6.24042400	4.78978500	1.40668300	C	-6.22779500	4.80676500	1.40786300
H	-6.24579300	4.76459300	-2.49398300	H	-6.23253300	4.78391800	-2.49516600
H	-6.24579300	4.76459300	2.49398300	H	-6.23253300	4.78391800	2.49516600
C	5.07245800	4.38115300	0.72606200	C	5.06362700	4.39358900	0.72646000
C	5.07245800	4.38115300	-0.72606200	C	5.06362700	4.39358900	-0.72646000
C	12.69328000	-2.36226200	1.40639900	C	12.66936500	-2.36641900	1.40907600
C	11.93049000	-3.34917300	0.73200300	C	11.92416100	-3.35265900	0.72999600
C	10.99896600	-4.12771100	1.40733700	C	10.98276300	-4.14150000	1.40930300
C	11.93049000	-3.34917300	-0.73200300	C	11.92416100	-3.35265900	-0.72999600
C	10.99896600	-4.12771100	-1.40733700	C	10.98276300	-4.14150000	-1.40930300
C	9.86927900	-4.66702400	-0.73039200	C	9.86595200	-4.67840300	-0.73011600
C	9.86927900	-4.66702400	0.73039200	C	9.86595200	-4.67840300	0.73011600
C	13.13506800	-1.22184300	0.73295000	C	13.12359200	-1.22191400	0.72964500
C	12.69328000	-2.36226200	-1.40639900	C	12.66936500	-2.36641900	-1.40907600
H	10.98885200	-4.11349400	-2.49480600	H	10.97108600	-4.12377000	-2.49655200
C	8.69080600	-4.95196700	-1.40751000	C	8.67695400	-4.97239300	-1.40916100
C	8.69080600	-4.95196700	1.40751000	C	8.67695400	-4.97239300	1.40916100
H	8.68666800	-4.92843000	-2.49484200	H	8.67347500	-4.95108600	-2.49642700
H	8.68666800	-4.92843000	2.49484200	H	8.67347500	-4.95108600	2.49642700
C	13.13506800	-1.22184300	-0.73295000	C	13.12359200	-1.22191400	-0.72964500
H	12.68513900	-2.35820200	2.49398800	H	12.65002100	-2.35555100	2.49631300
H	10.98885200	-4.11349400	2.49480600	H	10.97108600	-4.12377000	2.49655200
H	12.68513900	-2.35820200	-2.49398800	H	12.65002100	-2.35555100	-2.49631300
C	7.44295200	-4.98683600	0.72838900	C	7.43677000	-5.00291200	0.72910000
C	7.44295200	-4.98683600	-0.72838900	C	7.43677000	-5.00291200	-0.72910000
C	8.69080600	4.95196700	1.40751000	C	8.67695400	4.97239300	1.40916100
C	9.86927900	4.66702400	0.73039200	C	9.86595200	4.67840300	0.73011600
C	10.99896600	4.12771100	1.40733700	C	10.98276300	4.14150000	1.40930300
C	9.86927900	4.66702400	-0.73039200	C	9.86595200	4.67840300	-0.73011600
C	10.99896600	4.12771100	-1.40733700	C	10.98276300	4.14150000	-1.40930300
C	11.93049000	3.34917300	-0.73200300	C	11.92416100	3.35265900	-0.72999600
C	11.93049000	3.34917300	0.73200300	C	11.92416100	3.35265900	0.72999600
C	8.69080600	4.95196700	-1.40751000	C	8.67695400	4.97239300	-1.40916100
C	12.69328000	2.36226200	-1.40639900	C	12.66936500	2.36641900	-1.40907600
C	12.69328000	2.36226200	1.40639900	C	12.66936500	2.36641900	1.40907600
H	8.68666800	4.92843000	2.49484200	H	8.67347500	4.95108600	2.49642700
H	8.68666800	4.92843000	-2.49484200	H	8.67347500	4.95108600	-2.49642700
C	13.13506800	1.22184300	0.73295000	C	13.12359200	1.22191400	0.72964500
C	13.13506800	1.22184300	-0.73295000	C	13.12359200	1.22191400	-0.72964500
C	13.32268000	0.00000000	-1.40578100	C	13.29332700	0.00000000	-1.40895600
C	13.32268000	0.00000000	1.40578100	C	13.29332700	0.00000000	1.40895600
H	10.98885200	4.11349400	-2.49480600	H	10.97108600	4.12377000	-2.49655200
H	10.98885200	4.11349400	2.49480600	H	10.97108600	4.12377000	2.49655200
H	12.68513900	2.35820200	2.49398800	H	12.65002100	2.35555100	2.49631300
H	12.68513900	2.35820200	-2.49398800	H	12.65002100	2.35555100	-2.49631300
H	13.31768100	0.00000000	-2.49341000	H	13.27054800	0.00000000	-2.49618400
H	13.31768100	0.00000000	2.49341000	H	13.27054800	0.00000000	2.49618400
C	0.79343000	0.00000000	1.39807400	C	0.79376100	0.00000000	1.39759700
C	1.42745400	1.20182100	0.71980200	C	1.42691200	1.20266400	0.71969600
C	2.15680300	2.13431800	1.40449300	C	2.15451600	2.13667100	1.40439600
C	1.42745400	1.20182100	-0.71980200	C	1.42691200	1.20266400	-0.71969600
C	2.15680300	2.13431800	-1.40449300	C	2.15451600	2.13667100	-1.40439600
C	3.02504400	3.04472900	-0.72375200	C	3.01824900	3.05125600	-0.72343900
C	3.02504400	3.04472900	0.72375200	C	3.01824900	3.05125600	0.72343900
C	1.42745400	-1.20182100	0.71980200	C	1.42691200	-1.20266400	0.71969600

C	0.79343000	0.00000000	-1.39807400	C	0.79376100	0.00000000	-1.39759700
H	2.18884400	2.10802500	-2.49151400	H	2.18756200	2.10975200	-2.49138300
C	3.99558200	3.78155800	-1.40521800	C	3.98668400	3.79148600	-1.40547100
C	3.99558200	3.78155800	1.40521800	C	3.98668400	3.79148600	1.40547100
H	4.00959300	3.75870400	-2.49258000	H	4.00006200	3.76936500	-2.49285000
H	4.00959300	3.75870400	2.49258000	H	4.00006200	3.76936500	2.49285000
C	1.42745400	-1.20182100	-0.71980200	C	1.42691200	-1.20266400	-0.71969600
H	1.10132200	0.00000000	2.44686200	H	1.10114100	0.00000000	2.44654500
H	2.18884400	2.10802500	2.49151400	H	2.18756200	2.10975200	2.49138300
H	1.10132200	0.00000000	-2.44686200	H	1.10114100	0.00000000	-2.44654500
C	6.24042400	-4.78978500	1.40668300	C	6.22779500	-4.80676500	1.40786300
C	5.07245800	-4.38115300	0.72606200	C	5.06362700	-4.39358900	0.72646000
C	3.99558200	-3.78155800	1.40521800	C	3.98668400	-3.79148600	1.40547100
C	5.07245800	-4.38115300	-0.72606200	C	5.06362700	-4.39358900	-0.72646000
C	3.99558200	-3.78155800	-1.40521800	C	3.98668400	-3.79148600	-1.40547100
C	6.24042400	-4.78978500	-1.40668300	C	6.22779500	-4.80676500	-1.40786300
H	6.24579300	-4.76459300	2.49398300	H	6.23253300	-4.78391800	2.49516600
H	6.24579300	-4.76459300	-2.49398300	H	6.23253300	-4.78391800	-2.49516600
C	3.02504400	-3.04472900	0.72375200	C	3.01824900	-3.05125600	0.72343900
C	3.02504400	-3.04472900	-0.72375200	C	3.01824900	-3.05125600	-0.72343900
C	2.15680300	-2.13431800	-1.40449300	C	2.15451600	-2.13667100	-1.40439600
C	2.15680300	-2.13431800	1.40449300	C	2.15451600	-2.13667100	1.40439600
H	4.00959300	-3.75870400	-2.49258000	H	4.00006200	-3.76936500	-2.49285000
H	4.00959300	-3.75870400	2.49258000	H	4.00006200	-3.76936500	2.49285000
H	2.18884400	-2.10802500	-2.49151400	H	2.18756200	-2.10975200	-2.49138300
H	2.18884400	-2.10802500	2.49151400	H	2.18756200	-2.10975200	2.49138300
C	6.24042400	4.78978500	-1.40668300	C	6.22779500	4.80676500	-1.40786300
C	7.44295200	4.98683600	-0.72838900	C	7.43677000	5.00291200	-0.72910000
C	7.44295200	4.98683600	0.72838900	C	7.43677000	5.00291200	0.72910000
C	6.24042400	4.78978500	1.40668300	C	6.22779500	4.80676500	1.40786300
H	6.24579300	4.76459300	-2.49398300	H	6.23253300	4.78391800	-2.49516600
H	6.24579300	4.76459300	2.49398300	H	6.23253300	4.78391800	2.49516600
**Dcyc15_R**				**Dcyc15_U**			
C	13.58050000	2.37476200	0.73297900	C	13.57335200	2.37555900	0.72956600
C	13.58050000	2.37476200	-0.73297900	C	13.57335200	2.37555900	-0.72956600
C	6.10677000	-4.98348400	1.40705900	C	6.09695600	-5.00087400	1.40782300
C	4.96974600	-4.50487100	0.72634200	C	4.96156800	-4.51517000	0.72646300
C	3.92051700	-3.85309100	1.40561300	C	3.91513500	-3.86161400	1.40543100
C	4.96974600	-4.50487100	-0.72634200	C	4.96156800	-4.51517000	-0.72646300
C	3.92051700	-3.85309100	-1.40561300	C	3.91513500	-3.86161400	-1.40543100
C	2.97988700	-3.08315200	-0.72443500	C	2.97472300	-3.08651300	-0.72345600
C	2.97988700	-3.08315200	0.72443500	C	2.97472300	-3.08651300	0.72345600
C	7.29203600	-5.27679400	0.72835400	C	7.28646900	-5.29283500	0.72911100
C	6.10677000	-4.98348400	-1.40705900	C	6.09695600	-5.00087400	-1.40782300
H	3.93412200	-3.83313900	-2.49326900	H	3.92830300	-3.84193800	-2.49289600
C	2.13703900	-2.14677000	-1.40538200	C	2.13454800	-2.15040400	-1.40446200
C	2.13703900	-2.14677000	1.40538200	C	2.13454800	-2.15040400	1.40446200
H	2.17026100	-2.12083000	-2.49238600	H	2.16653100	-2.12558700	-2.49154200
H	2.17026100	-2.12083000	2.49238600	H	2.16653100	-2.12558700	2.49154200
C	7.29203600	-5.27679400	-0.72835400	C	7.28646900	-5.29283500	-0.72911100
H	6.11367800	-4.96024500	2.49439700	H	6.10273100	-4.98059000	2.49516700
H	3.93412200	-3.83313900	2.49326900	H	3.92830300	-3.84193800	2.49289600
H	6.11367800	-4.96024500	-2.49439700	H	6.10273100	-4.98059000	-2.49516700
C	1.42384200	-1.20412900	0.72069100	C	1.42284600	-1.20460200	0.71966200
C	1.42384200	-1.20412900	-0.72069100	C	1.42284600	-1.20460200	-0.71966200
C	14.04310900	-1.22307600	1.40612400	C	14.01711200	-1.22463100	1.40874100
C	13.58050000	-2.37476200	0.73297900	C	13.57335200	-2.37555900	0.72956600
C	12.90823500	-3.39091100	1.40709700	C	12.89023200	-3.39978100	1.40893500

C	13.58050000	-2.37476200	-0.73297900
C	12.90823500	-3.39091100	-1.40709700
C	11.97716600	-4.22426700	-0.73179100
C	11.97716600	-4.22426700	0.73179100
C	14.04310900	-1.22307600	-1.40612400
C	10.93096900	-4.83673200	-1.40797300
C	10.93096900	-4.83673200	1.40797300
H	14.03668500	-1.22177400	2.49376200
H	14.03668500	-1.22177400	-2.49376200
C	9.73940200	-5.21723300	0.73018800
C	9.73940200	-5.21723300	-0.73018800
C	8.53449300	-5.36069800	-1.40801000
C	8.53449300	-5.36069800	1.40801000
H	12.89849100	-3.38321000	-2.49466000
H	12.89849100	-3.38321000	2.49466000
H	10.92213600	-4.81897200	2.49532600
H	10.92213600	-4.81897200	-2.49532600
H	8.53308100	-5.33726900	-2.49534500
H	8.53308100	-5.33726900	2.49534500
C	8.53449300	5.36069800	1.40801000
C	9.73940200	5.21723300	0.73018800
C	10.93096900	4.83673200	1.40797300
C	9.73940200	5.21723300	-0.73018800
C	10.93096900	4.83673200	-1.40797300
C	11.97716600	4.22426700	-0.73179100
C	11.97716600	4.22426700	0.73179100
C	7.29203600	5.27679400	0.72835400
C	8.53449300	5.36069800	-1.40801000
H	10.92213600	4.81897200	-2.49532600
C	12.90823500	3.39091100	-1.40709700
C	12.90823500	3.39091100	1.40709700
H	12.89849100	3.38321000	-2.49466000
H	12.89849100	3.38321000	2.49466000
C	7.29203600	5.27679400	-0.72835400
H	8.53308100	5.33726900	2.49534500
H	10.92213600	4.81897200	2.49532600
H	8.53308100	5.33726900	-2.49534500
C	0.79419300	0.00000000	1.39782700
C	1.42384200	1.20412900	0.72069100
C	2.13703900	2.14677000	1.40538200
C	1.42384200	1.20412900	-0.72069100
C	2.13703900	2.14677000	-1.40538200
C	2.97988700	3.08315200	-0.72443500
C	2.97988700	3.08315200	0.72443500
C	0.79419300	0.00000000	-1.39782700
C	3.92051700	3.85309100	-1.40561300
C	3.92051700	3.85309100	1.40561300
H	1.10083700	0.00000000	2.44696900
H	1.10083700	0.00000000	-2.44696900
C	4.96974600	4.50487100	0.72634200
C	4.96974600	4.50487100	-0.72634200
C	6.10677000	4.98348400	-1.40705900
C	6.10677000	4.98348400	1.40705900
H	2.17026100	2.12083000	-2.49238600
H	2.17026100	2.12083000	2.49238600
H	3.93412200	3.83313900	2.49326900
H	3.93412200	3.83313900	-2.49326900
H	6.11367800	4.96024500	-2.49439700
H	6.11367800	4.96024500	2.49439700
C	14.04310900	1.22307600	-1.40612400

C	13.57335200	-2.37555900	-0.72956600
C	12.89023200	-3.39978100	-1.40893500
C	11.97610900	-4.23079500	-0.72998800
C	11.97610900	-4.23079500	0.72998800
C	14.01711200	-1.22463100	-1.40874100
C	10.91858400	-4.85557400	-1.40917500
C	10.91858400	-4.85557400	1.40917500
H	13.99551600	-1.21933700	2.49599200
H	13.99551600	-1.21933700	-2.49599200
C	9.73869900	-5.23187900	0.73016900
C	9.73869900	-5.23187900	-0.73016900
C	8.52315300	-5.38306400	-1.40908800
C	8.52315300	-5.38306400	1.40908800
H	12.87450400	-3.38630900	-2.49616600
H	12.87450400	-3.38630900	2.49616600
H	10.91047400	-4.83807900	2.49650100
H	10.91047400	-4.83807900	-2.49650100
H	8.52210000	-5.36372400	-2.49640500
H	8.52210000	-5.36372400	2.49640500
C	8.52315300	5.38306400	1.40908800
C	9.73869900	5.23187900	0.73016900
C	10.91858400	4.85557400	1.40917500
C	9.73869900	5.23187900	-0.73016900
C	10.91858400	4.85557400	-1.40917500
C	11.97610900	4.23079500	-0.72998800
C	11.97610900	4.23079500	0.72998800
C	7.28646900	5.29283500	0.72911100
C	8.52315300	5.38306400	-1.40908800
H	10.91047400	4.83807900	-2.49650100
C	12.89023200	3.39978100	-1.40893500
C	12.89023200	3.39978100	1.40893500
H	12.87450400	3.38630900	-2.49616600
H	12.87450400	3.38630900	2.49616600
C	7.28646900	5.29283500	-0.72911100
H	8.52210000	5.36372400	2.49640500
H	10.91047400	4.83807900	2.49650100
H	8.52210000	5.36372400	-2.49640500
C	0.79460700	0.00000000	1.39757300
C	1.42284600	1.20460200	0.71966200
C	2.13454800	2.15040400	1.40446200
C	1.42284600	1.20460200	-0.71966200
C	2.13454800	2.15040400	-1.40446200
C	2.97472300	3.08651300	-0.72345600
C	2.97472300	3.08651300	0.72345600
C	0.79460700	0.00000000	-1.39757300
C	3.91513500	3.86161400	-1.40543100
C	3.91513500	3.86161400	1.40543100
H	1.10222700	0.00000000	2.44645000
H	1.10222700	0.00000000	-2.44645000
C	4.96156800	4.51517000	0.72646300
C	4.96156800	4.51517000	-0.72646300
C	6.09695600	5.00087400	-1.40782300
C	6.09695600	5.00087400	1.40782300
H	2.16653100	2.12558700	-2.49154200
H	2.16653100	2.12558700	2.49154200
H	3.92830300	3.84193800	2.49289600
H	3.92830300	3.84193800	-2.49289600
H	6.10273100	4.98059000	-2.49516700
H	6.10273100	4.98059000	2.49516700
C	14.01711200	1.22463100	-1.40874100

C	14.16694800	0.00000000	-0.73326700	C	14.15633800	0.00000000	-0.72928100
C	14.16694800	0.00000000	0.73326700	C	14.15633800	0.00000000	0.72928100
C	14.04310900	1.22307600	1.40612400	C	14.01711200	1.22463100	1.40874100
H	14.03668500	1.22177400	-2.49376200	H	13.99551600	1.21933700	-2.49599200
H	14.03668500	1.22177400	2.49376200	H	13.99551600	1.21933700	2.49599200
C	-1.42384200	1.20412900	0.72069100	C	-1.42284600	1.20460200	0.71966200
C	-1.42384200	1.20412900	-0.72069100	C	-1.42284600	1.20460200	-0.71966200
C	-10.93096900	-4.83673200	1.40797300	C	-10.91858400	-4.85557400	1.40917500
C	-11.97716600	-4.22426700	0.73179100	C	-11.97610900	-4.23079500	0.72998800
C	-12.90823500	-3.39091100	1.40709700	C	-12.89023200	-3.39978100	1.40893500
C	-11.97716600	-4.22426700	-0.73179100	C	-11.97610900	-4.23079500	-0.72998800
C	-12.90823500	-3.39091100	-1.40709700	C	-12.89023200	-3.39978100	-1.40893500
C	-13.58050000	-2.37476200	-0.73297900	C	-13.57335200	-2.37555900	-0.72956600
C	-13.58050000	-2.37476200	0.73297900	C	-13.57335200	-2.37555900	0.72956600
C	-9.73940200	-5.21723300	0.73018800	C	-9.73869900	-5.23187900	0.73016900
C	-10.93096900	-4.83673200	-1.40797300	C	-10.91858400	-4.85557400	-1.40917500
H	-12.89849100	-3.38321000	-2.49466000	H	-12.87450400	-3.38630900	-2.49616600
C	-14.04310900	-1.22307600	-1.40612400	C	-14.01711200	-1.22463100	-1.40874100
C	-14.04310900	-1.22307600	1.40612400	C	-14.01711200	-1.22463100	1.40874100
H	-14.03668500	-1.22177400	-2.49376200	H	-13.99551600	-1.21933700	-2.49599200
H	-14.03668500	-1.22177400	2.49376200	H	-13.99551600	-1.21933700	2.49599200
C	-9.73940200	-5.21723300	-0.73018800	C	-9.73869900	-5.23187900	-0.73016900
H	-10.92213600	-4.81897200	2.49532600	H	-10.91047400	-4.83807900	2.49650100
H	-12.89849100	-3.38321000	2.49466000	H	-12.87450400	-3.38630900	2.49616600
H	-10.92213600	-4.81897200	-2.49532600	H	-10.91047400	-4.83807900	-2.49650100
C	-14.16694800	0.00000000	0.73326700	C	-14.15633800	0.00000000	0.72928100
C	-14.16694800	0.00000000	-0.73326700	C	-14.15633800	0.00000000	-0.72928100
C	-2.13703900	-2.14677000	1.40538200	C	-2.13454800	-2.15040400	1.40446200
C	-2.97988700	-3.08315200	0.72443500	C	-2.97472300	-3.08651300	0.72345600
C	-3.92051700	-3.85309100	1.40561300	C	-3.91513500	-3.86161400	1.40543100
C	-2.97988700	-3.08315200	-0.72443500	C	-2.97472300	-3.08651300	-0.72345600
C	-3.92051700	-3.85309100	-1.40561300	C	-3.91513500	-3.86161400	-1.40543100
C	-4.96974600	-4.50487100	-0.72634200	C	-4.96156800	-4.51517000	-0.72646300
C	-4.96974600	-4.50487100	0.72634200	C	-4.96156800	-4.51517000	0.72646300
C	-2.13703900	-2.14677000	-1.40538200	C	-2.13454800	-2.15040400	-1.40446200
C	-6.10677000	-4.98348400	-1.40705900	C	-6.09695600	-5.00087400	-1.40782300
C	-6.10677000	-4.98348400	1.40705900	C	-6.09695600	-5.00087400	1.40782300
H	-2.17026100	-2.12083000	2.49238600	H	-2.16653100	-2.12558700	2.49154200
H	-2.17026100	-2.12083000	-2.49238600	H	-2.16653100	-2.12558700	-2.49154200
C	-7.29203600	-5.27679400	0.72835400	C	-7.28646900	-5.29283500	0.72911100
C	-7.29203600	-5.27679400	-0.72835400	C	-7.28646900	-5.29283500	-0.72911100
C	-8.53449300	-5.36069800	-1.40801000	C	-8.52315300	-5.38306400	-1.40908800
C	-8.53449300	-5.36069800	1.40801000	C	-8.52315300	-5.38306400	1.40908800
H	-3.93412200	-3.83313900	-2.49326900	H	-3.92830300	-3.84193800	-2.49289600
H	-3.93412200	-3.83313900	2.49326900	H	-3.92830300	-3.84193800	2.49289600
H	-6.11367800	-4.96024500	2.49439700	H	-6.10273100	-4.98059000	2.49516700
H	-6.11367800	-4.96024500	-2.49439700	H	-6.10273100	-4.98059000	-2.49516700
H	-8.53308100	-5.33726900	-2.49534500	H	-8.52210000	-5.36372400	-2.49640500
H	-8.53308100	-5.33726900	2.49534500	H	-8.52210000	-5.36372400	2.49640500
C	-6.10677000	4.98348400	1.40705900	C	-6.09695600	5.00087400	1.40782300
C	-4.96974600	4.50487100	0.72634200	C	-4.96156800	4.51517000	0.72646300
C	-3.92051700	3.85309100	1.40561300	C	-3.91513500	3.86161400	1.40543100
C	-4.96974600	4.50487100	-0.72634200	C	-4.96156800	4.51517000	-0.72646300
C	-3.92051700	3.85309100	-1.40561300	C	-3.91513500	3.86161400	-1.40543100
C	-2.97988700	3.08315200	-0.72443500	C	-2.97472300	3.08651300	-0.72345600
C	-2.97988700	3.08315200	0.72443500	C	-2.97472300	3.08651300	0.72345600
C	-7.29203600	5.27679400	0.72835400	C	-7.28646900	5.29283500	0.72911100
C	-6.10677000	4.98348400	-1.40705900	C	-6.09695600	5.00087400	-1.40782300
H	-3.93412200	3.83313900	-2.49326900	H	-3.92830300	3.84193800	-2.49289600
C	-2.13703900	2.14677000	-1.40538200	C	-2.13454800	2.15040400	-1.40446200

C	-2.13703900	2.14677000	1.40538200	C	-2.13454800	2.15040400	1.40446200
H	-2.17026100	2.12083000	-2.49238600	H	-2.16653100	2.12558700	-2.49154200
H	-2.17026100	2.12083000	2.49238600	H	-2.16653100	2.12558700	2.49154200
C	-7.29203600	5.27679400	-0.72835400	C	-7.28646900	5.29283500	-0.72911100
H	-6.11367800	4.96024500	2.49439700	H	-6.10273100	4.98059000	2.49516700
H	-3.93412200	3.83313900	2.49326900	H	-3.92830300	3.84193800	2.49289600
H	-6.11367800	4.96024500	-2.49439700	H	-6.10273100	4.98059000	-2.49516700
C	-14.04310900	1.22307600	1.40612400	C	-14.01711200	1.22463100	1.40874100
C	-13.58050000	2.37476200	0.73297900	C	-13.57335200	2.37555900	0.72956600
C	-12.90823500	3.39091100	1.40709700	C	-12.89023200	3.39978100	1.40893500
C	-13.58050000	2.37476200	-0.73297900	C	-13.57335200	2.37555900	-0.72956600
C	-12.90823500	3.39091100	-1.40709700	C	-12.89023200	3.39978100	-1.40893500
C	-11.97716600	4.22426700	-0.73179100	C	-11.97610900	4.23079500	-0.72998800
C	-11.97716600	4.22426700	0.73179100	C	-11.97610900	4.23079500	0.72998800
C	-14.04310900	1.22307600	-1.40612400	C	-14.01711200	1.22463100	-1.40874100
C	-10.93096900	4.83673200	-1.40797300	C	-10.91858400	4.85557400	-1.40917500
C	-10.93096900	4.83673200	1.40797300	C	-10.91858400	4.85557400	1.40917500
H	-14.03668500	1.22177400	2.49376200	H	-13.99551600	1.21933700	2.49599200
H	-14.03668500	1.22177400	-2.49376200	H	-13.99551600	1.21933700	-2.49599200
C	-9.73940200	5.21723300	0.73018800	C	-9.73869900	5.23187900	0.73016900
C	-9.73940200	5.21723300	-0.73018800	C	-9.73869900	5.23187900	-0.73016900
C	-8.53449300	5.36069800	-1.40801000	C	-8.52315300	5.38306400	-1.40908800
C	-8.53449300	5.36069800	1.40801000	C	-8.52315300	5.38306400	1.40908800
H	-12.89849100	3.38321000	-2.49466000	H	-12.87450400	3.38630900	-2.49616600
H	-12.89849100	3.38321000	2.49466000	H	-12.87450400	3.38630900	2.49616600
H	-10.92213600	4.81897200	2.49532600	H	-10.91047400	4.83807900	2.49650100
H	-10.92213600	4.81897200	-2.49532600	H	-10.91047400	4.83807900	-2.49650100
H	-8.53308100	5.33726900	-2.49534500	H	-8.52210000	5.36372400	-2.49640500
H	-8.53308100	5.33726900	2.49534500	H	-8.52210000	5.36372400	2.49640500
C	-0.79419300	0.00000000	-1.39782700	C	-0.79460700	0.00000000	-1.39757300
C	-1.42384200	-1.20412900	-0.72069100	C	-1.42284600	-1.20460200	-0.71966200
C	-1.42384200	-1.20412900	0.72069100	C	-1.42284600	-1.20460200	0.71966200
C	-0.79419300	0.00000000	1.39782700	C	-0.79460700	0.00000000	1.39757300
H	-1.10083700	0.00000000	-2.44696900	H	-1.10222600	0.00000000	-2.44645000
H	-1.10083700	0.00000000	2.44696900	H	-1.10222700	0.00000000	2.44645000
**Dcyc16_R**				**Dcyc16_U**			
C	-11.94728800	4.98028600	0.73164300	C	-11.94447800	4.99267300	0.73019300
C	-11.94728800	4.98028600	-0.73164300	C	-11.94447800	4.99267300	-0.73019300
C	-0.79494000	0.00000000	1.39880200	C	-0.79527900	0.00000000	1.39788300
C	-1.42078900	-1.20508400	0.72120800	C	-1.41947200	-1.20597800	0.71966400
C	-2.11991800	-2.15743800	1.40573700	C	-2.11761700	-2.16150600	1.40454600
C	-1.42078900	-1.20508400	-0.72120800	C	-1.41947200	-1.20597800	-0.71966400
C	-2.11991800	-2.15743800	-1.40573700	C	-2.11761700	-2.16150600	-1.40454600
C	-2.94750600	-3.10811000	-0.72522600	C	-2.93880000	-3.11406600	-0.72349300
C	-2.94750600	-3.10811000	0.72522600	C	-2.93880000	-3.11406600	0.72349300
C	-1.42078900	1.20508400	0.72120800	C	-1.41947200	1.20597800	0.71966400
C	-0.79494000	0.00000000	-1.39880200	C	-0.79527900	0.00000000	-1.39788300
H	-2.14950600	-2.13501200	-2.49288400	H	-2.14760500	-2.13890300	-2.49168200
C	-3.86182700	-3.90651700	-1.40630800	C	-3.85413700	-3.91834000	-1.40544600
C	-3.86182700	-3.90651700	1.40630800	C	-3.85413700	-3.91834000	1.40544600
H	-3.87506300	-3.88839700	-2.49375100	H	-3.86670300	-3.90052000	-2.49291400
H	-3.87506300	-3.88839700	2.49375100	H	-3.86670300	-3.90052000	2.49291400
C	-1.42078900	1.20508400	-0.72120800	C	-1.41947200	1.20597800	-0.71966400
H	-1.10235800	0.00000000	2.44771500	H	-1.10286800	0.00000000	2.44677400
H	-2.14950600	-2.13501200	2.49288400	H	-2.14760500	-2.13890300	2.49168200
H	-1.10235800	0.00000000	-2.44771500	H	-1.10286800	0.00000000	-2.44677400
C	-4.88469100	-4.60191900	0.72723400	C	-4.87215400	-4.61502900	0.72661100
C	-4.88469100	-4.60191900	-0.72723400	C	-4.87215400	-4.61502900	-0.72661100

C	-8.38436900	5.69987000	1.40830600	C	-8.37048000	5.72700600	1.40900400
C	-7.15661900	5.51327700	0.72896200	C	-7.14596900	5.53396500	0.72929200
C	-5.99112100	5.14133300	1.40757500	C	-5.97919600	5.16183900	1.40779300
C	-7.15661900	5.51327700	-0.72896200	C	-7.14596900	5.53396500	-0.72929200
C	-5.99112100	5.14133300	-1.40757500	C	-5.97919600	5.16183900	-1.40779300
C	-4.88469100	4.60191900	-0.72723400	C	-4.87215400	4.61502900	-0.72661100
C	-4.88469100	4.60191900	0.72723400	C	-4.87215400	4.61502900	0.72661100
C	-8.38436900	5.69987000	-1.40830600	C	-8.37048000	5.72700600	-1.40900400
C	-3.86182700	3.90651700	-1.40630800	C	-3.85413700	3.91834000	-1.40544600
C	-3.86182700	3.90651700	1.40630800	C	-3.85413700	3.91834000	1.40544600
H	-8.38512600	5.67766600	2.49565300	H	-8.37107500	5.70954900	2.49637100
H	-8.38512600	5.67766600	-2.49565300	H	-8.37107500	5.70954900	-2.49637100
C	-2.94750600	3.10811000	0.72522600	C	-2.93880000	3.11406600	0.72349300
C	-2.94750600	3.10811000	-0.72522600	C	-2.93880000	3.11406600	-0.72349300
C	-2.11991800	2.15743800	-1.40573700	C	-2.11761700	2.16150600	-1.40454600
C	-2.11991800	2.15743800	1.40573700	C	-2.11761700	2.16150600	1.40454600
H	-5.99859600	5.12073100	-2.49492600	H	-5.98534900	5.14369800	-2.49515100
H	-5.99859600	5.12073100	2.49492600	H	-5.98534900	5.14369800	2.49515100
H	-3.87506300	3.88839700	2.49375100	H	-3.86670300	3.90052000	2.49291400
H	-3.87506300	3.88839700	-2.49375100	H	-3.86670300	3.90052000	-2.49291400
H	-2.14950600	2.13501200	-2.49288400	H	-2.14760500	2.13890300	-2.49168200
H	-2.14950600	2.13501200	2.49288400	H	-2.14760500	2.13890300	2.49168200
C	-15.06976000	0.00000000	1.40638300	C	-15.04010200	0.00000000	1.40850200
C	-14.90826200	1.22389200	0.73320300	C	-14.89354900	1.22367000	0.72926600
C	-14.51812200	2.38372500	1.40686000	C	-14.49285800	2.38898000	1.40857600
C	-14.90826200	1.22389200	-0.73320300	C	-14.89354900	1.22367000	-0.72926600
C	-14.51812200	2.38372500	-1.40686000	C	-14.49285800	2.38898000	-1.40857600
C	-13.84016700	3.42675400	-0.73262100	C	-13.83206600	3.42983900	-0.72965500
C	-13.84016700	3.42675400	0.73262100	C	-13.83206600	3.42983900	0.72965500
C	-14.90826200	-1.22389200	0.73320300	C	-14.89354900	-1.22367000	0.72926600
C	-15.06976000	0.00000000	-1.40638300	C	-15.04010200	0.00000000	-1.40850200
H	-14.50920100	2.37976200	-2.49447300	H	-14.47410800	2.37988100	-2.49589400
C	-12.99488100	4.30050600	-1.40780600	C	-12.97721200	4.31579600	-1.40881800
C	-12.99488100	4.30050600	1.40780600	C	-12.97721200	4.31579600	1.40881800
H	-12.98467400	4.28893800	-2.49531500	H	-12.96519900	4.30115800	-2.49615200
H	-12.98467400	4.28893800	2.49531500	H	-12.96519900	4.30115800	2.49615200
C	-14.90826200	-1.22389200	-0.73320300	C	-14.89354900	-1.22367000	-0.72926600
H	-15.06262800	0.00000000	2.49402000	H	-15.01850800	0.00000000	2.49578700
H	-14.50920100	2.37976200	2.49447300	H	-14.47410800	2.37988100	2.49589400
H	-15.06262800	0.00000000	-2.49402000	H	-15.01850800	0.00000000	-2.49578700
C	-5.99112100	-5.14133300	1.40757500	C	-5.97919600	-5.16183900	1.40779300
C	-10.82480100	-5.44003400	1.40838800	C	-10.81061600	-5.46507300	1.40909600
C	-10.82480100	-5.44003400	-1.40838800	C	-10.81061600	-5.46507300	-1.40909600
C	-11.94728800	-4.98028600	-0.73164300	C	-11.94447800	-4.99267300	-0.73019300
C	-11.94728800	-4.98028600	0.73164300	C	-11.94447800	-4.99267300	0.73019300
C	-5.99112100	-5.14133300	-1.40757500	C	-5.97919600	-5.16183900	-1.40779300
C	-12.99488100	-4.30050600	-1.40780600	C	-12.97721200	-4.31579600	-1.40881800
C	-12.99488100	-4.30050600	1.40780600	C	-12.97721200	-4.31579600	1.40881800
H	-5.99859600	-5.12073100	2.49492600	H	-5.98534900	-5.14369800	2.49515100
H	-5.99859600	-5.12073100	-2.49492600	H	-5.98534900	-5.14369800	-2.49515100
C	-13.84016700	-3.42675400	0.73262100	C	-13.83206600	-3.42983900	0.72965500
C	-13.84016700	-3.42675400	-0.73262100	C	-13.83206600	-3.42983900	-0.72965500
C	-14.51812200	-2.38372500	-1.40686000	C	-14.49285800	-2.38898000	-1.40857600
C	-14.51812200	-2.38372500	1.40686000	C	-14.49285800	-2.38898000	1.40857600
H	-10.81838000	-5.42092000	-2.49579100	H	-10.80549000	-5.44849500	-2.49645300
H	-10.81838000	-5.42092000	2.49579100	H	-10.80549000	-5.44849500	2.49645300
H	-12.98467400	-4.28893800	2.49531500	H	-12.96519900	-4.30115800	2.49615200
H	-12.98467400	-4.28893800	-2.49531500	H	-12.96519900	-4.30115800	-2.49615200
H	-14.50920100	-2.37976200	-2.49447300	H	-14.47410800	-2.37988100	-2.49589400
H	-14.50920100	-2.37976200	2.49447300	H	-14.47410800	-2.37988100	2.49589400

C	-10.82480100	5.44003400	-1.40838800	C	-10.81061600	5.46507300	-1.40909600
C	-9.59984400	5.67843200	-0.73040000	C	-9.59502700	5.69915400	-0.73034900
C	-9.59984400	5.67843200	0.73040000	C	-9.59502700	5.69915400	0.73034900
C	-10.82480100	5.44003400	1.40838800	C	-10.81061600	5.46507300	1.40909600
H	-10.81838000	5.42092000	-2.49579100	H	-10.80549000	5.44849500	-2.49645300
H	-10.81838000	5.42092000	2.49579100	H	-10.80549000	5.44849500	2.49645300
C	-9.59984400	-5.67843200	-0.73040000	C	-9.59502700	-5.69915400	-0.73034900
C	-8.38436900	-5.69987000	-1.40830600	C	-8.37048000	-5.72700600	-1.40900400
C	-7.15661900	-5.51327700	-0.72896200	C	-7.14596900	-5.53396500	-0.72929200
C	-7.15661900	-5.51327700	0.72896200	C	-7.14596900	-5.53396500	0.72929200
C	-8.38436900	-5.69987000	1.40830600	C	-8.37048000	-5.72700600	1.40900400
C	-9.59984400	-5.67843200	0.73040000	C	-9.59502700	-5.69915400	0.73034900
H	-8.38512600	-5.67766600	-2.49565300	H	-8.37107500	-5.70954900	-2.49637100
H	-8.38512600	-5.67766600	2.49565300	H	-8.37107500	-5.70954900	2.49637100
C	4.88469100	4.60191900	0.72723400	C	4.87215400	4.61502900	0.72661100
C	4.88469100	4.60191900	-0.72723400	C	4.87215400	4.61502900	-0.72661100
C	15.06976000	0.00000000	1.40638300	C	15.04010200	0.00000000	1.40850200
C	14.90826200	-1.22389200	0.73320300	C	14.89354900	-1.22367000	0.72926600
C	14.51812200	-2.38372500	1.40686000	C	14.49285800	-2.38898000	1.40857600
C	14.90826200	-1.22389200	-0.73320300	C	14.89354900	-1.22367000	-0.72926600
C	14.51812200	-2.38372500	-1.40686000	C	14.49285800	-2.38898000	-1.40857600
C	13.84016700	-3.42675400	-0.73262100	C	13.83206600	-3.42983900	-0.72965500
C	13.84016700	-3.42675400	0.73262100	C	13.83206600	-3.42983900	0.72965500
C	14.90826200	1.22389200	0.73320300	C	14.89354900	1.22367000	0.72926600
C	15.06976000	0.00000000	-1.40638300	C	15.04010200	0.00000000	-1.40850200
H	14.50920100	-2.37976200	-2.49447300	H	14.47410800	-2.37988100	-2.49589400
C	12.99488100	-4.30050600	-1.40780600	C	12.97721200	-4.31579600	-1.40881800
C	12.99488100	-4.30050600	1.40780600	C	12.97721200	-4.31579600	1.40881800
H	12.98467400	-4.28893800	-2.49531500	H	12.96519900	-4.30115800	-2.49615200
H	12.98467400	-4.28893800	2.49531500	H	12.96519900	-4.30115800	2.49615200
C	14.90826200	1.22389200	-0.73320300	C	14.89354900	1.22367000	-0.72926600
H	15.06262800	0.00000000	2.49402000	H	15.01850800	0.00000000	2.49578700
H	14.50920100	-2.37976200	2.49447300	H	14.47410800	-2.37988100	2.49589400
H	15.06262800	0.00000000	-2.49402000	H	15.01850800	0.00000000	-2.49578700
C	11.94728800	-4.98028600	0.73164300	C	11.94447800	-4.99267300	0.73019300
C	11.94728800	-4.98028600	-0.73164300	C	11.94447800	-4.99267300	-0.73019300
C	8.38436900	5.69987000	1.40830600	C	8.37048000	5.72700600	1.40900400
C	9.59984400	5.67843200	0.73040000	C	9.59502700	5.69915400	0.73034900
C	10.82480100	5.44003400	1.40838800	C	10.81061600	5.46507300	1.40909600
C	9.59984400	5.67843200	-0.73040000	C	9.59502700	5.69915400	-0.73034900
C	10.82480100	5.44003400	-1.40838800	C	10.81061600	5.46507300	-1.40909600
C	11.94728800	4.98028600	-0.73164300	C	11.94447800	4.99267300	-0.73019300
C	11.94728800	4.98028600	0.73164300	C	11.94447800	4.99267300	0.73019300
C	8.38436900	5.69987000	-1.40830600	C	8.37048000	5.72700600	-1.40900400
C	12.99488100	4.30050600	-1.40780600	C	12.97721200	4.31579600	-1.40881800
C	12.99488100	4.30050600	1.40780600	C	12.97721200	4.31579600	1.40881800
H	8.38512600	5.67766600	2.49565300	H	8.37107500	5.70954900	2.49637100
H	8.38512600	5.67766600	-2.49565300	H	8.37107500	5.70954900	-2.49637100
C	13.84016700	3.42675400	0.73262100	C	13.83206600	3.42983900	0.72965500
C	13.84016700	3.42675400	-0.73262100	C	13.83206600	3.42983900	-0.72965500
C	14.51812200	2.38372500	-1.40686000	C	14.49285800	2.38898000	-1.40857500
C	14.51812200	2.38372500	1.40686000	C	14.49285800	2.38898000	1.40857600
H	10.81838000	5.42092000	-2.49579100	H	10.80549000	5.44849500	-2.49645300
H	10.81838000	5.42092000	2.49579100	H	10.80549000	5.44849500	2.49645300
H	12.98467400	4.28893800	2.49531500	H	12.96519900	4.30115800	2.49615200
H	12.98467400	4.28893800	-2.49531500	H	12.96519900	4.30115800	-2.49615200
H	14.50920100	2.37976200	-2.49447300	H	14.47410800	2.37988100	-2.49589400
H	14.50920100	2.37976200	2.49447300	H	14.47410800	2.37988100	2.49589400
C	0.79494000	0.00000000	1.39880200	C	0.79527900	0.00000000	1.39788300
C	1.42078900	1.20508400	0.72120800	C	1.41947200	1.20597800	0.71966400

C	2.11991800	2.15743800	1.40573700	C	2.11761700	2.16150600	1.40454600
C	1.42078900	1.20508400	-0.72120800	C	1.41947200	1.20597800	-0.71966400
C	2.11991800	2.15743800	-1.40573700	C	2.11761700	2.16150600	-1.40454600
C	2.94750600	3.10811000	-0.72522600	C	2.93880000	3.11406600	-0.72349300
C	2.94750600	3.10811000	0.72522600	C	2.93880000	3.11406600	0.72349300
C	1.42078900	-1.20508400	0.72120800	C	1.41947200	-1.20597800	0.71966400
C	0.79494000	0.00000000	-1.39880200	C	0.79527900	0.00000000	-1.39788300
H	2.14950600	2.13501200	-2.49288400	H	2.14760500	2.13890300	-2.49168200
C	3.86182700	3.90651700	-1.40630800	C	3.85413700	3.91834000	-1.40544600
C	3.86182700	3.90651700	1.40630800	C	3.85413700	3.91834000	1.40544600
H	3.87506300	3.88839700	-2.49375100	H	3.86670300	3.90052000	-2.49291400
H	3.87506300	3.88839700	2.49375100	H	3.86670300	3.90052000	2.49291400
C	1.42078900	-1.20508400	-0.72120800	C	1.41947200	-1.20597800	-0.71966400
H	1.10235800	0.00000000	2.44771500	H	1.10286800	0.00000000	2.44677400
H	2.14950600	2.13501200	2.49288400	H	2.14760500	2.13890300	2.49168200
H	1.10235800	0.00000000	-2.44771500	H	1.10286800	0.00000000	-2.44677400
C	10.82480100	-5.44003400	1.40838800	C	10.81061600	-5.46507300	1.40909600
C	5.99112100	-5.14133300	1.40757500	C	5.97919600	-5.16183900	1.40779300
C	5.99112100	-5.14133300	-1.40757500	C	5.97919600	-5.16183900	-1.40779300
C	4.88469100	-4.60191900	-0.72723400	C	4.87215400	-4.61502900	-0.72661100
C	4.88469100	-4.60191900	0.72723400	C	4.87215400	-4.61502900	0.72661100
C	10.82480100	-5.44003400	-1.40838800	C	10.81061600	-5.46507300	-1.40909600
C	3.86182700	-3.90651700	-1.40630800	C	3.85413700	-3.91834000	-1.40544600
C	3.86182700	-3.90651700	1.40630800	C	3.85413700	-3.91834000	1.40544600
H	10.81838000	-5.42092000	2.49579100	H	10.80549000	-5.44849500	2.49645300
H	10.81838000	-5.42092000	-2.49579100	H	10.80549000	-5.44849500	-2.49645300
C	2.94750600	-3.10811000	0.72522600	C	2.93880000	-3.11406600	0.72349300
C	2.94750600	-3.10811000	-0.72522600	C	2.93880000	-3.11406600	-0.72349300
C	2.11991800	-2.15743800	-1.40573700	C	2.11761700	-2.16150600	-1.40454600
C	2.11991800	-2.15743800	1.40573700	C	2.11761700	-2.16150600	1.40454600
H	5.99859600	-5.12073100	-2.49492600	H	5.98534900	-5.14369800	-2.49515100
H	5.99859600	-5.12073100	2.49492600	H	5.98534900	-5.14369800	2.49515100
H	3.87506300	-3.88839700	2.49375100	H	3.86670300	-3.90052000	2.49291400
H	3.87506300	-3.88839700	-2.49375100	H	3.86670300	-3.90052000	-2.49291400
H	2.14950600	-2.13501200	-2.49288400	H	2.14760500	-2.13890300	-2.49168200
H	2.14950600	-2.13501200	2.49288400	H	2.14760500	-2.13890300	2.49168200
C	5.99112100	5.14133300	-1.40757500	C	5.97919600	5.16183900	-1.40779300
C	7.15661900	5.51327700	-0.72896200	C	7.14596900	5.53396500	-0.72929200
C	7.15661900	5.51327700	0.72896200	C	7.14596900	5.53396500	0.72929200
C	5.99112100	5.14133300	1.40757500	C	5.97919600	5.16183900	1.40779300
H	5.99859600	5.12073100	-2.49492600	H	5.98534900	5.14369800	-2.49515100
H	5.99859600	5.12073100	2.49492600	H	5.98534900	5.14369800	2.49515100
C	7.15661900	-5.51327700	-0.72896200	C	7.14596900	-5.53396500	-0.72929200
C	8.38436900	-5.69987000	-1.40830600	C	8.37048000	-5.72700600	-1.40900400
C	9.59984400	-5.67843200	-0.73040000	C	9.59502700	-5.69915400	-0.73034900
C	9.59984400	-5.67843200	0.73040000	C	9.59502700	-5.69915400	0.73034900
C	8.38436900	-5.69987000	1.40830600	C	8.37048000	-5.72700600	1.40900400
C	7.15661900	-5.51327700	0.72896200	C	7.14596900	-5.53396500	0.72929200
H	8.38512600	-5.67766600	-2.49565300	H	8.37107500	-5.70954900	-2.49637100
H	8.38512600	-5.67766600	2.49565300	H	8.37107500	-5.70954900	2.49637100
**Dcyc17_R**				**Dcyc17_U**			
C	-15.80714400	1.22406300	1.40658400	C	-15.77837600	1.22609700	1.40838400
C	-15.39965700	2.39420000	0.73315700	C	-15.38720800	2.39426100	0.72929900
C	-14.79731000	3.45445300	1.40741300	C	-14.77538900	3.46406500	1.40847800
C	-15.39965700	2.39420000	-0.73315700	C	-15.38720800	2.39426100	-0.72929900
C	-14.79731000	3.45445300	-1.40741300	C	-14.77538900	3.46406500	-1.40847800
C	-13.95259800	4.37084900	-0.73247600	C	-13.94806100	4.37742600	-0.72973200
C	-13.95259800	4.37084900	0.73247600	C	-13.94806100	4.37742600	0.72973200

C	-15.91844000	0.00000000	0.73341900	C	-15.90179100	0.00000000	0.72914600
C	-15.80714400	1.22406300	-1.40658400	C	-15.77837600	1.22609700	-1.40838400
H	-14.78733300	3.44765000	-2.49499200	H	-14.75978900	3.45264900	-2.49582000
C	-12.98330400	5.10164500	-1.40834000	C	-12.96776200	5.12238800	-1.40869800
C	-12.98330400	5.10164500	1.40834000	C	-12.96776200	5.12238800	1.40869800
H	-12.97401900	5.08736900	-2.49581700	H	-12.95883500	5.10764900	-2.49607600
H	-12.97401900	5.08736900	2.49581700	H	-12.95883500	5.10764900	2.49607600
C	-15.91844000	0.00000000	-0.73341900	C	-15.90179100	0.00000000	-0.72914600
H	-15.79931300	1.22251700	2.49422700	H	-15.75822900	1.22173800	2.49569700
H	-14.78733300	3.44765000	2.49499200	H	-14.75978900	3.45264900	2.49582000
H	-15.79931300	1.22251700	-2.49422700	H	-15.75822900	1.22173800	-2.49569700
C	-11.85520100	5.63682200	0.73151700	C	-11.85380600	5.65412900	0.73024800
C	-11.85520100	5.63682200	-0.73151700	C	-11.85380600	5.65412900	-0.73024800
C	-10.68617000	-5.96008900	1.40879900	C	-10.67210600	-5.98928800	1.40898000
C	-11.85520100	-5.63682200	0.73151700	C	-11.85380600	-5.65412900	0.73024800
C	-12.98330400	-5.10164500	1.40834000	C	-12.96776200	-5.12238800	1.40869800
C	-11.85520100	-5.63682200	-0.73151700	C	-11.85380600	-5.65412900	-0.73024800
C	-12.98330400	-5.10164500	-1.40834000	C	-12.96776200	-5.12238800	-1.40869800
C	-13.95259800	-4.37084900	-0.73247600	C	-13.94806100	-4.37742600	-0.72973200
C	-13.95259800	-4.37084900	0.73247600	C	-13.94806100	-4.37742600	0.72973200
C	-10.68617000	-5.96008900	-1.40879900	C	-10.67210600	-5.98928800	-1.40898000
C	-14.79731000	-3.45445300	-1.40741300	C	-14.77538900	-3.46406500	-1.40847800
C	-14.79731000	-3.45445300	1.40741300	C	-14.77538900	-3.46406500	1.40847800
H	-10.68193100	-5.94020800	2.49618600	H	-10.66913500	-5.97376900	2.49637600
H	-10.68193100	-5.94020800	-2.49618600	H	-10.66913500	-5.97376900	-2.49637600
C	-15.39965700	-2.39420000	0.73315700	C	-15.38720800	-2.39426100	0.72929900
C	-15.39965700	-2.39420000	-0.73315700	C	-15.38720800	-2.39426100	-0.72929900
C	-15.80714400	-1.22406300	-1.40658400	C	-15.77837600	-1.22609700	-1.40838400
C	-15.80714400	-1.22406300	1.40658400	C	-15.77837600	-1.22609700	1.40838400
H	-12.97401900	-5.08736900	-2.49581700	H	-12.95883500	-5.10764900	-2.49607600
H	-12.97401900	-5.08736900	2.49581700	H	-12.95883500	-5.10764900	2.49607600
H	-14.78733300	-3.44765000	2.49499200	H	-14.75978900	-3.45264900	2.49582000
H	-14.78733300	-3.44765000	-2.49499200	H	-14.75978900	-3.45264900	-2.49582000
H	-15.79931300	-1.22251700	-2.49422700	H	-15.75822900	-1.22173800	-2.49569700
H	-15.79931300	-1.22251700	2.49422700	H	-15.75822900	-1.22173800	2.49569700
C	-0.79571300	0.00000000	1.39862800	C	-0.79620800	0.00000000	1.39788500
C	-1.41817600	-1.20701300	0.72182200	C	-1.41606400	-1.20751600	0.71957300
C	-2.10459400	-2.16718100	1.40624700	C	-2.10106100	-2.17245500	1.40451500
C	-1.41817600	-1.20701300	-0.72182200	C	-1.41606400	-1.20751600	-0.71957300
C	-2.10459400	-2.16718100	-1.40624700	C	-2.10106100	-2.17245500	-1.40451500
C	-2.91231900	-3.13626100	-0.72566000	C	-2.90342300	-3.14060100	-0.72350900
C	-2.91231900	-3.13626100	0.72566000	C	-2.90342300	-3.14060100	0.72350900
C	-1.41817600	1.20701300	0.72182200	C	-1.41606400	1.20751600	0.71957300
C	-0.79571300	0.00000000	-1.39862800	C	-0.79620800	0.00000000	-1.39788500
H	-2.13453800	-2.14535700	-2.49342000	H	-2.12969800	-2.15163500	-2.49176000
C	-3.80244100	-3.95865200	-1.40672200	C	-3.79559000	-3.97046000	-1.40543800
C	-3.80244100	-3.95865200	1.40672200	C	-3.79559000	-3.97046000	1.40543800
H	-3.81509100	-3.94253900	-2.49420700	H	-3.80780800	-3.95427500	-2.49293800
H	-3.81509100	-3.94253900	2.49420700	H	-3.80780800	-3.95427500	2.49293800
C	-1.41817600	1.20701300	-0.72182200	C	-1.41606400	1.20751600	-0.71957300
H	-1.10229500	0.00000000	2.44777900	H	-1.10381200	0.00000000	2.44676600
H	-2.13453800	-2.14535700	2.49342000	H	-2.12969800	-2.15163500	2.49176000
H	-1.10229500	0.00000000	-2.44777900	H	-1.10381200	0.00000000	-2.44676600
C	-10.68617000	5.96008900	1.40879900	C	-10.67210600	5.98928800	1.40898000
C	-5.87825800	5.28090000	1.40797500	C	-5.86693200	5.30259600	1.40773200
C	-5.87825800	5.28090000	-1.40797500	C	-5.86693200	5.30259600	-1.40773200
C	-4.80053800	4.69176000	-0.72759000	C	-4.78707700	4.70410900	-0.72660900
C	-4.80053800	4.69176000	0.72759000	C	-4.78707700	4.70410900	0.72660900
C	-10.68617000	5.96008900	-1.40879900	C	-10.67210600	5.98928800	-1.40898000
C	-3.80244100	3.95865200	-1.40672200	C	-3.79559000	3.97046000	-1.40543800

C	-3.80244100	3.95865200	1.40672200	C	-3.79559000	3.97046000	1.40543800
H	-10.68193100	5.94020800	2.49618600	H	-10.66913500	5.97376900	2.49637600
H	-10.68193100	5.94020800	-2.49618600	H	-10.66913500	5.97376900	-2.49637600
C	-2.91231900	3.13626100	0.72566000	C	-2.90342300	3.14060100	0.72350900
C	-2.91231900	3.13626100	-0.72566000	C	-2.90342300	3.14060100	-0.72350900
C	-2.10459400	2.16718100	-1.40624700	C	-2.10106100	2.17245500	-1.40451500
C	-2.10459400	2.16718100	1.40624700	C	-2.10106100	2.17245500	1.40451500
H	-5.88640200	5.26199300	-2.49536500	H	-5.87349800	5.28621400	-2.49515100
H	-5.88640200	5.26199300	2.49536500	H	-5.87349800	5.28621400	2.49515100
H	-3.81509100	3.94253900	2.49420700	H	-3.80780800	3.95427500	2.49293800
H	-3.81509100	3.94253900	-2.49420700	H	-3.80780800	3.95427500	-2.49293800
H	-2.13453800	2.14535700	-2.49342000	H	-2.12969800	2.15163500	-2.49176000
H	-2.13453800	2.14535700	2.49342000	H	-2.12969800	2.15163500	2.49176000
C	-8.23107500	-5.99340300	-1.40865600	C	-8.21712000	-6.02236500	-1.40890400
C	-9.44561300	-6.07593800	-0.73040900	C	-9.43944400	-6.09995400	-0.73037800
C	-9.44561300	-6.07593800	0.73040900	C	-9.43944400	-6.09995400	0.73037800
C	-8.23107500	-5.99340300	1.40865600	C	-8.21712000	-6.02236500	1.40890400
H	-8.23353200	-5.97246600	-2.49601600	H	-8.21892400	-6.00654800	-2.49630500
H	-8.23353200	-5.97246600	2.49601600	H	-8.21892400	-6.00654800	2.49630500
C	-7.02172200	5.72172000	-0.72912900	C	-7.00963300	5.74276700	-0.72929100
C	-8.23107500	5.99340300	-1.40865600	C	-8.21712000	6.02236500	-1.40890400
C	-9.44561300	6.07593800	-0.73040900	C	-9.43944400	6.09995400	-0.73037800
C	-9.44561300	6.07593800	0.73040900	C	-9.43944400	6.09995400	0.73037800
C	-8.23107500	5.99340300	1.40865600	C	-8.21712000	6.02236500	1.40890400
C	-7.02172200	5.72172000	0.72912900	C	-7.00963300	5.74276700	0.72929100
H	-8.23353200	5.97246600	-2.49601600	H	-8.21892400	6.00654800	-2.49630500
H	-8.23353200	5.97246600	2.49601600	H	-8.21892400	6.00654800	2.49630500
C	-4.80053800	-4.69176000	-0.72759000	C	-4.78707700	-4.70410900	-0.72660900
C	-5.87825800	-5.28090000	-1.40797500	C	-5.86693200	-5.30259600	-1.40773200
C	-7.02172200	-5.72172000	-0.72912900	C	-7.00963300	-5.74276700	-0.72929100
C	-7.02172200	-5.72172000	0.72912900	C	-7.00963300	-5.74276700	0.72929100
C	-5.87825800	-5.28090000	1.40797500	C	-5.86693200	-5.30259600	1.40773200
C	-4.80053800	-4.69176000	0.72759000	C	-4.78707700	-4.70410900	0.72660900
H	-5.88640200	-5.26199300	-2.49536500	H	-5.87349800	-5.28621400	-2.49515100
H	-5.88640200	-5.26199300	2.49536500	H	-5.87349800	-5.28621400	2.49515100
C	0.79571300	0.00000000	1.39862800	C	0.79620800	0.00000000	1.39788500
C	1.41817600	1.20701300	0.72182200	C	1.41606400	1.20751600	0.71957300
C	2.10459400	2.16718100	1.40624700	C	2.10106100	2.17245500	1.40451500
C	1.41817600	1.20701300	-0.72182200	C	1.41606400	1.20751600	-0.71957300
C	2.10459400	2.16718100	-1.40624700	C	2.10106100	2.17245500	-1.40451500
C	2.91231900	3.13626100	-0.72566000	C	2.90342300	3.14060100	-0.72350900
C	2.91231900	3.13626100	0.72566000	C	2.90342300	3.14060100	0.72350900
C	1.41817600	-1.20701300	0.72182200	C	1.41606400	-1.20751600	0.71957300
C	0.79571300	0.00000000	-1.39862800	C	0.79620800	0.00000000	-1.39788500
H	2.13453800	2.14535700	-2.49342000	H	2.12969800	2.15163500	-2.49176000
C	3.80244100	3.95865200	-1.40672200	C	3.79559000	3.97046000	-1.40543800
C	3.80244100	3.95865200	1.40672200	C	3.79559000	3.97046000	1.40543800
H	3.81509100	3.94253900	-2.49420700	H	3.80780800	3.95427500	-2.49293800
H	3.81509100	3.94253900	2.49420700	H	3.80780800	3.95427500	2.49293800
C	1.41817600	-1.20701300	-0.72182200	C	1.41606400	-1.20751600	-0.71957300
H	1.10229500	0.00000000	2.44777900	H	1.10381200	0.00000000	2.44676600
H	2.13453800	2.14535700	2.49342000	H	2.12969800	2.15163500	2.49176000
H	1.10229500	0.00000000	-2.44777900	H	1.10381200	0.00000000	-2.44676600
C	4.80053800	4.69176000	0.72759000	C	4.78707700	4.70410900	0.72660900
C	4.80053800	4.69176000	-0.72759000	C	4.78707700	4.70410900	-0.72660900
C	8.23107500	-5.99340300	1.40865600	C	8.21712000	-6.02236500	1.40890400
C	7.02172200	-5.72172000	0.72912900	C	7.00963300	-5.74276700	0.72929100
C	5.87825800	-5.28090000	1.40797500	C	5.86693200	-5.30259600	1.40773200
C	7.02172200	-5.72172000	-0.72912900	C	7.00963300	-5.74276700	-0.72929100
C	5.87825800	-5.28090000	-1.40797500	C	5.86693200	-5.30259600	-1.40773200

C	4.80053800	-4.69176000	-0.72759000	C	4.78707700	-4.70410900	-0.72660900
C	4.80053800	-4.69176000	0.72759000	C	4.78707700	-4.70410900	0.72660900
C	8.23107500	-5.99340300	-1.40865600	C	8.21712000	-6.02236500	-1.40890400
C	3.80244100	-3.95865200	-1.40672200	C	3.79559000	-3.97046000	-1.40543800
C	3.80244100	-3.95865200	1.40672200	C	3.79559000	-3.97046000	1.40543800
H	8.23353200	-5.97246600	2.49601600	H	8.21892400	-6.00654800	2.49630500
H	8.23353200	-5.97246600	-2.49601600	H	8.21892400	-6.00654800	-2.49630500
C	2.91231900	-3.13626100	0.72566000	C	2.90342300	-3.14060100	0.72350900
C	2.91231900	-3.13626100	-0.72566000	C	2.90342300	-3.14060100	-0.72350900
C	2.10459400	-2.16718100	-1.40624700	C	2.10106100	-2.17245500	-1.40451500
C	2.10459400	-2.16718100	1.40624700	C	2.10106100	-2.17245500	1.40451500
H	5.88640200	-5.26199300	-2.49536500	H	5.87349800	-5.28621400	-2.49515100
H	5.88640200	-5.26199300	2.49536500	H	5.87349800	-5.28621400	2.49515100
H	3.81509100	-3.94253900	2.49420700	H	3.80780800	-3.95427500	2.49293800
H	3.81509100	-3.94253900	-2.49420700	H	3.80780800	-3.95427500	-2.49293800
H	2.13453800	-2.14535700	-2.49342000	H	2.12969800	-2.15163500	-2.49176000
H	2.13453800	-2.14535700	2.49342000	H	2.12969800	-2.15163500	2.49176000
C	15.80714400	1.22406300	1.40658400	C	15.77837600	1.22609700	1.40838400
C	15.91844000	0.00000000	0.73341900	C	15.90179100	0.00000000	0.72914600
C	15.80714400	-1.22406300	1.40658400	C	15.77837600	-1.22609700	1.40838400
C	15.91844000	0.00000000	-0.73341900	C	15.90179100	0.00000000	-0.72914600
C	15.80714400	-1.22406300	-1.40658400	C	15.77837600	-1.22609700	-1.40838400
C	15.39965700	-2.39420000	-0.73315700	C	15.38720800	-2.39426100	-0.72929900
C	15.39965700	-2.39420000	0.73315700	C	15.38720800	-2.39426100	0.72929900
C	15.39965700	2.39420000	0.73315700	C	15.38720800	2.39426100	0.72929900
C	15.80714400	1.22406300	-1.40658400	C	15.77837600	1.22609700	-1.40838400
H	15.79931300	-1.22251700	-2.49422700	H	15.75822900	-1.22173800	-2.49569700
C	14.79731000	-3.45445300	-1.40741300	C	14.77538900	-3.46406500	-1.40847800
C	14.79731000	-3.45445300	1.40741300	C	14.77538900	-3.46406500	1.40847800
H	14.78733300	-3.44765000	-2.49499200	H	14.75978900	-3.45264900	-2.49582000
H	14.78733300	-3.44765000	2.49499200	H	14.75978900	-3.45264900	2.49582000
C	15.39965700	2.39420000	-0.73315700	C	15.38720800	2.39426100	-0.72929900
H	15.79931300	1.22251700	2.49422700	H	15.75822900	1.22173800	2.49569700
H	15.79931300	-1.22251700	2.49422700	H	15.75822900	-1.22173800	2.49569700
H	15.79931300	1.22251700	-2.49422700	H	15.75822900	1.22173800	-2.49569700
C	5.87825800	5.28090000	1.40797500	C	5.86693200	5.30259600	1.40773200
C	10.68617000	5.96008900	1.40879900	C	10.67210600	5.98928800	1.40898000
C	10.68617000	5.96008900	-1.40879900	C	10.67210600	5.98928800	-1.40898000
C	11.85520100	5.63682200	-0.73151700	C	11.85380600	5.65412900	-0.73024800
C	11.85520100	5.63682200	0.73151700	C	11.85380600	5.65412900	0.73024800
C	5.87825800	5.28090000	-1.40797500	C	5.86693200	5.30259600	-1.40773200
C	12.98330400	5.10164500	-1.40834000	C	12.96776200	5.12238800	-1.40869800
C	12.98330400	5.10164500	1.40834000	C	12.96776200	5.12238800	1.40869800
H	5.88640200	5.26199300	2.49536500	H	5.87349800	5.28621400	2.49515100
H	5.88640200	5.26199300	-2.49536500	H	5.87349800	5.28621400	-2.49515100
C	13.95259800	4.37084900	0.73247600	C	13.94806100	4.37742600	0.72973200
C	13.95259800	4.37084900	-0.73247600	C	13.94806100	4.37742600	-0.72973200
C	14.79731000	3.45445300	-1.40741300	C	14.77538900	3.46406500	-1.40847800
C	14.79731000	3.45445300	1.40741300	C	14.77538900	3.46406500	1.40847800
H	10.68193100	5.94020800	-2.49618600	H	10.66913500	5.97376900	-2.49637600
H	10.68193100	5.94020800	2.49618600	H	10.66913500	5.97376900	2.49637600
H	12.97401900	5.08736900	2.49581700	H	12.95883500	5.10764900	2.49607600
H	12.97401900	5.08736900	-2.49581700	H	12.95883500	5.10764900	-2.49607600
H	14.78733300	3.44765000	-2.49499200	H	14.75978900	3.45264900	-2.49582000
H	14.78733300	3.44765000	2.49499200	H	14.75978900	3.45264900	2.49582000
C	10.68617000	-5.96008900	-1.40879900	C	10.67210600	-5.98928800	-1.40898000
C	9.44561300	-6.07593800	-0.73040900	C	9.43944400	-6.09995400	-0.73037800
C	9.44561300	-6.07593800	0.73040900	C	9.43944400	-6.09995400	0.73037800
C	10.68617000	-5.96008900	1.40879900	C	10.67210600	-5.98928800	1.40898000
H	10.68193100	-5.94020800	-2.49618600	H	10.66913500	-5.97376900	-2.49637600

H	10.68193100	-5.94020800	2.49618600	H	10.66913500	-5.97376900	2.49637600
C	9.44561300	6.07593800	-0.73040900	C	9.43944400	6.09995400	-0.73037800
C	8.23107500	5.99340300	-1.40865600	C	8.21712000	6.02236500	-1.40890400
C	7.02172200	5.72172000	-0.72912900	C	7.00963300	5.74276700	-0.72929100
C	7.02172200	5.72172000	0.72912900	C	7.00963300	5.74276700	0.72929100
C	8.23107500	5.99340300	1.40865600	C	8.21712000	6.02236500	1.40890400
C	9.44561300	6.07593800	0.73040900	C	9.43944400	6.09995400	0.73037800
H	8.23353200	5.97246600	-2.49601600	H	8.21892400	6.00654800	-2.49630500
H	8.23353200	5.97246600	2.49601600	H	8.21892400	6.00654800	2.49630500
C	13.95259800	-4.37084900	-0.73247600	C	13.94806100	-4.37742600	-0.72973200
C	12.98330400	-5.10164500	-1.40834000	C	12.96776200	-5.12238800	-1.40869800
C	11.85520100	-5.63682200	-0.73151700	C	11.85380600	-5.65412900	-0.73024800
C	11.85520100	-5.63682200	0.73151700	C	11.85380600	-5.65412900	0.73024800
C	12.98330400	-5.10164500	1.40834000	C	12.96776200	-5.12238800	1.40869800
C	13.95259800	-4.37084900	0.73247600	C	13.94806100	-4.37742600	0.72973200
H	12.97401900	-5.08736900	-2.49581700	H	12.95883500	-5.10764900	-2.49607600
H	12.97401900	-5.08736900	2.49581700	H	12.95883500	-5.10764900	2.49607600
**Dcyc18_R**				**Dcyc18_U**			
C	-12.92026900	5.79841200	1.40877100	C	-12.90581500	5.82477800	1.40863700
C	-11.73979200	6.19949600	0.73152700	C	-11.73747200	6.22233100	0.73034700
C	-10.54187800	6.40178600	1.40913900	C	-10.52780700	6.43437500	1.40891400
C	-11.73979200	6.19949600	-0.73152700	C	-11.73747200	6.22233100	-0.73034700
C	-10.54187800	6.40178600	-1.40913900	C	-10.52780700	6.43437500	-1.40891400
C	-9.29858700	6.40957000	-0.73064500	C	-9.29032300	6.43681800	-0.73046700
C	-9.29858700	6.40957000	0.73064500	C	-9.29032300	6.43681800	0.73046700
C	-13.97957400	5.20550400	0.73226100	C	-13.97677800	5.21755700	0.72985100
C	-12.92026900	5.79841200	-1.40877100	C	-12.90581500	5.82477800	-1.40863700
H	-10.53962400	6.38243200	-2.49653200	H	-10.52645200	6.42003500	-2.49635300
C	-8.09140400	6.23737600	-1.40894300	C	-8.07743000	6.26809200	-1.40888200
C	-8.09140400	6.23737600	1.40894300	C	-8.07743000	6.26809200	1.40888200
H	-8.09502400	6.21801000	-2.49631400	H	-8.08003700	6.25360800	-2.49630500
H	-8.09502400	6.21801000	2.49631400	H	-8.08003700	6.25360800	2.49630500
C	-13.97957400	5.20550400	-0.73226100	C	-13.97677800	5.21755700	-0.72985100
H	-12.91266400	5.78240300	2.49621800	H	-12.89942600	5.81034800	2.49604300
H	-10.53962400	6.38243200	2.49653200	H	-10.52645200	6.42003500	2.49635300
H	-12.91266400	5.78240300	-2.49621800	H	-12.89942600	5.81034800	-2.49604300
C	-6.90411700	5.89209100	0.72965000	C	-6.88964200	5.91448400	0.72932800
C	-6.90411700	5.89209100	-0.72965000	C	-6.88964200	5.91448400	-0.72932800
C	-16.32921900	-2.39845300	1.40708200	C	-16.30398900	-2.40408500	1.40836500
C	-16.67851000	-1.22514400	0.73323900	C	-16.66186900	-1.22476200	0.72919200
C	-16.82036700	0.00000000	1.40667500	C	-16.79156000	0.00000000	1.40835400
C	-16.67851000	-1.22514400	-0.73323900	C	-16.66186900	-1.22476200	-0.72919200
C	-16.82036700	0.00000000	-1.40667500	C	-16.79156000	0.00000000	-1.40835400
C	-16.67851000	1.22514400	-0.73323900	C	-16.66186900	1.22476200	-0.72919200
C	-16.67851000	1.22514400	0.73323900	C	-16.66186900	1.22476200	0.72919200
C	-16.32921900	-2.39845300	-1.40708200	C	-16.30398900	-2.40408500	-1.40836500
C	-16.32921900	2.39845300	-1.40708200	C	-16.30398900	2.40408500	-1.40836500
C	-16.32921900	2.39845300	1.40708200	C	-16.30398900	2.40408500	1.40836500
H	-16.31989200	-2.39472100	2.49469400	H	-16.28622500	-2.39652700	2.49570800
H	-16.31989200	-2.39472100	-2.49469400	H	-16.28622500	-2.39652700	-2.49570800
C	-15.72016100	3.48058100	0.73287200	C	-15.71032800	3.48334300	0.72941800
C	-15.72016100	3.48058100	-0.73287200	C	-15.71032800	3.48334300	-0.72941800
C	-14.94902200	4.42229100	-1.40816600	C	-14.93050300	4.43771100	-1.40843300
C	-14.94902200	4.42229100	1.40816600	C	-14.93050300	4.43771100	1.40843300
H	-16.81201800	0.00000000	-2.49458700	H	-16.77172300	0.00000000	-2.49566300
H	-16.81201800	0.00000000	2.49458700	H	-16.77172300	0.00000000	2.49566300
H	-16.31989200	2.39472100	2.49469400	H	-16.28622500	2.39652700	2.49570800
H	-16.31989200	2.39472100	-2.49469400	H	-16.28622500	2.39652700	-2.49570800

H	-14.93886500	4.41261500	-2.49570300	H	-14.91809000	4.42514700	-2.49580900
H	-14.93886500	4.41261500	2.49570300	H	-14.91809000	4.42514700	2.49580900
C	-3.75524900	-3.99796500	1.40722700	C	-3.74872900	-4.01093300	1.40544000
C	-4.73128800	-4.76267700	0.72824800	C	-4.71667900	-4.77524700	0.72663200
C	-5.78219100	-5.39464800	1.40838500	C	-5.77118800	-5.41698900	1.40769300
C	-4.73128800	-4.76267700	-0.72824800	C	-4.71667900	-4.77524700	-0.72663200
C	-5.78219100	-5.39464800	-1.40838500	C	-5.77118800	-5.41698900	-1.40769300
C	-6.90411700	-5.89209100	-0.72965000	C	-6.88964200	-5.91448400	-0.72932800
C	-6.90411700	-5.89209100	0.72965000	C	-6.88964200	-5.91448400	0.72932800
C	-2.88607700	-3.15513900	0.72637500	C	-2.87658600	-3.16038300	0.72355100
C	-3.75524900	-3.99796500	-1.40722700	C	-3.74872900	-4.01093300	-1.40544000
H	-5.79033100	-5.37769200	-2.49565900	H	-5.77792000	-5.40200800	-2.49516200
C	-8.09140400	-6.23737600	-1.40894300	C	-8.07743000	-6.26809200	-1.40888200
C	-8.09140400	-6.23737600	1.40894300	C	-8.07743000	-6.26809200	1.40888200
H	-8.09502400	-6.21801000	-2.49631400	H	-8.08003700	-6.25360800	-2.49630500
H	-8.09502400	-6.21801000	2.49631400	H	-8.08003700	-6.25360800	2.49630500
C	-2.88607700	-3.15513900	-0.72637500	C	-2.87658600	-3.16038300	-0.72355100
H	-3.76751700	-3.98309900	2.49471500	H	-3.76045000	-3.99611800	2.49296200
H	-5.79033100	-5.37769200	2.49565900	H	-5.77792000	-5.40200800	2.49516200
H	-3.76751700	-3.98309900	-2.49471500	H	-3.76045000	-3.99611800	-2.49296200
C	-5.78219100	5.39464800	1.40838500	C	-5.77118800	5.41698900	1.40769300
C	-2.09110400	2.17488400	1.40664800	C	-2.08889400	2.17987400	1.40452700
C	-2.09110400	2.17488400	-1.40664800	C	-2.08889400	2.17987400	-1.40452700
C	-1.41553300	1.20758500	-0.72201500	C	-1.41382700	1.20822200	-0.71964000
C	-1.41553300	1.20758500	0.72201500	C	-1.41382700	1.20822200	0.71964000
C	-5.78219100	5.39464800	-1.40838500	C	-5.77118800	5.41698900	-1.40769300
C	-0.79620900	0.00000000	-1.39915200	C	-0.79667900	0.00000000	-1.39807300
C	-0.79620900	0.00000000	1.39915200	C	-0.79667900	0.00000000	1.39807300
H	-5.79033100	5.37769200	2.49565900	H	-5.77792000	5.40200800	2.49516200
H	-5.79033100	5.37769200	-2.49565900	H	-5.77792000	5.40200800	-2.49516200
C	-1.41553300	-1.20758500	0.72201500	C	-1.41382700	-1.20822200	0.71964000
C	-1.41553300	-1.20758500	-0.72201500	C	-1.41382700	-1.20822200	-0.71964000
C	-2.09110400	-2.17488400	-1.40664800	C	-2.08889400	-2.17987400	-1.40452700
C	-2.09110400	-2.17488400	1.40664800	C	-2.08889400	-2.17987400	1.40452700
H	-2.11837300	2.15543400	-2.49373700	H	-2.11623100	2.16044100	-2.49187700
H	-2.11837300	2.15543400	2.49373700	H	-2.11623100	2.16044100	2.49187700
H	-1.10321200	0.00000000	2.44837000	H	-1.10407300	0.00000000	2.44700800
H	-1.10321200	0.00000000	-2.44837000	H	-1.10407300	0.00000000	-2.44700800
H	-2.11837300	-2.15543400	-2.49373700	H	-2.11623100	-2.16044100	-2.49187700
H	-2.11837300	-2.15543400	2.49373700	H	-2.11623100	-2.16044100	2.49187700
C	-14.94902200	-4.42229100	-1.40816600	C	-14.93050300	-4.43771100	-1.40843300
C	-15.72016100	-3.48058100	-0.73287200	C	-15.71032800	-3.48334300	-0.72941800
C	-15.72016100	-3.48058100	0.73287200	C	-15.71032800	-3.48334300	0.72941800
C	-14.94902200	-4.42229100	1.40816600	C	-14.93050300	-4.43771100	1.40843300
H	-14.93886500	-4.41261500	-2.49570300	H	-14.91809000	-4.42514700	-2.49580900
H	-14.93886500	-4.41261500	2.49570300	H	-14.91809000	-4.42514700	2.49580900
C	-2.88607700	3.15513900	-0.72637500	C	-2.87658600	3.16038300	-0.72355100
C	-3.75524900	3.99796500	-1.40722700	C	-3.74872900	4.01093300	-1.40544000
C	-4.73128800	4.76267700	-0.72824800	C	-4.71667900	4.77524700	-0.72663200
C	-4.73128800	4.76267700	0.72824800	C	-4.71667900	4.77524700	0.72663200
C	-3.75524900	3.99796500	1.40722700	C	-3.74872900	4.01093300	1.40544000
C	-2.88607700	3.15513900	0.72637500	C	-2.87658600	3.16038300	0.72355100
H	-3.76751700	3.98309900	-2.49471500	H	-3.76045000	3.99611800	-2.49296200
H	-3.76751700	3.98309900	2.49471500	H	-3.76045000	3.99611800	2.49296200
C	-9.29858700	-6.40957000	-0.73064500	C	-9.29032300	-6.43681800	-0.73046700
C	-10.54187800	-6.40178600	-1.40913900	C	-10.52780700	-6.43437500	-1.40891400
C	-10.54187800	-6.40178600	1.40913900	C	-10.52780700	-6.43437500	1.40891400
C	-9.29858700	-6.40957000	0.73064500	C	-9.29032300	-6.43681800	0.73046700
H	-10.53962400	-6.38243200	-2.49653200	H	-10.52645200	-6.42003500	-2.49635300
H	-10.53962400	-6.38243200	2.49653200	H	-10.52645200	-6.42003500	2.49635300

C	-11.73979200	-6.19949600	-0.73152700	C	-11.73747200	-6.22233100	-0.73034700
C	-12.92026900	-5.79841200	-1.40877100	C	-12.90581500	-5.82477800	-1.40863700
C	-13.97957400	-5.20550400	-0.73226100	C	-13.97677800	-5.21755700	-0.72985100
C	-13.97957400	-5.20550400	0.73226100	C	-13.97677800	-5.21755700	0.72985100
C	-12.92026900	-5.79841200	1.40877100	C	-12.90581500	-5.82477800	1.40863700
C	-11.73979200	-6.19949600	0.73152700	C	-11.73747200	-6.22233100	0.73034700
H	-12.91266400	-5.78240300	-2.49621800	H	-12.89942600	-5.81034800	-2.49604300
H	-12.91266400	-5.78240300	2.49621800	H	-12.89942600	-5.81034800	2.49604300
C	3.75524900	3.99796500	1.40722700	C	3.74872900	4.01093300	1.40544000
C	4.73128800	4.76267700	0.72824800	C	4.71667900	4.77524700	0.72663200
C	5.78219100	5.39464800	1.40838500	C	5.77118800	5.41698900	1.40769300
C	4.73128800	4.76267700	-0.72824800	C	4.71667900	4.77524700	-0.72663200
C	5.78219100	5.39464800	-1.40838500	C	5.77118800	5.41698900	-1.40769300
C	6.90411700	5.89209100	-0.72965000	C	6.88964200	5.91448400	-0.72932800
C	6.90411700	5.89209100	0.72965000	C	6.88964200	5.91448400	0.72932800
C	2.88607700	3.15513900	0.72637500	C	2.87658600	3.16038300	0.72355100
C	3.75524900	3.99796500	-1.40722700	C	3.74872900	4.01093300	-1.40544000
H	5.79033100	5.37769200	-2.49565900	H	5.77792000	5.40200800	-2.49516200
C	8.09140400	6.23737600	-1.40894300	C	8.07743000	6.26809200	-1.40888200
C	8.09140400	6.23737600	1.40894300	C	8.07743000	6.26809200	1.40888200
H	8.09502400	6.21801000	-2.49631400	H	8.08003700	6.25360800	-2.49630500
H	8.09502400	6.21801000	2.49631400	H	8.08003700	6.25360800	2.49630500
C	2.88607700	3.15513900	-0.72637500	C	2.87658600	3.16038300	-0.72355100
H	3.76751700	3.98309900	2.49471500	H	3.76045000	3.99611800	2.49296200
H	5.79033100	5.37769200	2.49565900	H	5.77792000	5.40200800	2.49516200
H	3.76751700	3.98309900	-2.49471500	H	3.76045000	3.99611800	-2.49296200
C	9.29858700	6.40957000	0.73064500	C	9.29032300	6.43681800	0.73046700
C	9.29858700	6.40957000	-0.73064500	C	9.29032300	6.43681800	-0.73046700
C	3.75524900	-3.99796500	1.40722700	C	3.74872900	-4.01093300	1.40544000
C	2.88607700	-3.15513900	0.72637500	C	2.87658600	-3.16038300	0.72355100
C	2.09110400	-2.17488400	1.40664800	C	2.08889400	-2.17987400	1.40452700
C	2.88607700	-3.15513900	-0.72637500	C	2.87658600	-3.16038300	-0.72355100
C	2.09110400	-2.17488400	-1.40664800	C	2.08889400	-2.17987400	-1.40452700
C	1.41553300	-1.20758500	-0.72201500	C	1.41382700	-1.20822200	-0.71964000
C	1.41553300	-1.20758500	0.72201500	C	1.41382700	-1.20822200	0.71964000
C	3.75524900	-3.99796500	-1.40722700	C	3.74872900	-4.01093300	-1.40544000
C	0.79620900	0.00000000	-1.39915200	C	0.79667900	0.00000000	-1.39807300
C	0.79620900	0.00000000	1.39915200	C	0.79667900	0.00000000	1.39807300
H	3.76751700	-3.98309900	2.49471500	H	3.76045000	-3.99611800	2.49296200
H	3.76751700	-3.98309900	-2.49471500	H	3.76045000	-3.99611800	-2.49296200
C	1.41553300	1.20758500	0.72201500	C	1.41382700	1.20822200	0.71964000
C	1.41553300	1.20758500	-0.72201500	C	1.41382700	1.20822200	-0.71964000
C	2.09110400	2.17488400	-1.40664800	C	2.08889400	2.17987400	-1.40452700
C	2.09110400	2.17488400	1.40664800	C	2.08889400	2.17987400	1.40452700
H	2.11837300	-2.15543400	-2.49373700	H	2.11623100	-2.16044100	-2.49187700
H	2.11837300	-2.15543400	2.49373700	H	2.11623100	-2.16044100	2.49187700
H	1.10321200	0.00000000	2.44837000	H	1.10407300	0.00000000	2.44700800
H	1.10321200	0.00000000	-2.44837000	H	1.10407300	0.00000000	-2.44700800
H	2.11837300	2.15543400	-2.49373700	H	2.11623100	2.16044100	-2.49187700
H	2.11837300	2.15543400	2.49373700	H	2.11623100	2.16044100	2.49187700
C	16.32921900	-2.39845300	1.40708200	C	16.30398900	-2.40408500	1.40836500
C	15.72016100	-3.48058100	0.73287200	C	15.71032800	-3.48334300	0.72941800
C	14.94902200	-4.42229100	1.40816600	C	14.93050300	-4.43771100	1.40843300
C	15.72016100	-3.48058100	-0.73287200	C	15.71032800	-3.48334300	-0.72941800
C	14.94902200	-4.42229100	-1.40816600	C	14.93050300	-4.43771100	-1.40843300
C	13.97957400	-5.20550400	-0.73226100	C	13.97677800	-5.21755700	-0.72985100
C	13.97957400	-5.20550400	0.73226100	C	13.97677800	-5.21755700	0.72985100
C	16.67851000	-1.22514400	0.73323900	C	16.66186900	-1.22476200	0.72919200
C	16.32921900	-2.39845300	-1.40708200	C	16.30398900	-2.40408500	-1.40836500
H	14.93886500	-4.41261500	-2.49570300	H	14.91809000	-4.42514700	-2.49580900

C	12.92026900	-5.79841200	-1.40877100	C	12.90581500	-5.82477800	-1.40863700
C	12.92026900	-5.79841200	1.40877100	C	12.90581500	-5.82477800	1.40863700
H	12.91266400	-5.78240300	-2.49621800	H	12.89942600	-5.81034800	-2.49604300
H	12.91266400	-5.78240300	2.49621800	H	12.89942600	-5.81034800	2.49604300
C	16.67851000	-1.22514400	-0.73323900	C	16.66186900	-1.22476200	-0.72919200
H	16.31989200	-2.39472100	2.49469400	H	16.28622500	-2.39652700	2.49570800
H	14.93886500	-4.41261500	2.49570300	H	14.91809000	-4.42514700	2.49580900
H	16.31989200	-2.39472100	-2.49469400	H	16.28622500	-2.39652700	-2.49570800
C	10.54187800	6.40178600	1.40913900	C	10.52780700	6.43437500	1.40891400
C	14.94902200	4.42229100	1.40816600	C	14.93050300	4.43771100	1.40843300
C	14.94902200	4.42229100	-1.40816600	C	14.93050300	4.43771100	-1.40843300
C	15.72016100	3.48058100	-0.73287200	C	15.71032800	3.48334300	-0.72941800
C	15.72016100	3.48058100	0.73287200	C	15.71032800	3.48334300	0.72941800
C	10.54187800	6.40178600	-1.40913900	C	10.52780700	6.43437500	-1.40891400
C	16.32921900	2.39845300	-1.40708200	C	16.30398900	2.40408500	-1.40836500
C	16.32921900	2.39845300	1.40708200	C	16.30398900	2.40408500	1.40836500
H	10.53962400	6.38243200	2.49653200	H	10.52645200	6.42003500	2.49635300
H	10.53962400	6.38243200	-2.49653200	H	10.52645200	6.42003500	-2.49635300
C	16.67851000	1.22514400	0.73323900	C	16.66186900	1.22476200	0.72919200
C	16.67851000	1.22514400	-0.73323900	C	16.66186900	1.22476200	-0.72919200
C	16.82036700	0.00000000	-1.40667500	C	16.79156000	0.00000000	-1.40835400
C	16.82036700	0.00000000	1.40667500	C	16.79156000	0.00000000	1.40835400
H	14.93886500	4.41261500	-2.49570300	H	14.91809000	4.42514700	-2.49580900
H	14.93886500	4.41261500	2.49570300	H	14.91809000	4.42514700	2.49580900
H	16.31989200	2.39472100	2.49469400	H	16.28622500	2.39652700	2.49570800
H	16.31989200	2.39472100	-2.49469400	H	16.28622500	2.39652700	-2.49570800
H	16.81201800	0.00000000	-2.49458700	H	16.77172300	0.00000000	-2.49566300
H	16.81201800	0.00000000	2.49458700	H	16.77172300	0.00000000	2.49566300
C	5.78219100	-5.39464800	-1.40838500	C	5.77118800	-5.41698900	-1.40769300
C	4.73128800	-4.76267700	-0.72824800	C	4.71667900	-4.77524700	-0.72663200
C	4.73128800	-4.76267700	0.72824800	C	4.71667900	-4.77524700	0.72663200
C	5.78219100	-5.39464800	1.40838500	C	5.77118800	-5.41698900	1.40769300
H	5.79033100	-5.37769200	-2.49565900	H	5.77792000	-5.40200800	-2.49516200
H	5.79033100	-5.37769200	2.49565900	H	5.77792000	-5.40200800	2.49516200
C	13.97957400	5.20550400	-0.73226100	C	13.97677800	5.21755700	-0.72985100
C	12.92026900	5.79841200	-1.40877100	C	12.90581500	5.82477800	-1.40863700
C	11.73979200	6.19949600	-0.73152700	C	11.73747200	6.22233100	-0.73034700
C	11.73979200	6.19949600	0.73152700	C	11.73747200	6.22233100	0.73034700
C	12.92026900	5.79841200	1.40877100	C	12.90581500	5.82477800	1.40863700
C	13.97957400	5.20550400	0.73226100	C	13.97677800	5.21755700	0.72985100
H	12.91266400	5.78240300	-2.49621800	H	12.89942600	5.81034800	-2.49604300
H	12.91266400	5.78240300	2.49621800	H	12.89942600	5.81034800	2.49604300
C	11.73979200	-6.19949600	-0.73152700	C	11.73747200	-6.22233100	-0.73034700
C	10.54187800	-6.40178600	-1.40913900	C	10.52780700	-6.43437500	-1.40891400
C	10.54187800	-6.40178600	1.40913900	C	10.52780700	-6.43437500	1.40891400
C	11.73979200	-6.19949600	0.73152700	C	11.73747200	-6.22233100	0.73034700
H	10.53962400	-6.38243200	-2.49653200	H	10.52645200	-6.42003500	-2.49635300
H	10.53962400	-6.38243200	2.49653200	H	10.52645200	-6.42003500	2.49635300
C	9.29858700	-6.40957000	-0.73064500	C	9.29032300	-6.43681800	-0.73046700
C	8.09140400	-6.23737600	-1.40894300	C	8.07743000	-6.26809200	-1.40888200
C	6.90411700	-5.89209100	-0.72965000	C	6.88964200	-5.91448400	-0.72932800
C	6.90411700	-5.89209100	0.72965000	C	6.88964200	-5.91448400	0.72932800
C	8.09140400	-6.23737600	1.40894300	C	8.07743000	-6.26809200	1.40888200
C	9.29858700	-6.40957000	0.73064500	C	9.29032300	-6.43681800	0.73046700
H	8.09502400	-6.21801000	-2.49631400	H	8.08003700	-6.25360800	-2.49630500
H	8.09502400	-6.21801000	2.49631400	H	8.08003700	-6.25360800	2.49630500
**Dcyc19_R**				**Dcyc19_U**			
C	-17.57802200	1.22489000	1.40698200	C	17.561183000	1.226828000	-1.408251000
C	-17.21335600	2.40729200	0.73332900	C	17.209771000	2.406922000	-0.729242000

C	-16.66699800	3.49866900	1.40766100	C	16.655363000	3.507890000	-1.408261000
C	-17.21335600	2.40729200	-0.73332900	C	17.209772000	2.406923000	0.729236000
C	-16.66699800	3.49866900	-1.40766100	C	16.655365000	3.507891000	1.408256000
C	-15.89539700	4.47429700	-0.73286400	C	15.899477000	4.479516000	0.729495000
C	-15.89539700	4.47429700	0.73286400	C	15.899476000	4.479516000	-0.729499000
C	-17.67923100	0.00000000	0.73351200	C	17.671939000	-0.000246000	-0.729153000
C	-17.57802200	1.22489000	-1.40698200	C	17.561186000	1.226829000	1.408246000
H	-16.65708400	3.49266600	-2.49523300	H	16.640120000	3.498174000	2.495640000
C	-14.99639900	5.29210100	-1.40856400	C	14.990111000	5.311504000	1.408321000
C	-14.99639900	5.29210100	1.40856400	C	14.990110000	5.311503000	-1.408325000
H	-14.98684600	5.28005800	-2.49607200	H	14.980222000	5.298301000	2.495736000
H	-14.98684600	5.28005800	2.49607200	H	14.980219000	5.298300000	-2.495740000
C	-17.67923100	0.00000000	-0.73351200	C	17.671940000	-0.000246000	0.729148000
H	-17.56938400	1.22333200	2.49462600	H	17.542446000	1.223211000	-2.495610000
H	-16.65708400	3.49266600	2.49523300	H	16.640118000	3.498173000	-2.495645000
H	-17.56938400	1.22333200	-2.49462600	H	17.542450000	1.223213000	2.495605000
C	-13.93481100	5.94547500	0.73222700	C	13.943513000	5.961027000	-0.729909000
C	-13.93481100	5.94547500	-0.73222700	C	13.943513000	5.961027000	0.729906000
C	-12.81421700	-6.41084200	1.40923800	C	12.809112000	-6.439969000	-1.408513000
C	-13.93481100	-5.94547500	0.73222700	C	13.943321000	-5.961357000	-0.729904000
C	-14.99639900	-5.29210100	1.40856400	C	14.989958000	-5.311900000	-1.408320000
C	-13.93481100	-5.94547500	-0.73222700	C	13.943322000	-5.961356000	0.729911000
C	-14.99639900	-5.29210100	-1.40856400	C	14.989960000	-5.311898000	1.408326000
C	-15.89539700	-4.47429700	-0.73286400	C	15.899364000	-4.479953000	0.729499000
C	-15.89539700	-4.47429700	0.73286400	C	15.899363000	-4.479954000	-0.729495000
C	-12.81421700	-6.41084200	-1.40923800	C	12.809113000	-6.439967000	1.408523000
C	-16.66699800	-3.49866900	-1.40766100	C	16.655291000	-3.508357000	1.408259000
C	-16.66699800	-3.49866900	1.40766100	C	16.655289000	-3.508358000	-1.408257000
H	-12.80840300	-6.39391600	2.49666900	H	12.804724000	-6.426129000	-2.495959000
H	-12.80840300	-6.39391600	-2.49666900	H	12.804725000	-6.426124000	2.495968000
C	-17.21335600	-2.40729200	0.73332900	C	17.209725000	-2.407406000	-0.729239000
C	-17.21335600	-2.40729200	-0.73332900	C	17.209726000	-2.407405000	0.729239000
C	-17.57802200	-1.22489000	-1.40698200	C	17.561167000	-1.227317000	1.408247000
C	-17.57802200	-1.22489000	1.40698200	C	17.561164000	-1.227318000	-1.408250000
H	-14.98684600	-5.28005800	-2.49607200	H	14.980085000	-5.298713000	2.495742000
H	-14.98684600	-5.28005800	2.49607200	H	14.980083000	-5.298716000	-2.495736000
H	-16.65708400	-3.49266600	2.49523300	H	16.640059000	-3.498653000	-2.495642000
H	-16.65708400	-3.49266600	-2.49523300	H	16.640062000	-3.498649000	2.495643000
H	-17.56938400	-1.22333200	-2.49462600	H	17.542439000	-1.223702000	2.495607000
H	-17.56938400	-1.22333200	2.49462600	H	17.542435000	-1.223705000	-2.495609000
C	-2.07899400	2.18198500	1.40689300	C	2.082935000	2.186429000	-1.404570000
C	-1.41349000	1.20861700	0.72252200	C	1.416985000	1.208031000	-0.719420000
C	-0.79707000	0.00000000	1.39912100	C	0.800709000	0.000248000	-1.396418000
C	-1.41349000	1.20861700	-0.72252200	C	1.416984000	1.208032000	0.719416000
C	-0.79707000	0.00000000	-1.39912100	C	0.800708000	0.000249000	1.396416000
C	-1.41349000	-1.20861700	-0.72252200	C	1.416980000	-1.207537000	0.719416000
C	-1.41349000	-1.20861700	0.72252200	C	1.416981000	-1.207538000	-0.719416000
C	-2.85825300	3.17556900	0.72661900	C	2.856688000	3.176975000	-0.723563000
C	-2.07899400	2.18198500	-1.40689300	C	2.082933000	2.186431000	1.404565000
H	-1.10337000	0.00000000	-2.44836100	H	1.104377000	0.000248000	2.446680000
C	-2.07899400	-2.18198500	-1.40689300	C	2.082917000	-2.185943000	1.404570000
C	-2.07899400	-2.18198500	1.40689300	C	2.082918000	-2.185945000	-1.404569000
H	-2.10645700	-2.16380800	-2.49420300	H	2.109053000	-2.168394000	2.491989000
H	-2.10645700	-2.16380800	2.49420300	H	2.109054000	-2.168396000	-2.491988000
C	-2.85825300	3.17556900	-0.72661900	C	2.856687000	3.176976000	0.723558000
H	-2.10645700	2.16380800	2.49420300	H	2.109059000	2.168888000	-2.491991000
H	-1.10337000	0.00000000	2.44836100	H	1.104375000	0.000245000	-2.446683000
H	-2.10645700	2.16380800	-2.49420300	H	2.109055000	2.168892000	2.491986000
C	-12.81421700	6.41084200	1.40923800	C	12.809338000	6.439715000	-1.408520000
C	-7.95450000	6.45215200	1.40929800	C	7.949775000	6.480519000	-1.408790000

C	-7.95450000	6.45215200	-1.40929800	C	7.949775000	6.480519000	1.408786000
C	-6.78948600	6.04461300	-0.72980800	C	6.782776000	6.063274000	0.729357000
C	-6.78948600	6.04461300	0.72980800	C	6.782776000	6.063275000	-0.729361000
C	-12.81421700	6.41084200	-1.40923800	C	12.809338000	6.439715000	1.408517000
C	-5.68936400	5.49664100	-1.40865600	C	5.687530000	5.516325000	1.407648000
C	-5.68936400	5.49664100	1.40865600	C	5.687531000	5.516325000	-1.407653000
H	-12.80840300	6.39391600	2.49666900	H	12.804941000	6.425853000	-2.495964000
H	-12.80840300	6.39391600	-2.49666900	H	12.804941000	6.425854000	2.495962000
C	-4.66337300	4.82855700	0.72849200	C	4.656683000	4.837002000	-0.726653000
C	-4.66337300	4.82855700	-0.72849200	C	4.656682000	4.837003000	0.726648000
C	-3.70800300	4.03608800	-1.40746000	C	3.710736000	4.046006000	1.405370000
C	-3.70800300	4.03608800	1.40746000	C	3.710737000	4.046005000	-1.405376000
H	-7.95904700	6.43426300	-2.49668600	H	7.952993000	6.467260000	2.496237000
H	-7.95904700	6.43426300	2.49668600	H	7.952993000	6.467262000	-2.496241000
H	-5.69779200	5.48104100	2.49606200	H	5.694377000	5.502557000	-2.495124000
H	-5.69779200	5.48104100	-2.49606200	H	5.694376000	5.502558000	2.495119000
H	-3.71971300	4.02264300	-2.49498000	H	3.722462000	4.032071000	2.492927000
H	-3.71971300	4.02264300	2.49498000	H	3.722464000	4.032069000	-2.492932000
C	-7.95450000	-6.45215200	-1.40929800	C	7.949549000	-6.480363000	1.408792000
C	-7.95450000	-6.45215200	1.40929800	C	7.949548000	-6.480367000	-1.408783000
H	-7.95904700	-6.43426300	-2.49668600	H	7.952768000	-6.467101000	2.496243000
H	-7.95904700	-6.43426300	2.49668600	H	7.952768000	-6.467110000	-2.496234000
C	-9.14977500	6.70194300	-0.73077000	C	9.148970000	6.727776000	0.730468000
C	-10.38799000	6.78724000	-1.40948200	C	10.383045000	6.819445000	1.408815000
C	-11.60032400	6.69181300	-0.73154900	C	11.606671000	6.716304000	0.730380000
C	-11.60032400	6.69181300	0.73154900	C	11.606670000	6.716304000	-0.730383000
C	-10.38799000	6.78724000	1.40948200	C	10.383044000	6.819445000	-1.408818000
C	-9.14977500	6.70194300	0.73077000	C	9.148970000	6.727776000	-0.730472000
H	-10.38749600	6.76841200	-2.49686400	H	10.382903000	6.806087000	2.496269000
H	-10.38749600	6.76841200	2.49686400	H	10.382904000	6.806088000	-2.496272000
C	-2.85825300	-3.17556900	-0.72661900	C	2.856646000	-3.176505000	0.723561000
C	-3.70800300	-4.03608800	-1.40746000	C	3.710664000	-4.045567000	1.405374000
C	-3.70800300	-4.03608800	1.40746000	C	3.710664000	-4.045571000	-1.405370000
C	-2.85825300	-3.17556900	0.72661900	C	2.856646000	-3.176506000	-0.723559000
H	-3.71971300	-4.02264300	-2.49498000	H	3.722405000	-4.031617000	2.492929000
H	-3.71971300	-4.02264300	2.49498000	H	3.722404000	-4.031623000	-2.492925000
C	-4.66337300	-4.82855700	-0.72849200	C	4.656571000	-4.836611000	0.726653000
C	-5.68936400	-5.49664100	-1.40865600	C	5.687378000	-5.515995000	1.407653000
C	-6.78948600	-6.04461300	-0.72980800	C	6.782583000	-6.063027000	0.729363000
C	-6.78948600	-6.04461300	0.72980800	C	6.782583000	-6.063029000	-0.729356000
C	-5.68936400	-5.49664100	1.40865600	C	5.687378000	-5.515999000	-1.407646000
C	-4.66337300	-4.82855700	0.72849200	C	4.656571000	-4.836613000	-0.726648000
H	-5.69779200	-5.48104100	-2.49606200	H	5.694232000	-5.502214000	2.495124000
H	-5.69779200	-5.48104100	2.49606200	H	5.694231000	-5.502221000	-2.495118000
C	-9.14977500	-6.70194300	-0.73077000	C	9.148723000	-6.727723000	0.730475000
C	-10.38799000	-6.78724000	-1.40948200	C	10.382789000	-6.819499000	1.408822000
C	-11.60032400	-6.69181300	-0.73154900	C	11.606425000	-6.716459000	0.730386000
C	-11.60032400	-6.69181300	0.73154900	C	11.606425000	-6.716461000	-0.730376000
C	-10.38799000	-6.78724000	1.40948200	C	10.382788000	-6.819503000	-1.408811000
C	-9.14977500	-6.70194300	0.73077000	C	9.148723000	-6.727726000	-0.730466000
H	-10.38749600	-6.76841200	-2.49686400	H	10.382650000	-6.806151000	2.496275000
H	-10.38749600	-6.76841200	2.49686400	H	10.382650000	-6.806159000	-2.496265000
C	2.07899400	2.18198500	1.40689300	C	-2.082932000	2.186433000	-1.404565000
C	2.85825300	3.17556900	0.72661900	C	-2.856687000	3.176977000	-0.723558000
C	3.70800300	4.03608800	1.40746000	C	-3.710736000	4.046007000	-1.405371000
C	2.85825300	3.17556900	-0.72661900	C	-2.856688000	3.176976000	0.723563000
C	3.70800300	4.03608800	-1.40746000	C	-3.710737000	4.046006000	1.405375000
C	4.66337300	4.82855700	-0.72849200	C	-4.656683000	4.837004000	0.726653000
C	4.66337300	4.82855700	0.72849200	C	-4.656683000	4.837004000	-0.726648000
C	1.41349000	1.20861700	0.72252200	C	-1.416982000	1.208035000	-0.719416000

C	2.07899400	2.18198500	-1.40689300	C	-2.082935000	2.186431000	1.404570000
H	3.71971300	4.02264300	-2.49498000	H	-3.722465000	4.032070000	2.492932000
C	5.68936400	5.49664100	-1.40865600	C	-5.687530000	5.516327000	1.407652000
C	5.68936400	5.49664100	1.40865600	C	-5.687530000	5.516326000	-1.407648000
H	5.69779200	5.48104100	-2.49606200	H	-5.694376000	5.502560000	2.495124000
H	5.69779200	5.48104100	2.49606200	H	-5.694376000	5.502559000	-2.495120000
C	1.41349000	1.20861700	-0.72252200	C	-1.416983000	1.208034000	0.719420000
H	2.10645700	2.16380800	2.49420300	H	-2.109056000	2.168893000	-2.491986000
H	3.71971300	4.02264300	2.49498000	H	-3.722464000	4.032072000	-2.492927000
H	2.10645700	2.16380800	-2.49420300	H	-2.109060000	2.168889000	2.491990000
C	6.78948600	6.04461300	0.72980800	C	-6.782776000	6.063277000	-0.729357000
C	6.78948600	6.04461300	-0.72980800	C	-6.782776000	6.063277000	0.729361000
C	5.68936400	-5.49664100	1.40865600	C	-5.687378000	-5.515994000	-1.407653000
C	4.66337300	-4.82855700	0.72849200	C	-4.656571000	-4.836609000	-0.726653000
C	3.70800300	-4.03608800	1.40746000	C	-3.710664000	-4.045566000	-1.405374000
C	4.66337300	-4.82855700	-0.72849200	C	-4.656571000	-4.836611000	0.726647000
C	3.70800300	-4.03608800	-1.40746000	C	-3.710664000	-4.045569000	1.405369000
C	2.85825300	-3.17556900	-0.72661900	C	-2.856646000	-3.176504000	0.723559000
C	2.85825300	-3.17556900	0.72661900	C	-2.856646000	-3.176503000	-0.723561000
C	5.68936400	-5.49664100	-1.40865600	C	-5.687378000	-5.515997000	1.407646000
C	2.07899400	-2.18198500	-1.40689300	C	-2.082918000	-2.185943000	1.404568000
C	2.07899400	-2.18198500	1.40689300	C	-2.082917000	-2.185941000	-1.404570000
H	5.69779200	-5.48104100	2.49606200	H	-5.694232000	-5.502212000	-2.495125000
H	5.69779200	-5.48104100	-2.49606200	H	-5.694231000	-5.502219000	2.495117000
C	1.41349000	-1.20861700	0.72252200	C	-1.416981000	-1.207534000	-0.719416000
C	1.41349000	-1.20861700	-0.72252200	C	-1.416982000	-1.207535000	0.719416000
C	0.79707000	0.00000000	-1.39912100	C	-0.800708000	0.000250000	1.396418000
C	0.79707000	0.00000000	1.39912100	C	-0.800708000	0.000251000	-1.396416000
H	3.71971300	-4.02264300	-2.49498000	H	-3.722404000	-4.031621000	2.492925000
H	3.71971300	-4.02264300	2.49498000	H	-3.722404000	-4.031616000	-2.492929000
H	2.10645700	-2.16380800	2.49420300	H	-2.109052000	-2.168393000	-2.491989000
H	2.10645700	-2.16380800	-2.49420300	H	-2.109053000	-2.168395000	2.491988000
H	1.10337000	0.00000000	-2.44836100	H	-1.104374000	0.000247000	2.446683000
H	1.10337000	0.00000000	2.44836100	H	-1.104376000	0.000252000	-2.446681000
C	17.57802200	1.22489000	1.40698200	C	-17.561189000	1.226829000	-1.408245000
C	17.67923100	0.00000000	0.73351200	C	-17.671942000	-0.000247000	-0.729148000
C	17.57802200	-1.22489000	1.40698200	C	-17.561167000	-1.227316000	-1.408246000
C	17.67923100	0.00000000	-0.73351200	C	-17.671941000	-0.000248000	0.729153000
C	17.57802200	-1.22489000	-1.40698200	C	-17.561165000	-1.227318000	1.408250000
C	17.21335600	-2.40729200	-0.73332900	C	-17.209725000	-2.407406000	0.729240000
C	17.21335600	-2.40729200	0.73332900	C	-17.209726000	-2.407405000	-0.729238000
C	17.21335600	2.40729200	0.73332900	C	-17.209774000	2.406922000	-0.729236000
C	17.57802200	1.22489000	-1.40698200	C	-17.561186000	1.226828000	1.408251000
H	17.56938400	-1.22333200	-2.49462600	H	-17.542435000	-1.223705000	2.495610000
C	16.66699800	-3.49866900	-1.40766100	C	-16.655288000	-3.508358000	1.408258000
C	16.66699800	-3.49866900	1.40766100	C	-16.655290000	-3.508356000	-1.408258000
H	16.65708400	-3.49266600	-2.49523300	H	-16.640057000	-3.498652000	2.495642000
H	16.65708400	-3.49266600	2.49523300	H	-16.640060000	-3.498648000	-2.495643000
C	17.21335600	2.40729200	-0.73332900	C	-17.209773000	2.406921000	0.729242000
H	17.56938400	1.22333200	2.49462600	H	-17.542454000	1.223213000	-2.495605000
H	17.56938400	-1.22333200	2.49462600	H	-17.542439000	-1.223703000	-2.495606000
H	17.56938400	1.22333200	-2.49462600	H	-17.542450000	1.223211000	2.495611000
C	7.95450000	6.45215200	1.40929800	C	-7.949774000	6.480520000	-1.408786000
C	12.81421700	6.41084200	1.40923800	C	-12.809338000	6.439712000	-1.408517000
C	12.81421700	6.41084200	-1.40923800	C	-12.809337000	6.439712000	1.408519000
C	13.93481100	5.94547500	-0.73222700	C	-13.943512000	5.961024000	0.729909000
C	13.93481100	5.94547500	0.73222700	C	-13.943512000	5.961024000	-0.729906000
C	7.95450000	6.45215200	-1.40929800	C	-7.949774000	6.480522000	1.408790000
C	14.99639900	5.29210100	-1.40856400	C	-14.990110000	5.311501000	1.408325000
C	14.99639900	5.29210100	1.40856400	C	-14.990111000	5.311501000	-1.408321000

H	7.95904700	6.43426300	2.49668600	H	-7.952992000	6.467263000	-2.496237000
H	7.95904700	6.43426300	-2.49668600	H	-7.952991000	6.467266000	2.496241000
C	15.89539700	4.47429700	0.73286400	C	-15.899477000	4.479514000	-0.729495000
C	15.89539700	4.47429700	-0.73286400	C	-15.899476000	4.479514000	0.729500000
C	16.66699800	3.49866900	-1.40766100	C	-16.655365000	3.507890000	1.408261000
C	16.66699800	3.49866900	1.40766100	C	-16.655367000	3.507891000	-1.408256000
H	12.80840300	6.39391600	-2.49666900	H	-12.804940000	6.425849000	2.495964000
H	12.80840300	6.39391600	2.49666900	H	-12.804940000	6.425849000	-2.495962000
H	14.98684600	5.28005800	2.49607200	H	-14.980221000	5.298297000	-2.495736000
H	14.98684600	5.28005800	-2.49607200	H	-14.980218000	5.298297000	2.495740000
H	16.65708400	3.49266600	-2.49523300	H	-16.640120000	3.498173000	2.495645000
H	16.65708400	3.49266600	2.49523300	H	-16.640123000	3.498173000	-2.495639000
C	10.38799000	-6.78724000	-1.40948200	C	-10.382788000	-6.819502000	1.408811000
C	10.38799000	-6.78724000	1.40948200	C	-10.382788000	-6.819499000	-1.408822000
H	10.38749600	-6.76841200	-2.49686400	H	-10.382650000	-6.806157000	2.496265000
H	10.38749600	-6.76841200	2.49686400	H	-10.382650000	-6.806150000	-2.496275000
C	11.60032400	6.69181300	-0.73154900	C	-11.606670000	6.716303000	0.730382000
C	10.38799000	6.78724000	-1.40948200	C	-10.383044000	6.819446000	1.408818000
C	9.14977500	6.70194300	-0.73077000	C	-9.148969000	6.727778000	0.730472000
C	9.14977500	6.70194300	0.73077000	C	-9.148969000	6.727777000	-0.730469000
C	10.38799000	6.78724000	1.40948200	C	-10.383045000	6.819445000	-1.408815000
C	11.60032400	6.69181300	0.73154900	C	-11.606670000	6.716302000	-0.730380000
H	10.38749600	6.76841200	-2.49686400	H	-10.382904000	6.806090000	2.496272000
H	10.38749600	6.76841200	2.49686400	H	-10.382902000	6.806088000	-2.496269000
C	15.89539700	-4.47429700	-0.73286400	C	-15.899363000	-4.479954000	0.729496000
C	14.99639900	-5.29210100	-1.40856400	C	-14.989958000	-5.311900000	1.408321000
C	14.99639900	-5.29210100	1.40856400	C	-14.989959000	-5.311898000	-1.408325000
C	15.89539700	-4.47429700	0.73286400	C	-15.899364000	-4.479953000	-0.729498000
H	14.98684600	-5.28005800	-2.49607200	H	-14.980082000	-5.298715000	2.495736000
H	14.98684600	-5.28005800	2.49607200	H	-14.980085000	-5.298712000	-2.495741000
C	13.93481100	-5.94547500	-0.73222700	C	-13.943321000	-5.961357000	0.729904000
C	12.81421700	-6.41084200	-1.40923800	C	-12.809112000	-6.439970000	1.408514000
C	11.60032400	-6.69181300	-0.73154900	C	-11.606425000	-6.716461000	0.730376000
C	11.60032400	-6.69181300	0.73154900	C	-11.606425000	-6.716459000	-0.730386000
C	12.81421700	-6.41084200	1.40923800	C	-12.809113000	-6.439967000	-1.408523000
C	13.93481100	-5.94547500	0.73222700	C	-13.943322000	-5.961356000	-0.729911000
H	12.80840300	-6.39391600	-2.49666900	H	-12.804724000	-6.426129000	2.495959000
H	12.80840300	-6.39391600	2.49666900	H	-12.804725000	-6.426124000	-2.495968000
C	9.14977500	-6.70194300	-0.73077000	C	-9.148723000	-6.727725000	0.730465000
C	7.95450000	-6.45215200	-1.40929800	C	-7.949548000	-6.480366000	1.408783000
C	6.78948600	-6.04461300	-0.72980800	C	-6.782583000	-6.063027000	0.729355000
C	6.78948600	-6.04461300	0.72980800	C	-6.782583000	-6.063025000	-0.729363000
C	7.95450000	-6.45215200	1.40929800	C	-7.949548000	-6.480362000	-1.408792000
C	9.14977500	-6.70194300	0.73077000	C	-9.148723000	-6.727723000	-0.730475000
H	7.95904700	-6.43426300	-2.49668600	H	-7.952768000	-6.467108000	2.496234000
H	7.95904700	-6.43426300	2.49668600	H	-7.952768000	-6.467100000	-2.496243000
**Dcyc20_R**				**Dcyc20_U**			
C	2.06830600	2.18876200	1.40697200	C	2.06467800	2.19530300	1.40460200
C	5.60880900	5.58299400	1.40879200	C	5.59500200	5.60864600	1.40765000
C	5.60880900	5.58299400	-1.40879200	C	5.59500200	5.60864600	-1.40765000
C	6.68826500	6.17349000	-0.73029500	C	6.66780500	6.19852800	-0.72937800
C	6.68826500	6.17349000	0.73029500	C	6.66780500	6.19852800	0.72937800
C	1.41144600	1.20993100	0.72256200	C	1.40908500	1.21060700	0.71954000
C	2.06830600	2.18876200	-1.40697200	C	2.06467800	2.19530300	-1.40460200
H	5.61711800	5.56878300	-2.49646500	H	5.60175400	5.59608000	-2.49512700
C	7.83070900	6.63561700	-1.40964600	C	7.81338400	6.67054700	-1.40877100
C	7.83070900	6.63561700	1.40964600	C	7.81338400	6.67054700	1.40877100
H	7.83582200	6.61917000	-2.49704000	H	7.81696300	6.65849400	-2.49623200

H	7.83582200	6.61917000	2.49704000	H	7.81696300	6.65849400	2.49623200
C	1.41144600	1.20993100	-0.72256200	C	1.40908500	1.21060700	-0.71954000
H	2.09359500	2.17158000	2.49432900	H	2.08967500	2.17856200	2.49200300
H	5.61711800	5.56878300	2.49646500	H	5.60175400	5.59608000	2.49512700
H	2.09359500	2.17158000	-2.49432900	H	2.08967500	2.17856200	-2.49200300
C	9.01150700	6.95305900	0.73098800	C	8.99645500	6.98593400	0.73050100
C	9.01150700	6.95305900	-0.73098800	C	8.99645500	6.98593400	-0.73050100
C	5.60880900	-5.58299400	1.40879200	C	5.59500200	-5.60864600	1.40765000
C	4.60565800	-4.88283100	0.72877100	C	4.58670600	-4.89691300	0.72669200
C	3.66946400	-4.06655900	1.40761200	C	3.66087600	-4.08232500	1.40545300
C	4.60565800	-4.88283100	-0.72877100	C	4.58670600	-4.89691300	-0.72669200
C	3.66946400	-4.06655900	-1.40761200	C	3.66087600	-4.08232500	-1.40545300
C	2.83673500	-3.19065200	-0.72718800	C	2.82441800	-3.19687700	-0.72360300
C	2.83673500	-3.19065200	0.72718800	C	2.82441800	-3.19687700	0.72360300
C	5.60880900	-5.58299400	-1.40879200	C	5.59500200	-5.60864600	-1.40765000
C	2.06830600	-2.18876200	-1.40697200	C	2.06467800	-2.19530300	-1.40460200
C	2.06830600	-2.18876200	1.40697200	C	2.06467800	-2.19530300	1.40460200
H	5.61711800	-5.56878300	2.49646500	H	5.60175400	-5.59608000	2.49512700
H	5.61711800	-5.56878300	-2.49646500	H	5.60175400	-5.59608000	-2.49512700
C	1.41144600	-1.20993100	0.72256200	C	1.40908500	-1.21060700	0.71954000
C	1.41144600	-1.20993100	-0.72256200	C	1.40908500	-1.21060700	-0.71954000
C	0.79737600	0.00000000	-1.39972000	C	0.79789400	0.00000000	-1.39812800
C	0.79737600	0.00000000	1.39972000	C	0.79789400	0.00000000	1.39812800
H	3.68084400	-4.05401000	-2.49541500	H	3.67191800	-4.06959400	-2.49300700
H	3.68084400	-4.05401000	2.49541500	H	3.67191800	-4.06959400	2.49300700
H	2.09359500	-2.17158000	2.49432900	H	2.08967500	-2.17856200	2.49200300
H	2.09359500	-2.17158000	-2.49432900	H	2.08967500	-2.17856200	-2.49200300
H	1.10404700	0.00000000	-2.44883000	H	1.10537800	0.00000000	-2.44705900
H	1.10404700	0.00000000	2.44883000	H	1.10537800	0.00000000	2.44705900
C	18.57601200	0.00000000	1.40733100	C	18.54495200	0.00000000	1.40829000
C	18.44943800	-1.22632500	0.73368800	C	18.42803900	-1.22598400	0.72928400
C	18.13314200	-2.40937800	1.40765700	C	18.10498700	-2.41500300	1.40826800
C	18.44943800	-1.22632500	-0.73368800	C	18.42803900	-1.22598400	-0.72928400
C	18.13314200	-2.40937800	-1.40765700	C	18.10498700	-2.41500300	-1.40826800
C	17.58075500	-3.51937800	-0.73301900	C	17.56597100	-3.52175600	-0.72937400
C	17.58075500	-3.51937800	0.73301900	C	17.56597100	-3.52175600	0.72937400
C	18.44943800	1.22632500	0.73368800	C	18.42803900	1.22598400	0.72928400
C	18.57601200	0.00000000	-1.40733100	C	18.54495200	0.00000000	-1.40829000
H	18.12387100	-2.40598100	-2.49487300	H	18.08849400	-2.40879800	-2.49565600
C	16.87323000	-4.51107600	-1.40809100	C	16.85111600	-4.52578100	-1.40824200
C	16.87323000	-4.51107600	1.40809100	C	16.85111600	-4.52578100	1.40824200
H	16.86320400	-4.50274300	-2.49565000	H	16.83848200	-4.51490800	-2.49564900
H	16.86320400	-4.50274300	2.49565000	H	16.83848200	-4.51490800	2.49564900
C	18.44943800	1.22632500	-0.73368800	C	18.42803900	1.22598400	-0.72928400
H	18.56727100	0.00000000	2.49497500	H	18.52698800	0.00000000	2.49566700
H	18.12387100	-2.40598100	2.49487300	H	18.08849400	-2.40879800	2.49565600
H	18.56727100	0.00000000	-2.49497500	H	18.52698800	0.00000000	-2.49566700
C	10.23908400	7.12044500	1.40973500	C	10.22148600	7.15921200	1.40879500
C	14.97872700	6.06725200	1.40914000	C	14.96112500	6.09356300	1.40827900
C	14.97872700	6.06725200	-1.40914000	C	14.96112500	6.09356300	-1.40827900
C	15.97495800	5.37170200	-0.73260900	C	15.96805700	5.38323700	-0.72957900
C	15.97495800	5.37170200	0.73260900	C	15.96805700	5.38323700	0.72957900
C	10.23908400	7.12044500	-1.40973500	C	10.22148600	7.15921200	-1.40879500
C	16.87323000	4.51107600	-1.40809100	C	16.85111600	4.52578100	-1.40824200
C	16.87323000	4.51107600	1.40809100	C	16.85111600	4.52578100	1.40824200
H	10.23989500	7.10270400	2.49712800	H	10.22224400	7.14696000	2.49626100
H	10.23989500	7.10270400	-2.49712800	H	10.22224400	7.14696000	-2.49626100
C	17.58075500	3.51937800	0.73301900	C	17.56597100	3.52175600	0.72937400
C	17.58075500	3.51937800	-0.73301900	C	17.56597100	3.52175600	-0.72937400
C	18.13314200	2.40937800	-1.40765700	C	18.10498700	2.41500300	-1.40826800

C	18.13314200	2.40937800	1.40765700	C	18.10498700	2.41500300	1.40826800
H	14.97060500	6.05362700	-2.49642000	H	14.95358400	6.08050700	-2.49571900
H	14.97060500	6.05362700	2.49642000	H	14.95358400	6.08050700	2.49571900
H	16.86320400	4.50274300	2.49565000	H	16.83848200	4.51490800	2.49564900
H	16.86320400	4.50274300	-2.49565000	H	16.83848200	4.51490800	-2.49564900
H	18.12387100	2.40598100	-2.49487300	H	18.08849400	2.40879800	-2.49565600
H	18.12387100	2.40598100	2.49487300	H	18.08849400	2.40879800	2.49565600
C	10.23908400	-7.12044500	-1.40973500	C	10.22148600	-7.15921200	-1.40879500
C	10.23908400	-7.12044500	1.40973500	C	10.22148600	-7.15921200	1.40879500
H	10.23989500	-7.10270400	-2.49712800	H	10.22224400	-7.14696000	-2.49626100
H	10.23989500	-7.10270400	2.49712800	H	10.22224400	-7.14696000	2.49626100
C	13.85184600	6.59740100	-0.73216000	C	13.84808100	6.62094800	-0.72997200
C	12.68822000	6.94677700	-1.40955100	C	12.67150600	6.98231900	-1.40848700
C	11.45684400	7.11931000	-0.73167900	C	11.44929200	7.15168500	-0.73042600
C	11.45684400	7.11931000	0.73167900	C	11.44929200	7.15168500	0.73042600
C	12.68822000	6.94677700	1.40955100	C	12.67150600	6.98231900	1.40848700
C	13.85184600	6.59740100	0.73216000	C	13.84808100	6.62094800	0.72997200
H	12.68416400	6.92967900	-2.49697200	H	12.66868100	6.96919400	-2.49594700
H	12.68416400	6.92967900	2.49697200	H	12.66868100	6.96919400	2.49594700
C	15.97495800	-5.37170200	-0.73260900	C	15.96805700	-5.38323700	-0.72957900
C	14.97872700	-6.06725200	-1.40914000	C	14.96112500	-6.09356300	-1.40827900
C	14.97872700	-6.06725200	1.40914000	C	14.96112500	-6.09356300	1.40827900
C	15.97495800	-5.37170200	0.73260900	C	15.96805700	-5.38323700	0.72957900
H	14.97060500	-6.05362700	-2.49642000	H	14.95358400	-6.08050700	-2.49571900
H	14.97060500	-6.05362700	2.49642000	H	14.95358400	-6.08050700	2.49571900
C	13.85184600	-6.59740100	-0.73216000	C	13.84808100	-6.62094800	-0.72997200
C	12.68822000	-6.94677700	-1.40955100	C	12.67150600	-6.98231900	-1.40848700
C	11.45684400	-7.11931000	-0.73167900	C	11.44929200	-7.15168500	-0.73042600
C	11.45684400	-7.11931000	0.73167900	C	11.44929200	-7.15168500	0.73042600
C	12.68822000	-6.94677700	1.40955100	C	12.67150600	-6.98231900	1.40848700
C	13.85184600	-6.59740100	0.73216000	C	13.84808100	-6.62094800	0.72997200
H	12.68416400	-6.92967900	-2.49697200	H	12.66868100	-6.96919400	-2.49594700
H	12.68416400	-6.92967900	2.49697200	H	12.66868100	-6.96919400	2.49594700
C	9.01150700	-6.95305900	-0.73098800	C	8.99645500	-6.98593400	-0.73050100
C	7.83070900	-6.63561700	-1.40964600	C	7.81338400	-6.67054700	-1.40877100
C	6.68826500	-6.17349000	-0.73029500	C	6.66780500	-6.19852800	-0.72937800
C	6.68826500	-6.17349000	0.73029500	C	6.66780500	-6.19852800	0.72937800
C	7.83070900	-6.63561700	1.40964600	C	7.81338400	-6.67054700	1.40877100
C	9.01150700	-6.95305900	0.73098800	C	8.99645500	-6.98593400	0.73050100
H	7.83582200	-6.61917000	-2.49704000	H	7.81696300	-6.65849400	-2.49623200
H	7.83582200	-6.61917000	2.49704000	H	7.81696300	-6.65849400	2.49623200
C	4.60565800	4.88283100	-0.72877100	C	4.58670600	4.89691300	-0.72669200
C	3.66946400	4.06655900	-1.40761200	C	3.66087600	4.08232500	-1.40545300
C	2.83673500	3.19065200	-0.72718800	C	2.82441800	3.19687700	-0.72360300
C	2.83673500	3.19065200	0.72718800	C	2.82441800	3.19687700	0.72360300
C	3.66946400	4.06655900	1.40761200	C	3.66087600	4.08232500	1.40545300
C	4.60565800	4.88283100	0.72877100	C	4.58670600	4.89691300	0.72669200
H	3.68084400	4.05401000	-2.49541500	H	3.67191800	4.06959400	-2.49300700
H	3.68084400	4.05401000	2.49541500	H	3.67191800	4.06959400	2.49300700
C	-18.13314200	2.40937800	1.40765700	C	-18.10498700	2.41500300	1.40826800
C	-14.97872700	6.06725200	1.40914000	C	-14.96112500	6.09356300	1.40827900
C	-14.97872700	6.06725200	-1.40914000	C	-14.96112500	6.09356300	-1.40827900
C	-13.85184600	6.59740100	-0.73216000	C	-13.84808100	6.62094800	-0.72997200
C	-13.85184600	6.59740100	0.73216000	C	-13.84808100	6.62094800	0.72997200
C	-18.44943800	1.22632500	0.73368800	C	-18.42803900	1.22598400	0.72928400
C	-18.13314200	2.40937800	-1.40765700	C	-18.10498700	2.41500300	-1.40826800
H	-14.97060500	6.05362700	-2.49642000	H	-14.95358400	6.08050700	-2.49571900
C	-12.68822000	6.94677700	-1.40955100	C	-12.67150600	6.98231900	-1.40848700
C	-12.68822000	6.94677700	1.40955100	C	-12.67150600	6.98231900	1.40848700
H	-12.68416400	6.92967900	-2.49697200	H	-12.66868100	6.96919400	-2.49594700

H	-12.68416400	6.92967900	2.49697200	H	-12.66868100	6.96919400	2.49594700
C	-18.44943800	1.22632500	-0.73368800	C	-18.42803900	1.22598400	-0.72928400
H	-18.12387100	2.40598100	2.49487300	H	-18.08849400	2.40879800	2.49565600
H	-14.97060500	6.05362700	2.49642000	H	-14.95358400	6.08050700	2.49571900
H	-18.12387100	2.40598100	-2.49487300	H	-18.08849400	2.40879800	-2.49565600
C	-11.45684400	7.11931000	0.73167900	C	-11.44929200	7.15168500	0.73042600
C	-11.45684400	7.11931000	-0.73167900	C	-11.44929200	7.15168500	-0.73042600
C	-14.97872700	-6.06725200	1.40914000	C	-14.96112500	-6.09356300	1.40827900
C	-15.97495800	-5.37170200	0.73260900	C	-15.96805700	-5.38323700	0.72957900
C	-16.87323000	-4.51107600	1.40809100	C	-16.85111600	-4.52578100	1.40824200
C	-15.97495800	-5.37170200	-0.73260900	C	-15.96805700	-5.38323700	-0.72957900
C	-16.87323000	-4.51107600	-1.40809100	C	-16.85111600	-4.52578100	-1.40824200
C	-17.58075500	-3.51937800	-0.73301900	C	-17.56597100	-3.52175600	-0.72937400
C	-17.58075500	-3.51937800	0.73301900	C	-17.56597100	-3.52175600	0.72937400
C	-14.97872700	-6.06725200	-1.40914000	C	-14.96112500	-6.09356300	-1.40827900
C	-18.13314200	-2.40937800	-1.40765700	C	-18.10498700	-2.41500300	-1.40826800
C	-18.13314200	-2.40937800	1.40765700	C	-18.10498700	-2.41500300	1.40826800
H	-14.97060500	-6.05362700	2.49642000	H	-14.95358400	-6.08050700	2.49571900
H	-14.97060500	-6.05362700	-2.49642000	H	-14.95358400	-6.08050700	-2.49571900
C	-18.44943800	-1.22632500	0.73368800	C	-18.42803900	-1.22598400	0.72928400
C	-18.44943800	-1.22632500	-0.73368800	C	-18.42803900	-1.22598400	-0.72928400
C	-18.57601200	0.00000000	-1.40733100	C	-18.54495200	0.00000000	-1.40829000
C	-18.57601200	0.00000000	1.40733100	C	-18.54495200	0.00000000	1.40829000
H	-16.86320400	-4.50274300	-2.49565000	H	-16.83848200	-4.51490800	-2.49564900
H	-16.86320400	-4.50274300	2.49565000	H	-16.83848200	-4.51490800	2.49564900
H	-18.12387100	-2.40598100	2.49487300	H	-18.08849400	-2.40879800	2.49565600
H	-18.12387100	-2.40598100	-2.49487300	H	-18.08849400	-2.40879800	-2.49565600
H	-18.56727100	0.00000000	-2.49497500	H	-18.52698800	0.00000000	-2.49566700
H	-18.56727100	0.00000000	2.49497500	H	-18.52698800	0.00000000	2.49566700
C	-0.79737600	0.00000000	1.39972000	C	-0.79789400	0.00000000	1.39812800
C	-1.41144600	-1.20993100	0.72256200	C	-1.40908500	-1.21060700	0.71954000
C	-2.06830600	-2.18876200	1.40697200	C	-2.06467800	-2.19530300	1.40460200
C	-1.41144600	-1.20993100	-0.72256200	C	-1.40908500	-1.21060700	-0.71954000
C	-2.06830600	-2.18876200	-1.40697200	C	-2.06467800	-2.19530300	-1.40460200
C	-2.83673500	-3.19065200	-0.72718800	C	-2.82441800	-3.19687700	-0.72360300
C	-2.83673500	-3.19065200	0.72718800	C	-2.82441800	-3.19687700	0.72360300
C	-1.41144600	1.20993100	0.72256200	C	-1.40908500	1.21060700	0.71954000
C	-0.79737600	0.00000000	-1.39972000	C	-0.79789400	0.00000000	-1.39812800
H	-2.09359500	-2.17158000	-2.49432900	H	-2.08967500	-2.17856200	-2.49200300
C	-3.66946400	-4.06655900	-1.40761200	C	-3.66087600	-4.08232500	-1.40545300
C	-3.66946400	-4.06655900	1.40761200	C	-3.66087600	-4.08232500	1.40545300
H	-3.68084400	-4.05401000	-2.49541500	H	-3.67191800	-4.06959400	-2.49300700
H	-3.68084400	-4.05401000	2.49541500	H	-3.67191800	-4.06959400	2.49300700
C	-1.41144600	1.20993100	-0.72256200	C	-1.40908500	1.21060700	-0.71954000
H	-1.10404700	0.00000000	2.44883000	H	-1.10537800	0.00000000	2.44705900
H	-2.09359500	-2.17158000	2.49432900	H	-2.08967500	-2.17856200	2.49200300
H	-1.10404700	0.00000000	-2.44883000	H	-1.10537800	0.00000000	-2.44705900
C	-10.23908400	7.12044500	1.40973500	C	-10.22148600	7.15921200	1.40879500
C	-5.60880900	5.58299400	1.40879200	C	-5.59500200	5.60864600	1.40765000
C	-5.60880900	5.58299400	-1.40879200	C	-5.59500200	5.60864600	-1.40765000
C	-4.60565800	4.88283100	-0.72877100	C	-4.58670600	4.89691300	-0.72669200
C	-4.60565800	4.88283100	0.72877100	C	-4.58670600	4.89691300	0.72669200
C	-10.23908400	7.12044500	-1.40973500	C	-10.22148600	7.15921200	-1.40879500
C	-3.66946400	4.06655900	-1.40761200	C	-3.66087600	4.08232500	-1.40545300
C	-3.66946400	4.06655900	1.40761200	C	-3.66087600	4.08232500	1.40545300
H	-10.23989500	7.10270400	2.49712800	H	-10.22224400	7.14696000	2.49626100
H	-10.23989500	7.10270400	-2.49712800	H	-10.22224400	7.14696000	-2.49626100
C	-2.83673500	3.19065200	0.72718800	C	-2.82441800	3.19687700	0.72360300
C	-2.83673500	3.19065200	-0.72718800	C	-2.82441800	3.19687700	-0.72360300
C	-2.06830600	2.18876200	-1.40697200	C	-2.06467800	2.19530300	-1.40460200

C	-2.06830600	2.18876200	1.40697200	C	-2.06467800	2.19530300	1.40460200
H	-5.61711800	5.56878300	-2.49646500	H	-5.60175400	5.59608000	-2.49512700
H	-5.61711800	5.56878300	2.49646500	H	-5.60175400	5.59608000	2.49512700
H	-3.68084400	4.05401000	2.49541500	H	-3.67191800	4.06959400	2.49300700
H	-3.68084400	4.05401000	-2.49541500	H	-3.67191800	4.06959400	-2.49300700
H	-2.09359500	2.17158000	-2.49432900	H	-2.08967500	2.17856200	-2.49200300
H	-2.09359500	2.17158000	2.49432900	H	-2.08967500	2.17856200	2.49200300
C	-10.23908400	-7.12044500	-1.40973500	C	-10.22148600	-7.15921200	-1.40879500
C	-10.23908400	-7.12044500	1.40973500	C	-10.22148600	-7.15921200	1.40879500
H	-10.23989500	-7.10270400	-2.49712800	H	-10.22224400	-7.14696000	-2.49626100
H	-10.23989500	-7.10270400	2.49712800	H	-10.22224400	-7.14696000	2.49626100
C	-6.68826500	6.17349000	-0.73029500	C	-6.66780500	6.19852800	-0.72937800
C	-7.83070900	6.63561700	-1.40964600	C	-7.81338400	6.67054700	-1.40877100
C	-9.01150700	6.95305900	-0.73098800	C	-8.99645500	6.98593400	-0.73050100
C	-9.01150700	6.95305900	0.73098800	C	-8.99645500	6.98593400	0.73050100
C	-7.83070900	6.63561700	1.40964600	C	-7.81338400	6.67054700	1.40877100
C	-6.68826500	6.17349000	0.73029500	C	-6.66780500	6.19852800	0.72937800
H	-7.83582200	6.61917000	-2.49704000	H	-7.81696300	6.65849400	-2.49623200
H	-7.83582200	6.61917000	2.49704000	H	-7.81696300	6.65849400	2.49623200
C	-4.60565800	-4.88283100	-0.72877100	C	-4.58670600	-4.89691300	-0.72669200
C	-5.60880900	-5.58299400	-1.40879200	C	-5.59500200	-5.60864600	-1.40765000
C	-5.60880900	-5.58299400	1.40879200	C	-5.59500200	-5.60864600	1.40765000
C	-4.60565800	-4.88283100	0.72877100	C	-4.58670600	-4.89691300	0.72669200
H	-5.61711800	-5.56878300	-2.49646500	H	-5.60175400	-5.59608000	-2.49512700
H	-5.61711800	-5.56878300	2.49646500	H	-5.60175400	-5.59608000	2.49512700
C	-6.68826500	-6.17349000	-0.73029500	C	-6.66780500	-6.19852800	-0.72937800
C	-7.83070900	-6.63561700	-1.40964600	C	-7.81338400	-6.67054700	-1.40877100
C	-9.01150700	-6.95305900	-0.73098800	C	-8.99645500	-6.98593400	-0.73050100
C	-9.01150700	-6.95305900	0.73098800	C	-8.99645500	-6.98593400	0.73050100
C	-7.83070900	-6.63561700	1.40964600	C	-7.81338400	-6.67054700	1.40877100
C	-6.68826500	-6.17349000	0.73029500	C	-6.66780500	-6.19852800	0.72937800
H	-7.83582200	-6.61917000	-2.49704000	H	-7.81696300	-6.65849400	-2.49623200
H	-7.83582200	-6.61917000	2.49704000	H	-7.81696300	-6.65849400	2.49623200
C	-11.45684400	-7.11931000	-0.73167900	C	-11.44929200	-7.15168500	-0.73042600
C	-12.68822000	-6.94677700	-1.40955100	C	-12.67150600	-6.98231900	-1.40848700
C	-13.85184600	-6.59740100	-0.73216000	C	-13.84808100	-6.62094800	-0.72997200
C	-13.85184600	-6.59740100	0.73216000	C	-13.84808100	-6.62094800	0.72997200
C	-12.68822000	-6.94677700	1.40955100	C	-12.67150600	-6.98231900	1.40848700
C	-11.45684400	-7.11931000	0.73167900	C	-11.44929200	-7.15168500	0.73042600
H	-12.68416400	-6.92967900	-2.49697200	H	-12.66868100	-6.96919400	-2.49594700
H	-12.68416400	-6.92967900	2.49697200	H	-12.66868100	-6.96919400	2.49594700
C	-15.97495800	5.37170200	-0.73260900	C	-15.96805700	5.38323700	-0.72957900
C	-16.87323000	4.51107600	-1.40809100	C	-16.85111600	4.52578100	-1.40824200
C	-17.58075500	3.51937800	-0.73301900	C	-17.56597100	3.52175600	-0.72937400
C	-17.58075500	3.51937800	0.73301900	C	-17.56597100	3.52175600	0.72937400
C	-16.87323000	4.51107600	1.40809100	C	-16.85111600	4.52578100	1.40824200
C	-15.97495800	5.37170200	0.73260900	C	-15.96805700	5.38323700	0.72957900
H	-16.86320400	4.50274300	-2.49565000	H	-16.83848200	4.51490800	-2.49564900
H	-16.86320400	4.50274300	2.49565000	H	-16.83848200	4.51490800	2.49564900

Table S11. Cartesian coordinates of the optimized geometries of [n]-cyclacene dimer at M06-2X/6-31G(d) level of theory.

RM06-2X/6-31G(d)				UM06-2X/6-31G(d)			
Dcyc6_R				Dcyc6_U			
C	-1.51261800	1.16770500	0.72589100	C	-1.51262800	1.16769200	0.72590700
C	-2.49669500	1.82339800	1.40507400	C	-2.49667600	1.82339100	1.40507600
C	-3.71324900	2.20598600	0.72187900	C	-3.71322300	2.20606900	0.72186900
C	-3.71324900	2.20598600	-0.72187900	C	-3.71322300	2.20606900	-0.72186900
C	-2.49669500	1.82339800	-1.40507400	C	-2.49667600	1.82339100	-1.40507600
C	-1.51261800	1.16770500	-0.72589100	C	-1.51262800	1.16769200	-0.72590700
C	-4.90116000	1.96486500	1.40439000	C	-4.90110500	1.96489500	1.40437600
C	-4.90116000	1.96486500	-1.40439000	C	-4.90110500	1.96489500	-1.40437600
C	-5.88502500	1.18798600	-0.72072300	C	-5.88494200	1.18798400	-0.72074200
C	-5.88502500	1.18798600	0.72072300	C	-5.88494200	1.18798400	0.72074200
C	-6.22090400	0.00000000	1.40458400	C	-6.22071600	0.00000000	1.40457300
H	-6.15216400	0.00000000	2.48987600	H	-6.15195800	0.00000000	2.48987000
C	-5.88502500	-1.18798600	0.72072300	C	-5.88494200	-1.18798400	0.72074200
C	-5.88502500	-1.18798600	-0.72072300	C	-5.88494200	-1.18798400	-0.72074200
C	-6.22090400	0.00000000	-1.40458400	C	-6.22071600	0.00000000	-1.40457300
H	-4.87126800	1.90528600	2.49010100	H	-4.87115900	1.90532300	2.49009100
H	-2.54016100	1.73976600	2.48941800	H	-2.54014700	1.73972100	2.48942100
H	-2.54016100	1.73976600	-2.48941800	H	-2.54014700	1.73972100	-2.48942100
H	-4.87126800	1.90528600	-2.49010100	H	-4.87115900	1.90532300	-2.49009100
H	-6.15216400	0.00000000	-2.48987600	H	-6.15195800	0.00000000	-2.48987000
C	-4.90116000	-1.96486500	1.40439000	C	-4.90110500	-1.96489500	1.40437600
C	-3.71324900	-2.20598600	0.72187900	C	-3.71322300	-2.20606900	0.72186900
C	-3.71324900	-2.20598600	-0.72187900	C	-3.71322300	-2.20606900	-0.72186900
C	-4.90116000	-1.96486500	-1.40439000	C	-4.90110500	-1.96489500	-1.40437600
C	-2.49669500	-1.82339800	1.40507400	C	-2.49667600	-1.82339100	1.40507600
C	-2.49669500	-1.82339800	-1.40507400	C	-2.49667600	-1.82339100	-1.40507600
C	-1.51261800	-1.16770500	-0.72589100	C	-1.51262800	-1.16769200	-0.72590700
C	-1.51261800	-1.16770500	0.72589100	C	-1.51262800	-1.16769200	0.72590700
C	-0.77915900	0.00000000	1.39329200	C	-0.77916200	0.00000000	1.39330100
H	-1.07814700	0.00000000	2.44540500	H	-1.07816400	0.00000000	2.44541200
C	-0.77915900	0.00000000	-1.39329200	C	-0.77916200	0.00000000	-1.39330100
H	-2.54016100	-1.73976600	2.48941800	H	-2.54014700	-1.73972100	2.48942100
H	-2.54016100	-1.73976600	-2.48941800	H	-2.54014700	-1.73972100	-2.48942100
H	-1.07814700	0.00000000	-2.44540500	H	-1.07816400	0.00000000	-2.44541200
H	-4.87126800	-1.90528600	2.49010100	H	-4.87115900	-1.90532300	2.49009100
H	-4.87126800	-1.90528600	-2.49010100	H	-4.87115900	-1.90532300	-2.49009100
C	3.71324900	-2.20598600	0.72187900	C	3.71322300	-2.20606900	0.72186900
C	4.90116000	-1.96486500	1.40439000	C	4.90110500	-1.96489500	1.40437600
C	5.88502500	-1.18798600	0.72072300	C	5.88494200	-1.18798400	0.72074200
C	5.88502500	-1.18798600	-0.72072300	C	5.88494200	-1.18798400	-0.72074200
C	4.90116000	-1.96486500	-1.40439000	C	4.90110500	-1.96489500	-1.40437600
C	3.71324900	-2.20598600	-0.72187900	C	3.71322300	-2.20606900	-0.72186900
C	6.22090400	0.00000000	1.40458400	C	6.22071600	0.00000000	1.40457300
C	6.22090400	0.00000000	-1.40458400	C	6.22071600	0.00000000	-1.40457300
C	5.88502500	1.18798600	-0.72072300	C	5.88494200	1.18798400	-0.72074200
C	5.88502500	1.18798600	0.72072300	C	5.88494200	1.18798400	0.72074200
C	4.90116000	1.96486500	1.40439000	C	4.90110500	1.96489500	1.40437600
H	4.87126800	1.90528600	2.49010100	H	4.87115900	1.90532300	2.49009100
C	3.71324900	2.20598600	0.72187900	C	3.71322300	2.20606900	0.72186900
C	3.71324900	2.20598600	-0.72187900	C	3.71322300	2.20606900	-0.72186900
C	4.90116000	1.96486500	-1.40439000	C	4.90110500	1.96489500	-1.40437600
H	6.15216400	0.00000000	2.48987600	H	6.15195800	0.00000000	2.48987000

H	4.87126800	-1.90528600	2.49010100	H	4.87115900	-1.90532300	2.49009100
H	4.87126800	-1.90528600	-2.49010100	H	4.87115900	-1.90532300	-2.49009100
H	6.15216400	0.00000000	-2.48987600	H	6.15195800	0.00000000	-2.48987000
H	4.87126800	1.90528600	-2.49010100	H	4.87115900	1.90532300	-2.49009100
C	2.49669500	1.82339800	1.40507400	C	2.49667600	1.82339100	1.40507600
C	1.51261800	1.16770500	0.72589100	C	1.51262800	1.16769200	0.72590700
C	1.51261800	1.16770500	-0.72589100	C	1.51262800	1.16769200	-0.72590700
C	2.49669500	1.82339800	-1.40507400	C	2.49667600	1.82339100	-1.40507600
C	0.77915900	0.00000000	1.39329200	C	0.77916200	0.00000000	1.39330100
C	0.77915900	0.00000000	-1.39329200	C	0.77916200	0.00000000	-1.39330100
C	1.51261800	-1.16770500	-0.72589100	C	1.51262800	-1.16769200	-0.72590700
C	1.51261800	-1.16770500	0.72589100	C	1.51262800	-1.16769200	0.72590700
C	2.49669500	-1.82339800	1.40507400	C	2.49667600	-1.82339100	1.40507600
H	2.54016100	-1.73976600	2.48941800	H	2.54014700	-1.73972100	2.48942100
C	2.49669500	-1.82339800	-1.40507400	C	2.49667600	-1.82339100	-1.40507600
H	1.07814700	0.00000000	2.44540500	H	1.07816400	0.00000000	2.44541200
H	1.07814700	0.00000000	-2.44540500	H	1.07816400	0.00000000	-2.44541200
H	2.54016100	-1.73976600	-2.48941800	H	2.54014700	-1.73972100	-2.48942100
H	2.54016100	1.73976600	2.48941800	H	2.54014700	1.73972100	2.48942100
H	2.54016100	1.73976600	-2.48941800	H	2.54014700	1.73972100	-2.48942100
**Dcyc7_R**				**Dcyc7_U**			
C	-5.94997900	2.08930100	0.72403300	C	-5.94998700	2.08939500	0.72400200
C	-4.76798900	2.50515800	1.40730600	C	-4.76780600	2.50494500	1.40724800
C	-3.56478900	2.44810100	0.72485900	C	-3.56457900	2.44818600	0.72482700
C	-3.56478900	2.44810100	-0.72485900	C	-3.56457900	2.44818600	-0.72482700
C	-4.76798900	2.50515800	-1.40730600	C	-4.76780600	2.50494500	-1.40724800
C	-5.94997900	2.08930100	-0.72403300	C	-5.94998700	2.08939500	-0.72400200
C	-2.40695100	1.91592900	1.40654100	C	-2.40647900	1.91629800	1.40643000
C	-2.40695100	1.91592900	-1.40654100	C	-2.40647900	1.91629800	-1.40643000
C	-1.49101600	1.17463700	-0.72601400	C	-1.49081300	1.17472300	-0.72606400
C	-1.49101600	1.17463700	0.72601400	C	-1.49081300	1.17472300	0.72606400
C	-0.78116500	0.00000000	1.39617800	C	-0.78102800	0.00000000	1.39614800
H	-1.08510300	0.00000000	2.44668500	H	-1.08480100	0.00000000	2.44669700
C	-0.78116500	0.00000000	-1.39617800	C	-0.78102800	0.00000000	-1.39614800
H	-2.44873300	1.85434000	2.49218900	H	-2.44850000	1.85478100	2.49208200
H	-4.75781700	2.44636400	2.49331400	H	-4.75781300	2.44611200	2.49327800
H	-4.75781700	2.44636400	-2.49331400	H	-4.75781300	2.44611200	-2.49327800
H	-2.44873300	1.85434000	-2.49218900	H	-2.44850000	1.85478100	-2.49208200
H	-1.08510300	0.00000000	-2.44668500	H	-1.08480100	0.00000000	-2.44669700
C	-1.49101600	-1.17463700	0.72601400	C	-1.49081300	-1.17472300	0.72606400
C	-2.40695100	-1.91592900	1.40654100	C	-2.40647900	-1.91629800	1.40643000
C	-3.56478900	-2.44810100	0.72485900	C	-3.56457900	-2.44818600	0.72482700
C	-3.56478900	-2.44810100	-0.72485900	C	-3.56457900	-2.44818600	-0.72482700
C	-2.40695100	-1.91592900	-1.40654100	C	-2.40647900	-1.91629800	-1.40643000
C	-1.49101600	-1.17463700	-0.72601400	C	-1.49081300	-1.17472300	-0.72606400
C	-4.76798900	-2.50515800	1.40730600	C	-4.76780600	-2.50494500	1.40724800
C	-4.76798900	-2.50515800	-1.40730600	C	-4.76780600	-2.50494500	-1.40724800
C	-5.94997900	-2.08930100	-0.72403300	C	-5.94998700	-2.08939500	-0.72400200
C	-5.94997900	-2.08930100	0.72403300	C	-5.94998700	-2.08939500	0.72400200
C	-6.78548900	-1.19565900	1.40776600	C	-6.78534500	-1.19563600	1.40762100
H	-6.73075400	-1.16645800	2.49331800	H	-6.73066300	-1.16640100	2.49315900
C	-6.78548900	-1.19565900	-1.40776600	C	-6.78534500	-1.19563600	-1.40762100
H	-4.75781700	-2.44636400	2.49331400	H	-4.75781300	-2.44611200	2.49327800
H	-2.44873300	-1.85434000	2.49218900	H	-2.44850000	-1.85478100	2.49208200
H	-2.44873300	-1.85434000	-2.49218900	H	-2.44850000	-1.85478100	-2.49208200
H	-4.75781700	-2.44636400	-2.49331400	H	-4.75781300	-2.44611200	-2.49327800
H	-6.73075400	-1.16645800	-2.49331800	H	-6.73066300	-1.16640100	-2.49315900
C	-7.10790000	0.00000000	0.72405100	C	-7.10793200	0.00000000	0.72399200

C	-6.78548900	1.19565900	1.40776600	C	-6.78534500	1.19563600	1.40762100
C	-6.78548900	1.19565900	-1.40776600	C	-6.78534500	1.19563600	-1.40762100
C	-7.10790000	0.00000000	-0.72405100	C	-7.10793200	0.00000000	-0.72399200
H	-6.73075400	1.16645800	2.49331800	H	-6.73066300	1.16640100	2.49315900
H	-6.73075400	1.16645800	-2.49331800	H	-6.73066300	1.16640100	-2.49315900
C	1.49101600	1.17463700	0.72601400	C	1.49081300	1.17472300	0.72606400
C	2.40695100	1.91592900	1.40654100	C	2.40647900	1.91629800	1.40643000
C	3.56478900	2.44810100	0.72485900	C	3.56457900	2.44818600	0.72482700
C	3.56478900	2.44810100	-0.72485900	C	3.56457900	2.44818600	-0.72482700
C	2.40695100	1.91592900	-1.40654100	C	2.40647900	1.91629800	-1.40643000
C	1.49101600	1.17463700	-0.72601400	C	1.49081300	1.17472300	-0.72606400
C	4.76798900	2.50515800	1.40730600	C	4.76780600	2.50494500	1.40724800
C	4.76798900	2.50515800	-1.40730600	C	4.76780600	2.50494500	-1.40724800
C	5.94997900	2.08930100	-0.72403300	C	5.94998700	2.08939500	-0.72400200
C	5.94997900	2.08930100	0.72403300	C	5.94998700	2.08939500	0.72400200
C	6.78548900	1.19565900	1.40776600	C	6.78534500	1.19563600	1.40762100
H	6.73075400	1.16645800	2.49331800	H	6.73066300	1.16640100	2.49315900
C	6.78548900	1.19565900	-1.40776600	C	6.78534500	1.19563600	-1.40762100
H	4.75781700	2.44636400	2.49331400	H	4.75781300	2.44611200	2.49327800
H	2.44873300	1.85434000	2.49218900	H	2.44850000	1.85478100	2.49208200
H	2.44873300	1.85434000	-2.49218900	H	2.44850000	1.85478100	-2.49208200
H	4.75781700	2.44636400	-2.49331400	H	4.75781300	2.44611200	-2.49327800
H	6.73075400	1.16645800	-2.49331800	H	6.73066300	1.16640100	-2.49315900
C	7.10790000	0.00000000	0.72405100	C	7.10793200	0.00000000	0.72399200
C	6.78548900	-1.19565900	1.40776600	C	6.78534500	-1.19563600	1.40762100
C	5.94997900	-2.08930100	0.72403300	C	5.94998700	-2.08939500	0.72400200
C	5.94997900	-2.08930100	-0.72403300	C	5.94998700	-2.08939500	-0.72400200
C	6.78548900	-1.19565900	-1.40776600	C	6.78534500	-1.19563600	-1.40762100
C	7.10790000	0.00000000	-0.72405100	C	7.10793200	0.00000000	-0.72399200
C	4.76798900	-2.50515800	1.40730600	C	4.76780600	-2.50494500	1.40724800
C	4.76798900	-2.50515800	-1.40730600	C	4.76780600	-2.50494500	-1.40724800
C	3.56478900	-2.44810100	-0.72485900	C	3.56457900	-2.44818600	-0.72482700
C	3.56478900	-2.44810100	0.72485900	C	3.56457900	-2.44818600	0.72482700
C	2.40695100	-1.91592900	1.40654100	C	2.40647900	-1.91629800	1.40643000
H	2.44873300	-1.85434000	2.49218900	H	2.44850000	-1.85478100	2.49208200
C	2.40695100	-1.91592900	-1.40654100	C	2.40647900	-1.91629800	-1.40643000
H	4.75781700	-2.44636400	2.49331400	H	4.75781300	-2.44611200	2.49327800
H	6.73075400	-1.16645800	2.49331800	H	6.73066300	-1.16640100	2.49315900
H	6.73075400	-1.16645800	-2.49331800	H	6.73066300	-1.16640100	-2.49315900
H	4.75781700	-2.44636400	-2.49331400	H	4.75781300	-2.44611200	-2.49327800
H	2.44873300	-1.85434000	-2.49218900	H	2.44850000	-1.85478100	-2.49208200
C	1.49101600	-1.17463700	0.72601400	C	1.49081300	-1.17472300	0.72606400
C	0.78116500	0.00000000	1.39617800	C	0.78102800	0.00000000	1.39614800
C	0.78116500	0.00000000	-1.39617800	C	0.78102800	0.00000000	-1.39614800
C	1.49101600	-1.17463700	-0.72601400	C	1.49081300	-1.17472300	-0.72606400
H	1.08510300	0.00000000	2.44668500	H	1.08480100	0.00000000	2.44669700
H	1.08510300	0.00000000	-2.44668500	H	1.08480100	0.00000000	-2.44669700
**Dcyc8_R**				**Dcyc8_U**			
C	-3.43783200	2.62944800	0.72544700	C	-3.43686800	2.62575000	0.72148300
C	-4.61163500	2.88201000	1.40730200	C	-4.62220000	2.87550900	1.40443100
C	-5.85940700	2.75380600	0.72544200	C	-5.85904800	2.75190200	0.72339100
C	-5.85940700	2.75380600	-0.72544200	C	-5.85904800	2.75190200	-0.72339100
C	-4.61163500	2.88201000	-1.40730200	C	-4.62220000	2.87550900	-1.40443100
C	-3.43783200	2.62944800	-0.72544700	C	-3.43686800	2.62575000	-0.72148300
C	-6.92619600	2.16919800	1.40859200	C	-6.94164200	2.16908200	1.40816700
C	-6.92619600	2.16919800	-1.40859200	C	-6.94164200	2.16908200	-1.40816700
C	-7.70217800	1.20097600	-0.72519800	C	-7.70648100	1.20147000	-0.72654900
C	-7.70217800	1.20097600	0.72519800	C	-7.70648100	1.20147000	0.72654900

C	-7.96574700	0.00000000	1.40862200	C	-7.99197900	0.00000000	1.40991000
H	-7.90857900	0.00000000	2.49458600	H	-7.94994200	0.00000000	2.49637100
C	-7.96574700	0.00000000	-1.40862200	C	-7.99197900	0.00000000	-1.40991000
H	-6.88919000	2.12696100	2.49470900	H	-6.90835000	2.13261800	2.49450300
H	-4.61317700	2.83190800	2.49400100	H	-4.62367900	2.82298800	2.49099500
H	-4.61317700	2.83190800	-2.49400100	H	-4.62367900	2.82298800	-2.49099500
H	-6.88919000	2.12696100	-2.49470900	H	-6.90835000	2.13261800	-2.49450300
H	-7.90857900	0.00000000	-2.49458600	H	-7.94994200	0.00000000	-2.49637100
C	-7.70217800	-1.20097600	0.72519800	C	-7.70648100	-1.20147000	0.72654900
C	-6.92619600	-2.16919800	1.40859200	C	-6.94164200	-2.16908200	1.40816700
C	-5.85940700	-2.75380600	0.72544200	C	-5.85904800	-2.75190200	0.72339100
C	-5.85940700	-2.75380600	-0.72544200	C	-5.85904800	-2.75190200	-0.72339100
C	-6.92619600	-2.16919800	-1.40859200	C	-6.94164200	-2.16908200	-1.40816700
C	-7.70217800	-1.20097600	-0.72519800	C	-7.70648100	-1.20147000	-0.72654900
C	-4.61163500	-2.88201000	1.40730200	C	-4.62220000	-2.87550900	1.40443100
C	-4.61163500	-2.88201000	-1.40730200	C	-4.62220000	-2.87550900	-1.40443100
C	-3.43783200	-2.62944800	-0.72544700	C	-3.43686800	-2.62575000	-0.72148300
C	-3.43783200	-2.62944800	0.72544700	C	-3.43686800	-2.62575000	0.72148300
C	-2.34532400	-1.97328700	1.40761700	C	-2.35000800	-1.97333200	1.40425300
H	-2.39004900	-1.91862000	2.49356600	H	-2.39520500	-1.91869300	2.49019200
C	-2.34532400	-1.97328700	-1.40761700	C	-2.35000800	-1.97333200	-1.40425300
H	-4.61317700	-2.83190800	2.49400100	H	-4.62367900	-2.82298800	2.49099500
H	-6.88919000	-2.12696100	2.49470900	H	-6.90835000	-2.13261800	2.49450300
H	-6.88919000	-2.12696100	-2.49470900	H	-6.90835000	-2.13261800	-2.49450300
H	-4.61317700	-2.83190800	-2.49400100	H	-4.62367900	-2.82298800	-2.49099500
H	-2.39004900	-1.91862000	-2.49356600	H	-2.39520500	-1.91869300	-2.49019200
C	-1.47621100	-1.18177400	0.72710800	C	-1.47437300	-1.18230200	0.72345400
C	-1.47621100	-1.18177400	-0.72710800	C	-1.47437300	-1.18230200	-0.72345400
H	-1.08806200	0.00000000	2.44690000	H	-1.08925800	0.00000000	2.44495300
C	-0.78330300	0.00000000	1.39649600	C	-0.78363800	0.00000000	1.39478500
C	-0.78330300	0.00000000	-1.39649600	C	-0.78363800	0.00000000	-1.39478500
H	-1.08806200	0.00000000	-2.44690000	H	-1.08925800	0.00000000	-2.44495300
C	-1.47621100	1.18177400	0.72710800	C	-1.47437300	1.18230200	0.72345400
C	-2.34532400	1.97328700	1.40761700	C	-2.35000800	1.97333200	1.40425300
C	-2.34532400	1.97328700	-1.40761700	C	-2.35000800	1.97333200	-1.40425300
C	-1.47621100	1.18177400	-0.72710800	C	-1.47437300	1.18230200	-0.72345400
H	-2.39004900	1.91862000	2.49356600	H	-2.39520500	1.91869300	2.49019200
H	-2.39004900	1.91862000	-2.49356600	H	-2.39520500	1.91869300	-2.49019200
C	7.70217800	1.20097600	0.72519800	C	7.70648100	1.20147000	0.72654900
C	6.92619600	2.16919800	1.40859200	C	6.94164200	2.16908200	1.40816700
C	5.85940700	2.75380600	0.72544200	C	5.85904800	2.75190200	0.72339100
C	5.85940700	2.75380600	-0.72544200	C	5.85904800	2.75190200	-0.72339100
C	6.92619600	2.16919800	-1.40859200	C	6.94164200	2.16908200	-1.40816700
C	7.70217800	1.20097600	-0.72519800	C	7.70648100	1.20147000	-0.72654900
C	4.61163500	2.88201000	1.40730200	C	4.62220000	2.87550900	1.40443100
C	4.61163500	2.88201000	-1.40730200	C	4.62220000	2.87550900	-1.40443100
C	3.43783200	2.62944800	-0.72544700	C	3.43686800	2.62575000	-0.72148300
C	3.43783200	2.62944800	0.72544700	C	3.43686800	2.62575000	0.72148300
C	2.34532400	1.97328700	1.40761700	C	2.35000800	1.97333200	1.40425300
H	2.39004900	1.91862000	2.49356600	H	2.39520500	1.91869300	2.49019200
C	2.34532400	1.97328700	-1.40761700	C	2.35000800	1.97333200	-1.40425300
H	4.61317700	2.83190800	2.49400100	H	4.62367900	2.82298800	2.49099500
H	6.88919000	2.12696100	2.49470900	H	6.90835000	2.13261800	2.49450300
H	6.88919000	2.12696100	-2.49470900	H	6.90835000	2.13261800	-2.49450300
H	4.61317700	2.83190800	-2.49400100	H	4.62367900	2.82298800	-2.49099500
H	2.39004900	1.91862000	-2.49356600	H	2.39520500	1.91869300	-2.49019200
C	1.47621100	1.18177400	0.72710800	C	1.47437300	1.18230200	0.72345400
C	0.78330300	0.00000000	1.39649600	C	0.78363800	0.00000000	1.39478500
C	1.47621100	-1.18177400	0.72710800	C	1.47437300	-1.18230200	0.72345400
C	1.47621100	-1.18177400	-0.72710800	C	1.47437300	-1.18230200	-0.72345400

C	0.78330300	0.00000000	-1.39649600	C	0.78363800	0.00000000	-1.39478500
C	1.47621100	1.18177400	-0.72710800	C	1.47437300	1.18230200	-0.72345400
C	2.34532400	-1.97328700	1.40761700	C	2.35000800	-1.97333200	1.40425300
C	2.34532400	-1.97328700	-1.40761700	C	2.35000800	-1.97333200	-1.40425300
C	3.43783200	-2.62944800	-0.72544700	C	3.43686800	-2.62575000	-0.72148300
C	3.43783200	-2.62944800	0.72544700	C	3.43686800	-2.62575000	0.72148300
C	4.61163500	-2.88201000	1.40730200	C	4.62220000	-2.87550900	1.40443100
H	4.61317700	-2.83190800	2.49400100	H	4.62367900	-2.82298800	2.49099500
C	4.61163500	-2.88201000	-1.40730200	C	4.62220000	-2.87550900	-1.40443100
H	2.39004900	-1.91862000	2.49356600	H	2.39520500	-1.91869300	2.49019200
H	1.08806200	0.00000000	2.44690000	H	1.08925800	0.00000000	2.44495300
H	1.08806200	0.00000000	-2.44690000	H	1.08925800	0.00000000	-2.44495300
H	2.39004900	-1.91862000	-2.49356600	H	2.39520500	-1.91869300	-2.49019200
H	4.61317700	-2.83190800	-2.49400100	H	4.62367900	-2.82298800	-2.49099500
C	5.85940700	-2.75380600	0.72544200	C	5.85904800	-2.75190200	0.72339100
C	5.85940700	-2.75380600	-0.72544200	C	5.85904800	-2.75190200	-0.72339100
H	6.88919000	-2.12696100	2.49470900	H	6.90835000	-2.13261800	2.49450300
C	6.92619600	-2.16919800	1.40859200	C	6.94164200	-2.16908200	1.40816700
C	6.92619600	-2.16919800	-1.40859200	C	6.94164200	-2.16908200	-1.40816700
H	6.88919000	-2.12696100	-2.49470900	H	6.90835000	-2.13261800	-2.49450300
C	7.70217800	-1.20097600	0.72519800	C	7.70648100	-1.20147000	0.72654900
C	7.96574700	0.00000000	1.40862200	C	7.99197900	0.00000000	1.40991000
C	7.96574700	0.00000000	-1.40862200	C	7.99197900	0.00000000	-1.40991000
C	7.70217800	-1.20097600	-0.72519800	C	7.70648100	-1.20147000	-0.72654900
H	7.90857900	0.00000000	2.49458600	H	7.94994200	0.00000000	2.49637100
H	7.90857900	0.00000000	-2.49458600	H	7.94994200	0.00000000	-2.49637100
**Dcyc9_R**				**Dcyc9_U**			
C	-5.71699300	3.23375300	0.72660800	C	-5.71415900	3.23112000	0.72238000
C	-4.46561800	3.14680000	1.40787100	C	-4.48178100	3.13919800	1.40295200
C	-3.33502000	2.74996400	0.72644600	C	-3.33199100	2.74801400	0.71989400
C	-3.33502000	2.74996400	-0.72644600	C	-3.33199100	2.74801400	-0.71989400
C	-4.46561800	3.14680000	-1.40787100	C	-4.48178100	3.13919800	-1.40295200
C	-5.71699300	3.23375300	-0.72660800	C	-5.71415900	3.23112000	-0.72238000
C	-2.29246500	2.01796400	1.40811200	C	-2.30058300	2.01739000	1.40346000
C	-2.29246500	2.01796400	-1.40811200	C	-2.30058300	2.01739000	-1.40346000
C	-1.46418400	1.18608400	-0.72733000	C	-1.46215400	1.18704000	-0.72217200
C	-1.46418400	1.18608400	0.72733000	C	-1.46215400	1.18704000	0.72217200
C	-0.78471000	0.00000000	1.39788600	C	-0.78501900	0.00000000	1.39507200
H	-1.09147600	0.00000000	2.44765600	H	-1.09272500	0.00000000	2.44465000
C	-0.78471000	0.00000000	-1.39788600	C	-0.78501900	0.00000000	-1.39507200
H	-2.33389500	1.97359800	2.49458100	H	-2.34461100	1.97059100	2.48978600
H	-4.47344700	3.10385300	2.49481600	H	-4.48973300	3.09198100	2.48979000
H	-4.47344700	3.10385300	-2.49481600	H	-4.48973300	3.09198100	-2.48979000
H	-2.33389500	1.97359800	-2.49458100	H	-2.34461100	1.97059100	-2.48978600
H	-1.09147600	0.00000000	-2.44765600	H	-1.09272500	0.00000000	-2.44465000
C	-1.46418400	-1.18608400	0.72733000	C	-1.46215400	-1.18704000	0.72217200
C	-2.29246500	-2.01796400	1.40811200	C	-2.30058300	-2.01739000	1.40346000
C	-3.33502000	-2.74996400	0.72644600	C	-3.33199100	-2.74801400	0.71989400
C	-3.33502000	-2.74996400	-0.72644600	C	-3.33199100	-2.74801400	-0.71989400
C	-2.29246500	-2.01796400	-1.40811200	C	-2.30058300	-2.01739000	-1.40346000
C	-1.46418400	-1.18608400	-0.72733000	C	-1.46215400	-1.18704000	-0.72217200
C	-4.46561800	-3.14680000	1.40787100	C	-4.48178100	-3.13919800	1.40295200
C	-4.46561800	-3.14680000	-1.40787100	C	-4.48178100	-3.13919800	-1.40295200
C	-5.71699300	-3.23375300	-0.72660800	C	-5.71415900	-3.23112000	-0.72238000
C	-5.71699300	-3.23375300	0.72660800	C	-5.71415900	-3.23112000	0.72238000
C	-6.88924400	-2.92322500	1.40921500	C	-6.90910800	-2.91745300	1.40710200
H	-6.86825100	-2.87999300	2.49573100	H	-6.88895800	-2.87942000	2.49378000
C	-6.88924400	-2.92322500	-1.40921500	C	-6.90910800	-2.91745300	-1.40710200

H	-4.47344700	-3.10385300	2.49481600	H	-4.48973300	-3.09198100	2.48979000
H	-2.33389500	-1.97359800	2.49458100	H	-2.34461100	-1.97059100	2.48978600
H	-2.33389500	-1.97359800	-2.49458100	H	-2.34461100	-1.97059100	-2.48978600
H	-4.47344700	-3.10385300	-2.49481600	H	-4.48973300	-3.09198100	-2.48979000
H	-6.86825100	-2.87999300	-2.49573100	H	-6.88895800	-2.87942000	-2.49378000
C	-7.91600600	-2.22412900	0.72696400	C	-7.91998200	-2.22641100	0.72720500
C	-8.59687000	-1.20739300	1.40981400	C	-8.63210800	-1.21053800	1.41058800
C	-8.84590100	0.00000000	0.72690400	C	-8.85501700	0.00000000	0.72974100
C	-8.84590100	0.00000000	-0.72690400	C	-8.85501700	0.00000000	-0.72974100
C	-8.59687000	-1.20739300	-1.40981400	C	-8.63210800	-1.21053800	-1.41058800
C	-7.91600600	-2.22412900	-0.72696400	C	-7.91998200	-2.22641100	-0.72720500
C	-8.59687000	1.20739300	1.40981400	C	-8.63210800	1.21053800	1.41058800
C	-8.59687000	1.20739300	-1.40981400	C	-8.63210800	1.21053800	-1.41058800
C	-7.91600600	2.22412900	-0.72696400	C	-7.91998200	2.22641100	-0.72720500
C	-7.91600600	2.22412900	0.72696400	C	-7.91998200	2.22641100	0.72720500
C	-6.88924400	2.92322500	1.40921500	C	-6.90910800	2.91745300	1.40710200
H	-6.86825100	2.87999300	2.49573100	H	-6.88895800	2.87942000	2.49378000
C	-6.88924400	2.92322500	-1.40921500	C	-6.90910800	2.91745300	-1.40710200
H	-8.54927700	1.18816800	2.49596700	H	-8.60373300	1.19997800	2.49737600
H	-8.54927700	-1.18816800	2.49596700	H	-8.60373300	-1.19997800	2.49737600
H	-8.54927700	-1.18816800	-2.49596700	H	-8.60373300	-1.19997800	-2.49737600
H	-8.54927700	1.18816800	-2.49596700	H	-8.60373300	1.19997800	-2.49737600
H	-6.86825100	2.87999300	-2.49573100	H	-6.88895800	2.87942000	-2.49378000
C	3.33502000	2.74996400	0.72644600	C	3.33199100	2.74801400	0.71989400
C	4.46561800	3.14680000	1.40787100	C	4.48178100	3.13919800	1.40295200
C	5.71699300	3.23375300	0.72660800	C	5.71415900	3.23112000	0.72238000
C	5.71699300	3.23375300	-0.72660800	C	5.71415900	3.23112000	-0.72238000
C	4.46561800	3.14680000	-1.40787100	C	4.48178100	3.13919800	-1.40295200
C	3.33502000	2.74996400	-0.72644600	C	3.33199100	2.74801400	-0.71989400
C	6.88924400	2.92322500	1.40921500	C	6.90910800	2.91745300	1.40710200
C	6.88924400	2.92322500	-1.40921500	C	6.90910800	2.91745300	-1.40710200
C	7.91600600	2.22412900	-0.72696400	C	7.91998200	2.22641100	-0.72720500
C	7.91600600	2.22412900	0.72696400	C	7.91998200	2.22641100	0.72720500
C	8.59687000	1.20739300	1.40981400	C	8.63210800	1.21053800	1.41058800
H	8.54927700	1.18816800	2.49596700	H	8.60373300	1.19997800	2.49737600
C	8.59687000	1.20739300	-1.40981400	C	8.63210800	1.21053800	-1.41058800
H	6.86825100	2.87999300	2.49573100	H	6.88895800	2.87942000	2.49378000
H	4.47344700	3.10385300	2.49481600	H	4.48973300	3.09198100	2.48979000
H	4.47344700	3.10385300	-2.49481600	H	4.48973300	3.09198100	-2.48979000
H	6.86825100	2.87999300	-2.49573100	H	6.88895800	2.87942000	-2.49378000
H	8.54927700	1.18816800	-2.49596700	H	8.60373300	1.19997800	-2.49737600
C	8.84590100	0.00000000	0.72690400	C	8.85501700	0.00000000	0.72974100
C	8.59687000	-1.20739300	1.40981400	C	8.63210800	-1.21053800	1.41058800
C	7.91600600	-2.22412900	0.72696400	C	7.91998200	-2.22641100	0.72720500
C	7.91600600	-2.22412900	-0.72696400	C	7.91998200	-2.22641100	-0.72720500
C	8.59687000	-1.20739300	-1.40981400	C	8.63210800	-1.21053800	-1.41058800
C	8.84590100	0.00000000	-0.72690400	C	8.85501700	0.00000000	-0.72974100
C	6.88924400	-2.92322500	1.40921500	C	6.90910800	-2.91745300	1.40710200
C	6.88924400	-2.92322500	-1.40921500	C	6.90910800	-2.91745300	-1.40710200
C	5.71699300	-3.23375300	-0.72660800	C	5.71415900	-3.23112000	-0.72238000
C	5.71699300	-3.23375300	0.72660800	C	5.71415900	-3.23112000	0.72238000
C	4.46561800	-3.14680000	1.40787100	C	4.48178100	-3.13919800	1.40295200
H	4.47344700	-3.10385300	2.49481600	H	4.48973300	-3.09198100	2.48979000
C	4.46561800	-3.14680000	-1.40787100	C	4.48178100	-3.13919800	-1.40295200
H	6.86825100	-2.87999300	2.49573100	H	6.88895800	-2.87942000	2.49378000
H	8.54927700	-1.18816800	2.49596700	H	8.60373300	-1.19997800	2.49737600
H	8.54927700	-1.18816800	-2.49596700	H	8.60373300	-1.19997800	-2.49737600
H	6.86825100	-2.87999300	-2.49573100	H	6.88895800	-2.87942000	-2.49378000
H	4.47344700	-3.10385300	-2.49481600	H	4.48973300	-3.09198100	-2.48979000
C	3.33502000	-2.74996400	0.72644600	C	3.33199100	-2.74801400	0.71989400

C	2.29246500	-2.01796400	1.40811200	C	2.30058300	-2.01739000	1.40346000
C	1.46418400	-1.18608400	0.72733000	C	1.46215400	-1.18704000	0.72217200
C	1.46418400	-1.18608400	-0.72733000	C	1.46215400	-1.18704000	-0.72217200
C	2.29246500	-2.01796400	-1.40811200	C	2.30058300	-2.01739000	-1.40346000
C	3.33502000	-2.74996400	-0.72644600	C	3.33199100	-2.74801400	-0.71989400
C	0.78471000	0.00000000	1.39788600	C	0.78501900	0.00000000	1.39507200
C	0.78471000	0.00000000	-1.39788600	C	0.78501900	0.00000000	-1.39507200
C	1.46418400	1.18608400	-0.72733000	C	1.46215400	1.18704000	-0.72217200
C	1.46418400	1.18608400	0.72733000	C	1.46215400	1.18704000	0.72217200
C	2.29246500	2.01796400	1.40811200	C	2.30058300	2.01739000	1.40346000
H	2.33389500	1.97359800	2.49458100	H	2.34461100	1.97059100	2.48978600
C	2.29246500	2.01796400	-1.40811200	C	2.30058300	2.01739000	-1.40346000
H	1.09147600	0.00000000	2.44765600	H	1.09272500	0.00000000	2.44465000
H	2.33389500	-1.97359800	2.49458100	H	2.34461100	-1.97059100	2.48978600
H	2.33389500	-1.97359800	-2.49458100	H	2.34461100	-1.97059100	-2.48978600
H	1.09147600	0.00000000	-2.44765600	H	1.09272500	0.00000000	-2.44465000
H	2.33389500	1.97359800	-2.49458100	H	2.34461100	1.97059100	-2.48978600
**Dcyc10_R**				**Dcyc10_U**			
C	1.45567800	1.19036500	0.72757000	C	1.45349500	1.19059100	0.72193300
C	1.45567800	1.19036500	-0.72757000	C	1.45349500	1.19059100	-0.72193300
C	0.78630100	0.00000000	1.39785600	C	0.78660000	0.00000000	1.39550500
C	0.78630100	0.00000000	-1.39785600	C	0.78660000	0.00000000	-1.39550500
H	1.09327200	0.00000000	2.44758800	H	1.09506800	0.00000000	2.44487000
H	1.09327200	0.00000000	-2.44758800	H	1.09506800	0.00000000	-2.44487000
C	3.25306500	-2.84022400	0.72723300	C	3.25019200	-2.83544400	0.71995500
C	3.25306500	-2.84022400	-0.72723300	C	3.25019200	-2.83544400	-0.71995500
C	8.87270500	-2.26797300	1.41004800	C	8.90428200	-2.26904000	1.40953000
C	7.93696600	-3.05032600	0.72762800	C	7.93790800	-3.05125900	0.72653200
C	6.77614100	-3.49552300	1.40936600	C	6.79780300	-3.48769400	1.40616500
C	7.93696600	-3.05032600	-0.72762800	C	7.93790800	-3.05125900	-0.72653200
C	6.77614100	-3.49552300	-1.40936600	C	6.79780300	-3.48769400	-1.40616500
C	5.57013100	-3.59121500	-0.72720200	C	5.56580900	-3.58491000	-0.72218400
C	5.57013100	-3.59121500	0.72720200	C	5.56580900	-3.58491000	0.72218400
C	9.50785100	-1.20865600	0.72769400	C	9.51998000	-1.21153400	0.72962600
C	8.87270500	-2.26797300	-1.41004800	C	8.90428200	-2.26904000	-1.40953000
H	6.76560100	-3.45485300	-2.49620300	H	6.78714400	-3.44980800	-2.49310300
C	4.34038100	-3.33984500	-1.40794700	C	4.35770800	-3.33281700	-1.40261400
C	4.34038100	-3.33984500	1.40794700	C	4.35770800	-3.33281700	1.40261400
H	4.35147900	-3.30444600	-2.49526400	H	4.36953400	-3.29219800	-2.48972100
H	4.35147900	-3.30444600	2.49526400	H	4.36953400	-3.29219800	2.48972100
C	9.50785100	-1.20865600	-0.72769400	C	9.51998000	-1.21153400	-0.72962600
H	8.83735600	-2.23863600	2.49660800	H	8.88122700	-2.25107500	2.49651900
H	6.76560100	-3.45485300	2.49620300	H	6.78714400	-3.44980800	2.49310300
H	8.83735600	-2.23863600	-2.49660800	H	8.88122700	-2.25107500	-2.49651900
C	2.25542600	-2.04797900	1.40861800	C	2.26161400	-2.04962500	1.40355000
C	1.45567800	-1.19036500	0.72757000	C	1.45349500	-1.19059100	0.72193300
C	1.45567800	-1.19036500	-0.72757000	C	1.45349500	-1.19059100	-0.72193300
C	2.25542600	-2.04797900	-1.40861800	C	2.26161400	-2.04962500	-1.40355000
H	2.29692800	-2.00765400	2.49524600	H	2.30331500	-2.00953000	2.49021500
H	2.29692800	-2.00765400	-2.49524600	H	2.30331500	-2.00953000	-2.49021500
C	9.72336100	0.00000000	1.41040700	C	9.76584300	0.00000000	1.41056900
C	9.72336100	0.00000000	-1.41040700	C	9.76584300	0.00000000	-1.41056900
H	9.67544900	0.00000000	2.49684000	H	9.74325900	0.00000000	2.49758600
H	9.67544900	0.00000000	-2.49684000	H	9.74325900	0.00000000	-2.49758600
C	7.93696600	3.05032600	0.72762800	C	7.93790800	3.05125900	0.72653200
C	7.93696600	3.05032600	-0.72762800	C	7.93790800	3.05125900	-0.72653200
C	2.25542600	2.04797900	1.40861800	C	2.26161400	2.04962500	1.40355000
C	3.25306500	2.84022400	0.72723300	C	3.25019200	2.83544400	0.71995500

C	4.34038100	3.33984500	1.40794700	C	4.35770800	3.33281700	1.40261400
C	3.25306500	2.84022400	-0.72723300	C	3.25019200	2.83544400	-0.71995500
C	4.34038100	3.33984500	-1.40794700	C	4.35770800	3.33281700	-1.40261400
C	5.57013100	3.59121500	-0.72720200	C	5.56580900	3.58491000	-0.72218400
C	5.57013100	3.59121500	0.72720200	C	5.56580900	3.58491000	0.72218400
C	2.25542600	2.04797900	-1.40861800	C	2.26161400	2.04962500	-1.40355000
H	4.35147900	3.30444600	-2.49526400	H	4.36953400	3.29219800	-2.48972100
C	6.77614100	3.49552300	-1.40936600	C	6.79780300	3.48769400	-1.40616500
C	6.77614100	3.49552300	1.40936600	C	6.79780300	3.48769400	1.40616500
H	6.76560100	3.45485300	-2.49620300	H	6.78714400	3.44980800	-2.49310300
H	6.76560100	3.45485300	2.49620300	H	6.78714400	3.44980800	2.49310300
H	2.29692800	2.00765400	2.49524600	H	2.30331500	2.00953000	2.49021500
H	4.35147900	3.30444600	2.49526400	H	4.36953400	3.29219800	2.48972100
H	2.29692800	2.00765400	-2.49524600	H	2.30331500	2.00953000	-2.49021500
C	8.87270500	2.26797300	1.41004800	C	8.90428200	2.26904000	1.40953000
C	9.50785100	1.20865600	0.72769400	C	9.51998000	1.21153400	0.72962600
C	9.50785100	1.20865600	-0.72769400	C	9.51998000	1.21153400	-0.72962600
C	8.87270500	2.26797300	-1.41004800	C	8.90428200	2.26904000	-1.40953000
H	8.83735600	2.23863600	2.49660800	H	8.88122700	2.25107500	2.49651900
H	8.83735600	2.23863600	-2.49660800	H	8.88122700	2.25107500	-2.49651800
C	-9.50785100	1.20865600	0.72769400	C	-9.51998000	1.21153400	0.72962600
C	-9.50785100	1.20865600	-0.72769400	C	-9.51998000	1.21153400	-0.72962600
C	-9.72336100	0.00000000	1.41040700	C	-9.76584300	0.00000000	1.41056900
C	-9.72336100	0.00000000	-1.41040700	C	-9.76584300	0.00000000	-1.41056900
H	-9.67544900	0.00000000	2.49684000	H	-9.74325900	0.00000000	2.49758600
H	-9.67544900	0.00000000	-2.49684000	H	-9.74325900	0.00000000	-2.49758600
C	-7.93696600	-3.05032600	0.72762800	C	-7.93790800	-3.05125900	0.72653200
C	-7.93696600	-3.05032600	-0.72762800	C	-7.93790800	-3.05125900	-0.72653200
C	-2.25542600	-2.04797900	1.40861800	C	-2.26161400	-2.04962500	1.40355000
C	-3.25306500	-2.84022400	0.72723300	C	-3.25019200	-2.83544400	0.71995500
C	-4.34038100	-3.33984500	1.40794700	C	-4.35770800	-3.33281700	1.40261400
C	-3.25306500	-2.84022400	-0.72723300	C	-3.25019200	-2.83544400	-0.71995500
C	-4.34038100	-3.33984500	-1.40794700	C	-4.35770800	-3.33281700	-1.40261400
C	-5.57013100	-3.59121500	-0.72720200	C	-5.56580900	-3.58491000	-0.72218400
C	-5.57013100	-3.59121500	0.72720200	C	-5.56580900	-3.58491000	0.72218400
C	-1.45567800	-1.19036500	0.72757000	C	-1.45349500	-1.19059100	0.72193300
C	-2.25542600	-2.04797900	-1.40861800	C	-2.26161400	-2.04962500	-1.40355000
H	-4.35147900	-3.30444600	-2.49526400	H	-4.36953400	-3.29219800	-2.48972100
C	-6.77614100	-3.49552300	-1.40936600	C	-6.79780300	-3.48769400	-1.40616500
C	-6.77614100	-3.49552300	1.40936600	C	-6.79780300	-3.48769400	1.40616500
H	-6.76560100	-3.45485300	-2.49620300	H	-6.78714400	-3.44980800	-2.49310300
H	-6.76560100	-3.45485300	2.49620300	H	-6.78714400	-3.44980800	2.49310300
C	-1.45567800	-1.19036500	-0.72757000	C	-1.45349500	-1.19059100	-0.72193300
H	-2.29692800	-2.00765400	2.49524600	H	-2.30331500	-2.00953000	2.49021500
H	-4.35147900	-3.30444600	2.49526400	H	-4.36953400	-3.29219800	2.48972100
H	-2.29692800	-2.00765400	-2.49524600	H	-2.30331500	-2.00953000	-2.49021500
C	-8.87270500	-2.26797300	1.41004800	C	-8.90428200	-2.26904000	1.40953000
C	-9.50785100	-1.20865600	0.72769400	C	-9.51998000	-1.21153400	0.72962600
C	-9.50785100	-1.20865600	-0.72769400	C	-9.51998000	-1.21153400	-0.72962600
C	-8.87270500	-2.26797300	-1.41004800	C	-8.90428200	-2.26904000	-1.40953000
H	-8.83735600	-2.23863600	2.49660800	H	-8.88122700	-2.25107500	2.49651900
H	-8.83735600	-2.23863600	-2.49660800	H	-8.88122700	-2.25107500	-2.49651900
C	-0.78630100	0.00000000	1.39785600	C	-0.78660000	0.00000000	1.39550500
C	-0.78630100	0.00000000	-1.39785600	C	-0.78660000	0.00000000	-1.39550500
H	-1.09327200	0.00000000	2.44758800	H	-1.09506800	0.00000000	2.44487000
H	-1.09327200	0.00000000	-2.44758800	H	-1.09506800	0.00000000	-2.44487000
C	-3.25306500	2.84022400	0.72723300	C	-3.25019200	2.83544400	0.71995500
C	-3.25306500	2.84022400	-0.72723300	C	-3.25019200	2.83544400	-0.71995500
C	-8.87270500	2.26797300	1.41004800	C	-8.90428200	2.26904000	1.40953000
C	-7.93696600	3.05032600	0.72762800	C	-7.93790800	3.05125900	0.72653200

C	-6.77614100	3.49552300	1.40936600	C	-6.79780300	3.48769400	1.40616500
C	-7.93696600	3.05032600	-0.72762800	C	-7.93790800	3.05125900	-0.72653200
C	-6.77614100	3.49552300	-1.40936600	C	-6.79780300	3.48769400	-1.40616500
C	-5.57013100	3.59121500	-0.72720200	C	-5.56580900	3.58491000	-0.72218400
C	-5.57013100	3.59121500	0.72720200	C	-5.56580900	3.58491000	0.72218400
C	-8.87270500	2.26797300	-1.41004800	C	-8.90428200	2.26904000	-1.40953000
H	-6.76560100	3.45485300	-2.49620300	H	-6.78714400	3.44980800	-2.49310300
C	-4.34038100	3.33984500	-1.40794700	C	-4.35770800	3.33281700	-1.40261400
C	-4.34038100	3.33984500	1.40794700	C	-4.35770800	3.33281700	1.40261400
H	-4.35147900	3.30444600	-2.49526400	H	-4.36953400	3.29219800	-2.48972100
H	-4.35147900	3.30444600	2.49526400	H	-4.36953400	3.29219800	2.48972100
H	-8.83735600	2.23863600	2.49660800	H	-8.88122700	2.25107500	2.49651900
H	-6.76560100	3.45485300	2.49620300	H	-6.78714400	3.44980800	2.49310300
H	-8.83735600	2.23863600	-2.49660800	H	-8.88122700	2.25107500	-2.49651900
C	-2.25542600	2.04797900	1.40861800	C	-2.26161400	2.04962500	1.40355000
C	-1.45567800	1.19036500	0.72757000	C	-1.45349500	1.19059100	0.72193300
C	-1.45567800	1.19036500	-0.72757000	C	-1.45349500	1.19059100	-0.72193300
C	-2.25542600	2.04797900	-1.40861800	C	-2.26161400	2.04962500	-1.40355000
H	-2.29692800	2.00765400	2.49524600	H	-2.30331500	2.00953000	2.49021500
H	-2.29692800	2.00765400	-2.49524600	H	-2.30331500	2.00953000	-2.49021500

**Dcyc11_R**				**Dcyc11_U**			
C	-1.44801900	1.19329700	0.72778300	C	-1.44614800	1.19400300	0.72247100
C	-1.44801900	1.19329700	-0.72778300	C	-1.44614800	1.19400300	-0.72247100
C	-7.85160200	-3.70689200	0.72821500	C	-7.84990500	-3.70830400	0.72615100
C	-7.85160200	-3.70689200	-0.72821500	C	-7.84990500	-3.70830400	-0.72615100
C	-6.62999700	3.94027200	1.40940800	C	-6.65107600	3.93399700	1.40573200
C	-5.42391200	3.86572800	0.72794600	C	-5.41924500	3.86091300	0.72224100
C	-4.22655700	3.49013000	1.40834000	C	-4.24255100	3.48666700	1.40274000
C	-5.42391200	3.86572800	-0.72794600	C	-5.41924500	3.86091300	-0.72224100
C	-4.22655700	3.49013000	-1.40834000	C	-4.24255100	3.48666700	-1.40274000
C	-3.18070700	2.91117900	-0.72722100	C	-3.17720500	2.90862100	-0.72037700
C	-3.18070700	2.91117900	0.72722100	C	-3.17720500	2.90862100	0.72037700
C	-7.85160200	3.70689200	0.72821500	C	-7.84990500	3.70830400	0.72615100
C	-6.62999700	3.94027200	-1.40940800	C	-6.65107600	3.93399700	-1.40573200
H	-4.23892500	3.45922800	-2.49550000	H	-4.25625100	3.45188400	-2.49004700
C	-2.22070300	2.07436400	-1.40892800	C	-2.22713200	2.07594600	-1.40418500
C	-2.22070300	2.07436400	1.40892800	C	-2.22713200	2.07594600	1.40418500
H	-2.25920700	2.04002200	-2.49585000	H	-2.26756400	2.04031700	-2.49104400
H	-2.25920700	2.04002200	2.49585000	H	-2.26756400	2.04031700	2.49104400
C	-7.85160200	3.70689200	-0.72821500	C	-7.84990500	3.70830400	-0.72615100
H	-6.62661300	3.90385100	2.49648500	H	-6.64704300	3.89792800	2.49284100
H	-4.23892500	3.45922800	2.49550000	H	-4.25625100	3.45188400	2.49004700
H	-6.62661300	3.90385100	-2.49648500	H	-6.64704300	3.89792800	-2.49284100
C	-8.93568900	-3.15306700	1.41008400	C	-8.96258200	-3.15266800	1.40879900
C	-9.82597100	-2.29547500	0.72855000	C	-9.83473800	-2.30073500	0.72922900
C	-10.39862100	-1.21342300	1.41066900	C	-10.43534400	-1.21558700	1.41013000
C	-9.82597100	-2.29547500	-0.72855000	C	-9.83473800	-2.30073500	-0.72922900
C	-10.39862100	-1.21342300	-1.41066900	C	-10.43534400	-1.21558700	-1.41013000
C	-10.60069200	0.00000000	-0.72868300	C	-10.61598800	0.00000000	-0.73020400
C	-10.60069200	0.00000000	0.72868300	C	-10.61598800	0.00000000	0.73020400
C	-8.93568900	-3.15306700	-1.41008400	C	-8.96258200	-3.15266800	-1.40879900
C	-10.39862100	1.21342300	-1.41066900	C	-10.43534400	1.21558700	-1.41013000
C	-10.39862100	1.21342300	1.41066900	C	-10.43534400	1.21558700	1.41013000
H	-8.91154000	-3.12072200	2.49690500	H	-8.94465200	-3.12955000	2.49588700
H	-8.91154000	-3.12072200	-2.49690500	H	-8.94465200	-3.12955000	-2.49588700
C	-9.82597100	2.29547500	0.72855000	C	-9.83473800	2.30073500	0.72922900
C	-9.82597100	2.29547500	-0.72855000	C	-9.83473800	2.30073500	-0.72922900
C	-8.93568900	3.15306700	-1.41008400	C	-8.96258200	3.15266800	-1.40879900

C	-8.93568900	3.15306700	1.41008400	C	-8.96258200	3.15266800	1.40879900
H	-10.35806600	-1.20002600	-2.49737800	H	-10.41461600	-1.20893800	-2.49723400
H	-10.35806600	-1.20002600	2.49737800	H	-10.41461600	-1.20893800	2.49723400
H	-10.35806600	1.20002600	2.49737800	H	-10.41461600	1.20893800	2.49723400
H	-10.35806600	1.20002600	-2.49737800	H	-10.41461600	1.20893800	-2.49723400
H	-8.91154000	3.12072200	-2.49690500	H	-8.94465200	3.12955000	-2.49588700
H	-8.91154000	3.12072200	2.49690500	H	-8.94465200	3.12955000	2.49588700
C	-6.62999700	-3.94027200	-1.40940800	C	-6.65107600	-3.93399700	-1.40573200
C	-5.42391200	-3.86572800	-0.72794600	C	-5.41924500	-3.86091300	-0.72224100
C	-4.22655700	-3.49013000	-1.40834000	C	-4.24255100	-3.48666700	-1.40274000
C	-5.42391200	-3.86572800	0.72794600	C	-5.41924500	-3.86091300	0.72224100
C	-4.22655700	-3.49013000	1.40834000	C	-4.24255100	-3.48666700	1.40274000
C	-3.18070700	-2.91117900	0.72722100	C	-3.17720500	-2.90862100	0.72037700
C	-3.18070700	-2.91117900	-0.72722100	C	-3.17720500	-2.90862100	-0.72037700
C	-6.62999700	-3.94027200	1.40940800	C	-6.65107600	-3.93399700	1.40573200
C	-2.22070300	-2.07436400	1.40892800	C	-2.22713200	-2.07594600	1.40418500
C	-2.22070300	-2.07436400	-1.40892800	C	-2.22713200	-2.07594600	-1.40418500
H	-6.62661300	-3.90385100	-2.49648500	H	-6.64704300	-3.89792800	-2.49284100
H	-6.62661300	-3.90385100	2.49648500	H	-6.64704300	-3.89792800	2.49284100
C	-1.44801900	-1.19329700	-0.72778300	C	-1.44614800	-1.19400300	-0.72247100
C	-1.44801900	-1.19329700	0.72778300	C	-1.44614800	-1.19400300	0.72247100
C	-0.78729800	0.00000000	1.39872700	C	-0.78770900	0.00000000	1.39599500
C	-0.78729800	0.00000000	-1.39872700	C	-0.78770900	0.00000000	-1.39599500
H	-4.23892500	-3.45922800	2.49550000	H	-4.25625100	-3.45188400	2.49004700
H	-4.23892500	-3.45922800	-2.49550000	H	-4.25625100	-3.45188400	-2.49004700
H	-2.25920700	-2.04002200	-2.49585000	H	-2.26756400	-2.04031700	-2.49104400
H	-2.25920700	-2.04002200	2.49585000	H	-2.26756400	-2.04031700	2.49104400
H	-1.09517800	0.00000000	2.44821200	H	-1.09658200	0.00000000	2.44525200
H	-1.09517800	0.00000000	-2.44821200	H	-1.09658200	0.00000000	-2.44525200
C	10.60069200	0.00000000	0.72868300	C	10.61598800	0.00000000	0.73020400
C	10.60069200	0.00000000	-0.72868300	C	10.61598800	0.00000000	-0.73020400
C	3.18070700	-2.91117900	0.72722100	C	3.17720500	-2.90862100	0.72037700
C	3.18070700	-2.91117900	-0.72722100	C	3.17720500	-2.90862100	-0.72037700
C	6.62999700	3.94027200	1.40940800	C	6.65107600	3.93399700	1.40573200
C	7.85160200	3.70689200	0.72821500	C	7.84990500	3.70830400	0.72615100
C	8.93568900	3.15306700	1.41008400	C	8.96258200	3.15266800	1.40879900
C	7.85160200	3.70689200	-0.72821500	C	7.84990500	3.70830400	-0.72615100
C	8.93568900	3.15306700	-1.41008400	C	8.96258200	3.15266800	-1.40879900
C	9.82597100	2.29547500	-0.72855000	C	9.83473800	2.30073500	-0.72922900
C	9.82597100	2.29547500	0.72855000	C	9.83473800	2.30073500	0.72922900
C	5.42391200	3.86572800	0.72794600	C	5.41924500	3.86091300	0.72224100
C	6.62999700	3.94027200	-1.40940800	C	6.65107600	3.93399700	-1.40573200
H	8.91154000	3.12072200	-2.49690500	H	8.94465200	3.12955000	-2.49588700
C	10.39862100	1.21342300	-1.41066900	C	10.43534400	1.21558700	-1.41013000
C	10.39862100	1.21342300	1.41066900	C	10.43534400	1.21558700	1.41013000
H	10.35806600	1.20002600	-2.49737800	H	10.41461600	1.20893800	-2.49723400
H	10.35806600	1.20002600	2.49737800	H	10.41461600	1.20893800	2.49723400
C	5.42391200	3.86572800	-0.72794600	C	5.41924500	3.86091300	-0.72224100
H	6.62661300	3.90385100	2.49648500	H	6.64704300	3.89792800	2.49284100
H	8.91154000	3.12072200	2.49690500	H	8.94465200	3.12955000	2.49588700
H	6.62661300	3.90385100	-2.49648500	H	6.64704300	3.89792800	-2.49284100
C	2.22070300	-2.07436400	1.40892800	C	2.22713200	-2.07594600	1.40418500
C	1.44801900	-1.19329700	0.72778300	C	1.44614800	-1.19400300	0.72247100
C	0.78729800	0.00000000	1.39872700	C	0.78770900	0.00000000	1.39599500
C	1.44801900	-1.19329700	-0.72778300	C	1.44614800	-1.19400300	-0.72247100
C	0.78729800	0.00000000	-1.39872700	C	0.78770900	0.00000000	-1.39599500
C	1.44801900	1.19329700	-0.72778300	C	1.44614800	1.19400300	-0.72247100
C	1.44801900	1.19329700	0.72778300	C	1.44614800	1.19400300	0.72247100
C	2.22070300	-2.07436400	-1.40892800	C	2.22713200	-2.07594600	-1.40418500
C	2.22070300	2.07436400	-1.40892800	C	2.22713200	2.07594600	-1.40418500

C	2.22070300	2.07436400	1.40892800	C	2.22713200	2.07594600	1.40418500
H	2.25920700	-2.04002200	2.49585000	H	2.26756400	-2.04031700	2.49104400
H	2.25920700	-2.04002200	-2.49585000	H	2.26756400	-2.04031700	-2.49104400
C	3.18070700	2.91117900	0.72722100	C	3.17720500	2.90862100	0.72037700
C	3.18070700	2.91117900	-0.72722100	C	3.17720500	2.90862100	-0.72037700
C	4.22655700	3.49013000	-1.40834000	C	4.24255100	3.48666700	-1.40274000
C	4.22655700	3.49013000	1.40834000	C	4.24255100	3.48666700	1.40274000
H	1.09517800	0.00000000	-2.44821200	H	1.09658200	0.00000000	-2.44525200
H	1.09517800	0.00000000	2.44821200	H	1.09658200	0.00000000	2.44525200
H	2.25920700	2.04002200	2.49585000	H	2.26756400	2.04031700	2.49104400
H	2.25920700	2.04002200	-2.49585000	H	2.26756400	2.04031700	-2.49104400
H	4.23892500	3.45922800	-2.49550000	H	4.25625100	3.45188400	-2.49004700
H	4.23892500	3.45922800	2.49550000	H	4.25625100	3.45188400	2.49004700
C	4.22655700	-3.49013000	-1.40834000	C	4.24255100	-3.48666700	-1.40274000
C	5.42391200	-3.86572800	-0.72794600	C	5.41924500	-3.86091300	-0.72224100
C	6.62999700	-3.94027200	-1.40940800	C	6.65107600	-3.93399700	-1.40573200
C	5.42391200	-3.86572800	0.72794600	C	5.41924500	-3.86091300	0.72224100
C	6.62999700	-3.94027200	1.40940800	C	6.65107600	-3.93399700	1.40573200
C	7.85160200	-3.70689200	0.72821500	C	7.84990500	-3.70830400	0.72615100
C	7.85160200	-3.70689200	-0.72821500	C	7.84990500	-3.70830400	-0.72615100
C	4.22655700	-3.49013000	1.40834000	C	4.24255100	-3.48666700	1.40274000
C	8.93568900	-3.15306700	1.41008400	C	8.96258200	-3.15266800	1.40879900
C	8.93568900	-3.15306700	-1.41008400	C	8.96258200	-3.15266800	-1.40879900
H	4.23892500	-3.45922800	-2.49550000	H	4.25625100	-3.45188400	-2.49004700
H	4.23892500	-3.45922800	2.49550000	H	4.25625100	-3.45188400	2.49004700
C	9.82597100	-2.29547500	-0.72855000	C	9.83473800	-2.30073500	-0.72922900
C	9.82597100	-2.29547500	0.72855000	C	9.83473800	-2.30073500	0.72922900
C	10.39862100	-1.21342300	1.41066900	C	10.43534400	-1.21558700	1.41013000
C	10.39862100	-1.21342300	-1.41066900	C	10.43534400	-1.21558700	-1.41013000
H	6.62661300	-3.90385100	2.49648500	H	6.64704300	-3.89792800	2.49284100
H	6.62661300	-3.90385100	-2.49648500	H	6.64704300	-3.89792800	-2.49284100
H	8.91154000	-3.12072200	-2.49690500	H	8.94465200	-3.12955000	-2.49588700
H	8.91154000	-3.12072200	2.49690500	H	8.94465200	-3.12955000	2.49588700
H	10.35806600	-1.20002600	2.49737800	H	10.41461600	-1.20893800	2.49723400
H	10.35806600	-1.20002600	-2.49737800	H	10.41461600	-1.20893800	-2.49723400

Dcyc12_R				Dcyc12_U			
C	-5.29116000	4.08143500	0.72810500	C	-5.28727200	4.07461300	0.72255600
C	-5.29116000	4.08143500	-0.72810500	C	-5.28727200	4.07461300	-0.72255600
C	-4.12875700	-3.60799600	1.40829200	C	-4.14457800	-3.60501600	1.40300300
C	-5.29116000	-4.08143500	0.72810500	C	-5.28727200	-4.07461300	0.72255600
C	-6.48024700	-4.28943300	1.40930900	C	-6.50156800	-4.28396300	1.40551500
C	-5.29116000	-4.08143500	-0.72810500	C	-5.28727200	-4.07461300	-0.72255600
C	-6.48024700	-4.28943300	-1.40930900	C	-6.50156800	-4.28396300	-1.40551500
C	-7.72300600	-4.23170600	-0.72863000	C	-7.72055800	-4.22989800	-0.72601600
C	-7.72300600	-4.23170600	0.72863000	C	-7.72055800	-4.22989800	0.72601600
C	-3.12007600	-2.96803400	0.72748500	C	-3.11776900	-2.96361100	0.72096600
C	-4.12875700	-3.60799600	-1.40829200	C	-4.14457800	-3.60501600	-1.40300300
H	-6.48127600	-4.25717200	-2.49656500	H	-6.50215600	-4.25131400	-2.49275900
C	-8.88614900	-3.87796300	-1.41013900	C	-8.91179600	-3.87605500	-1.40820200
C	-8.88614900	-3.87796300	1.41013900	C	-8.91179600	-3.87605500	1.40820200
H	-8.87060400	-3.84573500	-2.49717700	H	-8.89911700	-3.85077900	-2.49539300
H	-8.87060400	-3.84573500	2.49717700	H	-8.89911700	-3.85077900	2.49539300
C	-3.12007600	-2.96803400	-0.72748500	C	-3.11776900	-2.96361100	-0.72096600
H	-4.14175200	-3.58115400	2.49577700	H	-4.15925800	-3.57497300	2.49041700
H	-6.48127600	-4.25717200	2.49656500	H	-6.50215600	-4.25131400	2.49275900
H	-4.14175200	-3.58115400	-2.49577700	H	-4.15925800	-3.57497300	-2.49041700
C	-9.93833300	-3.22775300	0.72903500	C	-9.94532900	-3.23283500	0.72875300
C	-9.93833300	-3.22775300	-0.72903500	C	-9.94532900	-3.23283500	-0.72875300

C	-4.12875700	3.60799600	1.40829200	C	-4.14457800	3.60501600	1.40300300
C	-3.12007600	2.96803400	0.72748500	C	-3.11776900	2.96361100	0.72096600
C	-2.19340000	2.09453500	1.40925600	C	-2.19839100	2.09701500	1.40449000
C	-3.12007600	2.96803400	-0.72748500	C	-3.11776900	2.96361100	-0.72096600
C	-2.19340000	2.09453500	-1.40925600	C	-2.19839100	2.09701500	-1.40449000
C	-1.44204600	1.19619500	-0.72786500	C	-1.44009400	1.19647000	-0.72266800
C	-1.44204600	1.19619500	0.72786500	C	-1.44009400	1.19647000	0.72266800
C	-4.12875700	3.60799600	-1.40829200	C	-4.14457800	3.60501600	-1.40300300
C	-0.78847400	0.00000000	-1.39882800	C	-0.78874600	0.00000000	-1.39655800
C	-0.78847400	0.00000000	1.39882800	C	-0.78874600	0.00000000	1.39655800
H	-4.14175200	3.58115400	2.49577700	H	-4.15925800	3.57497300	2.49041700
H	-4.14175200	3.58115400	-2.49577700	H	-4.15925800	3.57497300	-2.49041700
C	-1.44204600	-1.19619500	0.72786500	C	-1.44009400	-1.19647000	0.72266800
C	-1.44204600	-1.19619500	-0.72786500	C	-1.44009400	-1.19647000	-0.72266800
C	-2.19340000	-2.09453500	-1.40925600	C	-2.19839100	-2.09701500	-1.40449000
C	-2.19340000	-2.09453500	1.40925600	C	-2.19839100	-2.09701500	1.40449000
H	-2.23079600	2.06338400	-2.49631800	H	-2.23627900	2.06614300	-2.49156500
H	-2.23079600	2.06338400	2.49631800	H	-2.23627900	2.06614300	2.49156500
H	-1.09642800	0.00000000	2.44831600	H	-1.09803100	0.00000000	2.44569000
H	-1.09642800	0.00000000	-2.44831600	H	-1.09803100	0.00000000	-2.44569000
H	-2.23079600	-2.06338400	-2.49631800	H	-2.23627900	-2.06614300	-2.49156500
H	-2.23079600	-2.06338400	2.49631800	H	-2.23627900	-2.06614300	2.49156500
C	-10.75927100	2.32265600	1.41075700	C	-10.79292300	2.32511900	1.40943200
C	-9.93833300	3.22775300	0.72903500	C	-9.94532900	3.23283500	0.72875300
C	-8.88614900	3.87796300	1.41013900	C	-8.91179600	3.87605500	1.40820200
C	-9.93833300	3.22775300	-0.72903500	C	-9.94532900	3.23283500	-0.72875300
C	-8.88614900	3.87796300	-1.41013900	C	-8.91179600	3.87605500	-1.40820200
C	-7.72300600	4.23170600	-0.72863000	C	-7.72055800	4.22989800	-0.72601600
C	-7.72300600	4.23170600	0.72863000	C	-7.72055800	4.22989800	0.72601600
C	-11.29424600	1.21333700	0.72919400	C	-11.31212900	1.21700800	0.72978700
C	-10.75927100	2.32265600	-1.41075700	C	-10.79292300	2.32511900	-1.40943200
H	-8.87060400	3.84573500	-2.49717700	H	-8.89911700	3.85077900	-2.49539300
C	-6.48024700	4.28943300	-1.40930900	C	-6.50156800	4.28396300	-1.40551500
C	-6.48024700	4.28943300	1.40930900	C	-6.50156800	4.28396300	1.40551500
H	-6.48127600	4.25717200	-2.49656500	H	-6.50215600	4.25131400	-2.49275900
H	-6.48127600	4.25717200	2.49656500	H	-6.50215600	4.25131400	2.49275900
C	-11.29424600	1.21333700	-0.72919400	C	-11.31212900	1.21700800	-0.72978700
H	-10.72699300	2.30157100	2.49762300	H	-10.77403100	2.31294600	2.49662700
H	-8.87060400	3.84573500	2.49717700	H	-8.89911700	3.85077900	2.49539300
H	-10.72699300	2.30157100	-2.49762300	H	-10.77403100	2.31294600	-2.49662700
C	-10.75927100	-2.32265600	-1.41075700	C	-10.79292300	-2.32511900	-1.40943200
C	-10.75927100	-2.32265600	1.41075700	C	-10.79292300	-2.32511900	1.40943200
C	-11.29424600	-1.21333700	0.72919400	C	-11.31212900	-1.21700800	0.72978700
C	-11.29424600	-1.21333700	-0.72919400	C	-11.31212900	-1.21700800	-0.72978700
C	-11.47663400	0.00000000	-1.41098500	C	-11.51491600	0.00000000	-1.40962000
C	-11.47663400	0.00000000	1.41098500	C	-11.51491600	0.00000000	1.40962000
H	-10.72699300	-2.30157100	2.49762300	H	-10.77403100	-2.31294600	2.49662700
H	-10.72699300	-2.30157100	-2.49762300	H	-10.77403100	-2.31294600	-2.49662700
H	-11.43702000	0.00000000	-2.49778600	H	-11.49491500	0.00000000	-2.49681900
H	-11.43702000	0.00000000	2.49778600	H	-11.49491500	0.00000000	2.49681900
C	9.93833300	3.22775300	0.72903500	C	9.94532900	3.23283500	0.72875300
C	9.93833300	3.22775300	-0.72903500	C	9.94532900	3.23283500	-0.72875300
C	6.48024700	-4.28943300	1.40930900	C	6.50156800	-4.28396300	1.40551500
C	5.29116000	-4.08143500	0.72810500	C	5.28727200	-4.07461300	0.72255600
C	4.12875700	-3.60799600	1.40829200	C	4.14457800	-3.60501600	1.40300300
C	5.29116000	-4.08143500	-0.72810500	C	5.28727200	-4.07461300	-0.72255600
C	4.12875700	-3.60799600	-1.40829200	C	4.14457800	-3.60501600	-1.40300300
C	3.12007600	-2.96803400	-0.72748500	C	3.11776900	-2.96361100	-0.72096600
C	3.12007600	-2.96803400	0.72748500	C	3.11776900	-2.96361100	0.72096600
C	7.72300600	-4.23170600	0.72863000	C	7.72055800	-4.22989800	0.72601600

C	6.48024700	-4.28943300	-1.40930900	C	6.50156800	-4.28396300	-1.40551500
H	4.14175200	-3.58115400	-2.49577700	H	4.15925800	-3.57497300	-2.49041700
C	2.19340000	-2.09453500	-1.40925600	C	2.19839100	-2.09701500	-1.40449000
C	2.19340000	-2.09453500	1.40925600	C	2.19839100	-2.09701500	1.40449000
H	2.23079600	-2.06338400	-2.49631800	H	2.23627900	-2.06614300	-2.49156500
H	2.23079600	-2.06338400	2.49631800	H	2.23627900	-2.06614300	2.49156500
C	7.72300600	-4.23170600	-0.72863000	C	7.72055800	-4.22989800	-0.72601600
H	6.48127600	-4.25717200	2.49656500	H	6.50215600	-4.25131400	2.49275900
H	4.14175200	-3.58115400	2.49577700	H	4.15925800	-3.57497300	2.49041700
H	6.48127600	-4.25717200	-2.49656500	H	6.50215600	-4.25131400	-2.49275900
C	1.44204600	-1.19619500	0.72786500	C	1.44009400	-1.19647000	0.72266800
C	1.44204600	-1.19619500	-0.72786500	C	1.44009400	-1.19647000	-0.72266800
C	10.75927100	2.32265600	1.41075700	C	10.79292300	2.32511900	1.40943200
C	11.29424600	1.21333700	0.72919400	C	11.31212900	1.21700800	0.72978700
C	11.47663400	0.00000000	1.41098500	C	11.51491600	0.00000000	1.40962000
C	11.29424600	1.21333700	-0.72919400	C	11.31212900	1.21700800	-0.72978700
C	11.47663400	0.00000000	-1.41098500	C	11.51491600	0.00000000	-1.40962000
C	11.29424600	-1.21333700	-0.72919400	C	11.31212900	-1.21700800	-0.72978700
C	11.29424600	-1.21333700	0.72919400	C	11.31212900	-1.21700800	0.72978700
C	10.75927100	2.32265600	-1.41075700	C	10.79292300	2.32511900	-1.40943200
C	10.75927100	-2.32265600	-1.41075700	C	10.79292300	-2.32511900	-1.40943200
C	10.75927100	-2.32265600	1.41075700	C	10.79292300	-2.32511900	1.40943200
H	10.72699300	2.30157100	2.49762300	H	10.77403100	2.31294600	2.49662700
H	10.72699300	2.30157100	-2.49762300	H	10.77403100	2.31294600	-2.49662700
C	9.93833300	-3.22775300	0.72903500	C	9.94532900	-3.23283500	0.72875300
C	9.93833300	-3.22775300	-0.72903500	C	9.94532900	-3.23283500	-0.72875300
C	8.88614900	-3.87796300	-1.41013900	C	8.91179600	-3.87605500	-1.40820200
C	8.88614900	-3.87796300	1.41013900	C	8.91179600	-3.87605500	1.40820200
H	11.43702000	0.00000000	-2.49778600	H	11.49491500	0.00000000	-2.49681900
H	11.43702000	0.00000000	2.49778600	H	11.49491500	0.00000000	2.49681900
H	10.72699300	-2.30157100	2.49762300	H	10.77403100	-2.31294600	2.49662700
H	10.72699300	-2.30157100	-2.49762300	H	10.77403100	-2.31294600	-2.49662700
H	8.87060400	-3.84573500	-2.49717700	H	8.89911700	-3.85077900	-2.49539300
H	8.87060400	-3.84573500	2.49717700	H	8.89911700	-3.85077900	2.49539300
C	4.12875700	3.60799600	1.40829200	C	4.14457800	3.60501600	1.40300300
C	5.29116000	4.08143500	0.72810500	C	5.28727200	4.07461300	0.72255600
C	6.48024700	4.28943300	1.40930900	C	6.50156800	4.28396300	1.40551500
C	5.29116000	4.08143500	-0.72810500	C	5.28727200	4.07461300	-0.72255600
C	6.48024700	4.28943300	-1.40930900	C	6.50156800	4.28396300	-1.40551500
C	7.72300600	4.23170600	-0.72863000	C	7.72055800	4.22989800	-0.72601600
C	7.72300600	4.23170600	0.72863000	C	7.72055800	4.22989800	0.72601600
C	3.12007600	2.96803400	0.72748500	C	3.11776900	2.96361100	0.72096600
C	4.12875700	3.60799600	-1.40829200	C	4.14457800	3.60501600	-1.40300300
H	6.48127600	4.25717200	-2.49656500	H	6.50215600	4.25131400	-2.49275900
C	8.88614900	3.87796300	-1.41013900	C	8.91179600	3.87605500	-1.40820200
C	8.88614900	3.87796300	1.41013900	C	8.91179600	3.87605500	1.40820200
H	8.87060400	3.84573500	-2.49717700	H	8.89911700	3.85077900	-2.49539300
H	8.87060400	3.84573500	2.49717700	H	8.89911700	3.85077900	2.49539300
C	3.12007600	2.96803400	-0.72748500	C	3.11776900	2.96361100	-0.72096600
H	4.14175200	3.58115400	2.49577700	H	4.15925800	3.57497300	2.49041700
H	6.48127600	4.25717200	2.49656500	H	6.50215600	4.25131400	2.49275900
H	4.14175200	3.58115400	-2.49577700	H	4.15925800	3.57497300	-2.49041700
C	0.78847400	0.00000000	-1.39882800	C	0.78874600	0.00000000	-1.39655800
C	0.78847400	0.00000000	1.39882800	C	0.78874600	0.00000000	1.39655800
C	1.44204600	1.19619500	0.72786500	C	1.44009400	1.19647000	0.72266800
C	1.44204600	1.19619500	-0.72786500	C	1.44009400	1.19647000	-0.72266800
C	2.19340000	2.09453500	-1.40925600	C	2.19839100	2.09701500	-1.40449000
C	2.19340000	2.09453500	1.40925600	C	2.19839100	2.09701500	1.40449000
H	1.09642800	0.00000000	2.44831600	H	1.09803100	0.00000000	2.44569000
H	1.09642800	0.00000000	-2.44831600	H	1.09803100	0.00000000	-2.44569000

H	2.23079600	2.06338400	-2.49631800	H	2.23627900	2.06614300	-2.49156500
H	2.23079600	2.06338400	2.49631800	H	2.23627900	2.06614300	2.49156500
**Dcyc13_R**				**Dcyc13_U**			
C	9.92606100	4.01198200	0.72943100	C	9.92847200	4.01768800	0.72854900
C	9.92606100	4.01198200	-0.72943100	C	9.92847200	4.01768800	-0.72854900
C	10.91300500	-3.29389500	1.41083100	C	10.94000200	-3.29732900	1.40907100
C	9.92606100	-4.01198200	0.72943100	C	9.92847200	-4.01768800	0.72854900
C	8.77733200	-4.47147600	1.41014000	C	8.79956900	-4.47238400	1.40796000
C	9.92606100	-4.01198200	-0.72943100	C	9.92847200	-4.01768800	-0.72854900
C	8.77733200	-4.47147600	-1.41014000	C	8.79956900	-4.47238400	-1.40796000
C	7.57647600	-4.65406000	-0.72895100	C	7.57159900	-4.65396800	-0.72602800
C	7.57647600	-4.65406000	0.72895100	C	7.57159900	-4.65396800	0.72602800
C	11.69096900	-2.33816800	0.72969700	C	11.70378500	-2.34433100	0.72939600
C	10.91300500	-3.29389500	-1.41083100	C	10.94000200	-3.29732900	-1.40907100
H	8.76806700	-4.44085800	-2.49730700	H	8.79167500	-4.44732600	-2.49523800
C	6.33521300	-4.56885200	-1.40933300	C	6.35387200	-4.56746000	-1.40550200
C	6.33521300	-4.56885200	1.40933300	C	6.35387200	-4.56746000	1.40550200
H	6.33899300	-4.54033100	-2.49669300	H	6.35740300	-4.53829800	-2.49286100
H	6.33899300	-4.54033100	2.49669300	H	6.35740300	-4.53829800	2.49286100
C	11.69096900	-2.33816800	-0.72969700	C	11.70378500	-2.34433100	-0.72939600
H	10.88879300	-3.26925700	2.49785400	H	10.92430200	-3.28129700	2.49632700
H	8.76806700	-4.44085800	2.49730700	H	8.79167500	-4.44732600	2.49523800
H	10.88879300	-3.26925700	-2.49785400	H	10.92430200	-3.28129700	-2.49632700
C	5.17066100	-4.25380300	0.72834600	C	5.16513700	-4.24912700	0.72277900
C	5.17066100	-4.25380300	-0.72834600	C	5.16513700	-4.24912700	-0.72277900
C	10.91300500	3.29389500	1.41083100	C	10.94000200	3.29732900	1.40907100
C	10.91300500	3.29389500	-1.41083100	C	10.94000200	3.29732900	-1.40907100
C	11.69096900	2.33816800	-0.72969700	C	11.70378500	2.34433100	-0.72939600
C	11.69096900	2.33816800	0.72969700	C	11.70378500	2.34433100	0.72939600
C	12.18355100	1.21677300	-1.41117300	C	12.21491300	1.21872100	-1.40910900
C	12.18355100	1.21677300	1.41117300	C	12.21491300	1.21872100	1.40910900
C	12.35329400	0.00000000	0.72978300	C	12.37112600	0.00000000	0.72945400
C	12.35329400	0.00000000	-0.72978300	C	12.37112600	0.00000000	-0.72945400
C	12.18355100	-1.21677300	-1.41117300	C	12.21491300	-1.21872100	-1.40910900
C	12.18355100	-1.21677300	1.41117300	C	12.21491300	-1.21872100	1.40910900
H	10.88879300	3.26925700	-2.49785400	H	10.92430200	3.28129700	-2.49632700
H	10.88879300	3.26925700	2.49785400	H	10.92430200	3.28129700	2.49632700
H	12.14877100	1.20717900	2.49810300	H	12.19529000	1.21320000	2.49636200
H	12.14877100	1.20717900	-2.49810300	H	12.19529000	1.21320000	-2.49636200
H	12.14877100	-1.20717900	-2.49810300	H	12.19529000	-1.21320000	-2.49636200
H	12.14877100	-1.20717900	2.49810300	H	12.19529000	-1.21320000	2.49636200
C	4.04306400	3.70271700	1.40836900	C	4.05614500	3.70313900	1.40318800
C	5.17066100	4.25380300	0.72834600	C	5.16513700	4.24912700	0.72277900
C	6.33521300	4.56885200	1.40933300	C	6.35387200	4.56746000	1.40550200
C	5.17066100	4.25380300	-0.72834600	C	5.16513700	4.24912700	-0.72277900
C	6.33521300	4.56885200	-1.40933300	C	6.35387200	4.56746000	-1.40550200
C	7.57647600	4.65406000	-0.72895100	C	7.57159900	4.65396800	-0.72602800
C	7.57647600	4.65406000	0.72895100	C	7.57159900	4.65396800	0.72602800
C	3.06787500	3.01367600	0.72768900	C	3.06399800	3.01135000	0.72126300
C	4.04306400	3.70271700	-1.40836900	C	4.05614500	3.70313900	-1.40318800
H	6.33899300	4.54033100	-2.49669300	H	6.35740300	4.53829800	-2.49286100
C	8.77733200	4.47147600	-1.41014000	C	8.79956900	4.47238400	-1.40796000
C	8.77733200	4.47147600	1.41014000	C	8.79956900	4.47238400	1.40796000
H	8.76806700	4.44085800	-2.49730700	H	8.79167500	4.44732600	-2.49523800
H	8.76806700	4.44085800	2.49730700	H	8.79167500	4.44732600	2.49523800
C	3.06787500	3.01367600	-0.72768900	C	3.06399800	3.01135000	-0.72126300
H	4.05627200	3.67907900	2.49592300	H	4.07090800	3.67705700	2.49071400
H	6.33899300	4.54033100	2.49669300	H	6.35740300	4.53829800	2.49286100

H	4.05627200	3.67907900	-2.49592300	H	4.07090800	3.67705700	-2.49071400
C	4.04306400	-3.70271700	1.40836900	C	4.05614500	-3.70313900	1.40318800
C	3.06787500	-3.01367600	0.72768900	C	3.06399800	-3.01135000	0.72126300
C	2.16919600	-2.11167500	1.40943300	C	2.17355200	-2.11462700	1.40487600
C	3.06787500	-3.01367600	-0.72768900	C	3.06399800	-3.01135000	-0.72126300
C	2.16919600	-2.11167500	-1.40943300	C	2.17355200	-2.11462700	-1.40487600
C	1.43683800	-1.19842300	-0.72788600	C	1.43488500	-1.19897900	-0.72294900
C	1.43683800	-1.19842300	0.72788600	C	1.43488500	-1.19897900	0.72294900
C	4.04306400	-3.70271700	-1.40836900	C	4.05614500	-3.70313900	-1.40318800
C	0.78933000	0.00000000	-1.39916200	C	0.78968000	0.00000000	-1.39677600
C	0.78933000	0.00000000	1.39916200	C	0.78968000	0.00000000	1.39677600
H	4.05627200	-3.67907900	2.49592300	H	4.07090800	-3.67705700	2.49071400
H	4.05627200	-3.67907900	-2.49592300	H	4.07090800	-3.67705700	-2.49071400
C	1.43683800	1.19842300	0.72788600	C	1.43488500	1.19897900	0.72294900
C	1.43683800	1.19842300	-0.72788600	C	1.43488500	1.19897900	-0.72294900
C	2.16919600	2.11167500	-1.40943300	C	2.17355200	2.11462700	-1.40487600
C	2.16919600	2.11167500	1.40943300	C	2.17355200	2.11462700	1.40487600
H	2.20461500	-2.08392200	-2.49663700	H	2.21027100	-2.08654800	-2.49206600
H	2.20461500	-2.08392200	2.49663700	H	2.21027100	-2.08654800	2.49206600
H	1.09775900	0.00000000	2.44851100	H	1.09908600	0.00000000	2.44588200
H	1.09775900	0.00000000	-2.44851100	H	1.09908600	0.00000000	-2.44588200
H	2.20461500	2.08392200	-2.49663700	H	2.21027100	2.08654800	-2.49206600
H	2.20461500	2.08392200	2.49663700	H	2.21027100	2.08654800	2.49206600
C	-5.17066100	4.25380300	0.72834600	C	-5.16513700	4.24912700	0.72277900
C	-5.17066100	4.25380300	-0.72834600	C	-5.16513700	4.24912700	-0.72277900
C	-2.16919600	-2.11167500	1.40943300	C	-2.17355200	-2.11462700	1.40487600
C	-3.06787500	-3.01367600	0.72768900	C	-3.06399800	-3.01135000	0.72126300
C	-4.04306400	-3.70271700	1.40836900	C	-4.05614500	-3.70313900	1.40318800
C	-3.06787500	-3.01367600	-0.72768900	C	-3.06399800	-3.01135000	-0.72126300
C	-4.04306400	-3.70271700	-1.40836900	C	-4.05614500	-3.70313900	-1.40318800
C	-5.17066100	-4.25380300	-0.72834600	C	-5.16513700	-4.24912700	-0.72277900
C	-5.17066100	-4.25380300	0.72834600	C	-5.16513700	-4.24912700	0.72277900
C	-1.43683800	-1.19842300	0.72788600	C	-1.43488500	-1.19897900	0.72294900
C	-2.16919600	-2.11167500	-1.40943300	C	-2.17355200	-2.11462700	-1.40487600
H	-4.05627200	-3.67907900	-2.49592300	H	-4.07090800	-3.67705700	-2.49071400
C	-6.33521300	-4.56885200	-1.40933300	C	-6.35387200	-4.56746000	-1.40550200
C	-6.33521300	-4.56885200	1.40933300	C	-6.35387200	-4.56746000	1.40550200
H	-6.33899300	-4.54033100	-2.49669300	H	-6.35740300	-4.53829800	-2.49286100
H	-6.33899300	-4.54033100	2.49669300	H	-6.35740300	-4.53829800	2.49286100
C	-1.43683800	-1.19842300	-0.72788600	C	-1.43488500	-1.19897900	-0.72294900
H	-2.20461500	-2.08392200	2.49663700	H	-2.21027100	-2.08654800	2.49206600
H	-4.05627200	-3.67907900	2.49592300	H	-4.07090800	-3.67705700	2.49071400
H	-2.20461500	-2.08392200	-2.49663700	H	-2.21027100	-2.08654800	-2.49206600
C	-7.57647600	-4.65406000	0.72895100	C	-7.57159900	-4.65396800	0.72602800
C	-7.57647600	-4.65406000	-0.72895100	C	-7.57159900	-4.65396800	-0.72602800
C	-4.04306400	3.70271700	1.40836900	C	-4.05614500	3.70313900	1.40318800
C	-4.04306400	3.70271700	-1.40836900	C	-4.05614500	3.70313900	-1.40318800
C	-3.06787500	3.01367600	-0.72768900	C	-3.06399800	3.01135000	-0.72126300
C	-3.06787500	3.01367600	0.72768900	C	-3.06399800	3.01135000	0.72126300
C	-2.16919600	2.11167500	-1.40943300	C	-2.17355200	2.11462700	-1.40487600
C	-2.16919600	2.11167500	1.40943300	C	-2.17355200	2.11462700	1.40487600
C	-1.43683800	1.19842300	0.72788600	C	-1.43488500	1.19897900	0.72294900
C	-1.43683800	1.19842300	-0.72788600	C	-1.43488500	1.19897900	-0.72294900
C	-0.78933000	0.00000000	-1.39916200	C	-0.78968000	0.00000000	-1.39677600
C	-0.78933000	0.00000000	1.39916200	C	-0.78968000	0.00000000	1.39677600
H	-4.05627200	3.67907900	-2.49592300	H	-4.07090800	3.67705700	-2.49071400
H	-4.05627200	3.67907900	2.49592300	H	-4.07090800	3.67705700	2.49071400
H	-2.20461500	2.08392200	2.49663700	H	-2.21027100	2.08654800	2.49206600
H	-2.20461500	2.08392200	-2.49663700	H	-2.21027100	2.08654800	-2.49206600
H	-1.09775900	0.00000000	-2.44851100	H	-1.09908600	0.00000000	-2.44588200

H	-1.09775900	0.00000000	2.44851100	H	-1.09908600	0.00000000	2.44588200
C	-10.91300500	3.29389500	1.41083100	C	-10.94000200	3.29732900	1.40907100
C	-9.92606100	4.01198200	0.72943100	C	-9.92847200	4.01768800	0.72854900
C	-8.77733200	4.47147600	1.41014000	C	-8.79956900	4.47238400	1.40796000
C	-9.92606100	4.01198200	-0.72943100	C	-9.92847200	4.01768800	-0.72854900
C	-8.77733200	4.47147600	-1.41014000	C	-8.79956900	4.47238400	-1.40796000
C	-7.57647600	4.65406000	-0.72895100	C	-7.57159900	4.65396800	-0.72602800
C	-7.57647600	4.65406000	0.72895100	C	-7.57159900	4.65396800	0.72602800
C	-11.69096900	2.33816800	0.72969700	C	-11.70378500	2.34433100	0.72939600
C	-10.91300500	3.29389500	-1.41083100	C	-10.94000200	3.29732900	-1.40907100
H	-8.76806700	4.44085800	-2.49730700	H	-8.79167500	4.44732600	-2.49523800
C	-6.33521300	4.56885200	-1.40933300	C	-6.35387200	4.56746000	-1.40550200
C	-6.33521300	4.56885200	1.40933300	C	-6.35387200	4.56746000	1.40550200
H	-6.33899300	4.54033100	-2.49669300	H	-6.35740300	4.53829800	-2.49286100
H	-6.33899300	4.54033100	2.49669300	H	-6.35740300	4.53829800	2.49286100
C	-11.69096900	2.33816800	-0.72969700	C	-11.70378500	2.34433100	-0.72939600
H	-10.88879300	3.26925700	2.49785400	H	-10.92430200	3.28129700	2.49632700
H	-8.76806700	4.44085800	2.49730700	H	-8.79167500	4.44732600	2.49523800
H	-10.88879300	3.26925700	-2.49785400	H	-10.92430200	3.28129700	-2.49632700
C	-8.77733200	-4.47147600	1.41014000	C	-8.79956900	-4.47238400	1.40796000
C	-9.92606100	-4.01198200	0.72943100	C	-9.92847200	-4.01768800	0.72854900
C	-10.91300500	-3.29389500	1.41083100	C	-10.94000200	-3.29732900	1.40907100
C	-9.92606100	-4.01198200	-0.72943100	C	-9.92847200	-4.01768800	-0.72854900
C	-10.91300500	-3.29389500	-1.41083100	C	-10.94000200	-3.29732900	-1.40907100
C	-11.69096900	-2.33816800	-0.72969700	C	-11.70378500	-2.34433100	-0.72939600
C	-11.69096900	-2.33816800	0.72969700	C	-11.70378500	-2.34433100	0.72939600
C	-8.77733200	-4.47147600	-1.41014000	C	-8.79956900	-4.47238400	-1.40796000
C	-12.18355100	-1.21677300	-1.41117300	C	-12.21491300	-1.21872100	-1.40910900
C	-12.18355100	-1.21677300	1.41117300	C	-12.21491300	-1.21872100	1.40910900
H	-8.76806700	-4.44085800	2.49730700	H	-8.79167500	-4.44732600	2.49523800
H	-8.76806700	-4.44085800	-2.49730700	H	-8.79167500	-4.44732600	-2.49523800
C	-12.35329400	0.00000000	0.72978300	C	-12.37112600	0.00000000	0.72945400
C	-12.35329400	0.00000000	-0.72978300	C	-12.37112600	0.00000000	-0.72945400
C	-12.18355100	1.21677300	-1.41117300	C	-12.21491300	1.21872100	-1.40910900
C	-12.18355100	1.21677300	1.41117300	C	-12.21491300	1.21872100	1.40910900
H	-10.88879300	-3.26925700	-2.49785400	H	-10.92430200	-3.28129700	-2.49632700
H	-10.88879300	-3.26925700	2.49785400	H	-10.92430200	-3.28129700	2.49632700
H	-12.14877100	-1.20717900	2.49810300	H	-12.19529000	-1.21320000	2.49636200
H	-12.14877100	-1.20717900	-2.49810300	H	-12.19529000	-1.21320000	-2.49636200
H	-12.14877100	1.20717900	-2.49810300	H	-12.19529000	1.21320000	-2.49636200
H	-12.14877100	1.20717900	2.49810300	H	-12.19529000	1.21320000	2.49636200
**Dcyc14_R**				**Dcyc14_U**			
C	-7.43308600	4.97411600	0.72451200	C	-7.42375200	4.99550100	0.72621800
C	-7.43308600	4.97411600	-0.72451200	C	-7.42375200	4.99550100	-0.72621800
C	-3.99346900	-3.76912200	1.40277800	C	-3.98187200	-3.78057100	1.40331700
C	-5.07029500	-4.37175600	0.72186300	C	-5.05879700	-4.38773800	0.72301900
C	-6.23732400	-4.77210900	1.40413900	C	-6.21926700	-4.79445300	1.40555200
C	-5.07029500	-4.37175600	-0.72186300	C	-5.05879700	-4.38773800	-0.72301900
C	-6.23732400	-4.77210900	-1.40413900	C	-6.21926700	-4.79445300	-1.40555200
C	-7.43308600	-4.97411600	-0.72451200	C	-7.42375200	-4.99550100	-0.72621800
C	-7.43308600	-4.97411600	0.72451200	C	-7.42375200	-4.99550100	0.72621800
C	-3.02753400	-3.04116400	0.72126000	C	-3.02018500	-3.04803700	0.72151500
C	-3.99346900	-3.76912200	-1.40277800	C	-3.98187200	-3.78057100	-1.40331700
H	-6.24360400	-4.74309200	-2.49160100	H	-6.22454400	-4.76866100	-2.49299400
C	-8.68500700	-4.93116500	-1.40625000	C	-8.66339300	-4.95981600	-1.40782200
C	-8.68500700	-4.93116500	1.40625000	C	-8.66339300	-4.95981600	1.40782200
H	-8.67983900	-4.90277500	-2.49372100	H	-8.65923400	-4.93632700	-2.49518000
H	-8.67983900	-4.90277500	2.49372100	H	-8.65923400	-4.93632700	2.49518000

C	-3.02753400	-3.04116400	-0.72126000	C	-3.02018500	-3.04803700	-0.72151500
H	-4.00876700	-3.74527800	2.49036100	H	-3.99649300	-3.75747600	2.49090800
H	-6.24360400	-4.74309200	2.49160100	H	-6.22454400	-4.76866100	2.49299400
H	-4.00876700	-3.74527800	-2.49036100	H	-3.99649300	-3.75747600	-2.49090800
C	-9.85280000	-4.65749800	0.72829900	C	-9.84610600	-4.67131100	0.72856600
C	-9.85280000	-4.65749800	-0.72829900	C	-9.84610600	-4.67131100	-0.72856600
C	-3.99346900	3.76912200	1.40277800	C	-3.98187200	3.78057100	1.40331700
C	-3.02753400	3.04116400	0.72126000	C	-3.02018500	3.04803700	0.72151500
C	-2.15665800	2.12605400	1.40493100	C	-2.15309600	2.12875300	1.40506500
C	-3.02753400	3.04116400	-0.72126000	C	-3.02018500	3.04803700	-0.72151500
C	-2.15665800	2.12605400	-1.40493100	C	-2.15309600	2.12875300	-1.40506500
C	-1.43155200	1.20003400	-0.72285900	C	-1.43080500	1.20070100	-0.72302500
C	-1.43155200	1.20003400	0.72285900	C	-1.43080500	1.20070100	0.72302500
C	-3.99346900	3.76912200	-1.40277800	C	-3.98187200	3.78057100	-1.40331700
C	-0.79032800	0.00000000	-1.39717800	C	-0.79047100	0.00000000	-1.39708900
C	-0.79032800	0.00000000	1.39717800	C	-0.79047100	0.00000000	1.39708900
H	-4.00876700	3.74527800	2.49036100	H	-3.99649300	3.75747600	2.49090800
H	-4.00876700	3.74527800	-2.49036100	H	-3.99649300	3.75747600	-2.49090800
C	-1.43155200	-1.20003400	0.72285900	C	-1.43080500	-1.20070100	0.72302500
C	-1.43155200	-1.20003400	-0.72285900	C	-1.43080500	-1.20070100	-0.72302500
C	-2.15665800	-2.12605400	-1.40493100	C	-2.15309600	-2.12875300	-1.40506500
C	-2.15665800	-2.12605400	1.40493100	C	-2.15309600	-2.12875300	1.40506500
H	-2.19114700	2.10089000	-2.49224500	H	-2.18778800	2.10366800	-2.49238800
H	-2.19114700	2.10089000	2.49224500	H	-2.18778800	2.10366800	2.49238800
H	-1.10009000	0.00000000	2.44616100	H	-1.10016500	0.00000000	2.44611500
H	-1.10009000	0.00000000	-2.44616100	H	-1.10016500	0.00000000	-2.44611500
H	-2.19114700	-2.10089000	-2.49224500	H	-2.18778800	-2.10366800	-2.49238800
H	-2.19114700	-2.10089000	2.49224500	H	-2.18778800	-2.10366800	2.49238800
C	-12.68851300	2.35416100	1.40565700	C	-12.64322100	2.35965300	1.40870000
C	-11.91269100	3.34595100	0.73214800	C	-11.89912000	3.34714100	0.72914700
C	-10.99076000	4.10983300	1.40741400	C	-10.96295500	4.13070000	1.40878400
C	-11.91269100	3.34595100	-0.73214800	C	-11.89912000	3.34714100	-0.72914700
C	-10.99076000	4.10983300	-1.40741400	C	-10.96295500	4.13070000	-1.40878400
C	-9.85280000	4.65749800	-0.72829900	C	-9.84610600	4.67131100	-0.72856600
C	-9.85280000	4.65749800	0.72829900	C	-9.84610600	4.67131100	0.72856600
C	-13.11733100	1.22123800	0.73481700	C	-13.09496900	1.21969200	0.72890400
C	-12.68851300	2.35416100	-1.40565700	C	-12.64322100	2.35965300	-1.40870000
H	-10.98012900	4.09560200	-2.49513400	H	-10.95114000	4.11295700	-2.49610700
C	-8.68500700	4.93116500	-1.40625000	C	-8.66339300	4.95981600	-1.40782200
C	-8.68500700	4.93116500	1.40625000	C	-8.66339300	4.95981600	1.40782200
H	-8.67983900	4.90277500	-2.49372100	H	-8.65923400	4.93632700	-2.49518000
H	-8.67983900	4.90277500	2.49372100	H	-8.65923400	4.93632700	2.49518000
C	-13.11733100	1.22123800	-0.73481700	C	-13.09496900	1.21969200	-0.72890400
H	-12.69040000	2.35605000	2.49353600	H	-12.62525000	2.34949100	2.49600800
H	-10.98012900	4.09560200	2.49513400	H	-10.95114000	4.11295700	2.49610700
H	-12.69040000	2.35605000	-2.49353600	H	-12.62525000	2.34949100	-2.49600800
C	-10.99076000	-4.10983300	1.40741400	C	-10.96295500	-4.13070000	1.40878400
C	-11.91269100	-3.34595100	0.73214800	C	-11.89912000	-3.34714100	0.72914700
C	-12.68851300	-2.35416100	1.40565700	C	-12.64322100	-2.35965300	1.40870000
C	-11.91269100	-3.34595100	-0.73214800	C	-11.89912000	-3.34714100	-0.72914700
C	-12.68851300	-2.35416100	-1.40565700	C	-12.64322100	-2.35965300	-1.40870000
C	-10.99076000	-4.10983300	-1.40741400	C	-10.96295500	-4.13070000	-1.40878400
H	-10.98012900	-4.09560200	2.49513400	H	-10.95114000	-4.11295700	2.49610700
H	-10.98012900	-4.09560200	-2.49513400	H	-10.95114000	-4.11295700	-2.49610700
C	-13.11733100	-1.22123800	0.73481700	C	-13.09496900	-1.21969200	0.72890400
C	-13.11733100	-1.22123800	-0.73481700	C	-13.09496900	-1.21969200	-0.72890400
C	-13.32349800	0.00000000	-1.40335200	C	-13.26397100	0.00000000	-1.40852100
C	-13.32349800	0.00000000	1.40335200	C	-13.26397100	0.00000000	1.40852100
H	-12.69040000	-2.35605000	-2.49353600	H	-12.62525000	-2.34949100	-2.49600800
H	-12.69040000	-2.35605000	2.49353600	H	-12.62525000	-2.34949100	2.49600800

H	-13.33739100	0.00000000	-2.49117300	H	-13.24314600	0.00000000	-2.49582500
H	-13.33739100	0.00000000	2.49117300	H	-13.24314600	0.00000000	2.49582500
C	-6.23732400	4.77210900	-1.40413900	C	-6.21926700	4.79445300	-1.40555200
C	-5.07029500	4.37175600	-0.72186300	C	-5.05879700	4.38773800	-0.72301900
C	-5.07029500	4.37175600	0.72186300	C	-5.05879700	4.38773800	0.72301900
C	-6.23732400	4.77210900	1.40413900	C	-6.21926700	4.79445300	1.40555200
H	-6.24360400	4.74309200	-2.49160100	H	-6.22454400	4.76866100	-2.49299400
H	-6.24360400	4.74309200	2.49160100	H	-6.22454400	4.76866100	2.49299400
C	5.07029500	4.37175600	0.72186300	C	5.05879700	4.38773800	0.72301900
C	5.07029500	4.37175600	-0.72186300	C	5.05879700	4.38773800	-0.72301900
C	12.68851300	-2.35416100	1.40565700	C	12.64322100	-2.35965300	1.40870000
C	11.91269100	-3.34595100	0.73214800	C	11.89912000	-3.34714100	0.72914700
C	10.99076000	-4.10983300	1.40741400	C	10.96295500	-4.13070000	1.40878400
C	11.91269100	-3.34595100	-0.73214800	C	11.89912000	-3.34714100	-0.72914700
C	10.99076000	-4.10983300	-1.40741400	C	10.96295500	-4.13070000	-1.40878400
C	9.85280000	-4.65749800	-0.72829900	C	9.84610600	-4.67131100	-0.72856600
C	9.85280000	-4.65749800	0.72829900	C	9.84610600	-4.67131100	0.72856600
C	13.11733100	-1.22123800	0.73481700	C	13.09496900	-1.21969200	0.72890400
C	12.68851300	-2.35416100	-1.40565700	C	12.64322100	-2.35965300	-1.40870000
H	10.98012900	-4.09560200	-2.49513400	H	10.95114000	-4.11295700	-2.49610700
C	8.68500700	-4.93116500	-1.40625000	C	8.66339300	-4.95981600	-1.40782200
C	8.68500700	-4.93116500	1.40625000	C	8.66339300	-4.95981600	1.40782200
H	8.67983900	-4.90277500	-2.49372100	H	8.65923400	-4.93632700	-2.49518000
H	8.67983900	-4.90277500	2.49372100	H	8.65923400	-4.93632700	2.49518000
C	13.11733100	-1.22123800	-0.73481700	C	13.09496900	-1.21969200	-0.72890400
H	12.69040000	-2.35605000	2.49353600	H	12.62525000	-2.34949100	2.49600800
H	10.98012900	-4.09560200	2.49513400	H	10.95114000	-4.11295700	2.49610700
H	12.69040000	-2.35605000	-2.49353600	H	12.62525000	-2.34949100	-2.49600800
C	7.43308600	-4.97411600	0.72451200	C	7.42375200	-4.99550100	0.72621800
C	7.43308600	-4.97411600	-0.72451200	C	7.42375200	-4.99550100	-0.72621800
C	8.68500700	4.93116500	1.40625000	C	8.66339300	4.95981600	1.40782200
C	9.85280000	4.65749800	0.72829900	C	9.84610600	4.67131100	0.72856600
C	10.99076000	4.10983300	1.40741400	C	10.96295500	4.13070000	1.40878400
C	9.85280000	4.65749800	-0.72829900	C	9.84610600	4.67131100	-0.72856600
C	10.99076000	4.10983300	-1.40741400	C	10.96295500	4.13070000	-1.40878400
C	11.91269100	3.34595100	-0.73214800	C	11.89912000	3.34714100	-0.72914700
C	11.91269100	3.34595100	0.73214800	C	11.89912000	3.34714100	0.72914700
C	8.68500700	4.93116500	-1.40625000	C	8.66339300	4.95981600	-1.40782200
C	12.68851300	2.35416100	-1.40565700	C	12.64322100	2.35965300	-1.40870000
C	12.68851300	2.35416100	1.40565700	C	12.64322100	2.35965300	1.40870000
H	8.67983900	4.90277500	2.49372100	H	8.65923400	4.93632700	2.49518000
H	8.67983900	4.90277500	-2.49372100	H	8.65923400	4.93632700	-2.49518000
C	13.11733100	1.22123800	0.73481700	C	13.09496900	1.21969200	0.72890400
C	13.11733100	1.22123800	-0.73481700	C	13.09496900	1.21969200	-0.72890400
C	13.32349800	0.00000000	-1.40335200	C	13.26397100	0.00000000	-1.40852100
C	13.32349800	0.00000000	1.40335200	C	13.26397100	0.00000000	1.40852100
H	10.98012900	4.09560200	-2.49513400	H	10.95114000	4.11295700	-2.49610700
H	10.98012900	4.09560200	2.49513400	H	10.95114000	4.11295700	2.49610700
H	12.69040000	2.35605000	2.49353600	H	12.62525000	2.34949100	2.49600800
H	12.69040000	2.35605000	-2.49353600	H	12.62525000	2.34949100	-2.49600800
H	13.33739100	0.00000000	-2.49117300	H	13.24314600	0.00000000	-2.49582500
H	13.33739100	0.00000000	2.49117300	H	13.24314600	0.00000000	2.49582500
C	0.79032800	0.00000000	1.39717800	C	0.79047100	0.00000000	1.39708900
C	1.43155200	1.20003400	0.72285900	C	1.43080500	1.20070100	0.72302500
C	2.15665800	2.12605400	1.40493100	C	2.15309600	2.12875300	1.40506500
C	1.43155200	1.20003400	-0.72285900	C	1.43080500	1.20070100	-0.72302500
C	2.15665800	2.12605400	-1.40493100	C	2.15309600	2.12875300	-1.40506500
C	3.02753400	3.04116400	-0.72126000	C	3.02018500	3.04803700	-0.72151500
C	3.02753400	3.04116400	0.72126000	C	3.02018500	3.04803700	0.72151500
C	1.43155200	-1.20003400	0.72285900	C	1.43080500	-1.20070100	0.72302500

C	0.79032800	0.00000000	-1.39717800	C	0.79047100	0.00000000	-1.39708900
H	2.19114700	2.10089000	-2.49224500	H	2.18778800	2.10366800	-2.49238800
C	3.99346900	3.76912200	-1.40277800	C	3.98187200	3.78057100	-1.40331700
C	3.99346900	3.76912200	1.40277800	C	3.98187200	3.78057100	1.40331700
H	4.00876700	3.74527800	-2.49036100	H	3.99649300	3.75747600	-2.49090800
H	4.00876700	3.74527800	2.49036100	H	3.99649300	3.75747600	2.49090800
C	1.43155200	-1.20003400	-0.72285900	C	1.43080500	-1.20070100	-0.72302500
H	1.10009000	0.00000000	2.44616100	H	1.10016500	0.00000000	2.44611500
H	2.19114700	2.10089000	2.49224500	H	2.18778800	2.10366800	2.49238800
H	1.10009000	0.00000000	-2.44616100	H	1.10016500	0.00000000	-2.44611500
C	6.23732400	-4.77210900	1.40413900	C	6.21926700	-4.79445300	1.40555200
C	5.07029500	-4.37175600	0.72186300	C	5.05879700	-4.38773800	0.72301900
C	3.99346900	-3.76912200	1.40277800	C	3.98187200	-3.78057100	1.40331700
C	5.07029500	-4.37175600	-0.72186300	C	5.05879700	-4.38773800	-0.72301900
C	3.99346900	-3.76912200	-1.40277800	C	3.98187200	-3.78057100	-1.40331700
C	6.23732400	-4.77210900	-1.40413900	C	6.21926700	-4.79445300	-1.40555200
H	6.24360400	-4.74309200	2.49160100	H	6.22454400	-4.76866100	2.49299400
H	6.24360400	-4.74309200	-2.49160100	H	6.22454400	-4.76866100	-2.49299400
C	3.02753400	-3.04116400	0.72126000	C	3.02018500	-3.04803700	0.72151500
C	3.02753400	-3.04116400	-0.72126000	C	3.02018500	-3.04803700	-0.72151500
C	2.15665800	-2.12605400	-1.40493100	C	2.15309600	-2.12875300	-1.40506500
C	2.15665800	-2.12605400	1.40493100	C	2.15309600	-2.12875300	1.40506500
H	4.00876700	-3.74527800	-2.49036100	H	3.99649300	-3.75747600	-2.49090800
H	4.00876700	-3.74527800	2.49036100	H	3.99649300	-3.75747600	2.49090800
H	2.19114700	-2.10089000	-2.49224500	H	2.18778800	-2.10366800	-2.49238800
H	2.19114700	-2.10089000	2.49224500	H	2.18778800	-2.10366800	2.49238800
C	6.23732400	4.77210900	-1.40413900	C	6.21926700	4.79445300	-1.40555200
C	7.43308600	4.97411600	-0.72451200	C	7.42375200	4.99550100	-0.72621800
C	7.43308600	4.97411600	0.72451200	C	7.42375200	4.99550100	0.72621800
C	6.23732400	4.77210900	1.40413900	C	6.21926700	4.79445300	1.40555200
H	6.24360400	4.74309200	-2.49160100	H	6.22454400	4.76866100	-2.49299400
H	6.24360400	4.74309200	2.49160100	H	6.22454400	4.76866100	2.49299400

**Dcyc15_R**				**Dcyc15_U**			
C	13.57027000	2.37356400	0.73405900	C	13.55150800	2.37068200	0.72852900
C	13.57027000	2.37356400	-0.73405900	C	13.55150800	2.37068200	-0.72852900
C	6.10869200	-4.96148200	1.40454300	C	6.09388100	-4.98337100	1.40559200
C	4.97420200	-4.49032700	0.72262200	C	4.96172400	-4.50397100	0.72306800
C	3.92354300	-3.83596900	1.40361700	C	3.91521500	-3.84642700	1.40331000
C	4.97420200	-4.49032700	-0.72262200	C	4.96172400	-4.50397100	-0.72306800
C	3.92354300	-3.83596900	-1.40361700	C	3.91521500	-3.84642700	-1.40331000
C	2.98764700	-3.07534500	-0.72238700	C	2.98033500	-3.08024700	-0.72142500
C	2.98764700	-3.07534500	0.72238700	C	2.98033500	-3.08024700	0.72142500
C	7.28940000	-5.25766400	0.72445600	C	7.27850500	-5.27943900	0.72641900
C	6.10869200	-4.96148200	-1.40454300	C	6.09388100	-4.98337100	-1.40559200
H	3.93817400	-3.81536400	-2.49129200	H	3.92928200	-3.82559100	-2.49097300
C	2.13820000	-2.13762600	-1.40593800	C	2.13521100	-2.14100000	-1.40522500
C	2.13820000	-2.13762600	1.40593800	C	2.13521100	-2.14100000	1.40522500
H	2.17224100	-2.11383300	-2.49330200	H	2.16858800	-2.11782400	-2.49263400
H	2.17224100	-2.11383300	2.49330200	H	2.16858800	-2.11782400	2.49263400
C	7.28940000	-5.25766400	-0.72445600	C	7.27850500	-5.27943900	-0.72641900
H	6.11644200	-4.93564200	2.49207700	H	6.10003600	-4.96052500	2.49311800
H	3.93817400	-3.81536400	2.49129200	H	3.92928200	-3.82559100	2.49097300
H	6.11644200	-4.93564200	-2.49207700	H	6.10003600	-4.96052500	-2.49311800
C	1.42823900	-1.20199600	0.72389000	C	1.42721300	-1.20242500	0.72301700
C	1.42823900	-1.20199600	-0.72389000	C	1.42721300	-1.20242500	-0.72301700
C	14.04908300	-1.21931600	1.40434400	C	13.99301200	-1.22108900	1.40810600
C	13.57027000	-2.37356400	0.73405900	C	13.55150800	-2.37068200	0.72852900
C	12.90671400	-3.37646300	1.40711500	C	12.87016200	-3.39018400	1.40847400

C	13.57027000	-2.37356400	-0.73405900	C	13.55150800	-2.37068200	-0.72852900
C	12.90671400	-3.37646300	-1.40711500	C	12.87016200	-3.39018400	-1.40847400
C	11.96460400	-4.21646900	-0.73094400	C	11.95702400	-4.22187800	-0.72907500
C	11.96460400	-4.21646900	0.73094400	C	11.95702400	-4.22187800	0.72907500
C	14.04908300	-1.21931600	-1.40434400	C	13.99301200	-1.22108900	-1.40810600
C	10.92769000	-4.81304000	-1.40767100	C	10.90440000	-4.84169400	-1.40869100
C	10.92769000	-4.81304000	1.40767100	C	10.90440000	-4.84169400	1.40869100
H	14.05831600	-1.22193100	2.49221900	H	13.97233700	-1.21587300	2.49544900
H	14.05831600	-1.22193100	-2.49221900	H	13.97233700	-1.21587300	-2.49544900
C	9.72930600	-5.20077800	0.72746700	C	9.72403700	-5.21992900	0.72871000
C	9.72930600	-5.20077800	-0.72746700	C	9.72403700	-5.21992900	-0.72871000
C	8.53394000	-5.33506300	-1.40622200	C	8.51492700	-5.36611900	-1.40788000
C	8.53394000	-5.33506300	1.40622200	C	8.51492700	-5.36611900	1.40788000
H	12.90073900	-3.37247400	-2.49498300	H	12.85527200	-3.37699000	-2.49583600
H	12.90073900	-3.37247400	2.49498300	H	12.85527200	-3.37699000	2.49583600
H	10.91747300	-4.79277800	2.49527700	H	10.89655200	-4.82412400	2.49607500
H	10.91747300	-4.79277800	-2.49527700	H	10.89655200	-4.82412400	-2.49607500
H	8.53215600	-5.30726100	-2.49371400	H	8.51359700	-5.34511100	-2.49531500
H	8.53215600	-5.30726100	2.49371400	H	8.51359700	-5.34511100	2.49531500
C	8.53394000	5.33506300	1.40622200	C	8.51492700	5.36611900	1.40788000
C	9.72930600	5.20077800	0.72746700	C	9.72403700	5.21992900	0.72871000
C	10.92769000	4.81304000	1.40767100	C	10.90440000	4.84169400	1.40869100
C	9.72930600	5.20077800	-0.72746700	C	9.72403700	5.21992900	-0.72871000
C	10.92769000	4.81304000	-1.40767100	C	10.90440000	4.84169400	-1.40869100
C	11.96460400	4.21646900	-0.73094400	C	11.95702400	4.22187800	-0.72907500
C	11.96460400	4.21646900	0.73094400	C	11.95702400	4.22187800	0.72907500
C	7.28940000	5.25766400	0.72445600	C	7.27850500	5.27943900	0.72641900
C	8.53394000	5.33506300	-1.40622200	C	8.51492700	5.36611900	-1.40788000
H	10.91747300	4.79277800	-2.49527700	H	10.89655200	4.82412400	-2.49607500
C	12.90671400	3.37646300	-1.40711500	C	12.87016200	3.39018400	-1.40847400
C	12.90671400	3.37646300	1.40711500	C	12.87016200	3.39018400	1.40847400
H	12.90073900	3.37247400	-2.49498300	H	12.85527200	3.37699000	-2.49583600
H	12.90073900	3.37247400	2.49498300	H	12.85527200	3.37699000	2.49583600
C	7.28940000	5.25766400	-0.72445600	C	7.27850500	5.27943900	-0.72641900
H	8.53215600	5.30726100	2.49371400	H	8.51359700	5.34511100	2.49531500
H	10.91747300	4.79277800	2.49527700	H	10.89655200	4.82412400	2.49607500
H	8.53215600	5.30726100	-2.49371400	H	8.51359700	5.34511100	-2.49531500
C	0.79108900	0.00000000	1.39748600	C	0.79125500	0.00000000	1.39713300
C	1.42823900	1.20199600	0.72389000	C	1.42721300	1.20242500	0.72301700
C	2.13820000	2.13762600	1.40593800	C	2.13521100	2.14100000	1.40522500
C	1.42823900	1.20199600	-0.72389000	C	1.42721300	1.20242500	-0.72301700
C	2.13820000	2.13762600	-1.40593800	C	2.13521100	2.14100000	-1.40522500
C	2.98764700	3.07534500	-0.72238700	C	2.98033500	3.08024700	-0.72142500
C	2.98764700	3.07534500	0.72238700	C	2.98033500	3.08024700	0.72142500
C	0.79108900	0.00000000	-1.39748600	C	0.79125500	0.00000000	-1.39713300
C	3.92354300	3.83596900	-1.40361700	C	3.91521500	3.84642700	-1.40331000
C	3.92354300	3.83596900	1.40361700	C	3.91521500	3.84642700	1.40331000
H	1.10034300	0.00000000	2.44661300	H	1.10109100	0.00000000	2.44613100
H	1.10034300	0.00000000	-2.44661300	H	1.10109100	0.00000000	-2.44613100
C	4.97420200	4.49032700	0.72262200	C	4.96172400	4.50397100	0.72306800
C	4.97420200	4.49032700	-0.72262200	C	4.96172400	4.50397100	-0.72306800
C	6.10869200	4.96148200	-1.40454300	C	6.09388100	4.98337100	-1.40559200
C	6.10869200	4.96148200	1.40454300	C	6.09388100	4.98337100	1.40559200
H	2.17224100	2.11383300	-2.49330200	H	2.16858800	2.11782400	-2.49263400
H	2.17224100	2.11383300	2.49330200	H	2.16858800	2.11782400	2.49263400
H	3.93817400	3.81536400	2.49129200	H	3.92928200	3.82559100	2.49097300
H	3.93817400	3.81536400	-2.49129200	H	3.92928200	3.82559100	-2.49097300
H	6.11644200	4.93564200	-2.49207700	H	6.10003600	4.96052500	-2.49311800
H	6.11644200	4.93564200	2.49207700	H	6.10003600	4.96052500	2.49311800
C	14.04908300	1.21931600	-1.40434400	C	13.99301200	1.22108900	-1.40810600

C	14.15746500	0.00000000	-0.73538700	C	14.13316500	0.00000000	-0.72818200
C	14.15746500	0.00000000	0.73538700	C	14.13316500	0.00000000	0.72818200
C	14.04908300	1.21931600	1.40434400	C	13.99301200	1.22108900	1.40810600
H	14.05831600	1.22193100	-2.49221900	H	13.97233700	1.21587300	-2.49544900
H	14.05831600	1.22193100	2.49221900	H	13.97233700	1.21587300	2.49544900
C	-1.42823900	1.20199600	0.72389000	C	-1.42721300	1.20242500	0.72301700
C	-1.42823900	1.20199600	-0.72389000	C	-1.42721300	1.20242500	-0.72301700
C	-10.92769000	-4.81304000	1.40767100	C	-10.90440000	-4.84169400	1.40869100
C	-11.96460400	-4.21646900	0.73094400	C	-11.95702400	-4.22187800	0.72907500
C	-12.90671400	-3.37646300	1.40711500	C	-12.87016200	-3.39018400	1.40847400
C	-11.96460400	-4.21646900	-0.73094400	C	-11.95702400	-4.22187800	-0.72907500
C	-12.90671400	-3.37646300	-1.40711500	C	-12.87016200	-3.39018400	-1.40847400
C	-13.57027000	-2.37356400	-0.73405900	C	-13.55150800	-2.37068200	-0.72852900
C	-13.57027000	-2.37356400	0.73405900	C	-13.55150800	-2.37068200	0.72852900
C	-9.72930600	-5.20077800	0.72746700	C	-9.72403700	-5.21992900	0.72871000
C	-10.92769000	-4.81304000	-1.40767100	C	-10.90440000	-4.84169400	-1.40869100
H	-12.90073900	-3.37247400	-2.49498300	H	-12.85527200	-3.37699000	-2.49583600
C	-14.04908300	-1.21931600	-1.40434400	C	-13.99301200	-1.22108900	-1.40810600
C	-14.04908300	-1.21931600	1.40434400	C	-13.99301200	-1.22108900	1.40810600
H	-14.05831600	-1.22193100	-2.49221900	H	-13.97233700	-1.21587300	-2.49544900
H	-14.05831600	-1.22193100	2.49221900	H	-13.97233700	-1.21587300	2.49544900
C	-9.72930600	-5.20077800	-0.72746700	C	-9.72403700	-5.21992900	-0.72871000
H	-10.91747300	-4.79277800	2.49527700	H	-10.89655200	-4.82412400	2.49607500
H	-12.90073900	-3.37247400	2.49498300	H	-12.85527200	-3.37699000	2.49583600
H	-10.91747300	-4.79277800	-2.49527700	H	-10.89655200	-4.82412400	-2.49607500
C	-14.15746500	0.00000000	0.73538700	C	-14.13316500	0.00000000	0.72818200
C	-14.15746500	0.00000000	-0.73538700	C	-14.13316500	0.00000000	-0.72818200
C	-2.13820000	-2.13762600	1.40593800	C	-2.13521100	-2.14100000	1.40522500
C	-2.98764700	-3.07534500	0.72238700	C	-2.98033500	-3.08024700	0.72142500
C	-3.92354300	-3.83596900	1.40361700	C	-3.91521500	-3.84642700	1.40331000
C	-2.98764700	-3.07534500	-0.72238700	C	-2.98033500	-3.08024700	-0.72142500
C	-3.92354300	-3.83596900	-1.40361700	C	-3.91521500	-3.84642700	-1.40331000
C	-4.97420200	-4.49032700	-0.72262200	C	-4.96172400	-4.50397100	-0.72306800
C	-4.97420200	-4.49032700	0.72262200	C	-4.96172400	-4.50397100	0.72306800
C	-2.13820000	-2.13762600	-1.40593800	C	-2.13521100	-2.14100000	-1.40522500
C	-6.10869200	-4.96148200	-1.40454300	C	-6.09388100	-4.98337100	-1.40559200
C	-6.10869200	-4.96148200	1.40454300	C	-6.09388100	-4.98337100	1.40559200
H	-2.17224100	-2.11383300	2.49330200	H	-2.16858800	-2.11782400	2.49263400
H	-2.17224100	-2.11383300	-2.49330200	H	-2.16858800	-2.11782400	-2.49263400
C	-7.28940000	-5.25766400	0.72445600	C	-7.27850500	-5.27943900	0.72641900
C	-7.28940000	-5.25766400	-0.72445600	C	-7.27850500	-5.27943900	-0.72641900
C	-8.53394000	-5.33506300	-1.40622200	C	-8.51492700	-5.36611900	-1.40788000
C	-8.53394000	-5.33506300	1.40622200	C	-8.51492700	-5.36611900	1.40788000
H	-3.93817400	-3.81536400	-2.49129200	H	-3.92928200	-3.82559100	-2.49097300
H	-3.93817400	-3.81536400	2.49129200	H	-3.92928200	-3.82559100	2.49097300
H	-6.11644200	-4.93564200	2.49207700	H	-6.10003600	-4.96052500	2.49311800
H	-6.11644200	-4.93564200	-2.49207700	H	-6.10003600	-4.96052500	-2.49311800
H	-8.53215600	-5.30726100	-2.49371400	H	-8.51359700	-5.34511100	-2.49531500
H	-8.53215600	-5.30726100	2.49371400	H	-8.51359700	-5.34511100	2.49531500
C	-6.10869200	4.96148200	1.40454300	C	-6.09388100	4.98337100	1.40559200
C	-4.97420200	4.49032700	0.72262200	C	-4.96172400	4.50397100	0.72306800
C	-3.92354300	3.83596900	1.40361700	C	-3.91521500	3.84642700	1.40331000
C	-4.97420200	4.49032700	-0.72262200	C	-4.96172400	4.50397100	-0.72306800
C	-3.92354300	3.83596900	-1.40361700	C	-3.91521500	3.84642700	-1.40331000
C	-2.98764700	3.07534500	-0.72238700	C	-2.98033500	3.08024700	-0.72142500
C	-2.98764700	3.07534500	0.72238700	C	-2.98033500	3.08024700	0.72142500
C	-7.28940000	5.25766400	0.72445600	C	-7.27850500	5.27943900	0.72641900
C	-6.10869200	4.96148200	-1.40454300	C	-6.09388100	4.98337100	-1.40559200
H	-3.93817400	3.81536400	-2.49129200	H	-3.92928200	3.82559100	-2.49097300
C	-2.13820000	2.13762600	-1.40593800	C	-2.13521100	2.14100000	-1.40522500

C	-2.13820000	2.13762600	1.40593800	C	-2.13521100	2.14100000	1.40522500
H	-2.17224100	2.11383300	-2.49330200	H	-2.16858800	2.11782400	-2.49263400
H	-2.17224100	2.11383300	2.49330200	H	-2.16858800	2.11782400	2.49263400
C	-7.28940000	5.25766400	-0.72445600	C	-7.27850500	5.27943900	-0.72641900
H	-6.11644200	4.93564200	2.49207700	H	-6.10003600	4.96052500	2.49311800
H	-3.93817400	3.81536400	2.49129200	H	-3.92928200	3.82559100	2.49097300
H	-6.11644200	4.93564200	-2.49207700	H	-6.10003600	4.96052500	-2.49311800
C	-14.04908300	1.21931600	1.40434400	C	-13.99301200	1.22108900	1.40810600
C	-13.57027000	2.37356400	0.73405900	C	-13.55150800	2.37068200	0.72852900
C	-12.90671400	3.37646300	1.40711500	C	-12.87016200	3.39018400	1.40847400
C	-13.57027000	2.37356400	-0.73405900	C	-13.55150800	2.37068200	-0.72852900
C	-12.90671400	3.37646300	-1.40711500	C	-12.87016200	3.39018400	-1.40847400
C	-11.96460400	4.21646900	-0.73094400	C	-11.95702400	4.22187800	-0.72907500
C	-11.96460400	4.21646900	0.73094400	C	-11.95702400	4.22187800	0.72907500
C	-14.04908300	1.21931600	-1.40434400	C	-13.99301200	1.22108900	-1.40810600
C	-10.92769000	4.81304000	-1.40767100	C	-10.90440000	4.84169400	-1.40869100
C	-10.92769000	4.81304000	1.40767100	C	-10.90440000	4.84169400	1.40869100
H	-14.05831600	1.22193100	2.49221900	H	-13.97233700	1.21587300	2.49544900
H	-14.05831600	1.22193100	-2.49221900	H	-13.97233700	1.21587300	-2.49544900
C	-9.72930600	5.20077800	0.72746700	C	-9.72403700	5.21992900	0.72871000
C	-9.72930600	5.20077800	-0.72746700	C	-9.72403700	5.21992900	-0.72871000
C	-8.53394000	5.33506300	-1.40622200	C	-8.51492700	5.36611900	-1.40788000
C	-8.53394000	5.33506300	1.40622200	C	-8.51492700	5.36611900	1.40788000
H	-12.90073900	3.37247400	-2.49498300	H	-12.85527200	3.37699000	-2.49583600
H	-12.90073900	3.37247400	2.49498300	H	-12.85527200	3.37699000	2.49583600
H	-10.91747300	4.79277800	2.49527700	H	-10.89655200	4.82412400	2.49607500
H	-10.91747300	4.79277800	-2.49527700	H	-10.89655200	4.82412400	-2.49607500
H	-8.53215600	5.30726100	-2.49371400	H	-8.51359700	5.34511100	-2.49531500
H	-8.53215600	5.30726100	2.49371400	H	-8.51359700	5.34511100	2.49531500
C	-0.79108900	0.00000000	-1.39748600	C	-0.79125500	0.00000000	-1.39713300
C	-1.42823900	-1.20199600	-0.72389000	C	-1.42721300	-1.20242500	-0.72301700
C	-1.42823900	-1.20199600	0.72389000	C	-1.42721300	-1.20242500	0.72301700
C	-0.79108900	0.00000000	1.39748600	C	-0.79125500	0.00000000	1.39713300
H	-1.10034300	0.00000000	-2.44661300	H	-1.10109100	0.00000000	-2.44613100
H	-1.10034300	0.00000000	2.44661300	H	-1.10109100	0.00000000	2.44613100
**Dcyc16_R**				**Dcyc16_U**			
C	-11.93369700	4.96740800	0.73000600	C	-11.92642900	4.98256500	0.72920200
C	-11.93369700	4.96740800	-0.73000600	C	-11.92642900	4.98256500	-0.72920200
C	-0.79165400	0.00000000	1.39814100	C	-0.79197300	0.00000000	1.39726300
C	-1.42518800	-1.20322600	0.72456000	C	-1.42414100	-1.20380700	0.72297200
C	-2.12175300	-2.14766900	1.40660000	C	-2.11936500	-2.15164600	1.40526200
C	-1.42518800	-1.20322600	-0.72456000	C	-1.42414100	-1.20380700	-0.72297200
C	-2.12175300	-2.14766900	-1.40660000	C	-2.11936500	-2.15164600	-1.40526200
C	-2.95470000	-3.10168300	-0.72347400	C	-2.94551100	-3.10741200	-0.72148600
C	-2.95470000	-3.10168300	0.72347400	C	-2.94551100	-3.10741200	0.72148600
C	-1.42518800	1.20322600	0.72456000	C	-1.42414100	1.20380700	0.72297200
C	-0.79165400	0.00000000	-1.39814100	C	-0.79197300	0.00000000	-1.39726300
H	-2.15355600	-2.12634000	-2.49407300	H	-2.15109900	-2.13055600	-2.49276500
C	-3.86420700	-3.88987100	-1.40444000	C	-3.85569000	-3.90252500	-1.40334400
C	-3.86420700	-3.88987100	1.40444000	C	-3.85569000	-3.90252500	1.40334400
H	-3.87835200	-3.87147800	-2.49212200	H	-3.86940500	-3.88368200	-2.49104200
H	-3.87835200	-3.87147800	2.49212200	H	-3.86940500	-3.88368200	2.49104200
C	-1.42518800	1.20322600	-0.72456000	C	-1.42414100	1.20380700	-0.72297200
H	-1.10110800	0.00000000	2.44720600	H	-1.10193300	0.00000000	2.44622500
H	-2.15355600	-2.12634000	2.49407300	H	-2.15109900	-2.13055600	2.49276500
H	-1.10110800	0.00000000	-2.44720600	H	-1.10193300	0.00000000	-2.44622500
C	-4.88992000	-4.58772700	0.72357200	C	-4.87376700	-4.60298700	0.72313600
C	-4.88992000	-4.58772700	-0.72357200	C	-4.87376700	-4.60298700	-0.72313600

C	-8.38417900	5.67292800	1.40630000	C	-8.36386800	5.70977300	1.40781900
C	-7.15558500	5.49314700	0.72484100	C	-7.13936300	5.51981800	0.72651100
C	-5.99300700	5.11877700	1.40505400	C	-5.97768600	5.14390900	1.40558300
C	-7.15558500	5.49314700	-0.72484100	C	-7.13936300	5.51981800	-0.72651100
C	-5.99300700	5.11877700	-1.40505400	C	-5.97768600	5.14390900	-1.40558300
C	-4.88992000	4.58772700	-0.72357200	C	-4.87376700	4.60298700	-0.72313600
C	-4.88992000	4.58772700	0.72357200	C	-4.87376700	4.60298700	0.72313600
C	-8.38417900	5.67292800	-1.40630000	C	-8.36386800	5.70977300	-1.40781900
C	-3.86420700	3.88987100	-1.40444000	C	-3.85569000	3.90252500	-1.40334400
C	-3.86420700	3.88987100	1.40444000	C	-3.85569000	3.90252500	1.40334400
H	-8.38503900	5.64683400	2.49381600	H	-8.36435400	5.69072300	2.49529800
H	-8.38503900	5.64683400	-2.49381600	H	-8.36435400	5.69072300	-2.49529800
C	-2.95470000	3.10168300	0.72347400	C	-2.94551100	3.10741200	0.72148600
C	-2.95470000	3.10168300	-0.72347400	C	-2.94551100	3.10741200	-0.72148600
C	-2.12175300	2.14766900	-1.40660000	C	-2.11936500	2.15164600	-1.40526200
C	-2.12175300	2.14766900	1.40660000	C	-2.11936500	2.15164600	1.40526200
H	-6.00145900	5.09604500	-2.49262800	H	-5.98448700	5.12344500	-2.49315500
H	-6.00145900	5.09604500	2.49262800	H	-5.98448700	5.12344500	2.49315500
H	-3.87835200	3.87147800	2.49212200	H	-3.86940500	3.88368200	2.49104200
H	-3.87835200	3.87147800	-2.49212200	H	-3.86940500	3.88368200	-2.49104200
H	-2.15355600	2.12634000	-2.49407300	H	-2.15109900	2.13055600	-2.49276500
H	-2.15355600	2.12634000	2.49407300	H	-2.15109900	2.13055600	2.49276500
C	-15.07893700	0.00000000	1.40409500	C	-15.01270100	0.00000000	1.40769700
C	-14.90045500	1.22380600	0.73500900	C	-14.86878100	1.22100100	0.72785500
C	-14.52151000	2.37504800	1.40598300	C	-14.46806500	2.38265300	1.40787800
C	-14.90045500	1.22380600	-0.73500900	C	-14.86878100	1.22100100	-0.72785500
C	-14.52151000	2.37504800	-1.40598300	C	-14.46806500	2.38265300	-1.40787800
C	-13.82875800	3.42319700	-0.73293600	C	-13.81013500	3.42271800	-0.72849200
C	-13.82875800	3.42319700	0.73293600	C	-13.81013500	3.42271800	0.72849200
C	-14.90045500	-1.22380600	0.73500900	C	-14.86878100	-1.22100100	0.72785500
C	-15.07893700	0.00000000	-1.40409500	C	-15.01270100	0.00000000	-1.40769700
H	-14.52267700	2.37619100	-2.49390500	H	-14.44976000	2.37362300	-2.49526600
C	-12.99175800	4.28025600	-1.40800400	C	-12.95805400	4.30489400	-1.40838200
C	-12.99175800	4.28025600	1.40800400	C	-12.95805400	4.30489400	1.40838200
H	-12.98175500	4.26941800	-2.49578200	H	-12.94662400	4.29059900	-2.49579100
H	-12.98175500	4.26941800	2.49578200	H	-12.94662400	4.29059900	2.49579100
C	-14.90045500	-1.22380600	-0.73500900	C	-14.86878100	-1.22100100	-0.72785500
H	-15.08913600	0.00000000	2.49199900	H	-14.99117100	0.00000000	2.49507300
H	-14.52267700	2.37619100	2.49390500	H	-14.44976000	2.37362300	2.49526600
H	-15.08913600	0.00000000	-2.49199900	H	-14.99117100	0.00000000	-2.49507300
C	-5.99300700	-5.11877700	1.40505400	C	-5.97768600	-5.14390900	1.40558300
C	-10.82177200	-5.41291900	1.40771300	C	-10.79786500	-5.45083100	1.40866300
C	-10.82177200	-5.41291900	-1.40771300	C	-10.79786500	-5.45083100	-1.40866300
C	-11.93369700	-4.96740800	-0.73000600	C	-11.92642900	-4.98256500	-0.72920200
C	-11.93369700	-4.96740800	0.73000600	C	-11.92642900	-4.98256500	0.72920200
C	-5.99300700	-5.11877700	-1.40505400	C	-5.97768600	-5.14390900	-1.40558300
C	-12.99175800	-4.28025600	-1.40800400	C	-12.95805400	-4.30489400	-1.40838200
C	-12.99175800	-4.28025600	1.40800400	C	-12.95805400	-4.30489400	1.40838200
H	-6.00145900	-5.09604500	2.49262800	H	-5.98448700	-5.12344500	2.49315500
H	-6.00145900	-5.09604500	-2.49262800	H	-5.98448700	-5.12344500	-2.49315500
C	-13.82875800	-3.42319700	0.73293600	C	-13.81013500	-3.42271800	0.72849200
C	-13.82875800	-3.42319700	-0.73293600	C	-13.81013500	-3.42271800	-0.72849200
C	-14.52151000	-2.37504800	-1.40598300	C	-14.46806500	-2.38265300	-1.40787800
C	-14.52151000	-2.37504800	1.40598300	C	-14.46806500	-2.38265300	1.40787800
H	-10.81389300	-5.38986400	-2.49528500	H	-10.79287400	-5.43422500	-2.49609300
H	-10.81389300	-5.38986400	2.49528500	H	-10.79287400	-5.43422500	2.49609300
H	-12.98175500	-4.26941800	2.49578200	H	-12.94662400	-4.29059900	2.49579100
H	-12.98175500	-4.26941800	-2.49578200	H	-12.94662400	-4.29059900	-2.49579100
H	-14.52267700	-2.37619100	-2.49390500	H	-14.44976000	-2.37362300	-2.49526600
H	-14.52267700	-2.37619100	2.49390500	H	-14.44976000	-2.37362300	2.49526600

C	-10.82177200	5.41291900	-1.40771300	C	-10.79786500	5.45083100	-1.40866300
C	-9.59086200	5.65796600	-0.72710400	C	-9.58157700	5.68619800	-0.72885100
C	-9.59086200	5.65796600	0.72710400	C	-9.58157700	5.68619800	0.72885100
C	-10.82177200	5.41291900	1.40771300	C	-10.79786500	5.45083100	1.40866300
H	-10.81389300	5.38986400	-2.49528500	H	-10.79287400	5.43422500	-2.49609300
H	-10.81389300	5.38986400	2.49528500	H	-10.79287400	5.43422500	2.49609300
C	-9.59086200	-5.65796600	-0.72710400	C	-9.58157700	-5.68619800	-0.72885100
C	-8.38417900	-5.67292800	-1.40630000	C	-8.36386800	-5.70977300	-1.40781900
C	-7.15558500	-5.49314700	-0.72484100	C	-7.13936300	-5.51981800	-0.72651100
C	-7.15558500	-5.49314700	0.72484100	C	-7.13936300	-5.51981800	0.72651100
C	-8.38417900	-5.67292800	1.40630000	C	-8.36386800	-5.70977300	1.40781900
C	-9.59086200	-5.65796600	0.72710400	C	-9.58157700	-5.68619800	0.72885100
H	-8.38503900	-5.64683400	-2.49381600	H	-8.36435400	-5.69072300	-2.49529800
H	-8.38503900	-5.64683400	2.49381600	H	-8.36435400	-5.69072300	2.49529800
C	4.88992000	4.58772700	0.72357200	C	4.87376700	4.60298700	0.72313600
C	4.88992000	4.58772700	-0.72357200	C	4.87376700	4.60298700	-0.72313600
C	15.07893700	0.00000000	1.40409500	C	15.01270100	0.00000000	1.40769700
C	14.90045500	-1.22380600	0.73500900	C	14.86878100	-1.22100100	0.72785500
C	14.52151000	-2.37504800	1.40598300	C	14.46806500	-2.38265300	1.40787800
C	14.90045500	-1.22380600	-0.73500900	C	14.86878100	-1.22100100	-0.72785500
C	14.52151000	-2.37504800	-1.40598300	C	14.46806500	-2.38265300	-1.40787800
C	13.82875800	-3.42319700	-0.73293600	C	13.81013500	-3.42271800	-0.72849200
C	13.82875800	-3.42319700	0.73293600	C	13.81013500	-3.42271800	0.72849200
C	14.90045500	1.22380600	0.73500900	C	14.86878100	1.22100100	0.72785500
C	15.07893700	0.00000000	-1.40409500	C	15.01270100	0.00000000	-1.40769700
H	14.52267700	-2.37619100	-2.49390500	H	14.44976000	-2.37362300	-2.49526600
C	12.99175800	-4.28025600	-1.40800400	C	12.95805400	-4.30489400	-1.40838200
C	12.99175800	-4.28025600	1.40800400	C	12.95805400	-4.30489400	1.40838200
H	12.98175500	-4.26941800	-2.49578200	H	12.94662400	-4.29059900	-2.49579100
H	12.98175500	-4.26941800	2.49578200	H	12.94662400	-4.29059900	2.49579100
C	14.90045500	1.22380600	-0.73500900	C	14.86878100	1.22100100	-0.72785500
H	15.08913600	0.00000000	2.49199900	H	14.99117100	0.00000000	2.49507300
H	14.52267700	-2.37619100	2.49390500	H	14.44976000	-2.37362300	2.49526600
H	15.08913600	0.00000000	-2.49199900	H	14.99117100	0.00000000	-2.49507300
C	11.93369700	-4.96740800	0.73000600	C	11.92642900	-4.98256500	0.72920200
C	11.93369700	-4.96740800	-0.73000600	C	11.92642900	-4.98256500	-0.72920200
C	8.38417900	5.67292800	1.40630000	C	8.36386800	5.70977300	1.40781900
C	9.59086200	5.65796600	0.72710400	C	9.58157700	5.68619800	0.72885100
C	10.82177200	5.41291900	1.40771300	C	10.79786500	5.45083100	1.40866300
C	9.59086200	5.65796600	-0.72710400	C	9.58157700	5.68619800	-0.72885100
C	10.82177200	5.41291900	-1.40771300	C	10.79786500	5.45083100	-1.40866300
C	11.93369700	4.96740800	-0.73000600	C	11.92642900	4.98256500	-0.72920200
C	11.93369700	4.96740800	0.73000600	C	11.92642900	4.98256500	0.72920200
C	8.38417900	5.67292800	-1.40630000	C	8.36386800	5.70977300	-1.40781900
C	12.99175800	4.28025600	-1.40800400	C	12.95805400	4.30489400	-1.40838200
C	12.99175800	4.28025600	1.40800400	C	12.95805400	4.30489400	1.40838200
H	8.38503900	5.64683400	2.49381600	H	8.36435400	5.69072300	2.49529800
H	8.38503900	5.64683400	-2.49381600	H	8.36435400	5.69072300	-2.49529800
C	13.82875800	3.42319700	0.73293600	C	13.81013500	3.42271800	0.72849200
C	13.82875800	3.42319700	-0.73293600	C	13.81013500	3.42271800	-0.72849200
C	14.52151000	2.37504800	-1.40598300	C	14.46806500	2.38265300	-1.40787800
C	14.52151000	2.37504800	1.40598300	C	14.46806500	2.38265300	1.40787800
H	10.81389300	5.38986400	-2.49528500	H	10.79287400	5.43422500	-2.49609300
H	10.81389300	5.38986400	2.49528500	H	10.79287400	5.43422500	2.49609300
H	12.98175500	4.26941800	2.49578200	H	12.94662400	4.29059900	2.49579100
H	12.98175500	4.26941800	-2.49578200	H	12.94662400	4.29059900	-2.49579100
H	14.52267700	2.37619100	-2.49390500	H	14.44976000	2.37362300	-2.49526600
H	14.52267700	2.37619100	2.49390500	H	14.44976000	2.37362300	2.49526600
C	0.79165400	0.00000000	1.39814100	C	0.79197300	0.00000000	1.39726300
C	1.42518800	1.20322600	0.72456000	C	1.42414100	1.20380700	0.72297200

C	2.12175300	2.14766900	1.40660000	C	2.11936500	2.15164600	1.40526200
C	1.42518800	1.20322600	-0.72456000	C	1.42414100	1.20380700	-0.72297200
C	2.12175300	2.14766900	-1.40660000	C	2.11936500	2.15164600	-1.40526200
C	2.95470000	3.10168300	-0.72347400	C	2.94551100	3.10741200	-0.72148600
C	2.95470000	3.10168300	0.72347400	C	2.94551100	3.10741200	0.72148600
C	1.42518800	-1.20322600	0.72456000	C	1.42414100	-1.20380700	0.72297200
C	0.79165400	0.00000000	-1.39814100	C	0.79197300	0.00000000	-1.39726300
H	2.15355600	2.12634000	-2.49407300	H	2.15109900	2.13055600	-2.49276500
C	3.86420700	3.88987100	-1.40444000	C	3.85569000	3.90252500	-1.40334400
C	3.86420700	3.88987100	1.40444000	C	3.85569000	3.90252500	1.40334400
H	3.87835200	3.87147800	-2.49212200	H	3.86940500	3.88368200	-2.49104200
H	3.87835200	3.87147800	2.49212200	H	3.86940500	3.88368200	2.49104200
C	1.42518800	-1.20322600	-0.72456000	C	1.42414100	-1.20380700	-0.72297200
H	1.10110800	0.00000000	2.44720600	H	1.10193300	0.00000000	2.44622500
H	2.15355600	2.12634000	2.49407300	H	2.15109900	2.13055600	2.49276500
H	1.10110800	0.00000000	-2.44720600	H	1.10193300	0.00000000	-2.44622500
C	10.82177200	-5.41291900	1.40771300	C	10.79786500	-5.45083100	1.40866300
C	5.99300700	-5.11877700	1.40505400	C	5.97768600	-5.14390900	1.40558300
C	5.99300700	-5.11877700	-1.40505400	C	5.97768600	-5.14390900	-1.40558300
C	4.88992000	-4.58772700	-0.72357200	C	4.87376700	-4.60298700	-0.72313600
C	4.88992000	-4.58772700	0.72357200	C	4.87376700	-4.60298700	0.72313600
C	10.82177200	-5.41291900	-1.40771300	C	10.79786500	-5.45083100	-1.40866300
C	3.86420700	-3.88987100	-1.40444000	C	3.85569000	-3.90252500	-1.40334400
C	3.86420700	-3.88987100	1.40444000	C	3.85569000	-3.90252500	1.40334400
H	10.81389300	-5.38986400	2.49528500	H	10.79287400	-5.43422500	2.49609300
H	10.81389300	-5.38986400	-2.49528500	H	10.79287400	-5.43422500	-2.49609300
C	2.95470000	-3.10168300	0.72347400	C	2.94551100	-3.10741200	0.72148600
C	2.95470000	-3.10168300	-0.72347400	C	2.94551100	-3.10741200	-0.72148600
C	2.12175300	-2.14766900	-1.40660000	C	2.11936500	-2.15164600	-1.40526200
C	2.12175300	-2.14766900	1.40660000	C	2.11936500	-2.15164600	1.40526200
H	6.00145900	-5.09604500	-2.49262800	H	5.98448700	-5.12344500	-2.49315500
H	6.00145900	-5.09604500	2.49262800	H	5.98448700	-5.12344500	2.49315500
H	3.87835200	-3.87147800	2.49212200	H	3.86940500	-3.88368200	2.49104200
H	3.87835200	-3.87147800	-2.49212200	H	3.86940500	-3.88368200	-2.49104200
H	2.15355600	-2.12634000	-2.49407300	H	2.15109900	-2.13055600	-2.49276500
H	2.15355600	-2.12634000	2.49407300	H	2.15109900	-2.13055600	2.49276500
C	5.99300700	5.11877700	-1.40505400	C	5.97768600	5.14390900	-1.40558300
C	7.15558500	5.49314700	-0.72484100	C	7.13936300	5.51981800	-0.72651100
C	7.15558500	5.49314700	0.72484100	C	7.13936300	5.51981800	0.72651100
C	5.99300700	5.11877700	1.40505400	C	5.97768600	5.14390900	1.40558300
H	6.00145900	5.09604500	-2.49262800	H	5.98448700	5.12344500	-2.49315500
H	6.00145900	5.09604500	2.49262800	H	5.98448700	5.12344500	2.49315500
C	7.15558500	-5.49314700	-0.72484100	C	7.13936300	-5.51981800	-0.72651100
C	8.38417900	-5.67292800	-1.40630000	C	8.36386800	-5.70977300	-1.40781900
C	9.59086200	-5.65796600	-0.72710400	C	9.58157700	-5.68619800	-0.72885100
C	9.59086200	-5.65796600	0.72710400	C	9.58157700	-5.68619800	0.72885100
C	8.38417900	-5.67292800	1.40630000	C	8.36386800	-5.70977300	1.40781900
C	7.15558500	-5.49314700	0.72484100	C	7.13936300	-5.51981800	0.72651100
H	8.38503900	-5.64683400	-2.49381600	H	8.36435400	-5.69072300	-2.49529800
H	8.38503900	-5.64683400	2.49381600	H	8.36435400	-5.69072300	2.49529800

**Dcyc17_R**				**Dcyc17_U**			
C	-15.82561100	1.21980300	1.40479400	C	-15.76095500	1.22282000	1.40750300
C	-15.40192600	2.39307600	0.73433600	C	-15.37271000	2.38863100	0.72782500
C	-14.80659600	3.43884800	1.40733000	C	-14.76023900	3.45447300	1.40777600
C	-15.40192600	2.39307600	-0.73433600	C	-15.37271000	2.38863100	-0.72782500
C	-14.80659600	3.43884800	-1.40733000	C	-14.76023900	3.45447300	-1.40777600
C	-13.94848500	4.36165400	-0.73189800	C	-13.93505600	4.36631400	-0.72856800
C	-13.94848500	4.36165400	0.73189800	C	-13.93505600	4.36631400	0.72856800

C	-15.92328600	0.00000000	0.73541200	C	-15.88663300	0.00000000	0.72751200
C	-15.82561100	1.21980300	-1.40479400	C	-15.76095500	1.22282000	-1.40750300
H	-14.80102200	3.43559800	-2.49522200	H	-14.74486100	3.44305000	-2.49520500
C	-12.98720300	5.07390900	-1.40835500	C	-12.95675900	5.10692100	-1.40829900
C	-12.98720300	5.07390900	1.40835500	C	-12.95675900	5.10692100	1.40829900
H	-12.97657600	5.05787400	-2.49605000	H	-12.94837800	5.09259800	-2.49575000
H	-12.97657600	5.05787400	2.49605000	H	-12.94837800	5.09259800	2.49575000
C	-15.92328600	0.00000000	-0.73541200	C	-15.88663300	0.00000000	-0.72751200
H	-15.83237300	1.22152700	2.49272900	H	-15.74050400	1.21835500	2.49491000
H	-14.80102200	3.43559800	2.49522200	H	-14.74486100	3.44305000	2.49520500
H	-15.83237300	1.22152700	-2.49272900	H	-15.74050400	1.21835500	-2.49491000
C	-11.84979000	5.61504000	0.72919000	C	-11.84330900	5.63787900	0.72927800
C	-11.84979000	5.61504000	-0.72919000	C	-11.84330900	5.63787900	-0.72927800
C	-10.69026900	-5.92573600	1.40767800	C	-10.66677600	-5.96868700	1.40857700
C	-11.84979000	-5.61504000	0.72919000	C	-11.84330900	-5.63787900	0.72927800
C	-12.98720300	-5.07390900	1.40835500	C	-12.95675900	-5.10692100	1.40829900
C	-11.84979000	-5.61504000	-0.72919000	C	-11.84330900	-5.63787900	-0.72927800
C	-12.98720300	-5.07390900	-1.40835500	C	-12.95675900	-5.10692100	-1.40829900
C	-13.94848500	-4.36165400	-0.73189800	C	-13.93505600	-4.36631400	-0.72856800
C	-13.94848500	-4.36165400	0.73189800	C	-13.93505600	-4.36631400	0.72856800
C	-10.69026900	-5.92573600	-1.40767800	C	-10.66677600	-5.96868700	-1.40857700
C	-14.80659600	-3.43884800	-1.40733000	C	-14.76023900	-3.45447300	-1.40777600
C	-14.80659600	-3.43884800	1.40733000	C	-14.76023900	-3.45447300	1.40777600
H	-10.68511400	-5.90183600	2.49523200	H	-10.66394900	-5.95325800	2.49604300
H	-10.68511400	-5.90183600	-2.49523200	H	-10.66394900	-5.95325800	-2.49604300
C	-15.40192600	-2.39307600	0.73433600	C	-15.37271000	-2.38863100	0.72782500
C	-15.40192600	-2.39307600	-0.73433600	C	-15.37271000	-2.38863100	-0.72782500
C	-15.82561100	-1.21980300	-1.40479400	C	-15.76095500	-1.22282000	-1.40750300
C	-15.82561100	-1.21980300	1.40479400	C	-15.76095500	-1.22282000	1.40750300
H	-12.97657600	-5.05787400	-2.49605000	H	-12.94837800	-5.09259800	-2.49575000
H	-12.97657600	-5.05787400	2.49605000	H	-12.94837800	-5.09259800	2.49575000
H	-14.80102200	-3.43559800	2.49522200	H	-14.74486100	-3.44305000	2.49520500
H	-14.80102200	-3.43559800	-2.49522200	H	-14.74486100	-3.44305000	-2.49520500
H	-15.83237300	-1.22152700	-2.49272900	H	-15.74050400	-1.21835500	-2.49491000
H	-15.83237300	-1.22152700	2.49272900	H	-15.74050400	-1.21835500	2.49491000
C	-0.79228400	0.00000000	1.39835900	C	-0.79261100	0.00000000	1.39733000
C	-1.42265500	-1.20463300	0.72519800	C	-1.42112500	-1.20517400	0.72287800
C	-2.10791200	-2.15610000	1.40724000	C	-2.10516100	-2.16103900	1.40526100
C	-1.42265500	-1.20463300	-0.72519800	C	-1.42112500	-1.20517400	-0.72287800
C	-2.10791200	-2.15610000	-1.40724000	C	-2.10516100	-2.16103900	-1.40526100
C	-2.92445400	-3.12578600	-0.72427600	C	-2.91433400	-3.13086700	-0.72146900
C	-2.92445400	-3.12578600	0.72427600	C	-2.91433400	-3.13086700	0.72146900
C	-1.42265500	1.20463300	0.72519800	C	-1.42112500	1.20517400	0.72287800
C	-0.79228400	0.00000000	-1.39835900	C	-0.79261100	0.00000000	-1.39733000
H	-2.13899900	-2.13591700	-2.49476100	H	-2.13563200	-2.14152400	-2.49283000
C	-3.81070200	-3.93675100	-1.40511900	C	-3.80295000	-3.95004800	-1.40334000
C	-3.81070200	-3.93675100	1.40511900	C	-3.80295000	-3.95004800	1.40334000
H	-3.82410700	-3.92043200	-2.49284000	H	-3.81616600	-3.93292800	-2.49107300
H	-3.82410700	-3.92043200	2.49284000	H	-3.81616600	-3.93292800	2.49107300
C	-1.42265500	1.20463300	-0.72519800	C	-1.42112500	1.20517400	-0.72287800
H	-1.10139600	0.00000000	2.44752200	H	-1.10260500	0.00000000	2.44628300
H	-2.13899900	-2.13591700	2.49476100	H	-2.13563200	-2.14152400	2.49283000
H	-1.10139600	0.00000000	-2.44752200	H	-1.10260500	0.00000000	-2.44628300
C	-10.69026900	5.92573600	1.40767800	C	-10.66677600	5.96868700	1.40857700
C	-5.88748600	5.25161700	1.40553700	C	-5.87291100	5.27771500	1.40554500
C	-5.88748600	5.25161700	-1.40553700	C	-5.87291100	5.27771500	-1.40554500
C	-4.81369100	4.67129900	-0.72430900	C	-4.79547500	4.68581800	-0.72316600
C	-4.81369100	4.67129900	0.72430900	C	-4.79547500	4.68581800	0.72316600
C	-10.69026900	5.92573600	-1.40767800	C	-10.66677600	5.96868700	-1.40857700
C	-3.81070200	3.93675100	-1.40511900	C	-3.80295000	3.95004800	-1.40334000

C	-3.81070200	3.93675100	1.40511900	C	-3.80295000	3.95004800	1.40334000
H	-10.68511400	5.90183600	2.49523200	H	-10.66394900	5.95325800	2.49604300
H	-10.68511400	5.90183600	-2.49523200	H	-10.66394900	5.95325800	-2.49604300
C	-2.92445400	3.12578600	0.72427600	C	-2.91433400	3.13086700	0.72146900
C	-2.92445400	3.12578600	-0.72427600	C	-2.91433400	3.13086700	-0.72146900
C	-2.10791200	2.15610000	-1.40724000	C	-2.10516100	2.16103900	-1.40526100
C	-2.10791200	2.15610000	1.40724000	C	-2.10516100	2.16103900	1.40526100
H	-5.89629400	5.23143300	-2.49315400	H	-5.88002100	5.25930400	-2.49315600
H	-5.89629400	5.23143300	2.49315400	H	-5.88002100	5.25930400	2.49315600
H	-3.82410700	3.92043200	2.49284000	H	-3.81616600	3.93292800	2.49107300
H	-3.82410700	3.92043200	-2.49284000	H	-3.81616600	3.93292800	-2.49107300
H	-2.13899900	2.13591700	-2.49476100	H	-2.13563200	2.14152400	-2.49283000
H	-2.13899900	2.13591700	2.49476100	H	-2.13563200	2.14152400	2.49283000
C	-8.23810300	-5.95901100	-1.40643700	C	-8.21806400	-5.99785800	-1.40775800
C	-9.44568700	-6.04641200	-0.72683900	C	-9.43331900	-6.07939500	-0.72889900
C	-9.44568700	-6.04641200	0.72683900	C	-9.43331900	-6.07939500	0.72889900
C	-8.23810300	-5.95901100	1.40643700	C	-8.21806400	-5.99785800	1.40775800
H	-8.24089100	-5.93518100	-2.49399200	H	-8.21983100	-5.98070800	-2.49527600
H	-8.24089100	-5.93518100	2.49399200	H	-8.21983100	-5.98070800	2.49527600
C	-7.03009700	5.69326000	-0.72518200	C	-7.01052800	5.72055600	-0.72657900
C	-8.23810300	5.95901100	-1.40643700	C	-8.21806400	5.99785800	-1.40775800
C	-9.44568700	6.04641200	-0.72683900	C	-9.43331900	6.07939500	-0.72889900
C	-9.44568700	6.04641200	0.72683900	C	-9.43331900	6.07939500	0.72889900
C	-8.23810300	5.95901100	1.40643700	C	-8.21806400	5.99785800	1.40775800
C	-7.03009700	5.69326000	0.72518200	C	-7.01052800	5.72055600	0.72657900
H	-8.24089100	5.93518100	-2.49399200	H	-8.21983100	5.98070800	-2.49527600
H	-8.24089100	5.93518100	2.49399200	H	-8.21983100	5.98070800	2.49527600
C	-4.81369100	-4.67129900	-0.72430900	C	-4.79547500	-4.68581800	-0.72316600
C	-5.88748600	-5.25161700	-1.40553700	C	-5.87291100	-5.27771500	-1.40554500
C	-7.03009700	-5.69326000	-0.72518200	C	-7.01052800	-5.72055600	-0.72657900
C	-7.03009700	-5.69326000	0.72518200	C	-7.01052800	-5.72055600	0.72657900
C	-5.88748600	-5.25161700	1.40553700	C	-5.87291100	-5.27771500	1.40554500
C	-4.81369100	-4.67129900	0.72430900	C	-4.79547500	-4.68581800	0.72316600
H	-5.89629400	-5.23143300	-2.49315400	H	-5.88002100	-5.25930400	-2.49315600
H	-5.89629400	-5.23143300	2.49315400	H	-5.88002100	-5.25930400	2.49315600
C	0.79228400	0.00000000	1.39835900	C	0.79261100	0.00000000	1.39733000
C	1.42265500	1.20463300	0.72519800	C	1.42112500	1.20517400	0.72287800
C	2.10791200	2.15610000	1.40724000	C	2.10516100	2.16103900	1.40526100
C	1.42265500	1.20463300	-0.72519800	C	1.42112500	1.20517400	-0.72287800
C	2.10791200	2.15610000	-1.40724000	C	2.10516100	2.16103900	-1.40526100
C	2.92445400	3.12578600	-0.72427600	C	2.91433400	3.13086700	-0.72146900
C	2.92445400	3.12578600	0.72427600	C	2.91433400	3.13086700	0.72146900
C	1.42265500	-1.20463300	0.72519800	C	1.42112500	-1.20517400	0.72287800
C	0.79228400	0.00000000	-1.39835900	C	0.79261100	0.00000000	-1.39733000
H	2.13899900	2.13591700	-2.49476100	H	2.13563200	2.14152400	-2.49283000
C	3.81070200	3.93675100	-1.40511900	C	3.80295000	3.95004800	-1.40334000
C	3.81070200	3.93675100	1.40511900	C	3.80295000	3.95004800	1.40334000
H	3.82410700	3.92043200	-2.49284000	H	3.81616600	3.93292800	-2.49107300
H	3.82410700	3.92043200	2.49284000	H	3.81616600	3.93292800	2.49107300
C	1.42265500	-1.20463300	-0.72519800	C	1.42112500	-1.20517400	-0.72287800
H	1.10139600	0.00000000	2.44752200	H	1.10260500	0.00000000	2.44628300
H	2.13899900	2.13591700	2.49476100	H	2.13563200	2.14152400	2.49283000
H	1.10139600	0.00000000	-2.44752200	H	1.10260500	0.00000000	-2.44628300
C	4.81369100	4.67129900	0.72430900	C	4.79547500	4.68581800	0.72316600
C	4.81369100	4.67129900	-0.72430900	C	4.79547500	4.68581800	-0.72316600
C	8.23810300	-5.95901100	1.40643700	C	8.21806400	-5.99785800	1.40775800
C	7.03009700	-5.69326000	0.72518200	C	7.01052800	-5.72055600	0.72657900
C	5.88748600	-5.25161700	1.40553700	C	5.87291100	-5.27771500	1.40554500
C	7.03009700	-5.69326000	-0.72518200	C	7.01052800	-5.72055600	-0.72657900
C	5.88748600	-5.25161700	-1.40553700	C	5.87291100	-5.27771500	-1.40554500

C	4.81369100	-4.67129900	-0.72430900	C	4.79547500	-4.68581800	-0.72316600
C	4.81369100	-4.67129900	0.72430900	C	4.79547500	-4.68581800	0.72316600
C	8.23810300	-5.95901100	-1.40643700	C	8.21806400	-5.99785800	-1.40775800
C	3.81070200	-3.93675100	-1.40511900	C	3.80295000	-3.95004800	-1.40334000
C	3.81070200	-3.93675100	1.40511900	C	3.80295000	-3.95004800	1.40334000
H	8.24089100	-5.93518100	2.49399200	H	8.21983100	-5.98070800	2.49527600
H	8.24089100	-5.93518100	-2.49399200	H	8.21983100	-5.98070800	-2.49527600
C	2.92445400	-3.12578600	0.72427600	C	2.91433400	-3.13086700	0.72146900
C	2.92445400	-3.12578600	-0.72427600	C	2.91433400	-3.13086700	-0.72146900
C	2.10791200	-2.15610000	-1.40724000	C	2.10516100	-2.16103900	-1.40526100
C	2.10791200	-2.15610000	1.40724000	C	2.10516100	-2.16103900	1.40526100
H	5.89629400	-5.23143300	-2.49315400	H	5.88002100	-5.25930400	-2.49315600
H	5.89629400	-5.23143300	2.49315400	H	5.88002100	-5.25930400	2.49315600
H	3.82410700	-3.92043200	2.49284000	H	3.81616600	-3.93292800	2.49107300
H	3.82410700	-3.92043200	-2.49284000	H	3.81616600	-3.93292800	-2.49107300
H	2.13899900	-2.13591700	-2.49476100	H	2.13563200	-2.14152400	-2.49283000
H	2.13899900	-2.13591700	2.49476100	H	2.13563200	-2.14152400	2.49283000
C	15.82561100	1.21980300	1.40479400	C	15.76095500	1.22282000	1.40750300
C	15.92328600	0.00000000	0.73541200	C	15.88663300	0.00000000	0.72751200
C	15.82561100	-1.21980300	1.40479400	C	15.76095500	-1.22282000	1.40750300
C	15.92328600	0.00000000	-0.73541200	C	15.88663300	0.00000000	-0.72751200
C	15.82561100	-1.21980300	-1.40479400	C	15.76095500	-1.22282000	-1.40750300
C	15.40192600	-2.39307600	-0.73433600	C	15.37271000	-2.38863100	-0.72782500
C	15.40192600	-2.39307600	0.73433600	C	15.37271000	-2.38863100	0.72782500
C	15.40192600	2.39307600	0.73433600	C	15.37271000	2.38863100	0.72782500
C	15.82561100	1.21980300	-1.40479400	C	15.76095500	1.22282000	-1.40750300
H	15.83237300	-1.22152700	-2.49272900	H	15.74050400	-1.21835500	-2.49491000
C	14.80659600	-3.43884800	-1.40733000	C	14.76023900	-3.45447300	-1.40777600
C	14.80659600	-3.43884800	1.40733000	C	14.76023900	-3.45447300	1.40777600
H	14.80102200	-3.43559800	-2.49522200	H	14.74486100	-3.44305000	-2.49520500
H	14.80102200	-3.43559800	2.49522200	H	14.74486100	-3.44305000	2.49520500
C	15.40192600	2.39307600	-0.73433600	C	15.37271000	2.38863100	-0.72782500
H	15.83237300	1.22152700	2.49272900	H	15.74050400	1.21835500	2.49491000
H	15.83237300	-1.22152700	2.49272900	H	15.74050400	-1.21835500	2.49491000
H	15.83237300	1.22152700	-2.49272900	H	15.74050400	1.21835500	-2.49491000
C	5.88748600	5.25161700	1.40553700	C	5.87291100	5.27771500	1.40554500
C	10.69026900	5.92573600	1.40767800	C	10.66677600	5.96868700	1.40857700
C	10.69026900	5.92573600	-1.40767800	C	10.66677600	5.96868700	-1.40857700
C	11.84979000	5.61504000	-0.72919000	C	11.84330900	5.63787900	-0.72927800
C	11.84979000	5.61504000	0.72919000	C	11.84330900	5.63787900	0.72927800
C	5.88748600	5.25161700	-1.40553700	C	5.87291100	5.27771500	-1.40554500
C	12.98720300	5.07390900	-1.40835500	C	12.95675900	5.10692100	-1.40829900
C	12.98720300	5.07390900	1.40835500	C	12.95675900	5.10692100	1.40829900
H	5.89629400	5.23143300	2.49315400	H	5.88002100	5.25930400	2.49315600
H	5.89629400	5.23143300	-2.49315400	H	5.88002100	5.25930400	-2.49315600
C	13.94848500	4.36165400	0.73189800	C	13.93505600	4.36631400	0.72856800
C	13.94848500	4.36165400	-0.73189800	C	13.93505600	4.36631400	-0.72856800
C	14.80659600	3.43884800	-1.40733000	C	14.76023900	3.45447300	-1.40777600
C	14.80659600	3.43884800	1.40733000	C	14.76023900	3.45447300	1.40777600
H	10.68511400	5.90183600	-2.49523200	H	10.66394900	5.95325800	-2.49604300
H	10.68511400	5.90183600	2.49523200	H	10.66394900	5.95325800	2.49604300
H	12.97657600	5.05787400	2.49605000	H	12.94837800	5.09259800	2.49575000
H	12.97657600	5.05787400	-2.49605000	H	12.94837800	5.09259800	-2.49575000
H	14.80102200	3.43559800	-2.49522200	H	14.74486100	3.44305000	-2.49520500
H	14.80102200	3.43559800	2.49522200	H	14.74486100	3.44305000	2.49520500
C	10.69026900	-5.92573600	-1.40767800	C	10.66677600	-5.96868700	-1.40857700
C	9.44568700	-6.04641200	-0.72683900	C	9.43331900	-6.07939500	-0.72889900
C	9.44568700	-6.04641200	0.72683900	C	9.43331900	-6.07939500	0.72889900
C	10.69026900	-5.92573600	1.40767800	C	10.66677600	-5.96868700	1.40857700
H	10.68511400	-5.90183600	-2.49523200	H	10.66394900	-5.95325800	-2.49604300

H	10.68511400	-5.90183600	2.49523200	H	10.66394900	-5.95325800	2.49604300
C	9.44568700	6.04641200	-0.72683900	C	9.43331900	6.07939500	-0.72889900
C	8.23810300	5.95901100	-1.40643700	C	8.21806400	5.99785800	-1.40775800
C	7.03009700	5.69326000	-0.72518200	C	7.01052800	5.72055600	-0.72657900
C	7.03009700	5.69326000	0.72518200	C	7.01052800	5.72055600	0.72657900
C	8.23810300	5.95901100	1.40643700	C	8.21806400	5.99785800	1.40775800
C	9.44568700	6.04641200	0.72683900	C	9.43331900	6.07939500	0.72889900
H	8.24089100	5.93518100	-2.49399200	H	8.21983100	5.98070800	-2.49527600
H	8.24089100	5.93518100	2.49399200	H	8.21983100	5.98070800	2.49527600
C	13.94848500	-4.36165400	-0.73189800	C	13.93505600	-4.36631400	-0.72856800
C	12.98720300	-5.07390900	-1.40835500	C	12.95675900	-5.10692100	-1.40829900
C	11.84979000	-5.61504000	-0.72919000	C	11.84330900	-5.63787900	-0.72927800
C	11.84979000	-5.61504000	0.72919000	C	11.84330900	-5.63787900	0.72927800
C	12.98720300	-5.07390900	1.40835500	C	12.95675900	-5.10692100	1.40829900
C	13.94848500	-4.36165400	0.73189800	C	13.93505600	-4.36631400	0.72856800
H	12.97657600	-5.05787400	-2.49605000	H	12.94837800	-5.09259800	-2.49575000
H	12.97657600	-5.05787400	2.49605000	H	12.94837800	-5.09259800	2.49575000

**Dcyc18_R**				**Dcyc18_U**			
C	-12.92658800	5.76445300	1.40847800	C	-12.89821500	5.80672900	1.40823000
C	-11.73791000	6.17016700	0.72869200	C	-11.73012000	6.20268700	0.72935600
C	-10.54969800	6.36252000	1.40766200	C	-10.52593400	6.41025500	1.40848500
C	-11.73791000	6.17016700	-0.72869200	C	-11.73012000	6.20268700	-0.72935600
C	-10.54969800	6.36252000	-1.40766200	C	-10.52593400	6.41025500	-1.40848500
C	-9.30386900	6.37476200	-0.72689900	C	-9.28755100	6.41214900	-0.72896100
C	-9.30386900	6.37476200	0.72689900	C	-9.28755100	6.41214900	0.72896100
C	-13.97661200	5.18937800	0.73095100	C	-13.96687100	5.20376300	0.72871700
C	-12.92658800	5.76445300	-1.40847800	C	-12.89821500	5.80672900	-1.40823000
H	-10.54708500	6.33952900	-2.49522900	H	-10.52473200	6.39605100	-2.49598500
C	-8.10216700	6.19935200	-1.40671100	C	-8.08201000	6.24018000	-1.40773000
C	-8.10216700	6.19935200	1.40671100	C	-8.08201000	6.24018000	1.40773000
H	-8.10626700	6.17771700	-2.49428900	H	-8.08466600	6.22455300	-2.49527700
H	-8.10626700	6.17771700	2.49428900	H	-8.08466600	6.22455300	2.49527700
C	-13.97661200	5.18937800	-0.73095100	C	-13.96687100	5.20376300	-0.72871700
H	-12.91741100	5.74536000	2.49611600	H	-12.89232200	5.79286400	2.49571200
H	-10.54708500	6.33952900	2.49522900	H	-10.52473200	6.39605100	2.49598500
H	-12.91741100	5.74536000	-2.49611600	H	-12.89232200	5.79286400	-2.49571200
C	-6.91681000	5.86054400	0.72571000	C	-6.89398200	5.88884700	0.72661400
C	-6.91681000	5.86054400	-0.72571000	C	-6.89398200	5.88884700	-0.72661400
C	-16.34930900	-2.38940400	1.40618300	C	-16.28847300	-2.39806800	1.40743400
C	-16.68889500	-1.22554900	0.73512300	C	-16.64801100	-1.22205800	0.72746800
C	-16.84659400	0.00000000	1.40450700	C	-16.77481900	0.00000000	1.40735200
C	-16.68889500	-1.22554900	-0.73512300	C	-16.64801100	-1.22205800	-0.72746800
C	-16.84659400	0.00000000	-1.40450700	C	-16.77481900	0.00000000	-1.40735200
C	-16.68889500	1.22554900	-0.73512300	C	-16.64801100	1.22205800	-0.72746800
C	-16.68889500	1.22554900	0.73512300	C	-16.64801100	1.22205800	0.72746800
C	-16.34930900	-2.38940400	-1.40618300	C	-16.28847300	-2.39806800	-1.40743400
C	-16.34930900	2.38940400	-1.40618300	C	-16.28847300	2.39806800	-1.40743400
C	-16.34930900	2.38940400	1.40618300	C	-16.28847300	2.39806800	1.40743400
H	-16.34984400	-2.39006800	2.49413500	H	-16.27036400	-2.39036400	2.49487900
H	-16.34984400	-2.39006800	-2.49413500	H	-16.27036400	-2.39036400	-2.49487900
C	-15.72418200	3.47710000	0.73341300	C	-15.69825500	3.47481800	0.72794500
C	-15.72418200	3.47710000	-0.73341300	C	-15.69825500	3.47481800	-0.72794500
C	-14.95926900	4.39995500	-1.40825700	C	-14.91848400	4.42559700	-1.40780400
C	-14.95926900	4.39995500	1.40825700	C	-14.91848400	4.42559700	1.40780400
H	-16.85447400	0.00000000	-2.49246200	H	-16.75429300	0.00000000	-2.49478400
H	-16.85447400	0.00000000	2.49246200	H	-16.75429300	0.00000000	2.49478400
H	-16.34984400	2.39006800	2.49413500	H	-16.27036400	2.39036400	2.49487900
H	-16.34984400	2.39006800	-2.49413500	H	-16.27036400	2.39036400	-2.49487900

H	-14.94983400	4.39136500	-2.49607700	H	-14.90635400	4.41318500	-2.49527100
H	-14.94983400	4.39136500	2.49607700	H	-14.90635400	4.41318500	2.49527100
C	-3.76540500	-3.97498300	1.40571000	C	-3.75795300	-3.98921300	1.40335300
C	-4.74747400	-4.74039200	0.72506400	C	-4.72756100	-4.75475800	0.72318500
C	-5.79433200	-5.36230800	1.40602500	C	-5.78017000	-5.38949600	1.40551600
C	-4.74747400	-4.74039200	-0.72506400	C	-4.72756100	-4.75475800	-0.72318500
C	-5.79433200	-5.36230800	-1.40602500	C	-5.78017000	-5.38949600	-1.40551600
C	-6.91681000	-5.86054400	-0.72571000	C	-6.89398200	-5.88884700	-0.72661400
C	-6.91681000	-5.86054400	0.72571000	C	-6.89398200	-5.88884700	0.72661400
C	-2.89934400	-3.14481900	0.72501700	C	-2.88848000	-3.14996100	0.72150500
C	-3.76540500	-3.97498300	-1.40571000	C	-3.75795300	-3.98921300	-1.40335300
H	-5.80317500	-5.34433600	-2.49366500	H	-5.78743400	-5.37274800	-2.49315800
C	-8.10216700	-6.19935200	-1.40671100	C	-8.08201000	-6.24018000	-1.40773000
C	-8.10216700	-6.19935200	1.40671100	C	-8.08201000	-6.24018000	1.40773000
H	-8.10626700	-6.17771700	-2.49428900	H	-8.08466600	-6.22455300	-2.49527700
H	-8.10626700	-6.17771700	2.49428900	H	-8.08466600	-6.22455300	2.49527700
C	-2.89934400	-3.14481900	-0.72501700	C	-2.88848000	-3.14996100	-0.72150500
H	-3.77823900	-3.96016800	2.49344500	H	-3.77070900	-3.97352600	2.49111100
H	-5.80317500	-5.34433600	2.49366500	H	-5.78743400	-5.37274800	2.49315800
H	-3.77823900	-3.96016800	-2.49344500	H	-3.77070900	-3.97352600	-2.49111100
C	-5.79433200	5.36230800	1.40602500	C	-5.78017000	5.38949600	1.40551600
C	-2.09584000	2.16329700	1.40769100	C	-2.09360200	2.16850200	1.40531300
C	-2.09584000	2.16329700	-1.40769100	C	-2.09360200	2.16850200	-1.40531300
C	-1.42038900	1.20560900	-0.72563900	C	-1.41898900	1.20618100	-0.72287500
C	-1.42038900	1.20560900	0.72563900	C	-1.41898900	1.20618100	0.72287500
C	-5.79433200	5.36230800	-1.40602500	C	-5.78017000	5.38949600	-1.40551600
C	-0.79272900	0.00000000	-1.39877000	C	-0.79315300	0.00000000	-1.39740200
C	-0.79272900	0.00000000	1.39877000	C	-0.79315300	0.00000000	1.39740200
H	-5.80317500	5.34433600	2.49366500	H	-5.78743400	5.37274800	2.49315800
H	-5.80317500	5.34433600	-2.49366500	H	-5.78743400	5.37274800	-2.49315800
C	-1.42038900	-1.20560900	0.72563900	C	-1.41898900	-1.20618100	0.72287500
C	-1.42038900	-1.20560900	-0.72563900	C	-1.41898900	-1.20618100	-0.72287500
C	-2.09584000	-2.16329700	-1.40769100	C	-2.09360200	-2.16850200	-1.40531300
C	-2.09584000	-2.16329700	1.40769100	C	-2.09360200	-2.16850200	1.40531300
H	-2.12533900	2.14472600	-2.49528000	H	-2.12282300	2.15042800	-2.49294100
H	-2.12533900	2.14472600	2.49528000	H	-2.12282300	2.15042800	2.49294100
H	-1.10191500	0.00000000	2.44791000	H	-1.10316300	0.00000000	2.44635400
H	-1.10191500	0.00000000	-2.44791000	H	-1.10316300	0.00000000	-2.44635400
H	-2.12533900	-2.14472600	-2.49528000	H	-2.12282300	-2.15042800	-2.49294100
H	-2.12533900	-2.14472600	2.49528000	H	-2.12282300	-2.15042800	2.49294100
C	-14.95926900	-4.39995500	-1.40825700	C	-14.91848400	-4.42559700	-1.40780400
C	-15.72418200	-3.47710000	-0.73341300	C	-15.69825500	-3.47481800	-0.72794500
C	-15.72418200	-3.47710000	0.73341300	C	-15.69825500	-3.47481800	0.72794500
C	-14.95926900	-4.39995500	1.40825700	C	-14.91848400	-4.42559700	1.40780400
H	-14.94983400	-4.39136500	-2.49607700	H	-14.90635400	-4.41318500	-2.49527100
H	-14.94983400	-4.39136500	2.49607700	H	-14.90635400	-4.41318500	2.49527100
C	-2.89934400	3.14481900	-0.72501700	C	-2.88848000	3.14996100	-0.72150500
C	-3.76540500	3.97498300	-1.40571000	C	-3.75795300	3.98921300	-1.40335300
C	-4.74747400	4.74039200	-0.72506400	C	-4.72756100	4.75475800	-0.72318500
C	-4.74747400	4.74039200	0.72506400	C	-4.72756100	4.75475800	0.72318500
C	-3.76540500	3.97498300	1.40571000	C	-3.75795300	3.98921300	1.40335300
C	-2.89934400	3.14481900	0.72501700	C	-2.88848000	3.14996100	0.72150500
H	-3.77823900	3.96016800	-2.49344500	H	-3.77070900	3.97352600	-2.49111100
H	-3.77823900	3.96016800	2.49344500	H	-3.77070900	3.97352600	2.49111100
C	-9.30386900	-6.37476200	-0.72689900	C	-9.28755100	-6.41214900	-0.72896100
C	-10.54969800	-6.36252000	-1.40766200	C	-10.52593400	-6.41025500	-1.40848500
C	-10.54969800	-6.36252000	1.40766200	C	-10.52593400	-6.41025500	1.40848500
C	-9.30386900	-6.37476200	0.72689900	C	-9.28755100	-6.41214900	0.72896100
H	-10.54708500	-6.33952900	-2.49522900	H	-10.52473200	-6.39605100	-2.49598500
H	-10.54708500	-6.33952900	2.49522900	H	-10.52473200	-6.39605100	2.49598500

C	-11.73791000	-6.17016700	-0.72869200	C	-11.73012000	-6.20268700	-0.72935600
C	-12.92658800	-5.76445300	-1.40847800	C	-12.89821500	-5.80672900	-1.40823000
C	-13.97661200	-5.18937800	-0.73095100	C	-13.96687100	-5.20376300	-0.72871700
C	-13.97661200	-5.18937800	0.73095100	C	-13.96687100	-5.20376300	0.72871700
C	-12.92658800	-5.76445300	1.40847800	C	-12.89821500	-5.80672900	1.40823000
C	-11.73791000	-6.17016700	0.72869200	C	-11.73012000	-6.20268700	0.72935600
H	-12.91741100	-5.74536000	-2.49611600	H	-12.89232200	-5.79286400	-2.49571200
H	-12.91741100	-5.74536000	2.49611600	H	-12.89232200	-5.79286400	2.49571200
C	3.76540500	3.97498300	1.40571000	C	3.75795300	3.98921300	1.40335300
C	4.74747400	4.74039200	0.72506400	C	4.72756100	4.75475800	0.72318500
C	5.79433200	5.36230800	1.40602500	C	5.78017000	5.38949600	1.40551600
C	4.74747400	4.74039200	-0.72506400	C	4.72756100	4.75475800	-0.72318500
C	5.79433200	5.36230800	-1.40602500	C	5.78017000	5.38949600	-1.40551600
C	6.91681000	5.86054400	-0.72571000	C	6.89398200	5.88884700	-0.72661400
C	6.91681000	5.86054400	0.72571000	C	6.89398200	5.88884700	0.72661400
C	2.89934400	3.14481900	0.72501700	C	2.88848000	3.14996100	0.72150500
C	3.76540500	3.97498300	-1.40571000	C	3.75795300	3.98921300	-1.40335300
H	5.80317500	5.34433600	-2.49366500	H	5.78743400	5.37274800	-2.49315800
C	8.10216700	6.19935200	-1.40671100	C	8.08201000	6.24018000	-1.40773000
C	8.10216700	6.19935200	1.40671100	C	8.08201000	6.24018000	1.40773000
H	8.10626700	6.17771700	-2.49428900	H	8.08466600	6.22455300	-2.49527700
H	8.10626700	6.17771700	2.49428900	H	8.08466600	6.22455300	2.49527700
C	2.89934400	3.14481900	-0.72501700	C	2.88848000	3.14996100	-0.72150500
H	3.77823900	3.96016800	2.49344500	H	3.77070900	3.97352600	2.49111100
H	5.80317500	5.34433600	2.49366500	H	5.78743400	5.37274800	2.49315800
H	3.77823900	3.96016800	-2.49344500	H	3.77070900	3.97352600	-2.49111100
C	9.30386900	6.37476200	0.72689900	C	9.28755100	6.41214900	0.72896100
C	9.30386900	6.37476200	-0.72689900	C	9.28755100	6.41214900	-0.72896100
C	3.76540500	-3.97498300	1.40571000	C	3.75795300	-3.98921300	1.40335300
C	2.89934400	-3.14481900	0.72501700	C	2.88848000	-3.14996100	0.72150500
C	2.09584000	-2.16329700	1.40769100	C	2.09360200	-2.16850200	1.40531300
C	2.89934400	-3.14481900	-0.72501700	C	2.88848000	-3.14996100	-0.72150500
C	2.09584000	-2.16329700	-1.40769100	C	2.09360200	-2.16850200	-1.40531300
C	1.42038900	-1.20560900	-0.72563900	C	1.41898900	-1.20618100	-0.72287500
C	1.42038900	-1.20560900	0.72563900	C	1.41898900	-1.20618100	0.72287500
C	3.76540500	-3.97498300	-1.40571000	C	3.75795300	-3.98921300	-1.40335300
C	0.79272900	0.00000000	-1.39877000	C	0.79315300	0.00000000	-1.39740200
C	0.79272900	0.00000000	1.39877000	C	0.79315300	0.00000000	1.39740200
H	3.77823900	-3.96016800	2.49344500	H	3.77070900	-3.97352600	2.49111100
H	3.77823900	-3.96016800	-2.49344500	H	3.77070900	-3.97352600	-2.49111100
C	1.42038900	1.20560900	0.72563900	C	1.41898900	1.20618100	0.72287500
C	1.42038900	1.20560900	-0.72563900	C	1.41898900	1.20618100	-0.72287500
C	2.09584000	2.16329700	-1.40769100	C	2.09360200	2.16850200	-1.40531300
C	2.09584000	2.16329700	1.40769100	C	2.09360200	2.16850200	1.40531300
H	2.12533900	-2.14472600	-2.49528000	H	2.12282300	-2.15042800	-2.49294100
H	2.12533900	-2.14472600	2.49528000	H	2.12282300	-2.15042800	2.49294100
H	1.10191500	0.00000000	2.44791000	H	1.10316300	0.00000000	2.44635400
H	1.10191500	0.00000000	-2.44791000	H	1.10316300	0.00000000	-2.44635400
H	2.12533900	2.14472600	-2.49528000	H	2.12282300	2.15042800	-2.49294100
H	2.12533900	2.14472600	2.49528000	H	2.12282300	2.15042800	2.49294100
C	16.34930900	-2.38940400	1.40618300	C	16.28847300	-2.39806800	1.40743400
C	15.72418200	-3.47710000	0.73341300	C	15.69825500	-3.47481800	0.72794500
C	14.95926900	-4.39995500	1.40825700	C	14.91848400	-4.42559700	1.40780400
C	15.72418200	-3.47710000	-0.73341300	C	15.69825500	-3.47481800	-0.72794500
C	14.95926900	-4.39995500	-1.40825700	C	14.91848400	-4.42559700	-1.40780400
C	13.97661200	-5.18937800	-0.73095100	C	13.96687100	-5.20376300	-0.72871700
C	13.97661200	-5.18937800	0.73095100	C	13.96687100	-5.20376300	0.72871700
C	16.68889500	-1.22554900	0.73512300	C	16.64801100	-1.22205800	0.72746800
C	16.34930900	-2.38940400	-1.40618300	C	16.28847300	-2.39806800	-1.40743400
H	14.94983400	-4.39136500	-2.49607700	H	14.90635400	-4.41318500	-2.49527100

C	12.92658800	-5.76445300	-1.40847800	C	12.89821500	-5.80672900	-1.40823000
C	12.92658800	-5.76445300	1.40847800	C	12.89821500	-5.80672900	1.40823000
H	12.91741100	-5.74536000	-2.49611600	H	12.89232200	-5.79286400	-2.49571200
H	12.91741100	-5.74536000	2.49611600	H	12.89232200	-5.79286400	2.49571200
C	16.68889500	-1.22554900	-0.73512300	C	16.64801100	-1.22205800	-0.72746800
H	16.34984400	-2.39006800	2.49413500	H	16.27036400	-2.39036400	2.49487900
H	14.94983400	-4.39136500	2.49607700	H	14.90635400	-4.41318500	2.49527100
H	16.34984400	-2.39006800	-2.49413500	H	16.27036400	-2.39036400	-2.49487900
C	10.54969800	6.36252000	1.40766200	C	10.52593400	6.41025500	1.40848500
C	14.95926900	4.39995500	1.40825700	C	14.91848400	4.42559700	1.40780400
C	14.95926900	4.39995500	-1.40825700	C	14.91848400	4.42559700	-1.40780400
C	15.72418200	3.47710000	-0.73341300	C	15.69825500	3.47481800	-0.72794500
C	15.72418200	3.47710000	0.73341300	C	15.69825500	3.47481800	0.72794500
C	10.54969800	6.36252000	-1.40766200	C	10.52593400	6.41025500	-1.40848500
C	16.34930900	2.38940400	-1.40618300	C	16.28847300	2.39806800	-1.40743400
C	16.34930900	2.38940400	1.40618300	C	16.28847300	2.39806800	1.40743400
H	10.54708500	6.33952900	2.49522900	H	10.52473200	6.39605100	2.49598500
H	10.54708500	6.33952900	-2.49522900	H	10.52473200	6.39605100	-2.49598500
C	16.68889500	1.22554900	0.73512300	C	16.64801100	1.22205800	0.72746800
C	16.68889500	1.22554900	-0.73512300	C	16.64801100	1.22205800	-0.72746800
C	16.84659400	0.00000000	-1.40450700	C	16.77481900	0.00000000	-1.40735200
C	16.84659400	0.00000000	1.40450700	C	16.77481900	0.00000000	1.40735200
H	14.94983400	4.39136500	-2.49607700	H	14.90635400	4.41318500	-2.49527100
H	14.94983400	4.39136500	2.49607700	H	14.90635400	4.41318500	2.49527100
H	16.34984400	2.39006800	2.49413500	H	16.27036400	2.39036400	2.49487900
H	16.34984400	2.39006800	-2.49413500	H	16.27036400	2.39036400	-2.49487900
H	16.85447400	0.00000000	-2.49246200	H	16.75429300	0.00000000	-2.49478400
H	16.85447400	0.00000000	2.49246200	H	16.75429300	0.00000000	2.49478400
C	5.79433200	-5.36230800	-1.40602500	C	5.78017000	-5.38949600	-1.40551600
C	4.74747400	-4.74039200	-0.72506400	C	4.72756100	-4.75475800	-0.72318500
C	4.74747400	-4.74039200	0.72506400	C	4.72756100	-4.75475800	0.72318500
C	5.79433200	-5.36230800	1.40602500	C	5.78017000	-5.38949600	1.40551600
H	5.80317500	-5.34433600	-2.49366500	H	5.78743400	-5.37274800	-2.49315800
H	5.80317500	-5.34433600	2.49366500	H	5.78743400	-5.37274800	2.49315800
C	13.97661200	5.18937800	-0.73095100	C	13.96687100	5.20376300	-0.72871700
C	12.92658800	5.76445300	-1.40847800	C	12.89821500	5.80672900	-1.40823000
C	11.73791000	6.17016700	-0.72869200	C	11.73012000	6.20268700	-0.72935600
C	11.73791000	6.17016700	0.72869200	C	11.73012000	6.20268700	0.72935600
C	12.92658800	5.76445300	1.40847800	C	12.89821500	5.80672900	1.40823000
C	13.97661200	5.18937800	0.73095100	C	13.96687100	5.20376300	0.72871700
H	12.91741100	5.74536000	-2.49611600	H	12.89232200	5.79286400	-2.49571200
H	12.91741100	5.74536000	2.49611600	H	12.89232200	5.79286400	2.49571200
C	11.73791000	-6.17016700	-0.72869200	C	11.73012000	-6.20268700	-0.72935600
C	10.54969800	-6.36252000	-1.40766200	C	10.52593400	-6.41025500	-1.40848500
C	10.54969800	-6.36252000	1.40766200	C	10.52593400	-6.41025500	1.40848500
C	11.73791000	-6.17016700	0.72869200	C	11.73012000	-6.20268700	0.72935600
H	10.54708500	-6.33952900	-2.49522900	H	10.52473200	-6.39605100	-2.49598500
H	10.54708500	-6.33952900	2.49522900	H	10.52473200	-6.39605100	2.49598500
C	9.30386900	-6.37476200	-0.72689900	C	9.28755100	-6.41214900	-0.72896100
C	8.10216700	-6.19935200	-1.40671100	C	8.08201000	-6.24018000	-1.40773000
C	6.91681000	-5.86054400	-0.72571000	C	6.89398200	-5.88884700	-0.72661400
C	6.91681000	-5.86054400	0.72571000	C	6.89398200	-5.88884700	0.72661400
C	8.10216700	-6.19935200	1.40671100	C	8.08201000	-6.24018000	1.40773000
C	9.30386900	-6.37476200	0.72689900	C	9.28755100	-6.41214900	0.72896100
H	8.10626700	-6.17771700	-2.49428900	H	8.08466600	-6.22455300	-2.49527700
H	8.10626700	-6.17771700	2.49428900	H	8.08466600	-6.22455300	2.49527700
**Dcyc19_R**				**Dcyc19_U**			
C	-17.60645900	1.21981300	1.40507300	C	-17.53877400	1.22390800	1.40728200

C	-17.22578600	2.40621700	0.73450900	C	-17.19028100	2.40121700	0.72760200
C	-16.68588800	3.48235900	1.40744100	C	-16.63438300	3.49866700	1.40741100
C	-17.22578600	2.40621700	-0.73450900	C	-17.19028100	2.40121700	-0.72760200
C	-16.68588800	3.48235900	-1.40744100	C	-16.63438300	3.49866700	-1.40741100
C	-15.89976300	4.46528500	-0.73241200	C	-15.88158000	4.46813800	-0.72812100
C	-15.89976300	4.46528500	0.73241200	C	-15.88158000	4.46813800	0.72812100
C	-17.69534900	0.00000000	0.73543700	C	-17.65200100	0.00000000	0.72737900
C	-17.60645900	1.21981300	-1.40507300	C	-17.53877400	1.22390800	-1.40728200
H	-16.68077800	3.47970200	-2.49534900	H	-16.61912000	3.48883600	-2.49489400
C	-15.00794000	5.26312800	-1.40871000	C	-14.97259000	5.29643300	-1.40768600
C	-15.00794000	5.26312800	1.40871000	C	-14.97259000	5.29643300	1.40768600
H	-14.99733300	5.24993500	-2.49645400	H	-14.96303400	5.28357000	-2.49518800
H	-14.99733300	5.24993500	2.49645400	H	-14.96303400	5.28357000	2.49518800
C	-17.69534900	0.00000000	-0.73543700	C	-17.65200100	0.00000000	-0.72737900
H	-17.61170200	1.22105600	2.49304600	H	-17.51960800	1.22013800	2.49473800
H	-16.68077800	3.47970200	2.49534900	H	-16.61912000	3.48883600	2.49489400
H	-17.61170200	1.22105600	-2.49304600	H	-17.51960800	1.22013800	-2.49473800
C	-13.93524100	5.92249300	0.73020200	C	-13.92808200	5.94435000	0.72884000
C	-13.93524100	5.92249300	-0.73020200	C	-13.92808200	5.94435000	-0.72884000
C	-12.82393300	-6.37320400	1.40852200	C	-12.79685700	-6.41910800	1.40813600
C	-13.93524100	-5.92249300	0.73020200	C	-13.92808200	-5.94435000	0.72884000
C	-15.00794000	-5.26312800	1.40871000	C	-14.97259000	-5.29643300	1.40768600
C	-13.93524100	-5.92249300	-0.73020200	C	-13.92808200	-5.94435000	-0.72884000
C	-15.00794000	-5.26312800	-1.40871000	C	-14.97259000	-5.29643300	-1.40768600
C	-15.89976300	-4.46528500	-0.73241200	C	-15.88158000	-4.46813800	-0.72812100
C	-15.89976300	-4.46528500	0.73241200	C	-15.88158000	-4.46813800	0.72812100
C	-12.82393300	-6.37320400	-1.40852200	C	-12.79685700	-6.41910800	-1.40813600
C	-16.68588800	-3.48235900	-1.40744100	C	-16.63438300	-3.49866700	-1.40741100
C	-16.68588800	-3.48235900	1.40744100	C	-16.63438300	-3.49866700	1.40741100
H	-12.81684800	-6.35272400	2.49613900	H	-12.79281200	-6.40586300	2.49564700
H	-12.81684800	-6.35272400	-2.49613900	H	-12.79281200	-6.40586300	-2.49564700
C	-17.22578600	-2.40621700	0.73450900	C	-17.19028100	-2.40121700	0.72760200
C	-17.22578600	-2.40621700	-0.73450900	C	-17.19028100	-2.40121700	-0.72760200
C	-17.60645900	-1.21981300	-1.40507300	C	-17.53877400	-1.22390800	-1.40728200
C	-17.60645900	-1.21981300	1.40507300	C	-17.53877400	-1.22390800	1.40728200
H	-14.99733300	-5.24993500	-2.49645400	H	-14.96303400	-5.28357000	-2.49518800
H	-14.99733300	-5.24993500	2.49645400	H	-14.96303400	-5.28357000	2.49518800
H	-16.68077800	-3.47970200	2.49534900	H	-16.61912000	-3.48883600	2.49489400
H	-16.68077800	-3.47970200	-2.49534900	H	-16.61912000	-3.48883600	-2.49489400
H	-17.61170200	-1.22105600	-2.49304600	H	-17.51960800	-1.22013800	-2.49473800
H	-17.61170200	-1.22105600	2.49304600	H	-17.51960800	-1.22013800	2.49473800
C	-2.08487400	2.17020200	1.40811100	C	-2.08218100	2.17577400	1.40522800
C	-1.41844000	1.20697300	0.72602700	C	-1.41650000	1.20723200	0.72276300
C	-0.79329700	0.00000000	1.39893400	C	-0.79368000	0.00000000	1.39753000
C	-1.41844000	1.20697300	-0.72602700	C	-1.41650000	1.20723200	-0.72276300
C	-0.79329700	0.00000000	-1.39893400	C	-0.79368000	0.00000000	-1.39753000
C	-1.41844000	-1.20697300	-0.72602700	C	-1.41650000	-1.20723200	-0.72276300
C	-1.41844000	-1.20697300	0.72602700	C	-1.41650000	-1.20723200	0.72276300
C	-2.87496500	3.16350900	0.72561600	C	-2.86361600	3.16775300	0.72165600
C	-2.08487400	2.17020200	-1.40811100	C	-2.08218100	2.17577400	-1.40522800
H	-1.10233600	0.00000000	-2.44811800	H	-1.10362200	0.00000000	-2.44648800
C	-2.08487400	-2.17020200	-1.40811100	C	-2.08218100	-2.17577400	-1.40522800
C	-2.08487400	-2.17020200	1.40811100	C	-2.08218100	-2.17577400	1.40522800
H	-2.11362900	-2.15188500	-2.49575500	H	-2.11030000	-2.15894800	-2.49291700
H	-2.11362900	-2.15188500	2.49575500	H	-2.11030000	-2.15894700	2.49291700
C	-2.87496500	3.16350900	-0.72561600	C	-2.86361600	3.16775300	-0.72165600
H	-2.11362900	2.15188500	2.49575500	H	-2.11030000	2.15894800	2.49291700
H	-1.10233600	0.00000000	2.44811800	H	-1.10362200	0.00000000	2.44648800
H	-2.11362900	2.15188500	-2.49575500	H	-2.11030000	2.15894800	-2.49291700
C	-12.82393300	6.37320400	1.40852200	C	-12.79685700	6.41910800	1.40813600

C	-7.97013700	6.41070000	1.40699900	C	-7.95025500	6.45171300	1.40762300
C	-7.97013700	6.41070000	-1.40699900	C	-7.95025500	6.45171300	-1.40762300
C	-6.80832700	6.00957400	-0.72617800	C	-6.78303700	6.03715600	-0.72658600
C	-6.80832700	6.00957400	0.72617800	C	-6.78303700	6.03715600	0.72658600
C	-12.82393300	6.37320400	-1.40852200	C	-12.79685700	6.41910800	-1.40813600
C	-5.70620200	5.46141900	-1.40650000	C	-5.69235800	5.48890700	-1.40547900
C	-5.70620200	5.46141900	1.40650000	C	-5.69235800	5.48890700	1.40547900
H	-12.81684800	6.35272400	2.49613900	H	-12.79281200	6.40586300	2.49564700
H	-12.81684800	6.35272400	-2.49613900	H	-12.79281200	6.40586300	-2.49564700
C	-4.68456100	4.80365900	0.72568800	C	-4.66342800	4.81694700	0.72324600
C	-4.68456100	4.80365900	-0.72568800	C	-4.66342800	4.81694700	-0.72324600
C	-3.72224800	4.01077500	-1.40621600	C	-3.71519100	4.02505700	-1.40336900
C	-3.72224800	4.01077500	1.40621600	C	-3.71519100	4.02505700	1.40336900
H	-7.97518300	6.39112400	-2.49461400	H	-7.95342700	6.43725700	-2.49519500
H	-7.97518300	6.39112400	2.49461400	H	-7.95342700	6.43725700	2.49519500
H	-5.71509000	5.44520500	2.49416500	H	-5.69982800	5.47356600	2.49313700
H	-5.71509000	5.44520500	-2.49416500	H	-5.69982800	5.47356600	-2.49313700
H	-3.73447700	3.99736600	-2.49398100	H	-3.72764700	4.01087200	-2.49114500
H	-3.73447700	3.99736600	2.49398100	H	-3.72764700	4.01087200	2.49114500
C	-7.97013700	-6.41070000	-1.40699900	C	-7.95025500	-6.45171300	-1.40762300
C	-7.97013700	-6.41070000	1.40699900	C	-7.95025500	-6.45171300	1.40762300
H	-7.97518300	-6.39112400	-2.49461400	H	-7.95342700	-6.43725700	-2.49519500
H	-7.97518300	-6.39112400	2.49461400	H	-7.95342700	-6.43725700	2.49519500
C	-9.16163900	6.66294500	-0.72702200	C	-9.14193800	6.70166800	-0.72896100
C	-10.40043100	6.74474700	-1.40773200	C	-10.37686800	6.79378000	-1.40839400
C	-11.60445400	6.65719000	-0.72834300	C	-11.59456400	6.69438300	-0.72942300
C	-11.60445400	6.65719000	0.72834300	C	-11.59456400	6.69438300	0.72942300
C	-10.40043100	6.74474700	1.40773200	C	-10.37686800	6.79378000	1.40839400
C	-9.16163900	6.66294500	0.72702200	C	-9.14193800	6.70166800	0.72896100
H	-10.39989500	6.72291800	-2.49531600	H	-10.37670000	6.78060300	-2.49592000
H	-10.39989500	6.72291800	2.49531600	H	-10.37670000	6.78060300	2.49592000
C	-2.87496500	-3.16350900	-0.72561600	C	-2.86361600	-3.16775300	-0.72165600
C	-3.72224800	-4.01077500	-1.40621600	C	-3.71519100	-4.02505700	-1.40336900
C	-3.72224800	-4.01077500	1.40621600	C	-3.71519100	-4.02505700	1.40336900
C	-2.87496500	-3.16350900	0.72561600	C	-2.86361600	-3.16775300	0.72165600
H	-3.73447700	-3.99736600	-2.49398100	H	-3.72764700	-4.01087200	-2.49114500
H	-3.73447700	-3.99736600	2.49398100	H	-3.72764700	-4.01087200	2.49114500
C	-4.68456100	-4.80365900	-0.72568800	C	-4.66342800	-4.81694700	-0.72324600
C	-5.70620200	-5.46141900	-1.40650000	C	-5.69235800	-5.48890700	-1.40547900
C	-6.80832700	-6.00957400	-0.72617800	C	-6.78303700	-6.03715600	-0.72658600
C	-6.80832700	-6.00957400	0.72617800	C	-6.78303700	-6.03715600	0.72658600
C	-5.70620200	-5.46141900	1.40650000	C	-5.69235800	-5.48890700	1.40547900
C	-4.68456100	-4.80365900	0.72568800	C	-4.66342800	-4.81694700	0.72324600
H	-5.71509000	-5.44520500	-2.49416500	H	-5.69982800	-5.47356600	-2.49313700
H	-5.71509000	-5.44520500	2.49416500	H	-5.69982800	-5.47356600	2.49313700
C	-9.16163900	-6.66294500	-0.72702200	C	-9.14193800	-6.70166800	-0.72896100
C	-10.40043100	-6.74474700	-1.40773200	C	-10.37686800	-6.79378000	-1.40839400
C	-11.60445400	-6.65719000	-0.72834300	C	-11.59456400	-6.69438300	-0.72942300
C	-11.60445400	-6.65719000	0.72834300	C	-11.59456400	-6.69438300	0.72942300
C	-10.40043100	-6.74474700	1.40773200	C	-10.37686800	-6.79378000	1.40839400
C	-9.16163900	-6.66294500	0.72702200	C	-9.14193800	-6.70166800	0.72896100
H	-10.39989500	-6.72291800	-2.49531600	H	-10.37670000	-6.78060300	-2.49592000
H	-10.39989500	-6.72291800	2.49531600	H	-10.37670000	-6.78060300	2.49592000
C	2.08487400	2.17020200	1.40811100	C	2.08218100	2.17577400	1.40522800
C	2.87496500	3.16350900	0.72561600	C	2.86361600	3.16775300	0.72165600
C	3.72224800	4.01077500	1.40621600	C	3.71519100	4.02505700	1.40336900
C	2.87496500	3.16350900	-0.72561600	C	2.86361600	3.16775300	-0.72165600
C	3.72224800	4.01077500	-1.40621600	C	3.71519100	4.02505700	-1.40336900
C	4.68456100	4.80365900	-0.72568800	C	4.66342800	4.81694700	-0.72324600
C	4.68456100	4.80365900	0.72568800	C	4.66342800	4.81694700	0.72324600

C	1.41844000	1.20697300	0.72602700	C	1.41650000	1.20723200	0.72276300
C	2.08487400	2.17020200	-1.40811100	C	2.08218100	2.17577400	-1.40522800
H	3.73447700	3.99736600	-2.49398100	H	3.72764700	4.01087200	-2.49114500
C	5.70620200	5.46141900	-1.40650000	C	5.69235800	5.48890700	-1.40547900
C	5.70620200	5.46141900	1.40650000	C	5.69235800	5.48890700	1.40547900
H	5.71509000	5.44520500	-2.49416500	H	5.69982800	5.47356600	-2.49313700
H	5.71509000	5.44520500	2.49416500	H	5.69982800	5.47356600	2.49313700
C	1.41844000	1.20697300	-0.72602700	C	1.41650000	1.20723200	-0.72276300
H	2.11362900	2.15188500	2.49575500	H	2.11030000	2.15894800	2.49291700
H	3.73447700	3.99736600	2.49398100	H	3.72764700	4.01087200	2.49114500
H	2.11362900	2.15188500	-2.49575500	H	2.11030000	2.15894800	-2.49291700
C	6.80832700	6.00957400	0.72617800	C	6.78303700	6.03715600	0.72658600
C	6.80832700	6.00957400	-0.72617800	C	6.78303700	6.03715600	-0.72658600
C	5.70620200	-5.46141900	1.40650000	C	5.69235800	-5.48890700	1.40547900
C	4.68456100	-4.80365900	0.72568800	C	4.66342800	-4.81694700	0.72324600
C	3.72224800	-4.01077500	1.40621600	C	3.71519100	-4.02505700	1.40336900
C	4.68456100	-4.80365900	-0.72568800	C	4.66342800	-4.81694700	-0.72324600
C	3.72224800	-4.01077500	-1.40621600	C	3.71519100	-4.02505700	-1.40336900
C	2.87496500	-3.16350900	-0.72561600	C	2.86361600	-3.16775300	-0.72165600
C	2.87496500	-3.16350900	0.72561600	C	2.86361600	-3.16775300	0.72165600
C	5.70620200	-5.46141900	-1.40650000	C	5.69235800	-5.48890700	-1.40547900
C	2.08487400	-2.17020200	-1.40811100	C	2.08218100	-2.17577400	-1.40522800
C	2.08487400	-2.17020200	1.40811100	C	2.08218100	-2.17577400	1.40522800
H	5.71509000	-5.44520500	2.49416500	H	5.69982800	-5.47356600	2.49313700
H	5.71509000	-5.44520500	-2.49416500	H	5.69982800	-5.47356600	-2.49313700
C	1.41844000	-1.20697300	0.72602700	C	1.41650000	-1.20723200	0.72276300
C	1.41844000	-1.20697300	-0.72602700	C	1.41650000	-1.20723200	-0.72276300
C	0.79329700	0.00000000	-1.39893400	C	0.79368000	0.00000000	-1.39753000
C	0.79329700	0.00000000	1.39893400	C	0.79368000	0.00000000	1.39753000
H	3.73447700	-3.99736600	-2.49398100	H	3.72764700	-4.01087200	-2.49114500
H	3.73447700	-3.99736600	2.49398100	H	3.72764700	-4.01087200	2.49114500
H	2.11362900	-2.15188500	2.49575500	H	2.11030000	-2.15894800	2.49291700
H	2.11362900	-2.15188500	-2.49575500	H	2.11030000	-2.15894800	-2.49291700
H	1.10233600	0.00000000	-2.44811800	H	1.10362200	0.00000000	-2.44648800
H	1.10233600	0.00000000	2.44811800	H	1.10362200	0.00000000	2.44648800
C	17.60645900	1.21981300	1.40507300	C	17.53877400	1.22390800	1.40728200
C	17.69534900	0.00000000	0.73543700	C	17.65200100	0.00000000	0.72737900
C	17.60645900	-1.21981300	1.40507300	C	17.53877400	-1.22390800	1.40728200
C	17.69534900	0.00000000	-0.73543700	C	17.65200100	0.00000000	-0.72737900
C	17.60645900	-1.21981300	-1.40507300	C	17.53877400	-1.22390800	-1.40728200
C	17.22578600	-2.40621700	-0.73450900	C	17.19028100	-2.40121700	-0.72760200
C	17.22578600	-2.40621700	0.73450900	C	17.19028100	-2.40121700	0.72760200
C	17.22578600	2.40621700	0.73450900	C	17.19028100	2.40121700	0.72760200
C	17.60645900	1.21981300	-1.40507300	C	17.53877400	1.22390800	-1.40728200
H	17.61170200	-1.22105600	-2.49304600	H	17.51960800	-1.22013800	-2.49473800
C	16.68588800	-3.48235900	-1.40744100	C	16.63438300	-3.49866700	-1.40741100
C	16.68588800	-3.48235900	1.40744100	C	16.63438300	-3.49866700	1.40741100
H	16.68077800	-3.47970200	-2.49534900	H	16.61912000	-3.48883600	-2.49489400
H	16.68077800	-3.47970200	2.49534900	H	16.61912000	-3.48883600	2.49489400
C	17.22578600	2.40621700	-0.73450900	C	17.19028100	2.40121700	-0.72760200
H	17.61170200	1.22105600	2.49304600	H	17.51960800	1.22013800	2.49473800
H	17.61170200	-1.22105600	2.49304600	H	17.51960800	-1.22013800	2.49473800
H	17.61170200	1.22105600	-2.49304600	H	17.51960800	1.22013800	-2.49473800
C	7.97013700	6.41070000	1.40699900	C	7.95025500	6.45171300	1.40762300
C	12.82393300	6.37320400	1.40852200	C	12.79685700	6.41910800	1.40813600
C	12.82393300	6.37320400	-1.40852200	C	12.79685700	6.41910800	-1.40813600
C	13.93524100	5.92249300	-0.73020200	C	13.92808200	5.94435000	-0.72884000
C	13.93524100	5.92249300	0.73020200	C	13.92808200	5.94435000	0.72884000
C	7.97013700	6.41070000	-1.40699900	C	7.95025500	6.45171300	-1.40762300
C	15.00794000	5.26312800	-1.40871000	C	14.97259000	5.29643300	-1.40768600

C	15.00794000	5.26312800	1.40871000	C	14.97259000	5.29643300	1.40768600
H	7.97518300	6.39112400	2.49461400	H	7.95342700	6.43725700	2.49519500
H	7.97518300	6.39112400	-2.49461400	H	7.95342700	6.43725700	-2.49519500
C	15.89976300	4.46528500	0.73241200	C	15.88158000	4.46813800	0.72812100
C	15.89976300	4.46528500	-0.73241200	C	15.88158000	4.46813800	-0.72812100
C	16.68588800	3.48235900	-1.40744100	C	16.63438300	3.49866700	-1.40741100
C	16.68588800	3.48235900	1.40744100	C	16.63438300	3.49866700	1.40741100
H	12.81684800	6.35272400	-2.49613900	H	12.79281200	6.40586300	-2.49564700
H	12.81684800	6.35272400	2.49613900	H	12.79281200	6.40586300	2.49564700
H	14.99733300	5.24993500	2.49645400	H	14.96303400	5.28357000	2.49518800
H	14.99733300	5.24993500	-2.49645400	H	14.96303400	5.28357000	-2.49518800
H	16.68077800	3.47970200	-2.49534900	H	16.61912000	3.48883600	-2.49489400
H	16.68077800	3.47970200	2.49534900	H	16.61912000	3.48883600	2.49489400
C	10.40043100	-6.74474700	-1.40773200	C	10.37686800	-6.79378000	-1.40839400
C	10.40043100	-6.74474700	1.40773200	C	10.37686800	-6.79378000	1.40839400
H	10.39989500	-6.72291800	-2.49531600	H	10.37670000	-6.78060300	-2.49592000
H	10.39989500	-6.72291800	2.49531600	H	10.37670000	-6.78060300	2.49592000
C	11.60445400	6.65719000	-0.72834300	C	11.59456400	6.69438300	-0.72942300
C	10.40043100	6.74474700	-1.40773200	C	10.37686800	6.79378000	-1.40839400
C	9.16163900	6.66294500	-0.72702200	C	9.14193800	6.70166800	-0.72896100
C	9.16163900	6.66294500	0.72702200	C	9.14193800	6.70166800	0.72896100
C	10.40043100	6.74474700	1.40773200	C	10.37686800	6.79378000	1.40839400
C	11.60445400	6.65719000	0.72834300	C	11.59456400	6.69438300	0.72942300
H	10.39989500	6.72291800	-2.49531600	H	10.37670000	6.78060300	-2.49592000
H	10.39989500	6.72291800	2.49531600	H	10.37670000	6.78060300	2.49592000
C	15.89976300	-4.46528500	-0.73241200	C	15.88158000	-4.46813800	-0.72812100
C	15.00794000	-5.26312800	-1.40871000	C	14.97258900	-5.29643300	-1.40768600
C	15.00794000	-5.26312800	1.40871000	C	14.97258900	-5.29643300	1.40768600
C	15.89976300	-4.46528500	0.73241200	C	15.88158000	-4.46813800	0.72812100
H	14.99733300	-5.24993500	-2.49645400	H	14.96303400	-5.28357000	-2.49518800
H	14.99733300	-5.24993500	2.49645400	H	14.96303400	-5.28357000	2.49518800
C	13.93524100	-5.92249300	-0.73020200	C	13.92808200	-5.94435000	-0.72884000
C	12.82393300	-6.37320400	-1.40852200	C	12.79685700	-6.41910800	-1.40813600
C	11.60445400	-6.65719000	-0.72834300	C	11.59456400	-6.69438300	-0.72942300
C	11.60445400	-6.65719000	0.72834300	C	11.59456400	-6.69438300	0.72942300
C	12.82393300	-6.37320400	1.40852200	C	12.79685700	-6.41910800	1.40813600
C	13.93524100	-5.92249300	0.73020200	C	13.92808200	-5.94435000	0.72884000
H	12.81684800	-6.35272400	-2.49613900	H	12.79281200	-6.40586300	-2.49564700
H	12.81684800	-6.35272400	2.49613900	H	12.79281200	-6.40586300	2.49564700
C	9.16163900	-6.66294500	-0.72702200	C	9.14193800	-6.70166800	-0.72896100
C	7.97013700	-6.41070000	-1.40699900	C	7.95025500	-6.45171300	-1.40762300
C	6.80832700	-6.00957400	-0.72617800	C	6.78303700	-6.03715600	-0.72658600
C	6.80832700	-6.00957400	0.72617800	C	6.78303700	-6.03715600	0.72658600
C	7.97013700	-6.41070000	1.40699900	C	7.95025500	-6.45171300	1.40762300
C	9.16163900	-6.66294500	0.72702200	C	9.14193800	-6.70166800	0.72896100
H	7.97518300	-6.39112400	-2.49461400	H	7.95342700	-6.43725700	-2.49519500
H	7.97518300	-6.39112400	2.49461400	H	7.95342700	-6.43725700	2.49519500
**Dcyc20_R**				**Dcyc20_U**			
C	2.07532100	2.17557700	1.40841100	C	2.07222200	2.18201000	1.40535900
C	5.62891800	5.54431800	1.40690800	C	5.61378500	5.57391100	1.40548600
C	5.62891800	5.54431800	-1.40690800	C	5.61378500	5.57391100	-1.40548600
C	6.71163900	6.13469200	-0.72668800	C	6.68227100	6.16378300	-0.72666700
C	6.71163900	6.13469200	0.72668800	C	6.68227100	6.16378300	0.72666700
C	1.41665500	1.20748800	0.72632100	C	1.41465600	1.20814200	0.72282000
C	2.07532100	2.17557700	-1.40841100	C	2.07222200	2.18201000	-1.40535900
H	5.63765000	5.52969600	-2.49459000	H	5.62106300	5.55977000	-2.49318600
C	7.85034900	6.58912400	-1.40732000	C	7.82853200	6.63310000	-1.40766600
C	7.85034900	6.58912400	1.40732000	C	7.82853200	6.63310000	1.40766600

H	7.85594800	6.57141700	-2.49494400	H	7.83224000	6.61998800	-2.49525600
H	7.85594800	6.57141700	2.49494400	H	7.83224000	6.61998800	2.49525600
C	1.41665500	1.20748800	-0.72632100	C	1.41465600	1.20814200	-0.72282000
H	2.10279900	2.15911500	2.49609700	H	2.09923800	2.16629300	2.49308700
H	5.63765000	5.52969600	2.49459000	H	5.62106300	5.55977000	2.49318600
H	2.10279900	2.15911500	-2.49609700	H	2.09923800	2.16629300	-2.49308700
C	9.02914400	6.90758800	0.72728400	C	9.00409900	6.95005900	0.72898500
C	9.02914400	6.90758800	-0.72728400	C	9.00409900	6.95005900	-0.72898500
C	5.62891800	-5.54431800	1.40690800	C	5.61378500	-5.57391100	1.40548600
C	4.62996400	-4.85603000	0.72627000	C	4.60627000	-4.87009700	0.72322200
C	3.68557100	-4.03983500	1.40664100	C	3.67741700	-4.05589500	1.40337000
C	4.62996400	-4.85603000	-0.72627000	C	4.60627000	-4.87009700	-0.72322200
C	3.68557100	-4.03983500	-1.40664100	C	3.67741700	-4.05589500	-1.40337000
C	2.85448400	-3.17812700	-0.72614700	C	2.84157100	-3.18332800	-0.72156300
C	2.85448400	-3.17812700	0.72614700	C	2.84157100	-3.18332800	0.72156300
C	5.62891800	-5.54431800	-1.40690800	C	5.61378500	-5.57391100	-1.40548600
C	2.07532100	-2.17557700	-1.40841100	C	2.07222200	-2.18201000	-1.40535900
C	2.07532100	-2.17557700	1.40841100	C	2.07222200	-2.18201000	1.40535900
H	5.63765000	-5.52969600	2.49459000	H	5.62106300	-5.55977000	2.49318600
H	5.63765000	-5.52969600	-2.49459000	H	5.62106300	-5.55977000	-2.49318600
C	1.41665500	-1.20748800	0.72632100	C	1.41465600	-1.20814200	0.72282000
C	1.41665500	-1.20748800	-0.72632100	C	1.41465600	-1.20814200	-0.72282000
C	0.79370200	0.00000000	-1.39919700	C	0.79410600	0.00000000	-1.39754500
C	0.79370200	0.00000000	1.39919700	C	0.79410600	0.00000000	1.39754500
H	3.69736900	-4.02749700	-2.49441300	H	3.68930900	-4.04246300	-2.49117900
H	3.69736900	-4.02749700	2.49441300	H	3.68930900	-4.04246300	2.49117900
H	2.10279900	-2.15911500	2.49609700	H	2.09923800	-2.16629300	2.49308700
H	2.10279900	-2.15911500	-2.49609700	H	2.09923800	-2.16629300	-2.49308700
H	1.10276300	0.00000000	-2.44836600	H	1.10414900	0.00000000	-2.44649100
H	1.10276300	0.00000000	2.44836600	H	1.10414900	0.00000000	2.44649100
C	18.61661400	0.00000000	1.40474100	C	18.54113200	0.00000000	1.40733300
C	18.47497100	-1.22678900	0.73520600	C	18.42659500	-1.22301100	0.72752400
C	18.16740300	-2.39974200	1.40628700	C	18.10085400	-2.40859700	1.40733600
C	18.47497100	-1.22678900	-0.73520600	C	18.42659500	-1.22301100	-0.72752400
C	18.16740300	-2.39974200	-1.40628700	C	18.10085400	-2.40859700	-1.40733600
C	17.59815300	-3.51627000	-0.73366400	C	17.56398700	-3.51225800	-0.72771800
C	17.59815300	-3.51627000	0.73366400	C	17.56398700	-3.51225800	0.72771800
C	18.47497100	1.22678900	0.73520600	C	18.42659500	1.22301100	0.72752400
C	18.61661400	0.00000000	-1.40474100	C	18.54113200	0.00000000	-1.40733300
H	18.16773000	-2.40033100	-2.49426200	H	18.08376800	-2.40219700	-2.49481300
C	16.89557700	-4.48800500	-1.40836800	C	16.84696300	-4.51199600	-1.40744200
C	16.89557700	-4.48800500	1.40836800	C	16.84696300	-4.51199600	1.40744200
H	16.88661000	-4.48105600	-2.49622000	H	16.83417500	-4.50098600	-2.49494900
H	16.88661000	-4.48105600	2.49622000	H	16.83417500	-4.50098600	2.49494900
C	18.47497100	1.22678900	-0.73520600	C	18.42659500	1.22301100	-0.72752400
H	18.62318100	0.00000000	2.49273000	H	18.52242900	0.00000000	2.49480900
H	18.16773000	-2.40033100	2.49426200	H	18.08376800	-2.40219700	2.49481300
H	18.62318100	0.00000000	-2.49273000	H	18.52242900	0.00000000	-2.49480900
C	10.25600200	7.07059800	1.40784800	C	10.23003800	7.12383000	1.40835600
C	14.99373900	6.03037600	1.40890900	C	14.95873100	6.07166600	1.40768100
C	14.99373900	6.03037600	-1.40890900	C	14.95873100	6.07166600	-1.40768100
C	15.98226200	5.35542100	-0.73151800	C	15.96593200	5.36622100	-0.72814500
C	15.98226200	5.35542100	0.73151800	C	15.96593200	5.36622100	0.72814500
C	10.25600200	7.07059800	-1.40784800	C	10.23003800	7.12383000	-1.40835600
C	16.89557700	4.48800500	-1.40836800	C	16.84696300	4.51199600	-1.40744200
C	16.89557700	4.48800500	1.40836800	C	16.84696300	4.51199600	1.40744200
H	10.25699100	7.05043000	2.49544500	H	10.23092900	7.11170400	2.49589800
H	10.25699100	7.05043000	-2.49544500	H	10.23092900	7.11170400	-2.49589800
C	17.59815300	3.51627000	0.73366400	C	17.56398700	3.51225800	0.72771800
C	17.59815300	3.51627000	-0.73366400	C	17.56398700	3.51225800	-0.72771800

C	18.16740300	2.39974200	-1.40628700	C	18.10085400	2.40859700	-1.40733600
C	18.16740300	2.39974200	1.40628700	C	18.10085400	2.40859700	1.40733600
H	14.98376900	6.01413300	-2.49659400	H	14.95149100	6.05884900	-2.49520800
H	14.98376900	6.01413300	2.49659400	H	14.95149100	6.05884900	2.49520800
H	16.88661000	4.48105600	2.49622000	H	16.83417500	4.50098600	2.49494900
H	16.88661000	4.48105600	-2.49622000	H	16.83417500	4.50098600	-2.49494900
H	18.16773000	2.40033100	-2.49426200	H	18.08376800	2.40219700	-2.49481300
H	18.16773000	2.40033100	2.49426200	H	18.08376800	2.40219700	2.49481300
C	10.25600200	-7.07059800	-1.40784800	C	10.23003800	-7.12383000	-1.40835600
C	10.25600200	-7.07059800	1.40784800	C	10.23003800	-7.12383000	1.40835600
H	10.25699100	-7.05043000	-2.49544500	H	10.23092900	-7.11170400	-2.49589800
H	10.25699100	-7.05043000	2.49544500	H	10.23092900	-7.11170400	2.49589800
C	13.85644600	6.56419700	-0.72965100	C	13.84731900	6.59616500	-0.72884200
C	12.70227400	6.90012600	-1.40852700	C	12.67351100	6.95279400	-1.40811700
C	11.46660100	7.07510500	-0.72823700	C	11.45166900	7.11981300	-0.72941900
C	11.46660100	7.07510500	0.72823700	C	11.45166900	7.11981300	0.72941900
C	12.70227400	6.90012600	1.40852700	C	12.67351100	6.95279400	1.40811700
C	13.85644600	6.56419700	0.72965100	C	13.84731900	6.59616500	0.72884200
H	12.69735300	6.87963900	-2.49612900	H	12.67106800	6.94027800	-2.49565000
H	12.69735300	6.87963900	2.49612900	H	12.67106800	6.94027800	2.49565000
C	15.98226200	-5.35542100	-0.73151800	C	15.96593200	-5.36622100	-0.72814500
C	14.99373900	-6.03037600	-1.40890900	C	14.95873100	-6.07166600	-1.40768100
C	14.99373900	-6.03037600	1.40890900	C	14.95873100	-6.07166600	1.40768100
C	15.98226200	-5.35542100	0.73151800	C	15.96593200	-5.36622100	0.72814500
H	14.98376900	-6.01413300	-2.49659400	H	14.95149100	-6.05884900	-2.49520800
H	14.98376900	-6.01413300	2.49659400	H	14.95149100	-6.05884900	2.49520800
C	13.85644600	-6.56419700	-0.72965100	C	13.84731900	-6.59616500	-0.72884200
C	12.70227400	-6.90012600	-1.40852700	C	12.67351100	-6.95279400	-1.40811700
C	11.46660100	-7.07510500	-0.72823700	C	11.45166900	-7.11981300	-0.72941900
C	11.46660100	-7.07510500	0.72823700	C	11.45166900	-7.11981300	0.72941900
C	12.70227400	-6.90012600	1.40852700	C	12.67351100	-6.95279400	1.40811700
C	13.85644600	-6.56419700	0.72965100	C	13.84731900	-6.59616500	0.72884200
H	12.69735300	-6.87963900	-2.49612900	H	12.67106800	-6.94027800	-2.49565000
H	12.69735300	-6.87963900	2.49612900	H	12.67106800	-6.94027800	2.49565000
C	9.02914400	-6.90758800	-0.72728400	C	9.00409900	-6.95005900	-0.72898500
C	7.85034900	-6.58912400	-1.40732000	C	7.82853200	-6.63310000	-1.40766600
C	6.71163900	-6.13469200	-0.72668800	C	6.68227100	-6.16378300	-0.72666700
C	6.71163900	-6.13469200	0.72668800	C	6.68227100	-6.16378300	0.72666700
C	7.85034900	-6.58912400	1.40732000	C	7.82853200	-6.63310000	1.40766600
C	9.02914400	-6.90758800	0.72728400	C	9.00409900	-6.95005900	0.72898500
H	7.85594800	-6.57141700	-2.49494400	H	7.83224000	-6.61998800	-2.49525600
H	7.85594800	-6.57141700	2.49494400	H	7.83224000	-6.61998800	2.49525600
C	4.62996400	4.85603000	-0.72627000	C	4.60627000	4.87009700	-0.72322200
C	3.68557100	4.03983500	-1.40664100	C	3.67741700	4.05589500	-1.40337000
C	2.85448400	3.17812700	-0.72614700	C	2.84157100	3.18332800	-0.72156300
C	2.85448400	3.17812700	0.72614700	C	2.84157100	3.18332800	0.72156300
C	3.68557100	4.03983500	1.40664100	C	3.67741700	4.05589500	1.40337000
C	4.62996400	4.85603000	0.72627000	C	4.60627000	4.87009700	0.72322200
H	3.69736900	4.02749700	-2.49441300	H	3.68930900	4.04246300	-2.49117900
H	3.69736900	4.02749700	2.49441300	H	3.68930900	4.04246300	2.49117900
C	-18.16740300	2.39974200	1.40628700	C	-18.10085400	2.40859700	1.40733600
C	-14.99373900	6.03037600	1.40890900	C	-14.95873100	6.07166600	1.40768100
C	-14.99373900	6.03037600	-1.40890900	C	-14.95873100	6.07166600	-1.40768100
C	-13.85644600	6.56419700	-0.72965100	C	-13.84731900	6.59616500	-0.72884200
C	-13.85644600	6.56419700	0.72965100	C	-13.84731900	6.59616500	0.72884200
C	-18.47497100	1.22678900	0.73520600	C	-18.42659500	1.22301100	0.72752400
C	-18.16740300	2.39974200	-1.40628700	C	-18.10085400	2.40859700	-1.40733600
H	-14.98376900	6.01413300	-2.49659400	H	-14.95149100	6.05884900	-2.49520800
C	-12.70227400	6.90012600	-1.40852700	C	-12.67351100	6.95279400	-1.40811700
C	-12.70227400	6.90012600	1.40852700	C	-12.67351100	6.95279400	1.40811700

H	-12.69735300	6.87963900	-2.49612900	H	-12.67106800	6.94027800	-2.49565000
H	-12.69735300	6.87963900	2.49612900	H	-12.67106800	6.94027800	2.49565000
C	-18.47497100	1.22678900	-0.73520600	C	-18.42659500	1.22301100	-0.72752400
H	-18.16773000	2.40033100	2.49426200	H	-18.08376800	2.40219700	2.49481300
H	-14.98376900	6.01413300	2.49659400	H	-14.95149100	6.05884900	2.49520800
H	-18.16773000	2.40033100	-2.49426200	H	-18.08376800	2.40219700	-2.49481300
C	-11.46660100	7.07510500	0.72823700	C	-11.45166900	7.11981300	0.72941900
C	-11.46660100	7.07510500	-0.72823700	C	-11.45166900	7.11981300	-0.72941900
C	-14.99373900	-6.03037600	1.40890900	C	-14.95873100	-6.07166600	1.40768100
C	-15.98226200	-5.35542100	0.73151800	C	-15.96593200	-5.36622100	0.72814500
C	-16.89557700	-4.48800500	1.40836800	C	-16.84696300	-4.51199600	1.40744200
C	-15.98226200	-5.35542100	-0.73151800	C	-15.96593200	-5.36622100	-0.72814500
C	-16.89557700	-4.48800500	-1.40836800	C	-16.84696300	-4.51199600	-1.40744200
C	-17.59815300	-3.51627000	-0.73366400	C	-17.56398700	-3.51225800	-0.72771800
C	-17.59815300	-3.51627000	0.73366400	C	-17.56398700	-3.51225800	0.72771800
C	-14.99373900	-6.03037600	-1.40890900	C	-14.95873100	-6.07166600	-1.40768100
C	-18.16740300	-2.39974200	-1.40628700	C	-18.10085400	-2.40859700	-1.40733600
C	-18.16740300	-2.39974200	1.40628700	C	-18.10085400	-2.40859700	1.40733600
H	-14.98376900	-6.01413300	2.49659400	H	-14.95149100	-6.05884900	2.49520800
H	-14.98376900	-6.01413300	-2.49659400	H	-14.95149100	-6.05884900	-2.49520800
C	-18.47497100	-1.22678900	0.73520600	C	-18.42659500	-1.22301100	0.72752400
C	-18.47497100	-1.22678900	-0.73520600	C	-18.42659500	-1.22301100	-0.72752400
C	-18.61661400	0.00000000	-1.40474100	C	-18.54113200	0.00000000	-1.40733300
C	-18.61661400	0.00000000	1.40474100	C	-18.54113200	0.00000000	1.40733300
H	-16.88661000	-4.48105600	-2.49622000	H	-16.83417500	-4.50098600	-2.49494900
H	-16.88661000	-4.48105600	2.49622000	H	-16.83417500	-4.50098600	2.49494900
H	-18.16773000	-2.40033100	2.49426200	H	-18.08376800	-2.40219700	2.49481300
H	-18.16773000	-2.40033100	-2.49426200	H	-18.08376800	-2.40219700	-2.49481300
H	-18.62318100	0.00000000	-2.49273000	H	-18.52242900	0.00000000	-2.49480900
H	-18.62318100	0.00000000	2.49273000	H	-18.52242900	0.00000000	2.49480900
C	-0.79370200	0.00000000	1.39919700	C	-0.79410600	0.00000000	1.39754500
C	-1.41665500	-1.20748800	0.72632100	C	-1.41465600	-1.20814200	0.72282000
C	-2.07532100	-2.17557700	1.40841100	C	-2.07222200	-2.18201000	1.40535900
C	-1.41665500	-1.20748800	-0.72632100	C	-1.41465600	-1.20814200	-0.72282000
C	-2.07532100	-2.17557700	-1.40841100	C	-2.07222200	-2.18201000	-1.40535900
C	-2.85448400	-3.17812700	-0.72614700	C	-2.84157100	-3.18332800	-0.72156300
C	-2.85448400	-3.17812700	0.72614700	C	-2.84157100	-3.18332800	0.72156300
C	-1.41665500	1.20748800	0.72632100	C	-1.41465600	1.20814200	0.72282000
C	-0.79370200	0.00000000	-1.39919700	C	-0.79410600	0.00000000	-1.39754500
H	-2.10279900	-2.15911500	-2.49609700	H	-2.09923800	-2.16629300	-2.49308700
C	-3.68557100	-4.03983500	-1.40664100	C	-3.67741700	-4.05589500	-1.40337000
C	-3.68557100	-4.03983500	1.40664100	C	-3.67741700	-4.05589500	1.40337000
H	-3.69736900	-4.02749700	-2.49441300	H	-3.68930900	-4.04246300	-2.49117900
H	-3.69736900	-4.02749700	2.49441300	H	-3.68930900	-4.04246300	2.49117900
C	-1.41665500	1.20748800	-0.72632100	C	-1.41465600	1.20814200	-0.72282000
H	-1.10276300	0.00000000	2.44836600	H	-1.10414900	0.00000000	2.44649100
H	-2.10279900	-2.15911500	2.49609700	H	-2.09923800	-2.16629300	2.49308700
H	-1.10276300	0.00000000	-2.44836600	H	-1.10414900	0.00000000	-2.44649100
C	-10.25600200	7.07059800	1.40784800	C	-10.23003800	7.12383000	1.40835600
C	-5.62891800	5.54431800	1.40690800	C	-5.61378500	5.57391100	1.40548600
C	-5.62891800	5.54431800	-1.40690800	C	-5.61378500	5.57391100	-1.40548600
C	-4.62996400	4.85603000	-0.72627000	C	-4.60627000	4.87009700	-0.72322200
C	-4.62996400	4.85603000	0.72627000	C	-4.60627000	4.87009700	0.72322200
C	-10.25600200	7.07059800	-1.40784800	C	-10.23003800	7.12383000	-1.40835600
C	-3.68557100	4.03983500	-1.40664100	C	-3.67741700	4.05589500	-1.40337000
C	-3.68557100	4.03983500	1.40664100	C	-3.67741700	4.05589500	1.40337000
H	-10.25699100	7.05043000	2.49544500	H	-10.23092900	7.11170400	2.49589800
H	-10.25699100	7.05043000	-2.49544500	H	-10.23092900	7.11170400	-2.49589800
C	-2.85448400	3.17812700	0.72614700	C	-2.84157100	3.18332800	0.72156300
C	-2.85448400	3.17812700	-0.72614700	C	-2.84157100	3.18332800	-0.72156300

C	-2.07532100	2.17557700	-1.40841100	C	-2.07222200	2.18201000	-1.40535900
C	-2.07532100	2.17557700	1.40841100	C	-2.07222200	2.18201000	1.40535900
H	-5.63765000	5.52969600	-2.49459000	H	-5.62106300	5.55977000	-2.49318600
H	-5.63765000	5.52969600	2.49459000	H	-5.62106300	5.55977000	2.49318600
H	-3.69736900	4.02749700	2.49441300	H	-3.68930900	4.04246300	2.49117900
H	-3.69736900	4.02749700	-2.49441300	H	-3.68930900	4.04246300	-2.49117900
H	-2.10279900	2.15911500	-2.49609700	H	-2.09923800	2.16629300	-2.49308700
H	-2.10279900	2.15911500	2.49609700	H	-2.09923800	2.16629300	2.49308700
C	-10.25600200	-7.07059800	-1.40784800	C	-10.23003800	-7.12383000	-1.40835600
C	-10.25600200	-7.07059800	1.40784800	C	-10.23003800	-7.12383000	1.40835600
H	-10.25699100	-7.05043000	-2.49544500	H	-10.23092900	-7.11170400	-2.49589800
H	-10.25699100	-7.05043000	2.49544500	H	-10.23092900	-7.11170400	2.49589800
C	-6.71163900	6.13469200	-0.72668800	C	-6.68227100	6.16378300	-0.72666700
C	-7.85034900	6.58912400	-1.40732000	C	-7.82853200	6.63310000	-1.40766600
C	-9.02914400	6.90758800	-0.72728400	C	-9.00409900	6.95005900	-0.72898500
C	-9.02914400	6.90758800	0.72728400	C	-9.00409900	6.95005900	0.72898500
C	-7.85034900	6.58912400	1.40732000	C	-7.82853200	6.63310000	1.40766600
C	-6.71163900	6.13469200	0.72668800	C	-6.68227100	6.16378300	0.72666700
H	-7.85594800	6.57141700	-2.49494400	H	-7.83224000	6.61998800	-2.49525600
H	-7.85594800	6.57141700	2.49494400	H	-7.83224000	6.61998800	2.49525600
C	-4.62996400	-4.85603000	-0.72627000	C	-4.60627000	-4.87009700	-0.72322200
C	-5.62891800	-5.54431800	-1.40690800	C	-5.61378500	-5.57391100	-1.40548600
C	-5.62891800	-5.54431800	1.40690800	C	-5.61378500	-5.57391100	1.40548600
C	-4.62996400	-4.85603000	0.72627000	C	-4.60627000	-4.87009700	0.72322200
H	-5.63765000	-5.52969600	-2.49459000	H	-5.62106300	-5.55977000	-2.49318600
H	-5.63765000	-5.52969600	2.49459000	H	-5.62106300	-5.55977000	2.49318600
C	-6.71163900	-6.13469200	-0.72668800	C	-6.68227100	-6.16378300	-0.72666700
C	-7.85034900	-6.58912400	-1.40732000	C	-7.82853200	-6.63310000	-1.40766600
C	-9.02914400	-6.90758800	-0.72728400	C	-9.00409900	-6.95005900	-0.72898500
C	-9.02914400	-6.90758800	0.72728400	C	-9.00409900	-6.95005900	0.72898500
C	-7.85034900	-6.58912400	1.40732000	C	-7.82853200	-6.63310000	1.40766600
C	-6.71163900	-6.13469200	0.72668800	C	-6.68227100	-6.16378300	0.72666700
H	-7.85594800	-6.57141700	-2.49494400	H	-7.83224000	-6.61998800	-2.49525600
H	-7.85594800	-6.57141700	2.49494400	H	-7.83224000	-6.61998800	2.49525600
C	-11.46660100	-7.07510500	-0.72823700	C	-11.45166900	-7.11981300	-0.72941900
C	-12.70227400	-6.90012600	-1.40852700	C	-12.67351100	-6.95279400	-1.40811700
C	-13.85644600	-6.56419700	-0.72965100	C	-13.84731900	-6.59616500	-0.72884200
C	-13.85644600	-6.56419700	0.72965100	C	-13.84731900	-6.59616500	0.72884200
C	-12.70227400	-6.90012600	1.40852700	C	-12.67351100	-6.95279400	1.40811700
C	-11.46660100	-7.07510500	0.72823700	C	-11.45166900	-7.11981300	0.72941900
H	-12.69735300	-6.87963900	-2.49612900	H	-12.67106800	-6.94027800	-2.49565000
H	-12.69735300	-6.87963900	2.49612900	H	-12.67106800	-6.94027800	2.49565000
C	-15.98226200	5.35542100	-0.73151800	C	-15.96593200	5.36622100	-0.72814500
C	-16.89557700	4.48800500	-1.40836800	C	-16.84696300	4.51199600	-1.40744200
C	-17.59815300	3.51627000	-0.73366400	C	-17.56398700	3.51225800	-0.72771800
C	-17.59815300	3.51627000	0.73366400	C	-17.56398700	3.51225800	0.72771800
C	-16.89557700	4.48800500	1.40836800	C	-16.84696300	4.51199600	1.40744200
C	-15.98226200	5.35542100	0.73151800	C	-15.96593200	5.36622100	0.72814500
H	-16.88661000	4.48105600	-2.49622000	H	-16.83417500	4.50098600	-2.49494900
H	-16.88661000	4.48105600	2.49622000	H	-16.83417500	4.50098600	2.49494900
